# Gas-phase Curtius and Wolff rearrangement reactions investigated by tandem-MS, IR ion spectroscopy and theory†

**DOI:** 10.1039/d5cp01532d

**Published:** 2025-06-10

**Authors:** Wacharee Harnying, Hui-Chung Wen, Jonathan Martens, Giel Berden, Jos Oomens, Jana Roithová, Albrecht Berkessel, Mathias Schäfer, Anthony J. H. M. Meijer

**Affiliations:** a Department of Chemistry, Institute of Organic Chemistry, University of Cologne Cologne Germany mathias.schaefer@uni-koeln.de; b Radboud University, Institute for Molecules and Materials, FELIX Laboratory Nijmegen The Netherlands; c van’t Hoff Institute for Molecular Sciences, University of Amsterdam Amsterdam The Netherlands; d Faculty of Sciences, Department of Spectroscopy and Catalysis, Radboud University Nijmegen The Netherlands; e Department of Chemistry, University of Sheffield Sheffield UK a.meijer@sheffield.ac.uk

## Abstract

The Curtius and the Wolff rearrangement reactions are investigated in the gas phase by tandem mass spectrometry (MS) and infrared ion spectroscopy (IRIS), probing the nature and intrinsic reactivity of three acyl azides and of one α-diazo keto analyte and that of their N_2_-loss products at temperatures around 300 K. Our study uses tailor-made precursor ions with innocent charge tags, which are activated upon collision-induced dissociation (CID). Our tandem-MS infrared ion spectroscopy (IRIS) study clearly evidences concerted N_2_-loss reactions delivering the ultimate reaction products of the Curtius reaction, *i.e.*, the isocyanates, and the ones of the Wolff reaction, *i.e.*, the ketenes. We show that this is fully consistent with the reaction mechanism predicted by quantum-chemical calculations. All IRIS data interpretation rests on computed linear IR spectra of ion structures identified by computational analysis based on DFT calculations with CCSD(T)-F12b energies.

## Abstract 2

The Curtius and the Wolff rearrangement reactions are investigated in the gas phase. Acyl azide and α-diazo keto analytes and respective N_2_-loss products are investigated by tandem mass spectrometry, infrared ion spectroscopy, and theory.

## Graphical abstract

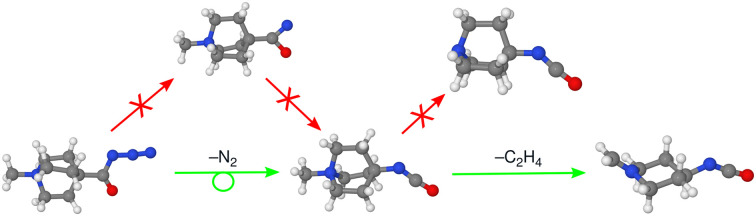

## Introduction

**Scheme 1:**
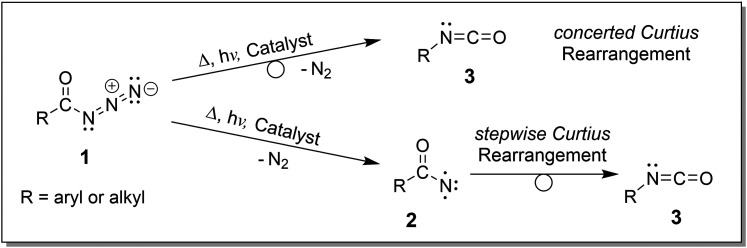
The Curtius rearrangement reaction of acyl azides 1 leads to the N_2_ release upon activation and ultimately yields isocyanates 3 either *via* acyl nitrene intermediates 2 (shown here in the triplet electronic state with two unpaired electrons) in a stepwise reaction or along a direct concerted reaction pathway.^3^

**Scheme 2:**
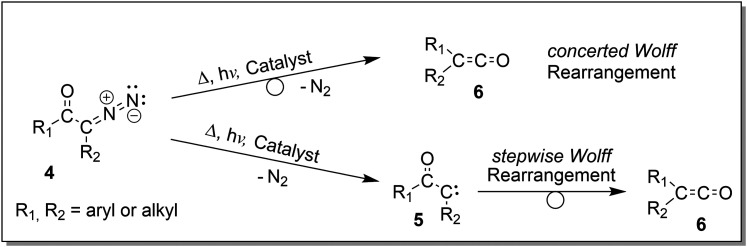
The Wolff rearrangement reaction of α-diazo ketone 4 leads to the N_2_ loss upon activation, generating α-keto carbene intermediate 5 with an electron sextet at the carbon atom, ultimately delivering ketenes 6 in a stepwise rearrangement reaction or along a direct concerted reaction pathway.^5,6^

In synthetic organic chemistry, acyl azides and α-diazo keto compounds are important precursors for the generation of isocyanates and ketenes, respectively.^1–12^ Both types of compounds are available from well-established transformations, *e.g.*, α-diazo keto compounds are synthesised by reaction of acid chlorides with diazomethane.^13^ Alternatively, they can be synthesised very safely, as described in the ESI,† (see Materials and synthesis Part I). In the condensed phase, the loss of nitrogen (N_2_) from acyl azides and α-diazo keto compounds can be achieved by thermal or photochemical activation, either directly or aided by catalysts.^1–12^ These so-called Curtius and Wolff reactions proceed either stepwise or concerted and yield isocyanates and ketenes as rearrangement products, respectively. This pronounced N_2_-loss of both types of compounds can be explained from the resonance structures of compounds 1 and 4, as Schemes 1 and 2 illustrate. It should be noted that ketenes cannot be isolated in the condensed phase as they are directly transformed into stable products. One prominent and important application of the Wolff rearrangement is the Arndt–Eistert homologation of carboxylic acids in which acyl chlorides are transformed with diazomethane to the respective ketenes and ultimately converted to their ester homologues by nucleophilic attack of an alcohol.^1,5,6,14^ The reaction mechanisms of the Curtius^3^ and Wolff rearrangement reactions,^5,6^ have been extensively investigated theoretically and experimentally, *e.g.*, with regard to the activation mode (heat, light, catalysis), the stereochemistry and the starting condition of the precursors (ground-state *vs.* excited-state reactivity).^15–19^ The monovalent acyl nitrene nitrogen in 2 and the divalent acyl carbene carbon in 5 both deviate from the octet rule and have an electron sextet, where the electronic spin state depends on the extent of stabilisation *via* inductive effects and resonance provided by adjacent substituents.^3,20,21^ For instance, trifluoroacetyl nitrene^16^ and the aromatic 2-formylaryl nitrene^17^ adopt triplet ground states,^15,21,22^ but other acyl nitrenes can also be found with singlet ground states.^23^ Acyl carbenes were identified as singlets in their ground state.^6,7^ Remarkably, acyl carbenes,^21,24^ and more recently, also acyl nitrenes^22,25^ have been detected in the solid state of a noble gas matrix by absorption spectroscopy after flash vacuum pyrolysis of appropriate precursors at cryogenic temperatures. In selected cases, carbenes and nitrenes were observed to rearrange with contributions of heavy atom quantum mechanical tunnelling (QMT), as indicated by temperature-independent rates and large kinetic isotope effects (KIEs).^16,25^ The contribution of QMT to the rearrangement reactivity of acyl nitrenes at cryogenic temperatures was also confirmed theoretically.^15^

The special nature of the nitrene and carbene reaction intermediates, as well as the labile nature of the rearrangement products, especially against hydrolysis, motivates us to study these reactions in a stepwise fashion in the gas phase *via* tandem-MS, without any influence of solvent, using an ion trap at room temperature.^26–28^ Additionally, the nitrenes and isocyanates, as well as the carbenes and ketenes, differ substantially in molecular structure and, therefore, should be distinguishable using vibrational action spectroscopy, even in mixtures.^26–28^ In tandem with the experimental studies, we also perform electronic structure calculations to find any potential intermediates accumulating during either the Curtius or Wolff rearrangements. The harmonic and anharmonic frequencies obtained from the optimised structures will give us a clear indication of the potential presence of these structures through comparison with the infrared spectra of the ions using previously reported procedures.^26–29^ As in ref. 27 and 28, we will use CCSD(T)-F12b calculations to determine the relative importance of the potential reaction pathways.

**Scheme 3:**
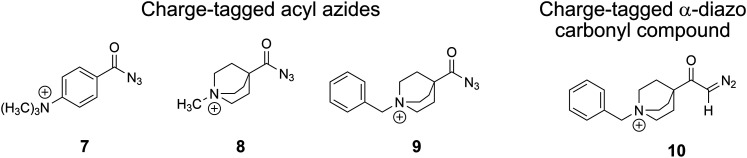
Remotely charge-tagged analyte cations for the gas-phase investigation of the Curtius and the Wolff rearrangement reactions by tandem-MS, IRIS and theory.

For this project, we synthesised a set of tailor-made acyl azides and one α-diazo keto analyte, which provide a permanent charge placed remotely from the reactive moieties, similar to our earlier studies,^26–28^ as presented in Scheme 3. All analytes are purposely designed and possess a stiff - either aliphatic or aromatic – backbone to prevent direct interaction of the positive charge itself or any polarised α-methylene protons with the carbene or nitrene atoms, an interaction that was found to effectively hamper carbene reactivity.^28^ In addition, we purposely included a benzylic substituent in the *N*-benzyl-4-quinuclidinium analyte ions 9 and 10 with the aim to effectively form [C_7_H_7_]^+^ upon photoactivation. This strategy was adopted from our recent study, which allowed an exceptionally sensitive acquisition of IRIS spectra.^28^

## Methods

### Materials

The three cationic acyl azides were freshly synthesised as a triflate 7 or as chlorides 8, 9, as detailed in the ESI,† Section I. The *N*-benzyl-4-quinuclidinium α-diazo keto analyte 10 was also derived from the respective chloride salt as summarised in the ESI,† (see Section I).

### Mass spectrometry

All charge-tagged analytes 7–10 were dissolved (*c* ∼10^−5^ M) in 1 mL CH_3_CN or in CH_3_OH, for (+)ESI-MS, MS^2^ and subsequent IRIS analysis. All (+)ESI, tandem-MS and accurate ion mass measurements were conducted on an LTQ-Orbitrap XL instrument (ThermoFisher, Bremen, Germany). Accurate ion mass measurements were executed in the orbitrap analyser with a resolution of at least 30 000 fwhm with external calibration (Δ*m* < 2 ppm) or with the addition of internal standards (Δ*m* < 1–2 ppm) by a lock mass procedure (see Table S1 in Part II on MS in the ESI†). The product-ion experiments upon CID to trigger the N_2_-loss reaction were performed in the linear ion trap (LTQ) part of the LTQ-Orbitrap XL instrument by CID with the He bath gas present (*P* = 2 × 10^−5^ Torr; 2.7 mbar) at individual normalised collision energies in the range from 10–26. The precursor ions and all product ions were analysed in the orbitrap (Fig. S24–S28, in Part II on MS, ESI†). Typical (+)ESI-MS conditions: flow rate: 5 μL min^−1^; capillary voltage: 3.20 kV; sheath gas: 4.99 [arb. units]; Aux gas: 2.00 [arb. units]; resolution: 30 000 fwhm.^26–28^ Additional MS-data and spectra are presented in the ESI.†

### Infrared ion spectroscopy

**Fig. 1:**
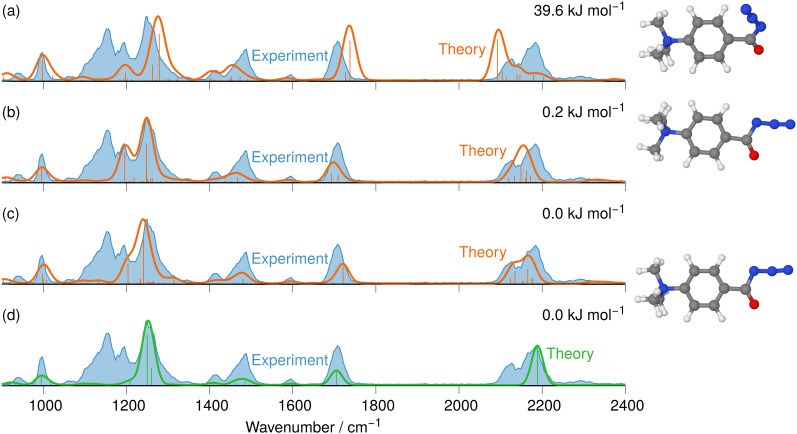
IR ion spectrum of the molecular ion of the aromatic carbonyl azide precursor 7 at *m/z* 205 (blue shadowed trace) compared with calculated, linear IR spectra of three structural alternatives: (a) isomer 3 anharmonic IR spectrum (39.6 kJ mol^−1^); (b) isomer 2 anharmonic IR spectrum (0.2 kJ mol^−1^); (c) isomer 1 anharmonic IR spectrum (0.0 kJ mol^−1^); (d) isomer 1 harmonic IR spectrum (0.0 kJ mol^−1^). The numerous combinations of fundamental modes considered in the anharmonic IR spectra are included as wavenumber lines with respective intensities in traces (a)–(c). The harmonic modes are included as lines in trace (d). Scaling of harmonic/anharmonic spectra below 2000 cm^−1^: 0.97/0.99. Scaling of harmonic/anharmonic spectra above 2000 cm^−1^: 0.95/0.955.

**Fig. 2:**
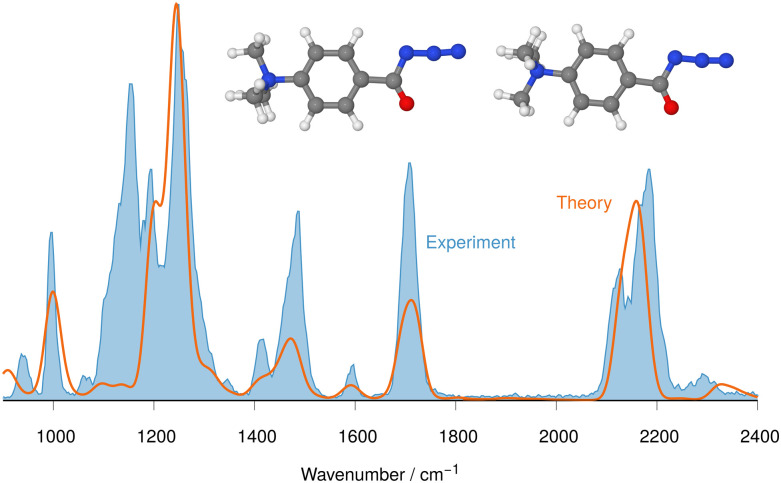
IRIS spectrum of the aromatic acyl azide precursor 7 at *m/z* 205 (blue shadowed trace) compared with the weighted average spectrum of isomer 1 (0.0 kJ mol^−1^) and isomer 2 (0.2 kJ mol^−1^) orange trace (anharmonic computations, traces (b) and (c) in Fig. 1). Scaling of below 2000 cm^−1^: 0.99. Scaling above 2000 cm^−1^: 0.955.

A modified 3D quadrupole ion trap mass spectrometer (Bruker, Amazon Speed) was used for the infrared (IR) ion spectroscopy study, which has been described in detail elsewhere.^30^ The 3D quadrupole ion trap was operated at ambient temperature (∼320 K) with He buffer gas at a pressure of ∼10^−3^ mbar. Wavelength tunable laser radiation was generated by the “Free Electron Laser for Infrared eXperiments” (FELIX) in the 800–2400 cm^−1^ range for all IRIS experiments.^30,31^ The FEL pulse energies were approximately 50–100 mJ per 5 μs long macropulse (at 10 Hz repetition frequency). The full width at half-maximum bandwidth of the FEL is approximately 0.4% of the central wavelength. Gas-phase precursor ions for IR ion spectroscopy were generated by electrospray ionisation in positive ion mode from 0.5 μM solutions of analyte in CH_3_CN at a flow rate of 120 μL h^−1^. The IR spectra result from a series of mass spectra recorded while the FEL was scanned over the wavenumber range from 800–2400 cm^−1^ irradiating the ions with a single FEL macropulse (see Fig. 1, 2, 4, 7, 9–11 and Fig. S29–S36 in Part III on IRIS in the ESI†). The depletion of the precursor ion signal and the increase of intensity of selected photodissociation product ion signals are monitored as a function of IR photon energy. Unimolecular dissociation results from the absorption of multiple IR photons (IRMPD) with effective intramolecular vibrational redistribution (IVR) of the excitation energy, leading to non-coherent photo activation until the threshold for dissociation is reached.^26–28^ The IR dissociation yield 
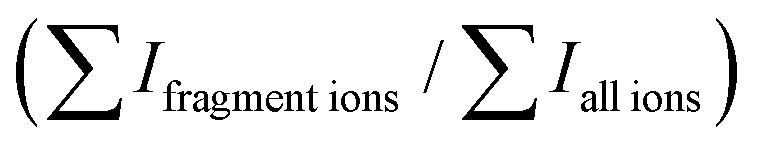
 was determined after laser irradiation at each frequency. The IRMPD intensity is equal to -ln(1-yield) and was linearly corrected for frequency-dependent variations in laser power.^32^ The photofragments of each precursor ion are listed in Table S2 the ESI,† (Part II on MS).

### Computational details

Density functional theory (DFT) calculations were performed using Gaussian16, version C.01.^33^ Gaussian was compiled with Gaussian-supplied versions of BLAS and ATLAS.^34,35^ The B3LYP^36^ functional was used throughout this study with the D3-BJ correction^37^ to account for dispersion interactions. The cc-pVTZ basis set^38^ was used throughout with the ultrafine setting for the integrals. This computational procedure is an improvement on the procedure from our earlier work, which already gave a good correlation with experiments^39–41^ and is consistent with the approach taken in ref. 27, 28 and 42. All calculations performed on these systems were done *in vacuo*. Minima were confirmed through the absence of any imaginary frequencies. Intrinsic reaction coordinate (IRC) calculations were performed with updated frequencies at every 3rd step of the profile to check the validity of the transition states, which were identified by a single imaginary frequency. CCSD(T)-F12b^43^ calculations were carried out on all singlet states for the analytes 7 and 8 and their descendents with the MOLPRO package of *ab initio* programs,^44,45^ using the cc-pVDZ-F12 basis set^43^ along with matching auxiliary fitting bases.^46^ The geminal Slater exponent was set to 1.0 a_0_^−1^. CCSD(T)-F12b calculations were attempted for a small sample of triplet geometries, but all showed a value for the t_1_ diagnostic indicative of multi-reference character.^47^ Hence, CASPT2 calculations^48^ were carried out on all singlet and triplet states for analytes 7 and 8 and their descendents with MOLPRO^44^ using the cc-pVTZ basis.^38^ For all calculations the active space was chosen such that all UHF natural orbitals with occupation numbers between 0.020 and 1.980 were included in the active space, since this achieved the best agreement between the CASPT2 and CCSD(T)-F12 calculations for the singlet states. A level-shift of .25 was used throughout to exclude intruder states. For all calculations on analytes 9 and 10 and their descendents, our computational resources only allowed B3LYP-D3(BJ)//cc-pVTZ calculations.

All ion energies are zero-point energy corrected with the B3LYP-D3(BJ) harmonic vibrational energies. The frequencies of the harmonic IR spectra (green traces in the figures) are scaled by a factor of 0.97 below 2000 cm^−1^ and by 0.95 above 2000 cm^−1^. For selected geometries, the anharmonically corrected frequences were calculated using the GVPT2 method with default parameters.^49^ These anharmonic spectra (orange traces in the figures) are scaled by a factor 0.99 below 2000 cm^−1^. Above 2000 cm^−1^ they are scaled by 0.955 for all azides and 0.985 for all nitrenes and isocyanates.^50,51^ Where a different scaling is used to obtain an even better agreement with the experimental data, this is indicated in the caption of the figure. The vibrational stick spectra are convoluted with a 25 cm^−1^ Gaussian broadening function to facilitate comparison with the experimental IR spectra. The ESI,† on computations was created by using in-house developed software based on the OpenEye toolkit.^52^ Images of molecules were created using Jmol (version 16.1.45)^53^ and POV-Ray (version 3.7).^54^

## Results and discussion

### Gas-phase investigation of the Curtius rearrangement

A set of three acyl azides was freshly synthesised from acid chlorides and were transferred to the gas-phase with electrospray ionisation (+ESI) to provide the cationic molecular ions 7–9 (Scheme 3) for IRIS experiments (see Part I in the ESI,† for details on the synthesis and Part II for the MS methods). The accurate ion masses of all three precursor ions as well as the tandem MS and IRIS analysis allowed a straightforward structure assignment (see Tables S1–S3 in the ESI,† for (+)ESI-MS, MS^2^ and IRIS details). The ion structures of the molecular ions 7–9 are assigned according to convincing agreement of IRIS data with IR spectra from anharmonic computations of ion structures proposed by theory, as discussed in the next section.

### Determination of ion structures of the charge-tagged acyl azide molecular ions 7–9 by IRIS and theory

The (+)ESI-MS IRIS spectra of the molecular ions of 7–9 were recorded and allow a convincing structure assignment. In particular, the characteristic azide stretching modes *v*_as_ R–N=N=N at around 2100–2200 cm^−1^ are instrumental for this analysis, as Fig. 1 and 2 for analyte 7 illustrate.

In Fig. S32 and S34 in the ESI,† the IRIS spectra of analytes 8 and 9 are compared to the respective computed IR spectra of ion structures proposed by theory. To achieve a realistic overlay of the computed IR bands with the acquired ones from IRIS, as outlined in the computational details, we apply two sets of scaling factors for either the harmonic and the anharmonic computed IR spectra. This approach allows us to correctly match the majority of absorption frequencies and intensities of the mainly bending, wagging and twisting modes in the lower wavenumber range at around 600–1500 cm^−1^ and of the substantial stretching modes above 2000 cm^−1^. Obviously, even with scaling, our calculations cannot correctly predict all vibrational transition energies and, hence, the band locations in the IR spectra. The apparent absence of some bands can be explained by the fact that our simulations model linear absorption spectra and not action spectra.^50,51^

The IRIS spectrum of the molecular ion of analyte 7 is shown in Fig. 1 (blue-shadowed trace). Therein, two bands are found in the wavenumber range 2100–2220 cm^−1^, in which the *v*_as_ R–N=N=N mode is expected to be found. This finding could suggest the presence of more than one species as the band positions of this mode in the IR spectra of the three conformers of 7 computed with a harmonic model do not match the experimental bands convincingly (see Fig. S29 in the ESI†). Alternatively, this could result from a Fermi resonance, which has also been found in other azides.^55,56^ This motivated us to compute anharmonic frequencies, which would allow us to decide between the two options. These calculations improved the agreement with the experimental data, as Fig. 1 and 2 show. The fine structure of the significant *v*_as_ R–N=N=N band at 2100–2220 cm^−1^ is perfectly matched by the IR spectrum of the ground structure of 7 shown adjacent to trace (c) in Fig. 1 (see also Table S22, ESI†). A more detailed analysis of this band shows that in the harmonic approximation, there is only a single contribution from the *v*_as_ R–N=N=N fundamental bond, as is clear from the sticks underpinning the convoluted spectrum in trace (d). In contrast, in the anharmonic calculations, this mode loses more than 75% of its intensity, making the major contributors to this band the combination bands between ring-deformation modes and ring breathing-CH-wagging modes, a phenomenon found earlier by us in unrelated systems.^57^ Overall, more than 10 lines make up this band in the anharmonic spectrum in trace (c). Similar observations can be made for all spectra below, hence only convoluted spectra will be reported to aid visibility and understanding.

The experimental IRIS spectrum is nicely matched by a weighted average of the spectra associated with the nearly isoenergetic conformers 1 (0.0 kJ mol^−1^) and 2 (0.2 kJ mol^−1^), which differ by a rotation of the NMe_3_-group, shown in Fig. 2. The only prominent mode less intense in the calculated spectra compared to the experimental spectrum of analyte 7 is found at around 1130 cm^−1^, as Fig. 2 illustrates. However, combination bands of C–N(CH_3_)_3_ stretching and HC–C–CH bending modes at 1095 cm^−1^ and of aromatic in-plane C–H bending and methyl C–H bending modes at 1128 cm^−1^ are predicted by theory in this wavenumber range, albeit with much lower intensity (see Tables S4 and S22 in the ESI†). This finding also holds for the IRIS spectra of the other two quinuclidinium acyl azide ions 8 and 9, in which this band at 1130 cm^−1^ is also present experimentally with a much lower predicted intensity. We note in this respect that the calculated IR spectra simulate exclusively linear absorption modes. The fact that the predicted intensities for these modes differ from the ones found in the IRIS spectra suggests that not only linear absorption modes contribute.

Apart from the mode at 1130 cm^−1^, the anharmonic spectra show an excellent agreement between the spectra for the molecular ions of the other two acyl azide analytes 8, 9 (see Tables S23 and S24, ESI†). The computed IR spectra of the most stable ion structures proposed by the theory are consistent with the IRIS spectra of the respective molecular ions as Fig. S32 and S34 (in Part III on IRIS in the ESI†) show. As for the acyl azide 7, the ground structure conformers for the acyl azide analytes 8 and 9 are also much more stable than other isomers. Therefore, the spectra of those other structures are not presented in Fig. S32 and S34 (as well as in Fig. S37 and S38 in Part III on IRIS in the ESI†).

### Gas-phase investigation of the N_2_-loss product ions, generated in MS^2^-product ion experiments, by IRIS and theory

It is known that upon activation with heat or light or in the presence of a catalyst, carbonyl azides release molecular nitrogen, and carbonyl nitrenes can be formed as important but elusive intermediates.^3,21^ In the condensed phase, the Curtius rearrangement yields isocyanates (Scheme 1).^1^

**Scheme 4:**
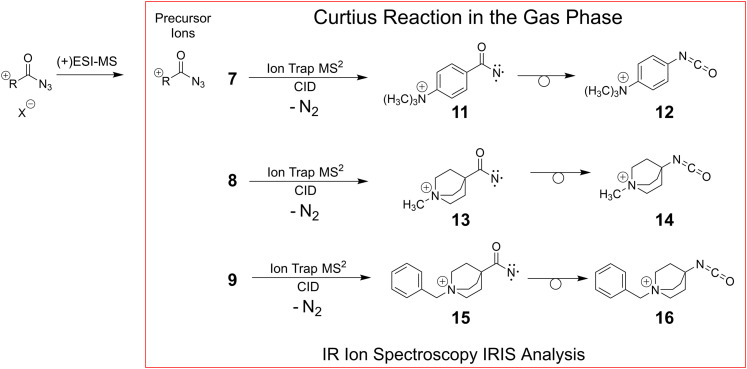
The three remotely charge-tagged acyl azide molecular ions 7–9 produced by (+)ESI-MS are collisionally activated in the gas phase of a quadrupole ion trap (see Fig. 3 and Fig. S24 and S26 in the ESI;† gas-phase chemistry in the red box). The nature of the product ions, *i.e.*, nitrenes or isocyanates, generated upon CID (potential product ions 11 or 12 of 7, 13 or 14 of 8, and 15 or 16 of 9), as well as the molecular structures of the acyl azide precursors 7–9 are investigated in the gas phase *via* IRIS.

For our gas-phase investigations, solutions of the freshly synthesised analyte salts (see Materials and synthesis Part I of the ESI,† for details) were prepared and the charge-tagged molecular ions of the carbonyl azides 7–9 were cleanly transferred from the condensed phase to the gas phase by positive electrospray ionisation, (+)ESI. The respective molecular ions are mass-selected and submitted to collisional activation in a quadrupole ion trap (QIT) to initiate nitrogen loss. The products of the N_2_-loss reaction are then mass-isolated and stored for gas-phase characterisation with IRIS as outlined in Schemes 3 and 4.

**Fig. 3:**
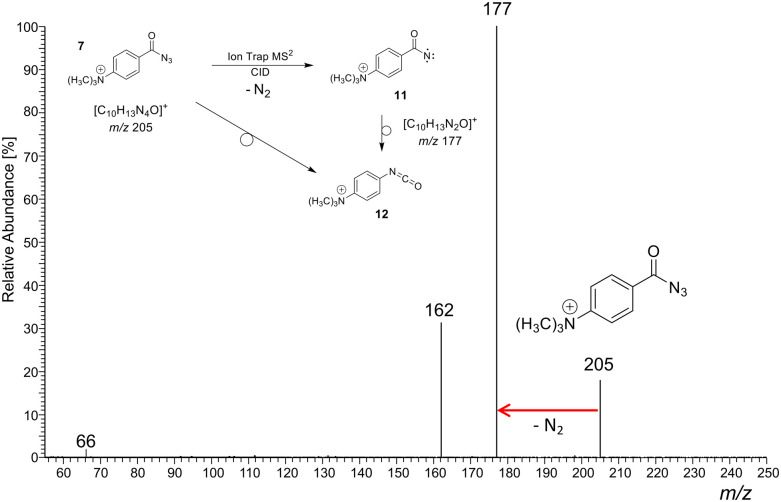
(+)ESI-MS^2^ product ion spectrum of the molecular ion of the aromatic acyl azide precursor 7 at *m*/*z* 205 upon collision activation in a linear ion trap. Ion detection was performed in an orbitrap (accurate ion masses are presented in Table S1, ESI†). Upon collision activation with He (normalised collision energy, NCE 13%), the carbonyl azide expels cleanly N_2_ and delivers either the respective nitrene 11 or the rearranged isocyanate 12 at *m/z* 177.^58–60^ The product ion at *m/z* 162 refers to an additional loss of a methyl radical ˙CH_3_. The precursor and product ions of the N_2_-loss are analysed by IRIS (see Fig. 1 and 4).

As a representative example for the gas-phase CID experiments, the MS^2^ product ion spectrum of the molecular ion of analyte 7 at *m/z* 205 is presented in Fig. 3. This analyte and the two other acyl azide precursor ions 8, 9 expel N_2_ as is evident from their MS^2^ product ion spectra (see Fig. 3 and Fig. S24, S26, respectively, ESI†). The accurate ion mass measurements provide evidence that N_2_ is lost and confirm the composition of the precursor and the [M-N_2_]^+^ product ions, which were submitted to IRIS analysis (see Tables S1 and S2 in the ESI†).

**Fig. 4:**
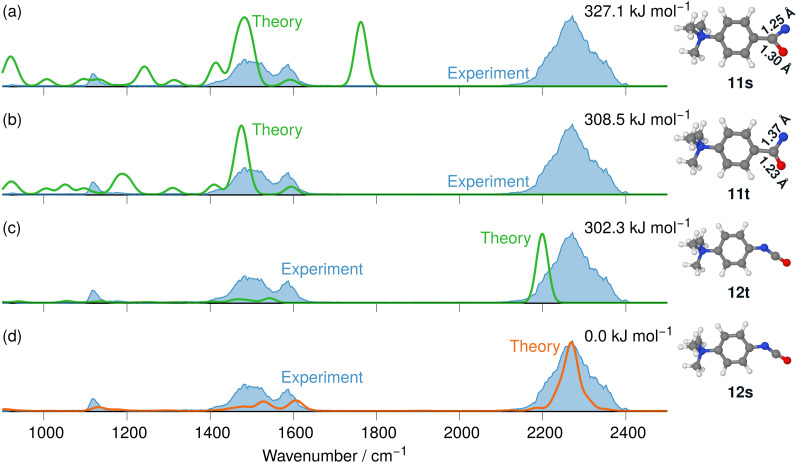
IR ion spectrum of the N_2_-loss product ion at *m/z* 177 of precursor ion 7 (blue shadowed trace) compared with calculated, IR spectra of four structural alternatives of the nitrene ions 11 and the isocyanate ions 12: (a) singlet nitrene 11s (harmonic; 327.1 kJ mol^−1^); (b) triplet nitrene 11t (harmonic; 308.5 kJ mol^−1^); (c) triplet isocyanate 12t (harmonic; 302.3 kJ mol^−1^); (d) singlet isocyanate 12s (anharmonic, 0.0 kJ mol^−1^, see also Table S25, ESI†). All energies evaluated at the CASPT2//cc-pVTZ level. Scaling of harmonic/anharmonic spectra below 2000 cm^−1^: 0.97/0.99. Scaling of harmonic/anharmonic spectra above 2000 cm^−1^: 0.95/0.985.

The IRIS spectrum of the N_2_-loss product ion at *m/z* 177 of precursor ion 7 is presented in Fig. 4, together with the calculated IR spectra of the respective nitrene and isocyanate ions 11 and 12 in both the singlet and the triplet electronic state. Inspection of the spectra in Fig. 4 demonstrates that the singlet isocyanate ion 12s, which has the lowest energy of all four structural alternatives, is the best match (see trace d in Fig. 4 and Tables S8–S10 in the ESI,† for detailed mode assignments; see Fig. S30 (ESI†) for the harmonic spectrum associated with 12s). Importantly, the isocyanate stretching mode *v*_R–N=C=O_ of singlet isocyanate 12s, which is predicted to be found around 2250 cm^−1^, is well represented in the IRIS spectrum (compare Table S8 in the ESI†). Both nitrenes show characteristic bending and stretching modes below 1600 cm^−1^ (see Tables S9 and S10 in the ESI†). However, the calculations also evidence that the electronic state still has a strong effect on the NC=O stretching mode of the nitrene moiety. For the singlet nitrene 11s this mode is found at 1243 cm^−1^, whereas for the triplet nitrene 11t this absorption is substantially blue-shifted to around 1476 cm^−1^ (modes in the harmonic calculations are scaled by 0.97), sensitively reflecting the C=O bond strength in the two electronic states, which is also evident from the change in bond length (see Fig. 4).

**Fig. 5:**
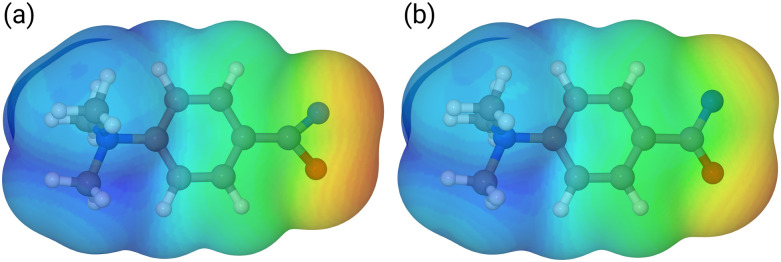
Mapped electrostatic potential (at a density of 0.0004) for 11s (panel (a)) and 11t (panel (b)). Colour map between 0.02 (red) *via* yellow and green to 0.18 (blue).

Our calculations show that the geometries for the singlet and triplet states are similar, apart from a larger NCO angle in the triplet state. This can qualitatively be explained by the change in electron distribution for the C(N)O group, where the molecular electrostatic potential (MEP) shown in Fig. 5 shows a shift of charge away from both oxygen and nitrogen onto the central carbon atom of the C(N)O group, with little effect on the rest of the molecule. Moreover, the calculation of partial charges for both 11s and 11t using the Merz–Kollman scheme shows a larger charge difference between the carbon and oxygen atoms in the C(N)O group for 11t (charge difference: 1.2e) compared to 11s (charge difference: 0.55e) in agreement with a stronger C(N)=O bond and a consequent blue-shift.^61,62^ Interestingly, the IR spectrum of the singlet nitrene 11s shows the characteristic OC=N stretch coupled with a C_6_H_5_–C(O)N stretching mode at 1763 cm^−1^, which is not found in the experimental IRIS spectrum nor in the spectrum of 11t, but which is consistent with the shorter (stronger) C=N bond in 11s, which is hence more oxaziridine-like. Finally, an isomer population analysis experiment was conducted at 2250 cm^−1^, where the isocyanate ions 12 absorb (*v*_R–N=C=O_ stretching mode), but the nitrenes are transparent.^63^ The ions are irradiated with 4 FEL pulses, which leads to a full depletion of the precursor ions at *m/z* 177 (Fig. S31, ESI†). Thus, all ions of *m/z* 177 absorb at 2250 cm^−1^. Hence, only singlet isocyanates ions 12 are present, formed by the gas-phase N_2_-loss reaction upon activation of analyte ion 7.

**Fig. 6:**
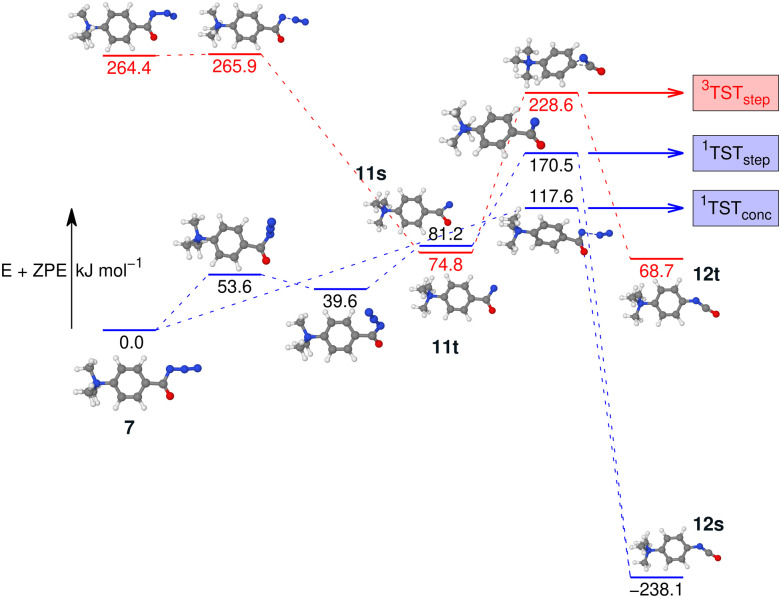
Potential energy surface (PES) of the N_2_-loss reaction of the aromatic carbonyl azide 7*via* the respective nitrenes 11s/11t in either a stepwise or a concerted Curtius rearrangement reaction to the isocyanates 12s/12t. The singlet energies are calculated with CCSD(T)-F12//cc-pVDZ-F12. The triplet energies are calculated at the CASPT2//cc-pVTZ level of theory. All geometry optimisations and vibrational frequency calculations used B3LYP-D3(BJ)//cc-pVTZ.

To investigate this further, the energy profile for the N_2_-loss reaction of the aromatic carbonyl azide 7 was calculated. Three pathways were considered: a stepwise Curtius rearrangement reaction on the singlet and triplet surface *via* nitrene intermediates 11s and 11t, respectively, as well as a concerted pathway on the singlet surface for direct formation of the isocyanate 12 from 7 (see Fig. 6). Our calculations show that the triplet nitrene 11t (+69.1 kJ mol^−1^ above the azide 7) is more stable than the singlet nitrene 11s (84.3 kJ mol^−1^ above the azide 7), an ordering also found in similar molecules.^16,21,22^ We appreciate that we are comparing two energies calculated using different methods, which was necessary as the triplet CCSD(T) calculations show considerable multi-reference character. However, this ordering is also evident if we either only consider B3LYP-D3(BJ) energies and frequencies or CASPT2//cc-pVTZ with B3LYP-D3(BJ) frequencies (see Fig. S37 and S38 in the ESI†) and, therefore, can be assumed an accurate reflection of the energy-ordering of the states.

Our calculations show two rate-determining transition states for the step-wise formation of the isocyanates 12s and 12t, labelled as ^1^TST_step_ and ^3^TST_step_, respectively, and a single rate-determining transition state for the concerted pathway leading to 12s, labelled as ^1^TST_conc_ in Fig. 6. The PES matches the experimental outcome as ^1^TST_conc_ at 116.4 kJ mol^−1^ is the lowest transition state found, considerably lower than ^1^TST_step_ for the step-wise reaction *via* the nitrene 11s or ^3^TST_step_ for the step-wise reaction *via* nitrene 11t. This explains the direct formation of the ultimate reaction products, *i.e.*, the isocyanate 12s evidenced by IRIS (see Fig. 4). The absence of any nitrene formation in the gas-phase tandem MS experiments is obviously related to the substantially less competitive pathways over substantially higher energy barriers of both the singlet and triplet surfaces towards the isocyanate product.

**Fig. 7:**
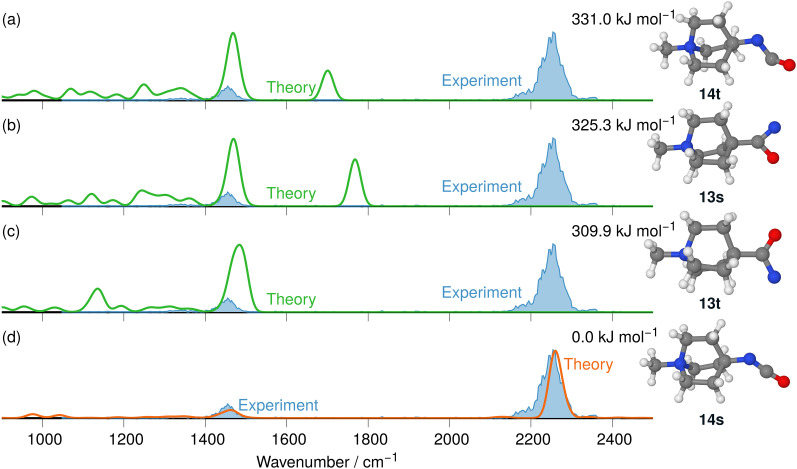
IR ion spectrum of the N_2_-loss product ion at *m/z* 167 of precursor ion 8 (blue shadowed trace), compared with the calculated IR spectra of four structural alternatives for the nitrene and the isocyanate ions 13 and 14: (a) IR spectrum of the triplet isocyanate 14t (harm. frequency calculation, +331.0 kJ mol^−1^); (b) singlet nitrene 13s (harm. frequency calculation, +325.3 kJ mol^−1^); (c) triplet nitrene 13t (harm. frequency calculation, +309.9 kJ mol^−1^); (d) singlet isocyanate 14s (anharm. frequency calculation, 0.0 kJ mol^−1^, Table S26, ESI†). All energies were evaluated at the CASPT2//cc-pVTZ level. Scaling of harmonic/anharmonic spectra below 2000 cm^−1^: 0.97/0.99. Scaling of harmonic/anharmonic spectra above 2000 cm^−1^: 0.95/0.985.

The IRIS ion spectrum of the N_2_-loss product ion at *m/z* 167 of precursor ion 8 is presented in Fig. 7. The harmonic and anharmonic IR spectra of the four structural alternatives of the respective nitrene and isocyanate ions 13 and 14 are compared to the IRIS spectrum (see Tables S11–S13 in the ESI,† for detailed mode assignments). Similar to the data set for precursor 7 discussed above, the anharmonic IR spectrum of the ground state ion structure, *i.e.*, the singlet isocyanate 14s, exhibits a convincing overall match in both frequency and intensity of all significant bands in the IRIS spectrum (see also Table S26, ESI†). It should be noted that the ordering of the isomers differs from the ordering starting from precursor 7. Structurally, similar conclusions can be drawn as for precursor 7, except that the triplet isocyanate 14t is now no longer linear. Finally, the exclusive presence of singlet isocyanate 14s is verified by an isomer population analysis at the photon energy of the isocyanate stretching mode, *i.e.* 2245 cm^−1^ (see Fig. S33 in the ESI,† for details).

To complement the spectroscopic data, a potential energy profile was also calculated for the N_2_-loss reactions of the N-methyl quinuclidinium acyl azide ion 8 leading to the singlet isocyanate 14, for both the singlet and triplet states, as presented in Fig. S39 in the ESI.† The calculations show that the energetic demand for the concerted rearrangement and the N_2_-loss is much lower than the stepwise reaction *via* the respective nitrene intermediates on either the singlet or triplet surface in line with the non-detection of either the singlet nitrene 13s or the triplet nitrene 13t as intermediates in the gas phase.

**Scheme 5:**
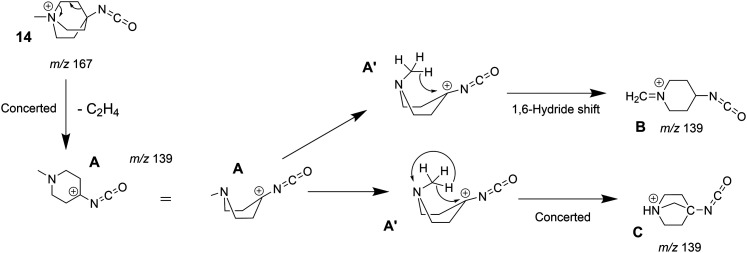
Fragmentation mechanism of the ethylene-loss observed in the MS^3^ product ion spectrum of the quinuclidinium isocyanate 14 (see Fig. S25, ESI†).

**Fig. 8:**
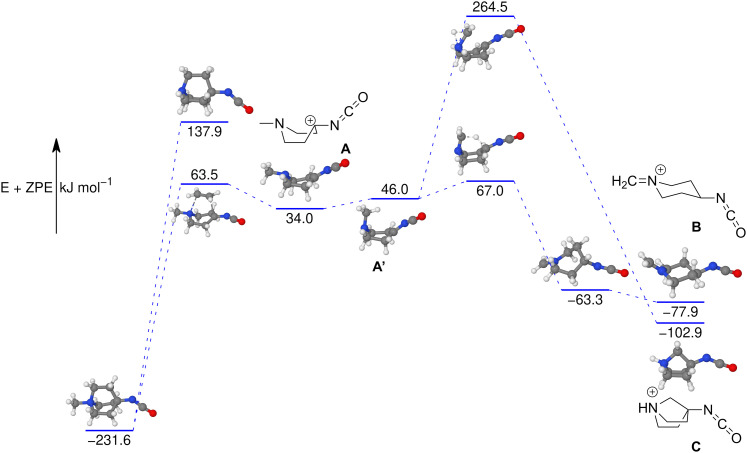
Potential energy surface of the C_2_H_4_-loss reaction of methyl quinuclidinium isocyanate 14s to the primary product A and subsequent rearrangement reactions leading to ions B and C (compare Scheme 5). The loss of a methyl radical from 14 is not competitive. The energies are calculated with CCSD(T)-F12//cc-pVDZ. All geometry optimisations performed and frequencies calculated using B3LYP-D3(BJ)//cc-pVTZ.

**Fig. 9:**
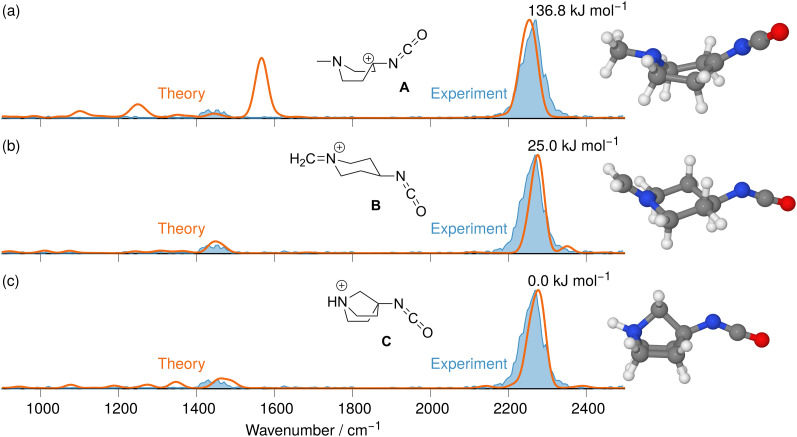
IR ion spectrum of the C_2_H_4_-loss product ion at *m/z* 139 generated from the N_2_-loss ion 14s at *m/z* 167 (blue shadowed trace) compared with the anharm. Calculated IR spectra of three structural alternatives: (a) IR spectrum of the C_2_H_4_-loss product ion A (+115.2 kJ mol^−1^); (b) 1,6-Hydride rearrangement product B (+15.8 kJ mol^−1^, Table S27, ESI†); (c) Bicyclic rearrangement product C (0.0 kJ mol^−1^, Table S28, ESI†). All energies were calculated using CCSD(T)-F12//cc-pVDZ using B3LYP-D3(BJ) geometries and vibrational energies. Scaling of harmonic/anharmonic spectra below 2000 cm^−1^: 0.97/0.99. Scaling of harmonic/anharmonic spectra above 2000 cm^−1^: 0.95/0.985.

The tandem-MS study of analyte 8 also showed that an interesting loss of C_2_H_4_ dominates the MS^3^ product ion spectrum of the isocyanate precursor ions 14 at *m/z* 167 (see Fig. S25 in the ESI†). This C_2_H_4_-loss product ion at *m/z* 139 was then investigated by IRIS and theory (see Table S1 in the ESI† for accurate ion mass). A fragmentation mechanism for the ethene-loss was developed, as shown in Scheme 5. The energy profile in Fig. 8 shows that three different product ions (labelled as A, B, and C) at *m/z* 139 are conceivable for the C_2_H_4_-loss from the isocyanate precursor ion 14 at *m/z* 167. The ethene-loss reaction of the isocyanate precursor ions 14 first leads to the piperidinium ion A at *m/z* 139 *via* a concerted reaction. From A, the *N*-methyl substituent can rotate from an equatorial to an axial position *via* a low-energy transition state, leading to A′. From A′, a subsequent intramolecular 1,6-hydride shift produces ions B. Alternatively, from A′ a 1,2-proton shift in concert with a ring closure reaction affords the formation of the bicyclic ion C (compare Scheme 5). The anharmonic IR spectra of the primary C_2_H_4_-loss product ion A, the secondary 1,6-hydride rearrangement product B, and also of the bicyclic rearrangement product C were compared with the IRIS data of *m/z* 139 in Fig. 9. Ions B and C show a convincing agreement with the IRIS spectrum. However, the kinetic product B is most likely solely present, as the higher energy barrier towards the thermodynamic product C should effectively prevent its formation, as shown in Fig. 8 (see Tables S20 and S21 for detailed mode assignments, ESI†). In contrast, a methyl-loss channel is clearly not competitive as it is barrierless and endothermic by 369.5 kJ mol^−1^.

**Fig. 10:**
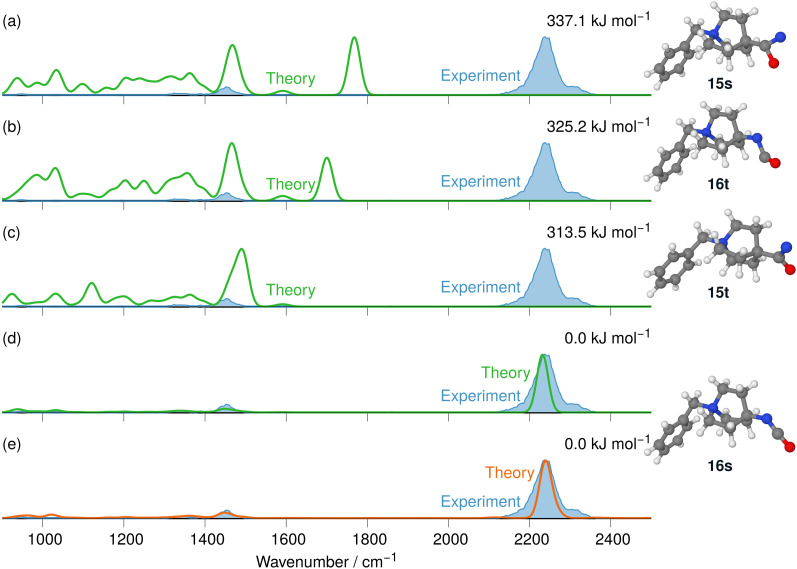
IR ion spectrum of the N_2_-loss product ion at *m/z* 243 (blue shadowed trace) compared with the calculated IR spectra of the nitrene and the isocyanate ions 15 and 16: (a) singlet nitrene 15s (337.1 kJ mol^−1^); (b) triplet isocyanate 16t (325.2 kJ mol^−1^); (c) triplet nitrene 15t (313.5 kJ mol^−1^); (d) linear IR spectrum of the singlet isocyanate 16s (0.0 kJ mol^−1^). Spectra (a)–(d) are the result of harm. computed frequencies. Trace (e) anharm. computed IR spectrum of singlet isocyanate 16s (0.0 kJ mol^−1^) for comparison (see also Table S29 (ESI†) for mode description of the significant anharmonic absorption band at 2237 cm^−1^). All energies were evaluated at the B3LYP-D3(BJ)//cc-pVTZ level. Scaling of harmonic/anharmonic spectra below 2000 cm^−1^: 0.97/0.99. Scaling of harmonic/anharmonic spectra above 2000 cm^−1^: 0.95/0.98.

The IRIS ion spectrum of the N_2_-loss product ion at *m/z* 243 of the precursor ion 9 is presented in Fig. 10. In the analysis of this *N*-benzyl-4-quinuclidinium acyl azide analyte ion, the benzylic substituent was of great advantage, as the effective formation of the [C_7_H_7_]^+^ upon photoactivation allowed a sensitive acquisition of the IRIS spectrum, shown in Fig. 10 (see Table S2 and also Fig. S27 in the ESI†). The anharmonically and harmonically calculated IR spectra of the four structural alternatives of the respective nitrene and isocyanate ions 15 and 16 are compared to the IRIS spectrum (see Tables S14–S16 in the ESI† for detailed mode assignments). Similar to the data set discussed above, the ground state ion structure, *i.e.*, the singlet isocyanate 16s, matches all significant bands found in the IRIS spectrum. In this case, the harmonic model is giving results that are comparable to the much more costly anharmonic computations (compare traces d and e in Fig. 10). Furthermore, the exclusive presence of singlet isocyanate 16s is verified by an isomer population analysis at the photon energy of the isocyanate stretching mode, *i.e.* at 2245 cm^−1^ (see Fig. S35 in the ESI† for details).

Finally, the PES of the N_2_-loss reaction of the *N*-benzyl quinuclidinium carbonyl azide 9 complemented the spectroscopic results (see Fig. S40 in the ESI†). The respective singlet 15s and triplet 15t nitrenes were not detected experimentally, which is consistent with the much lower energetic demand for the concerted Curtius rearrangement reaction to the singlet isocyanate 16s. It is interesting to note here that, for isocyanate 16s, the ethene-loss channel is not observed. This fits with our calculations, which show (at B3LYP-D3(BJ)-level of theory) that the transition state for the ethene-loss channel is 4.2 kJ mol^−1^ higher than the [C_7_H_7_]^+^-loss channel, which is 239.3 kJ mol^−1^ above the isocyanate 16s.

The stepwise reactions *via* the nitrene intermediates are not competitive for all acyl azide analytes 7–9 investigated in this study. Indeed, all three charge-tagged acyl azide analytes 7–9 show an analogous behaviour upon CID in the gas phase, which is consistent with similar relative energies, as well as with comparable barrier heights for N_2_-loss and for the rearrangement reaction pathways towards the respective isocyanate products of the Curtius reaction. This consistent outcome verifies the structural concept of the analyte design. The analogous results clearly document that the remotely attached charge tag is ‘innocent’ and not influencing the reactivity of the acyl azides. Moreover, it is reasonable to assume that aromatic and stiff aliphatic backbone structures allow an unperturbed mechanistic study of the degradation of acyl azide analytes *via* tandem-MS CID in the gas phase.^26–28,64^

### Gas-phase investigation of the N_2_-loss reaction and the Wolff rearrangement reaction by Tandem-MS, IRIS and theory

**Scheme 6:**
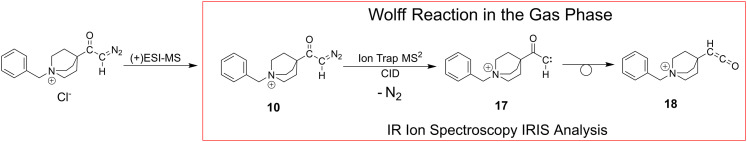
The Wolff rearrangement reaction is investigated in the gas phase with the charge-tagged benzyl quinuclidinium α-diazo ketone 10 (see Scheme 3), which loses N_2_ upon collisional activation (see Fig. S28 in the ESI†) to generate the α-keto carbene intermediate 17 with an electron sextet at the carbon, and ultimately the respective ketene 18. The Wolff reaction can proceed stepwise as shown or can lead *via* a concerted reaction pathway to the ketene as illustrated in Scheme 2.^6,7^

For the same reasons as outlined above for the Curtius case, we studied the Wolff rearrangement in the gas phase to characterise the intrinsic reactivity of charge-tagged α-diazo keto compounds and their N_2_-loss products, as Scheme 6 illustrates.

The IRIS ion spectrum of the charge-tagged *N*-benzyl-4-quinuclidinium α-diazo ketone 10 was analysed as shown in Fig. S36 in the ESI.† Two conformers of precursor ion 10 were identified by theory and their harmonic IR spectra were compared with the IRIS spectrum of the molecular ion at *m/z* 270. The harmonic IR spectrum of the ground structure of 10 matches all relevant bands of the experimental spectrum, and the abundance of the band around 1100 cm^−1^ is again underestimated (see also Fig. 1, 2, and Fig. S32, S34 in the ESI† and the discussion of that feature in the spectra of the acyl azide analytes 7–9).

The molecular ion of analyte 10 shows efficient N_2_-loss upon collisional activation, but we note a relatively high collision energy for the reaction to take place (NCE 20%; Fig. S28 in the ESI†).^58–60^ Whilst we note that assumptions solely based on NCE value comparisons are, at best, tentative, effective N_2_-loss was achieved at lower NCE values in the case of the acyl azide molecular ions 7–9 (typically NCE 12–13%, see Fig. 3 and Fig. S24, S26 in the ESI†).

**Fig. 11:**
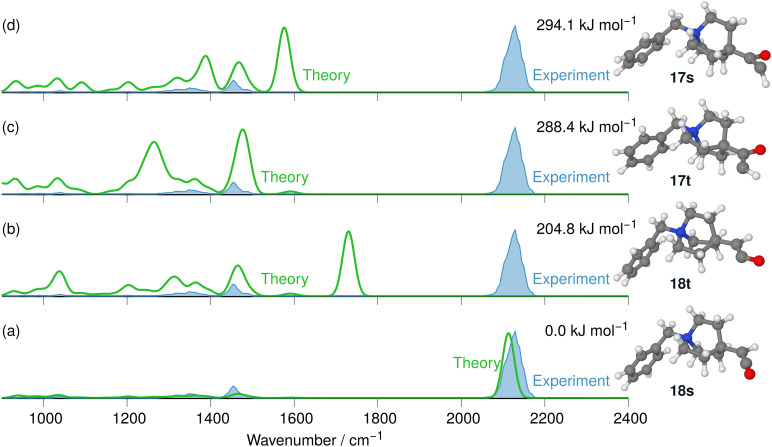
IR ion spectrum of the N_2_-loss product ion at *m/z* 242 of precursor ion 10 (blue shadowed trace) compared with the harmonic calculated IR spectra of four structural alternatives for carbene 17 and ketene 18 ions: (a) singlet carbene 17s (294.1 kJ mol^−1^); (b) triplet carbene 17t (288.4 kJ mol^−1^); (c) triplet ketene 18t (204.8 kJ mol^−1^); (d) singlet ketene 18s (0.0 kJ mol^−1^). All energies are evaluated at the B3LYP-D3(BJ)//cc-pVTZ level. Scaling of harmonic spectra below 2000 cm^−1^: 0.97. Scaling of harmonic spectra above 2000 cm^−1^: 0.95.

The respective product ions at *m/z* 242 are found with high intensity in the MS^2^ product ion spectrum of 10 and are analysed by IRIS (see Fig. 11). Additionally, the formation of the benzylic fragment ion [C_7_H_7_]^+^ at *m*/*z* 91 is also observed in Fig. S28 in the ESI.† This fragmentation channel was instrumental for the sensitive characterisation of the IRIS spectrum of the N_2_-loss ions at *m/z* 243 presented in Fig. 11. The harmonic IR spectra of the four structural alternatives of the respective carbene and ketene ions 17 and 18 are compared to the IRIS spectrum (see Tables S17–S19 in the ESI† for detailed mode assignments). Similar to the interpretation of the Curtius reaction products, the ground state ion structure, *i.e.*, the singlet ketene 18s, has all significant bands matching the IRIS spectrum of the ions at *m/z* 242. As the IR spectra computed with the harmonic model are sufficiently accurate and deliver a satisfactory agreement with the IRIS spectrum, anharmonic calculations are not deemed necessary. Finally, the exclusive presence of the singlet ketene 18s Wolff products was verified by a population analysis at the photon energy of the ketene stretching mode (2125 cm^−1^).

**Fig. 12:**
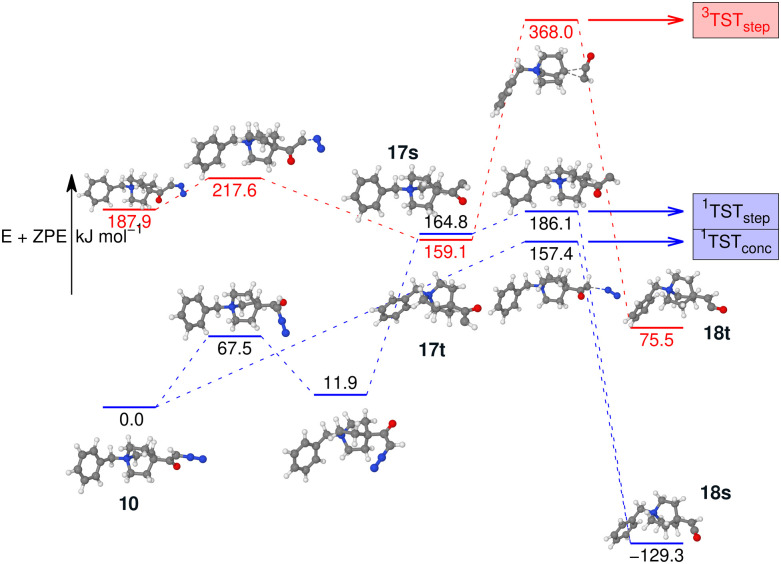
Potential energy surface of the N_2_-loss reaction of benzyl quinuclidinium α-diazo ketone 10*via* the respective carbenes 17s/17t in either a stepwise or a concerted Wolff rearrangement reaction to the singlet ketene 18. All optimisations, energies, and frequencies calculated using B3LYP-D3(BJ)//cc-pVTZ.

Finally, the PES of the N_2_-loss reaction of the *N*-benzyl-4-quinuclidinium α-diazo ketone 10 complemented the spectroscopic results (see Fig. 12). The respective singlet 17s (164.8 kJ mol^−1^) and triplet 17t carbene (159.1 kJ mol^−1^) were not detected experimentally, which is in obvious agreement with the much lower energetic demand for the concerted Wolff rearrangement reaction along the singlet surface to the singlet ketene 18. The stepwise reactions *via* the carbene intermediates are not competitive as their respective energy barriers are higher than the barrier for the concerted pathway. However, we do note that the barrier towards the concerted formation of the ketene is significantly higher than the corresponding barriers for the concerted formation of the isocyanate for 9, in agreement with the higher NCE for the formation of 18 compared to the NCE for the formation of 16. We did not investigate the ethene-loss channel in this case, as this MS^3^ pathway was not found in the experiments, which is consistent with our findings for isocyanate 16.

## Conclusions

In the present study on gas-phase Curtius and Wolff rearrangement reactions, carefully designed precursor ions exhibited effective N_2_-loss upon CID. The intrinsic reactivity of a set of acyl azides and of one α-diazo keto analyte and that of their N_2_-loss products was probed with tandem-MS, extensive calculations, and gas-phase IRIS spectroscopy. The analytical strategy and the molecular design of the acyl azides and the α-diazo ketone proved to be pertinent as the remotely positioned charge-tags attached to either aliphatic quinuclidine or aromatic backbones did not influence the rearrangement chemistry under investigation, as our study evidences. The analysis of all precursor ions and their respective N_2_-loss products with IR ion spectroscopy was complemented by DFT calculations, including the calculation of harmonic and anharmonic linear IR spectra followed up by either CASPT2 or CCSD(T)-F12b calculations, where affordable. The extensive set of calculations fully confirms the experimental results of the IRIS study. Thus, the preferred pathway for N_2_-loss in all cases follows the singlet surface with a concerted rearrangement reaction to the ultimate reaction products, which are the isocyanates resulting from the Curtius reaction and the ketenes generated by the Wolff rearrangement reaction. Moreover, these N_2_-loss reaction products were unambiguously identified in our study, whereby neither carbene nor nitrene intermediates could be detected. This uniform outcome of the gas-phase reactions triggered by collisional activation points towards photo activation as an alternative approach to enable effective N_2_-loss and to potentially allow access to the triplet surface and eventually to the triplet nitrene and triplet carbene intermediates. We are currently working on the design and the synthesis of analytes with appropriate absorption characteristics adjusted to the wavelength of the light source available for activation.

## Author contributions

WH and H-CW synthesised the analytes. JM, GB, and JO conducted the IRIS experiments and provided resources. JR conducted preliminary IRIS experiments and was involved in conceptualisation and funding acquisition. AB supervised the synthetic works and was involved in the conceptualisation. MS developed the analytical strategy, conducted the Tandem-MS and IRIS experiments together with JM and GB, was responsible for funding acquisition, data analysis, and project administration. AJHMM was responsible for the computational analysis of the data, was involved in conceptualisation, and visualisation. MS and AJHMM wrote the manuscript and were responsible for review and editing.

## Conflicts of interest

There are no conflicts to declare.

## Supplementary Material

CP-027-D5CP01532D-s001

## Data Availability

The data supporting this article have been included as part of the ESI.†
