# Supplementary material for: Gas-phase Curtius and Wolff rearrangement reactions investigated by tandem-MS, IR ion spectroscopy and theory
Source: Phys Chem Chem Phys. 2025 Jun 10;27(25):13543–56. doi: 10.1039/d5cp01532d (PMC12164736; doi:10.1039/d5cp01532d)
Supplement: CP-027-D5CP01532D-s001 [file CP-027-D5CP01532D-s001.pdf]

Supplementary Information (SI) for Physical Chemistry Chemical Physics.
This journal is © the Owner Societies 2025

Supporting Information

to

Gas-phase Curtius and Wolff rearrangement reactions investigated by tandem-MS, IR ion spectroscopy and theory

by

Wacharee Harnying, Hui-Chung Wen, Jonathan Martens, Giel Berden, Jos Oomens, Jana Roithová, Albrecht Berkessel, Mathias Schäfer and Anthony J.H.M. Meijer

Table of Contents

Part I

Materials and Synthesis

1. General **4**
2. Synthesis of 4-(trimethylammonio) benzoyl azide **7** **5**
3. Synthesis of N-methyl-4-quinuclidinium acyl azide **8** **6**
4. Synthesis of N-benzyl-4-quinuclidinium acyl azide **9** **8**
5. Synthesis of N-benzyl-4-quinuclidinium diazo carbonyl compound **10** **10**
6. NMR spectra **11**
7. IR spectra **21**

Part II

Mass Spectrometry

Table S1. (+)ESI-MS Accurate ion mass measurements of the precursor ions analyzed with IRIS. **23**
Table S2. Tandem-MS and Photofragmentation of the selected precursor ions used for IRIS. **24**
Figure S24. (+)ESI-MS^2 of the molecular ion of analyte **8** at *m/z* 195. **25**
Figure S25. (+)ESI-MS^3 of the product ion at *m/z* 167 formed from the molecular ion of analyte **8** at *m/z* 195. **26**
Figure S26. (+)ESI-MS^2 of the molecular ion of analyte **9** at *m/z* 271. **27**
Figure S27. (+)ESI-MS^3 of the product ion at *m/z* 243 formed from the molecular ion of analyte **9** at *m/z* 271. **28**
Figure S28. (+)ESI-MS^2 of the molecular ion of analyte **10** at *m/z* 270. **29**

Part III

IR Ion Spectroscopy IRIS

Table S3. Photofragmentation of the selected precursor ions used for IRIS. **30**

Figure S29. IR ion spectrum of the 4-(trimethylammonio) benzoyl azide **7** at *m/z* 205 compared with the calculated, linear IR spectra of three structural alternatives **31**

Figure S30. IR ion spectrum of the N_2-loss product ion at *m/z* 177 of precursor ion **7** compared with the calculated, IR spectra of the singlet isocyanate **12s**. **32**

Figure S31. Depletion scan at the $\nu_{as\ R-N=C=O}$ band of the isocyanate ions **12** at *m/z* 177 formed by (+)ESI-MS^2 of the molecular ion of analyte **7** at *m/z* 205. **33**

Figure S32. (+)ESI-MS IR ion spectrum of the N-methyl-4-quinuclidinium acyl azide precursor ion **8** at *m/z* 195 compared with the linear IR spectrum of the most stable ion structure (0.0 $kJmol^{-1}$). **34**

Figure S33. Depletion scan at the $\nu_{as\ R-N=C=O}$ band of the isocyanate ions **14** at *m/z* 167. **35**

Figure S34. (+)ESI-MS IR ion spectrum of the N-benzyl-4-quinuclidinium acyl azide precursor ion **9** at *m/z* 271 compared with the linear IR spectrum of the most stable ion structure (0.0 $kJmol^{-1}$). **36**

Figure S35. Depletion scan at the $\nu_{as\ R-N=C=O}$ band of the isocyanate ions **16** at *m/z* 243. **37**

Figure S36. IR ion spectrum of the molecular ion of the N-benzyl-4-quinuclidinium diazo carbonyl compound **10** at *m/z* 270 compared with the harmonic IR spectra of two ion structures. **38**

Table S4 Mode descriptions of the harmonic vibrational modes of the 4-(trimethylammonio) benzoyl azide **7.** **39**

Table S5: Mode descriptions of the harmonic vibrational modes of the N-methyl-4-quinuclidinium acyl azide **8.** **41**

Table S6: Mode descriptions of the harmonic vibrational modes of the N-benzyl-4-quinuclidinium acyl azide **9.** **42**

Table S7: Mode descriptions of the harmonic vibrational modes of the N-benzyl-4-quinuclidinium diazo carbonyl compound **10.** **43**

Table S8: Mode descriptions of the harmonic vibrational modes of the aromatic singlet nitrene **11s.** **44**

Table S9: Mode descriptions of the harmonic vibrational modes of the aromatic triplet nitrene **11t**. **45**

Table S10: Mode descriptions of the harmonic vibrational modes of the charge-tagged aromatic singlet isocyanate **12s.** **46**

Table S11: Mode descriptions of the harmonic vibrational modes of the singlet nitrene **13s.** **47**

Table S12: Mode descriptions of the harmonic vibrational modes of the triplet nitrene **13t.** **48**

Table S13: Mode descriptions of the harmonic vibrational modes of the singlet isocyanate **14s.** **49**

Table S14: Mode descriptions of the harmonic vibrational modes of the singlet nitrene **15s.** **50**

Table S15: Mode descriptions of the harmonic vibrational modes of the triplet nitrene **15t.** **51**

Table S16: Mode descriptions of the harmonic vibrational modes of the singlet isocyanate **16s.** **52**

Table S17: Mode descriptions of the harmonic vibrational modes of the singlet carbene **17s.** **53**

Table S18: Mode descriptions of the harmonic vibrational modes of the triplet carbene **17t.** **54**

Table S19: Mode descriptions of the harmonic vibrational modes of the singlet ketene **18s.** **55**

Table S20: Mode descriptions of the harmonic calculated vibrational modes of the 1-methylene-1-piperidinium isocyanate cation **B.** **56**

Table S21: Mode descriptions of the harmonic calculated vibrational modes of the 1-azabi-cyclo[2.2.1]heptanium isocyanate ion **C.** **57**

Table S22. Mode descriptions of the significant anharmonic absorption bands of isomer 1 of the charge-tagged aromatic carbonyl azide precursor ion **7**. **58**

Table S23. Mode descriptions of the significant anharmonic absorption bands of isomer 1 (0.0 $kJmol^{-1}$) of the charge-tagged methyl quinuclidinium carbonyl azide precursor ion **8**. **59**

Table S24. Mode descriptions of the significant anharmonic absorption bands of isomer 1 (0.0 $kJmol^{-1}$) of the charge-tagged benzyl quinuclidinium carbonyl azide precursor ion **9**. **60**

Table S25. Mode descriptions of the significant anharmonic absorption bands of the singlet aromatic isocyanate **12s** (0.0 $kJmol^{-1}$) important for the depletion scan. **61**

Table S26. Mode descriptions of the significant anharmonic absorption bands of the methyl quinuclidinium singlet isocyanate **14s** (0.0 kJmol^{-1}) important for the depletion scan. **61**
Table S27. Mode descriptions of the significant anharmonic absorption bands of the rearranged C_2H_4-loss product, the piperidinium isocyanate **B** (+15.8 kJmol^{-1}). **62**
Table S28. Mode descriptions of the significant anharmonic absorption bands of the rearranged C_2H_4-loss product at *m/z* 139, the isocyanate **C** (0.0 kJmol^{-1}). **63**
Table S29. Mode descriptions of the significant anharmonic absorption bands of the singlet benzyl quinuclidinium isocyanate **16s** (0.0 kJmol^{-1}) important for the depletion scan. **63**

Part IV

Calculations and Theory

Table of Contents **64-69**

Figure S37. Potential energy surface (PES) of the N_2-loss reaction of the aromatic carbonyl azide **7** via the respective nitrenes **11s/11t** in either a stepwise or a concerted Curtius rearrangement reaction to the isocyanate **12**. B3LYP-D3(BJ) level of theory. **70**
Figure S38. Potential energy surface (PES) of the N_2-loss reaction of the aromatic carbonyl azide **7** via the respective nitrenes **11s/11t** in either a stepwise or a concerted Curtius rearrangement reaction to the isocyanate **12**. CASPT2//def2tzvp with B3LYP-D3(BJ) level of theory. **71**
Figure S39. Potential energy surface of the N_2-loss reaction of methyl quinuclidinium carbonyl azide **8** via the respective nitrenes **13s/13t** in either a stepwise or a concerted Curtius rearrangement reaction to the singlet isocyanate **14**. Singlet surface computed on the B3LYP/cc-pVTZ and triplet surface computed on the B3LYP/cc-pVTZ level of theory. **72**
Figure S40. Potential energy surface of the N_2-loss reaction of benzyl quinuclidinium carbonyl azide **9** via the respective nitrenes **15s/15t** in either a stepwise or a concerted Curtius rearrangement reaction to the singlet isocyanate **16.** Computed on the B3LYP-D3(BJ)/cc-pVTZ level of theory. **73**
Cartesian coordinates of all computed structures **74-271**

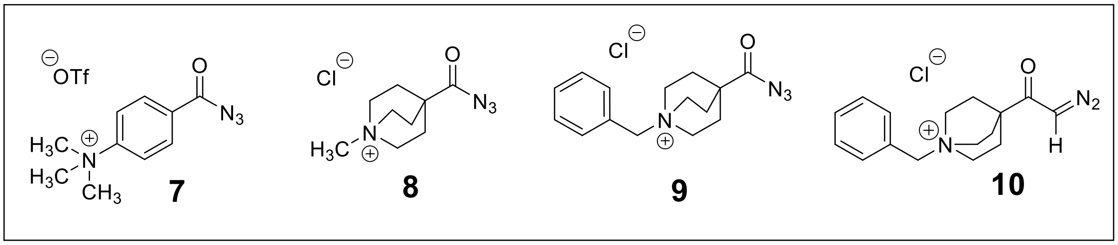

1. General

Chemicals and solvents were purchased from standard suppliers and used as received. (4-Dimethylamino)benzoic acid (**1**) was purchased from Merck-Sigma-Aldrich. Ethyl quinuclidine-4-carboxylate (**3**) was prepared according to a procedure described earlier.[1] (*N*-Isocyanoimino) triphenyl-phosphorane was purchased from *Thermo Fisher Scientific.* Dry DCM for reactions under anhydrous conditions was purchased from *Acros Organic.* Silica gel 60 (0.035-0.070 mm, 60 Å pore size) from *Macherey-Nagel* was used for flash column chromatography. Pressure was applied during packing and elution. For TLC analysis, *ALUGRAM Xtra SIL G/UV254* plates (0.2 mm silica layer, fluorescence indicator) from *Macherey-Nagel* were used. UV light analysis or phosphomolybdic acid (PMA) in ethanol solution were used for detection. All glassware was dried in an oven at 75 °C before use. *Schlenk* tubes/flasks were additionally dried with a heat gun, under argon, while being connected to a *Schlenk* line.

Nuclear magnetic resonance (NMR) spectra were recorded on a Bruker Avance 300 instrument (^{1}H: 300.13 MHz, ^{13}C: 75.46 MHz, ^{19}F: 282.40 MHz), or on a Bruker AV 500 instrument (^{1}H: 500.13 MHz, ^{13}C: 125.76 MHz) at ambient temperature. Chemical shifts (δ) are reported in parts per million (ppm) relative to tetramethylsilane (TMS) or solvent residual signals. The following abbreviations were used for chemical shift multiplicities in ^{1}H NMR spectra: br = broad, s = singlet, d = doublet, t = triplet, q = quartet, quint = quintet, sext = sextet, sept = septet, m = multiplet, m_c = centered multiplet. IR spectra were recorded on a Shimadzu IR Affinity-1 FT-IR spectrometer. Elemental analyses were performed using an Elementar Vario MICRO cube elemental analyzer. Melting points were determined on a Büchi apparatus and are uncorrected.

2. Synthesis of the 4-(trimethylammonio)benzoyl azide 7

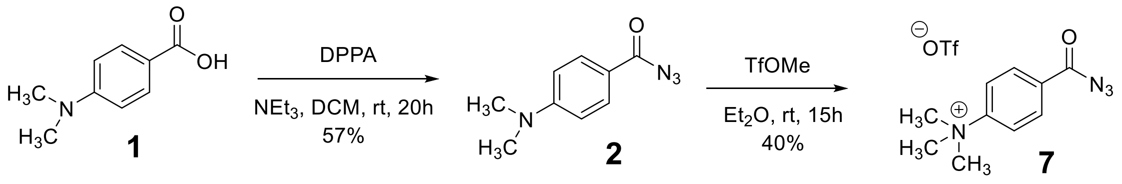

To a suspension of the carboxylic acid **1** (0.58 g, 3.5 mmol, 1.0 equiv) in dry DCM (10 mL) was added triethylamine (0.55 mL, 3.8 mmol, 1.1 equiv) under argon atmosphere, resulting in dissolution and a homogeneous reaction mixture. Diphenylphosphoryl azide (DPPA, 0.81 mL, 3.5 mmol, 1.0 equiv) was added dropwise and the reaction mixture was stirred at rt for 20h. The reaction mixture was then washed with H_2O (3x), dried over anhydrous Na_2SO_4, filtered, and evaporated under reduced pressure. Et_2O was added and the solvent was removed on a rotary evaporator. This operation was repeated several times for the azeotropic removal of DCM. The remaining off-white solid was re-dissolved in Et_2O and filtered through a short layer of silica gel. After removal of the solvent, the product **2** was obtained as an off-white solid (0.38 g, 2.0 mmol, 57%); mp 99–100 °C; ^{1}H NMR (300 MHz, $CDCl_3$): δ (ppm) = 8.01 – 7.79 (m, 2H, 2xArH), 6.73 – 6.56 (m, 2H, 2xArH), 3.08 (s, 6H, $2xCH_3$); ^{13}C NMR (75 MHz, $CDCl_3$): δ (ppm) = 171.5 (C=O), 154.3 (ArC), 131.5 (2xArCH), 117.4 (ArC), 110.7 (2xArCH), 40.0 ($2xCH_3$); IR (ATR): $\tilde{\nu}$ [cm^{-1}] = 2913 (w), 2129 (m), 1665 (m), 1597 (m), 1528 (m), 1371 (m), 1125 (s), 1007 (s), 1261 (s), 1200 (s), 1169 (s), 972 (s), 941 (s), 814 (s), 748 (s), 681 (s).

To a solution of the benzoyl azide **2** (95 mg, 0.5 mmol, 1.0 equiv) in dry Et_2O (5 mL) was added methyl trifluoromethanesulfonate (0.06 mL, 0.5 mmol, 1.0 equiv) under argon atmosphere, resulting in a white solid precipitate. The reaction mixture was stirred at rt for 2h and diluted with Et_2O. The solid was collected by suction filtration and washed with Et_2O to give the ammonium salt **7** as a colorless solid (72 mg, 0.2 mmol, 40%); mp 123–124 °C; ^{1}H NMR (300 MHz, acetone-d_6): δ (ppm) = 8.32 (d, J = 9.3 Hz, 2H, 2xArH), 8.24 (d, J = 9.3 Hz, 2H, 2xArH), 3.95 (s, 9H, $3xCH_3$); ^{13}C NMR (70 MHz, acetone-d_6): δ (ppm) = 171.7 (C=O), 152.4 (ArC), 133.2 (ArC), 132.0 (2xArCH), 122.4 (2xArCH), 57.8 ($3xCH_3$); ^{19}F NMR (282 MHz, acetone-d_6): δ (ppm) = 78.9 (CF_3); IR (ATR): $\tilde{\nu}$ [cm^{-1}] = 3053 (w), 2143 (m), 1684 (m), 1605 (w), 1495 (w), 1420 (w), 1254 (s), 1169 (s), 1028 (s), 1001 (m), 872 (w), 758 (m), 685 (m), 635 (s).

3. Synthesis of the N-methyl-4-quinuclidinium acyl azide 8

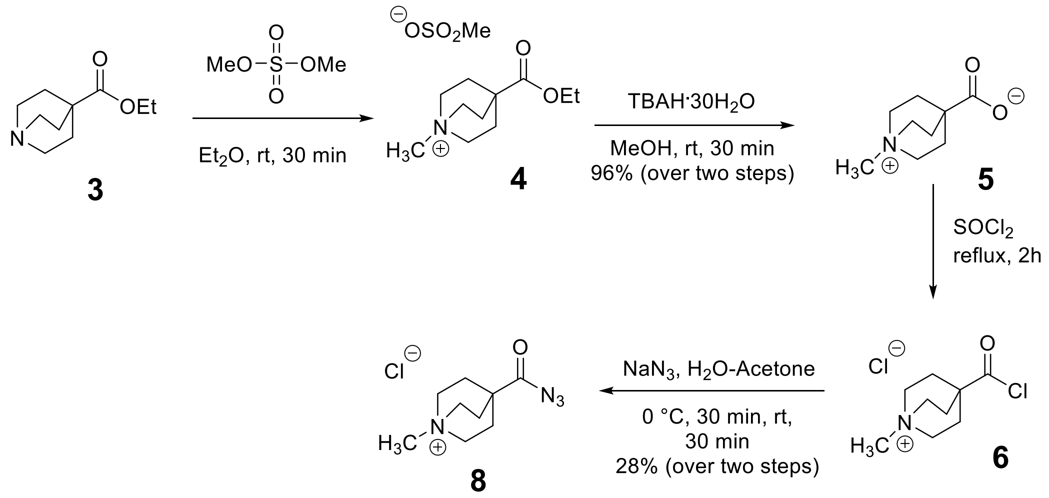

3.1 Synthesis of the zwitterion 5

To a solution of the ester **3** (458 mg, 2.5 mmol, 1.0 equiv) in dry Et_2O (10.0 mL) was added dimethyl sulfate (DMS, 0.35 mL, 3.75 mmol, 1.5 equiv) under argon atmosphere, resulting in a white precipitate. After stirring at rt for 30 min, the solvent was decanted. The remaining residue was triturated with Et_2O with vigorous stirring, to give **4** as a colorless solid, which was highly hygroscopic; ^{1}H NMR (300 MHz, $CDCl_3$): δ (ppm) = 4.16 (q, J = 7.1 Hz, 2H, OCH_2), 3.74 – 3.65 (m, 6H, 3xCH_2), 3.19 (s, 3H, OSO_2CH_3), 2.34 – 2.12 (m, 6H, 3xCH_2), 1.26 (t, J = 7.1 Hz, 3H, CH_3); ^{13}C NMR (75 MHz, $CDCl_3$): δ (ppm) = 173.2 (C=O), 61.6 (OCH_2), 56.2 (3xCH_2), 54.4 (CH_3), 51.7 (CH_3), 34.8 (C_q), 26.5 (3xCH_2), 14.0 (CH_3). The product **4** was used in the next step without further purification.

The ester **4** (ca. 2.5 mmol, 1.0 equiv) was dissolved in MeOH (2.5 mL) and tetrabutyl-ammoniumhydroxide (TBAH•30H_2O, 2.1 g, 2.5 mmol, 1.0 equiv) was added. The resulting homogenous solution was stirred at rt for 1h. The solvent was removed on a rotary evaporator under reduced pressure. Toluene was added and the solvent was again evaporated on a rotary evaporator, for the azeotropic removal of water. The semi-solid residue was then triturated with DCM with vigorous stirring. The solid was collected by suction filtration and washed with DCM to give **5** as a colorless solid (407 mg, 2.4 mmol, 96%); mp > 280 °C; ^{1}H NMR (300 MHz, D_2O): δ (ppm) = 3.59 – 3.35 (m, 6H, 3xCH_2), 2.95 (s, 3H, CH_3), 2.22 – 1.97 (m, 6H, 3xCH_2); ^{13}C NMR (75 MHz, D_2O) δ 181.7 (C=O), 56.8 (3xCH_2), 51.4 (CH_3), 35.7 (C_q), 26.8 (3xCH_2); IR (ATR): $\tilde{\nu}$ [cm^{-1}] = 3345 (br), 1568 (s), 1559 (s), 1472 (w), 1373 (s), 1339 (m), 1003 (w), 835 (m), 779n (m); elemental analysis calcd (%) for $C_9H_{15}NO_2 \bullet H_2O$: C, 57.73; H, 9.15; N, 7.48; found: C, 57.69; H, 8.76; N, 7.23.

3.2 Conversion of the zwitterion 5 to the acyl azide 8

The zwitterion **5** (170 mg, 1.0 mmol, 1.0 equiv) was refluxed in $SOCl_2$ (1.0 mL) for 2h. The excess of $SOCl_2$ was distilled off in vacuo and collected in a trap cooled with liquid nitrogen. The residue was further dried in high vacuum to give the corresponding acid chloride **6** as a colorless solid, which was used in the next step without further purification.

A solution of NaN_3 (57 mg, 0.8 mmol, 1.0 equiv) in H_2O (1.0 mL) was cooled in an ice-water bath. A suspension of the acid chloride **6** obtained above in acetone (4.0 mL) was added slowly at 0 °C. The reaction mixture was stirred at 0 °C for 30 min and then at rt for 30 min. The solvent was evaporated under reduced pressure. Toluene was added and the solvent was again removed on a rotary evaporator. The solid residue was washed with DCM and the solvent was decanted. The remaining residue was extracted several times with acetone with vigorous stirring. The combined organic phases were filtered and evaporated under reduced pressure. Toluene was added and the solvent was evaporated again on a rotary evaporator. The remaining material was dried further under high vacuum. The product **8** was obtained as a colorless amorphous solid (64 mg, 0.28 mmol, 28%) which was highly hygroscopic, liquification occured upon contact with air; ^{1}H NMR (300 MHz, CD_3CN): δ (ppm) = 3.67 – 3.57 (m, 6H, 3xCH_2), 3.07 (s, 3H, CH_3), 2.18 – 2.09 (m, 6H, 3xCH_2); ^{13}C NMR (75 MHz, CD_3CN): δ (ppm) = 182.2 C=O), 56.9 (3xCH_2), 52.4 (CH_3), 37.8 (C_q), 26.9 (3xCH_2); IR (ATR): $\tilde{\nu}$ [cm^{-1}] = 3352 (br), 2974 (w), 2149 (m), 1694 (s), 1466 (m), 1211 (s), 1188 (s), 1038 (m), 943 (s), 928 (s), 868 (m), 841 (m).

4. Synthesis of N-benzyl-4-quinuclidinium acyl azide 9

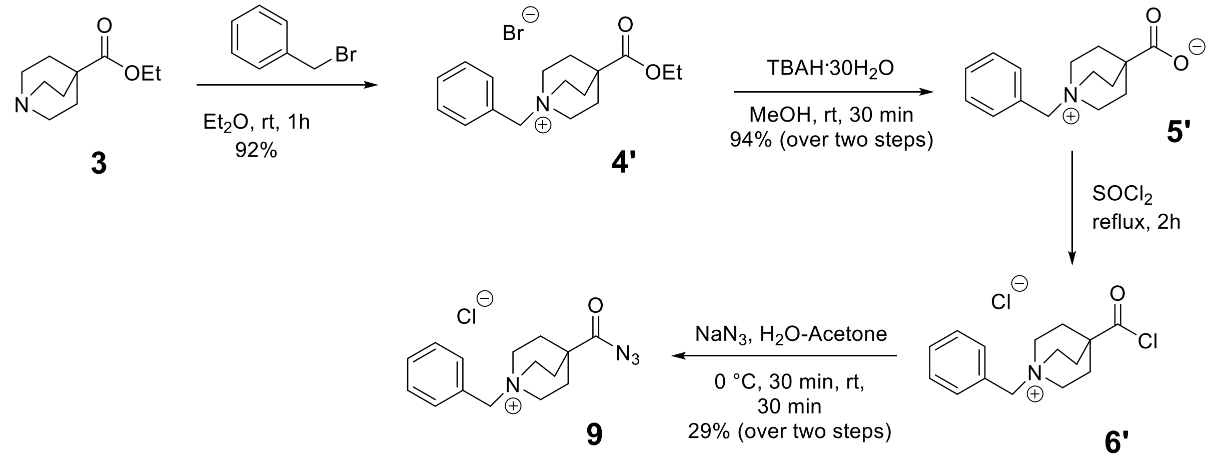

4.1 Synthesis of the zwitterion 5’

To a solution of the ester **3** (515 mg, 2.8 mmol, 1.0 equiv) in dry Et_2O (9.0 mL) was added benzyl bromide (0.50 mL, 4.2 mmol, 1.5 equiv) under argon atmosphere, resulting in a white precipitate. After stirring at rt for 1h, the solid was collected by suction filtration and washed with Et_2O to give **4’** as a colorless solid (913 mg, 2.6 mmol, 92%); mp 183–185 °C; ^{1}H NMR (300 MHz, $CDCl_3$): δ (ppm) = 7.71 (d, J = 7.1 Hz, 2H, 2xArH), 7.41 (m_c, 3H, 3xArH), 5.09 (s, 2H, $PhCH_2$), 4.13 (q, J = 7.1 Hz, 2H, OCH_2), 4.05 – 3.86 (m, 6H, 3xCH_2), 2.27 – 2.08 (m, 6H, 3xCH_2), 1.23 (t, J = 7.1 Hz, 3H, CH_3); ^{13}C NMR (75 MHz, $CDCl_3$): δ (ppm) = 173.0 (C=O), 133.3 (2xArCH), 130.5 (ArCH), 129.1 (2xArCH), 127.1 (ArC), 66.3 ($PhCH_2$), 61.5 (OCH_2), 53.5 (3xCH_2), 35.7 (C_q), 26.3 (3xCH_2), 14.0 (CH_3); IR (ATR): $\tilde{\nu}$ [cm^{-1}] = 2968 (m), 2884 (w), 1717 (s), 1456 (m), 1373 9m), 1273 (s), 1067 (m), 1026 (m), 860 (m), 841 (m), 762 (m), 700 (s).

The ester **4’** (457 mg, 1.3 mmol, 1.0 equiv) was dissolved in MeOH (2.0 mL) and tetra-butylammoniumhydroxide (TBAH•30H_2O, 1.0 g, 1.3 mmol, 1.0 equiv) was added. The resulting homogenous solution was stirred at rt for 1h. The solvent was removed on a rotary evaporator under reduced pressure. Toluene was added and the solvent was removed again on a rotary evaporator. The semi-solid residue was then triturated with DCM with vigorous stirring. The solid was collected by suction filtration and washed with DCM to give **5’** as an off white solid (298 mg, 1.21 mmol, 94%); mp > 280 °C; ^{1}H NMR (300 MHz, D_2O): δ (ppm) = 7.86 – 7.26 (m, 5H, ArH), 4.38 (s, 2H, $PhCH_2$), 3.48 (m, 6H, 3xCH_2), 2.29 – 1.90 (m, 6H, 3xCH_2); ^{13}C NMR (75 MHz, D_2O): δ (ppm) = 181.7 (C=O), 132.8 (2xArCH), 130.6 (ArCH), 129.1 (2xArCH), 126.8 (ArC), 67.5 ($PhCH_2$), 54.4 (3xCH_2), 36.6 (C_q), 26.7 (3xCH_2); IR (ATR): $\tilde{\nu}$ [cm^{-1}] = 3674 (w), 2986 (s), 2893 (m), 1576 (w), 1361 (m), 1250 (w), 1074 (s), 1057 (s), 860 (w), 774 (m), 714 (m); elemental analysis calcd (%) for $C_{15}H_{19}NO_2$•H_2O: C, 68.42; H, 8.04; N, 5.32; found: C, 68.22; H, 7.60; N, 5.20.

4.2 Conversion of the zwitterion 5' to the acyl azide 9

The zwitterion **5'** (196 mg, 0.8 mmol, 1.0 equiv) was refluxed in $SOCl_2$ (0.5 mL) for 2h under argon. The excess of $SOCl_2$ was distilled off *in vacuo* and collected in a trap cooled with liquid nitrogen. The residue was further dried in high vacuum to give the acid chloride **6'** as a colorless solid, which was used in the next step without further purification.

The acid chloride **6'** obtained above was suspended in acetone (4.0 mL), and a solution of NaN_3 (57 mg, 0.8 mmol, 1.0 equiv) in H_2O (1.0 mL) was slowly added at 0 °C. The reaction mixture was stirred at 0 °C for 30 min, the acetone was then evaporated under reduced pressure. The residue was saturated with solid NaCl and extracted with DCM (3 x 5 mL). The combined organic extracts were dried over Na_2SO_4, filtered and evaporated under reduced pressure. Et_2O was added and the solvent was evporated on a rotary evaporator, for the azeotropic removal of DCM. The remaining solid was triturated with Et_2O, and the solvent was decanted. After drying under high vacuum, the product **9** was obtained as a colorless amorphous solid (70 mg, 0.23 mmol, 29%) which was highly hygroscopic, liquification occurred upon contact with air; ^{1}H NMR (500 MHz, $CDCl_3$): δ (ppm) = 7.65 (d, *J* = 6.9 Hz, 2H, 2xArH), 7.37 (m_c, 3H, 3xArH), 5.01 (s, 2H, $PhCH_2$), 3.96 – 3.78 (m, 6H, $3xCH_2$), 2.22 – 2.03 (m, 6H, $3xCH_2$); ^{13}C NMR (75 MHz, $CDCl_3$): δ (ppm) = 180.9 (C=O), 133.4 (2xArCH), 130.5 (ArCH), 129.2 (2xArCH), 127.2 (ArC), 66.6 ($PhCH_2$), 53.4 ($3xCH_2$), 37.7 (C_q), 26.3 ($3xCH_2$); IR (ATR): $\tilde{\nu}$ [cm^{-1}] = 3368 (br), 2972 (w), 2886 (w), 2143 (m), 1697 (m), 1458 (m), 1207 (s), 1177 (s), 928 (m), 862 (m), 768 (m), 706 (s).

5. Synthesis of the N-benzyl-4-quinuclidinium diazomethyl ketone 10

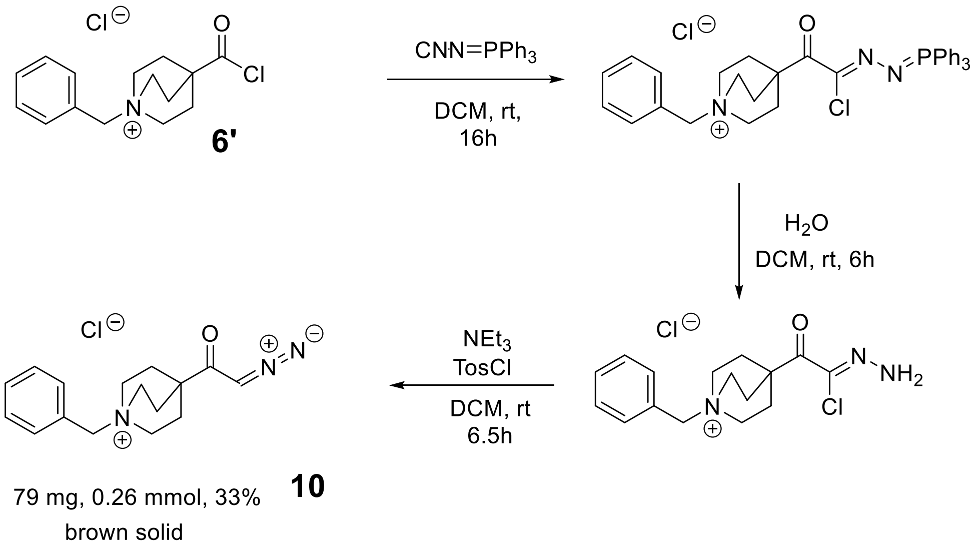

The acid chloride **6’** (0.8 mmol, 1.0 eq.) was prepared as described before for the synthesis of the acyl azide **9**. For its conversion to the diazomethyl ketone **10**, dried DCM (9.5 mL) and (*N*-isocyanoimino) triphenyl-phosphorane (531 mg, 1.6 mmol, 2.0 eq., 90%) were added to the *Schlenk tube* containing the acid chloride **6’**, similar to the procedure described by Aller *et al*.[2] After stirring at rt for 16h, the mixture had turned dark. Water (476 µL) was added, and the resulting mixture was stirred at rt for 6h. The solvent was distilled off under reduced pressure, and collected in a trap cooled with liquid nitrogen. The residue was dried under vacuum overnight, yielding a red solid. The crude hydrazone product was dissolved in dried DCM (9.5 mL) and Et_3N (0.11 mL, 0.8 mmol, 1.0 eq.), then toluenesulfonyl chloride (30 mg, 0.16 mmol, 0.2 eq.) was added. The reaction mixture was stirred at rt for 6.5h. Volatiles were removed under reduced pressure, and the crude product was purified by column chromatography on silica gel (MeOH/DCM = 1/5), affording the diazomethyl ketone **10** as a brown solid (79 mg, 0.26 mmol, 33 %).

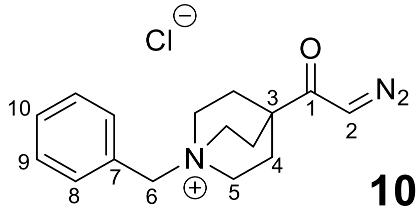

10

^1H NMR (300 MHz, $CDCl_3$): δ (ppm) = 7.60-7.58 (H-9, m, 2H), 7.49-7.42 (H-8, H-10, m, 3H), 5.86 (H-2, s, 1H), 4.93 (H-6, s, 2H), 3.89-3.84 (H-5, m, 6H), 2.21-2.16 (H-4, m, 6H); ^{13}C NMR (126 MHz, $CDCl_3$): δ (ppm) = 194.8 (C-1), 133.4 (C-9), 130.8 (C-10), 129.4 (C-8), 127.1 (C-7), 67.1 (C-3), 54.5 (C-2), 54.1 (C-5), 39.5 (C-6), 26.5 (C-4).

[1] O. Dumele, B. Schreib, U. Warzok, N. Trapp, C. A. Schalley, F. Diederich, *Angew. Chem. Int. Ed.* **2017**, *56*, 1152-1157.

[2] E. Aller, P. Molina, Á. Lorenzo, *Synlett* **2000**, *4*, 526–528.

6. NMR spectra

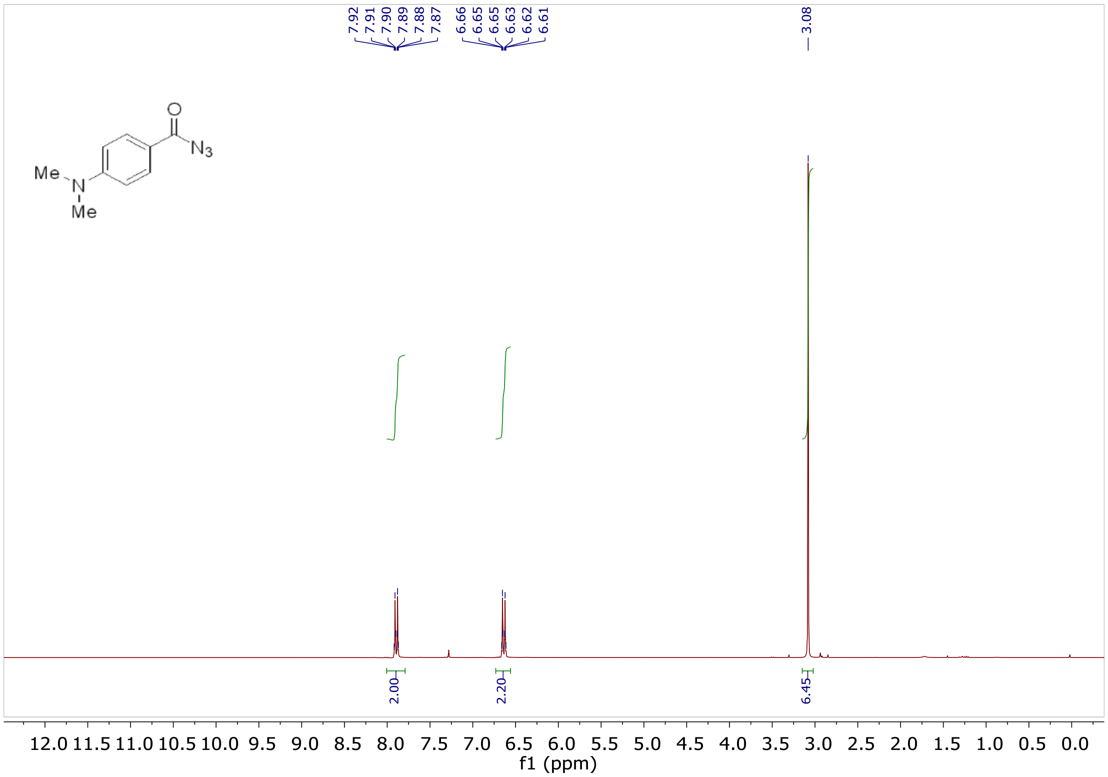

Figure S1. ^{1}H NMR (300 MHz, $CDCl_3$) of **2**.

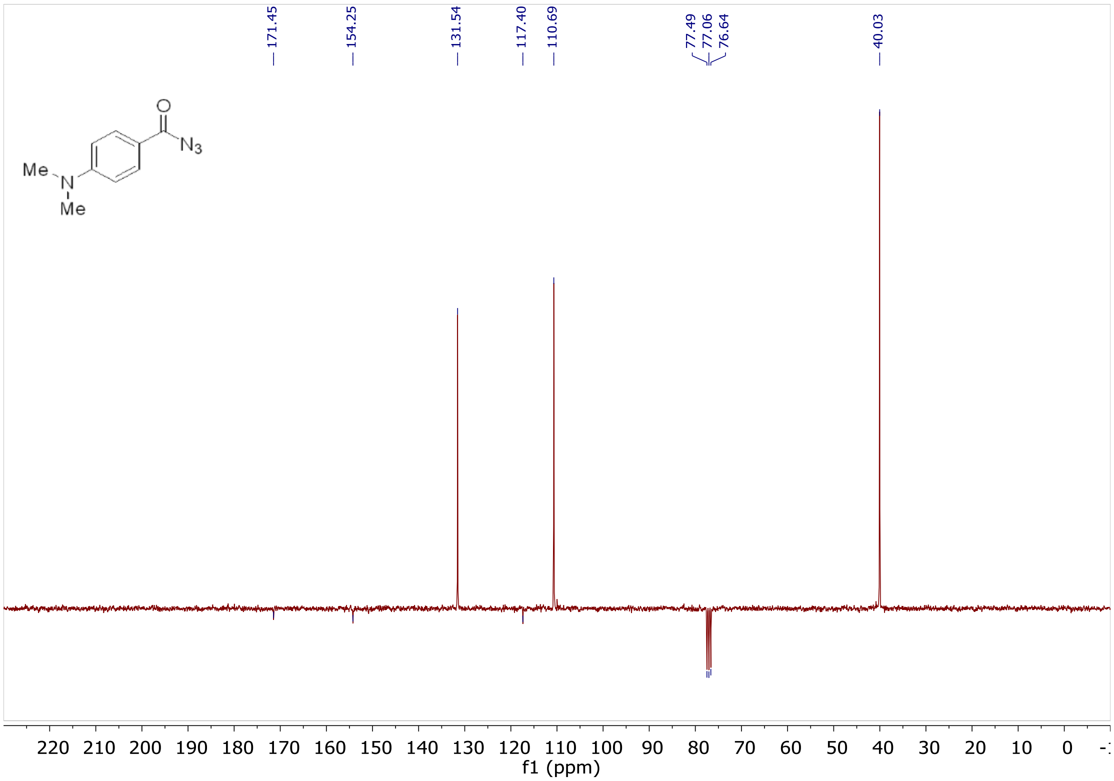

Figure S2. ^{13}C NMR (75 MHz, APT, $CDCl_3$) of **2.**

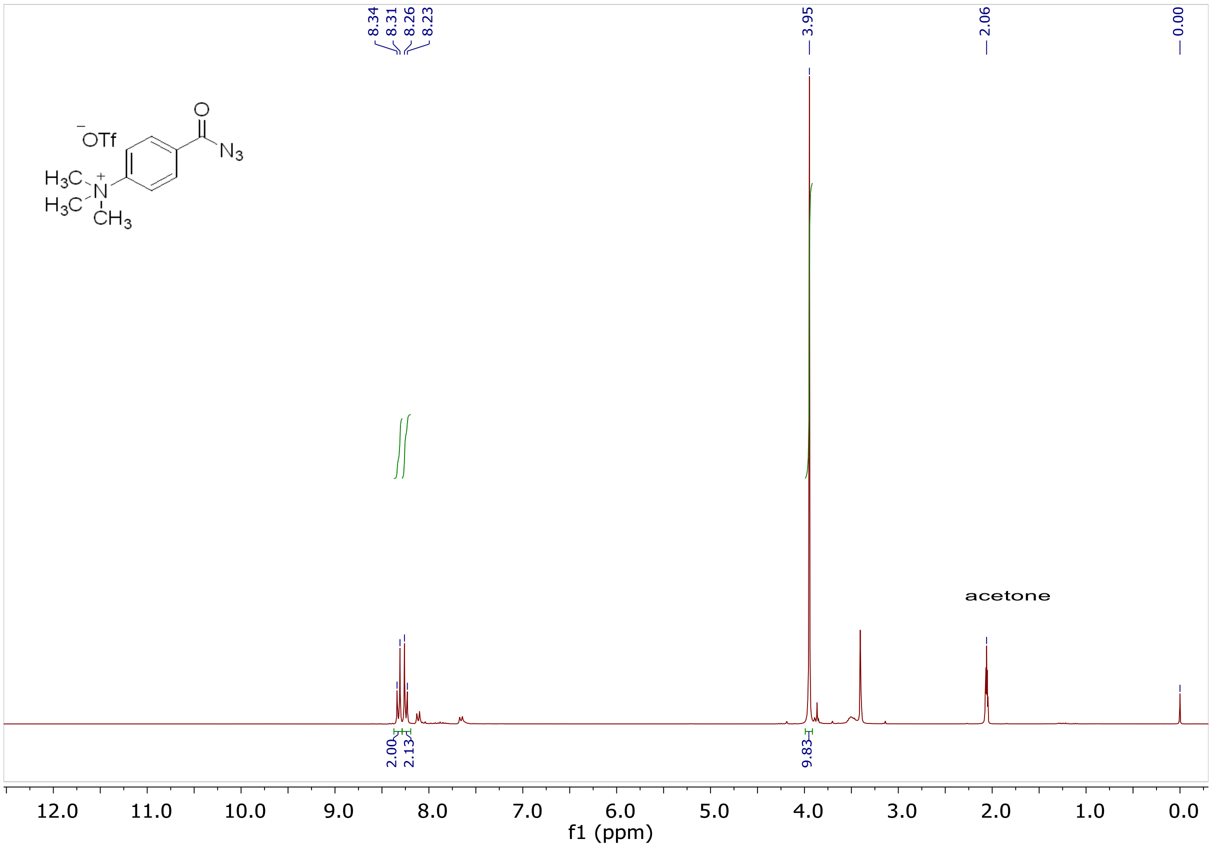

Figure S3. ^{1}H NMR (300 MHz, acetone-d_6) of **7**.

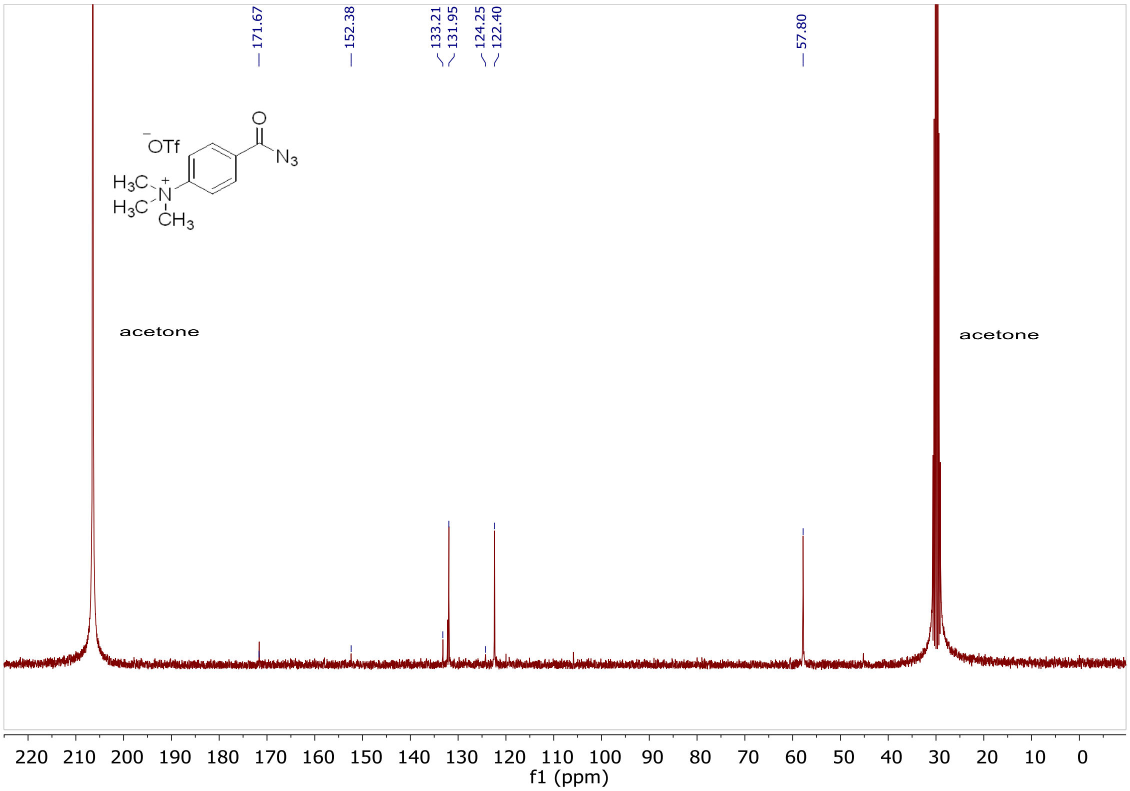

Figure S4. ^{13}C NMR (75 MHz, acetone-d_6) of **7**.

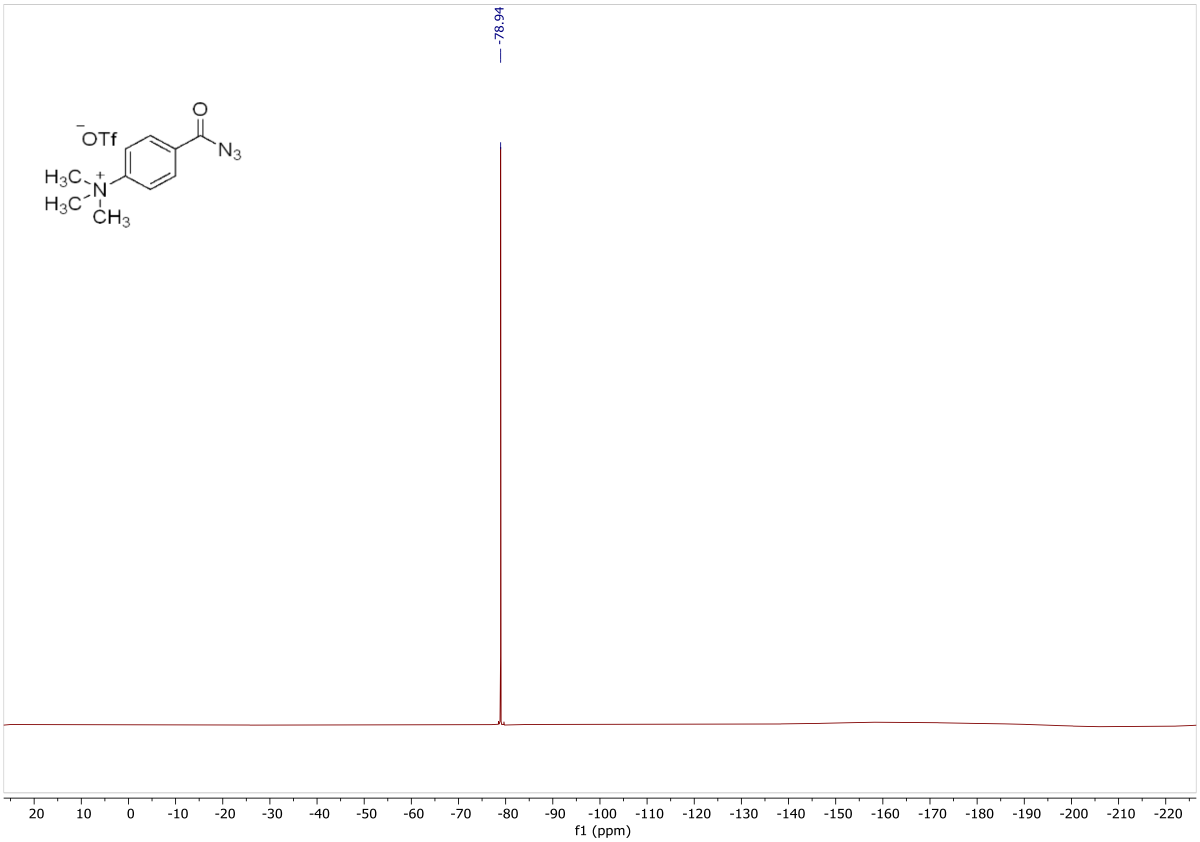

Figure S5. ^{19}F NMR (282 MHz, acetone-d_6) of **7**

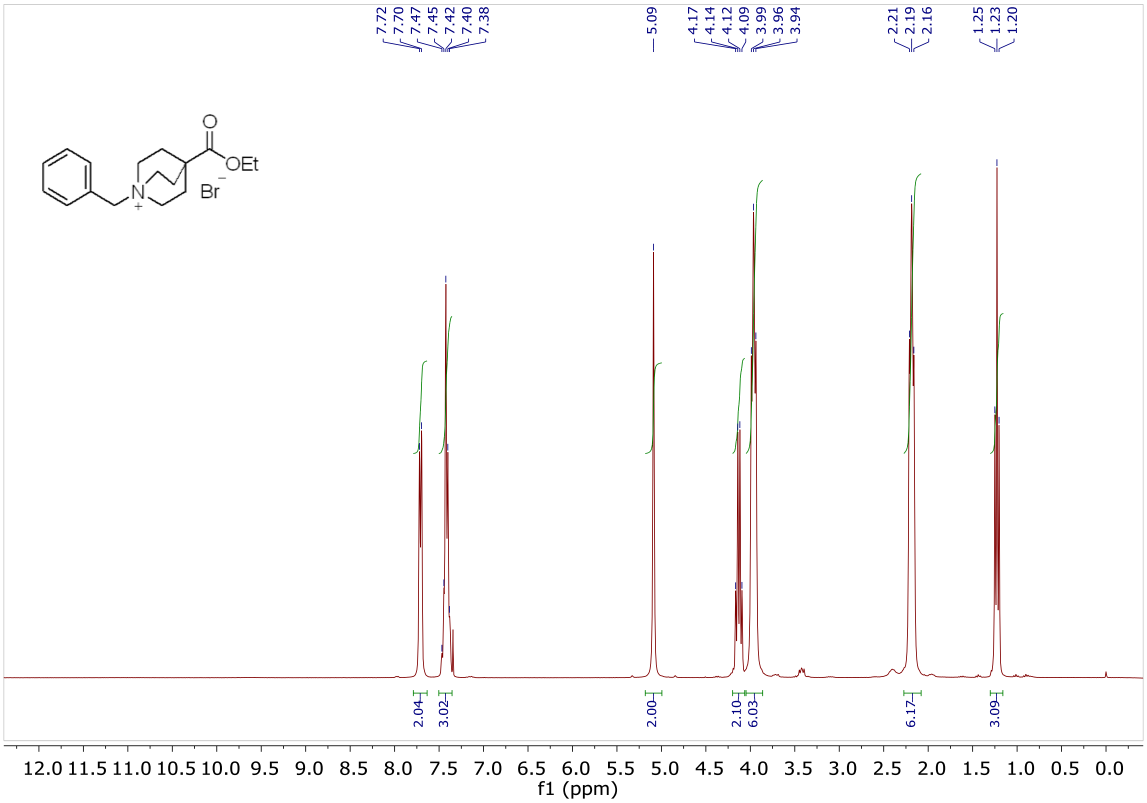

Figure S6. ^{1}H NMR (300 MHz, $CDCl_3$) of **4'**.

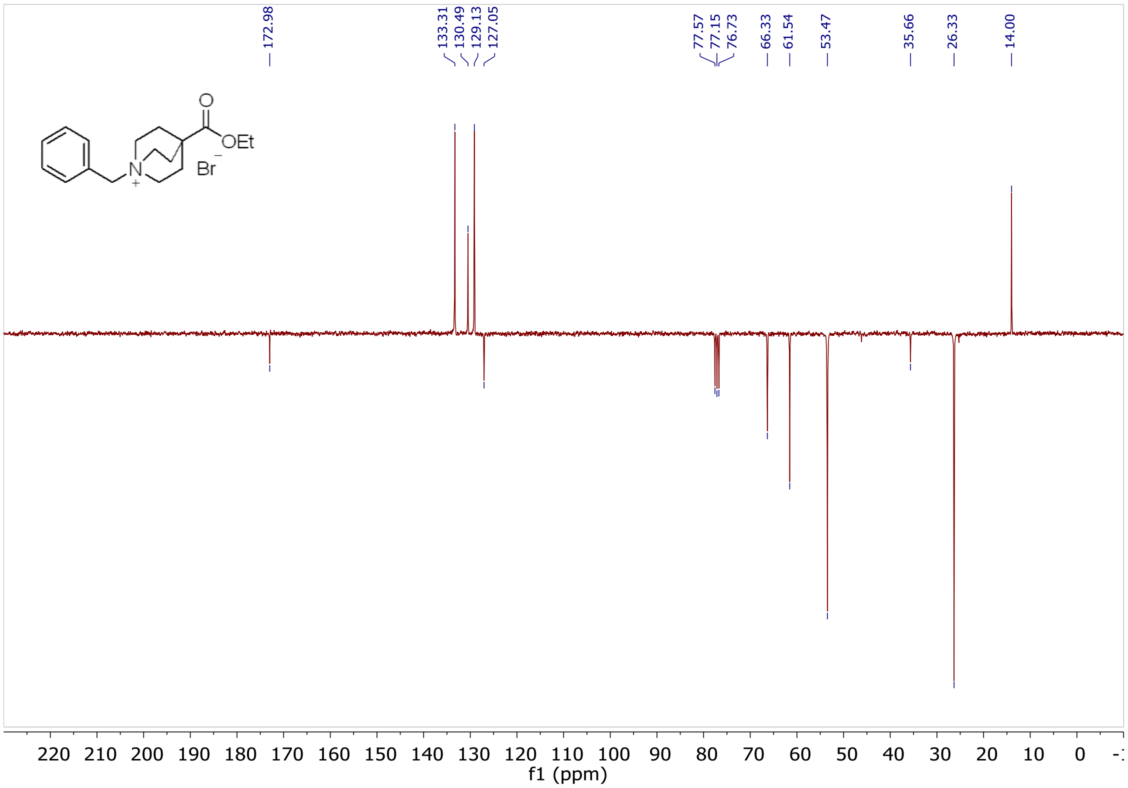

Figure S7. ^{13}C NMR (75 MHz, APT, $CDCl_3$) of **4'**.

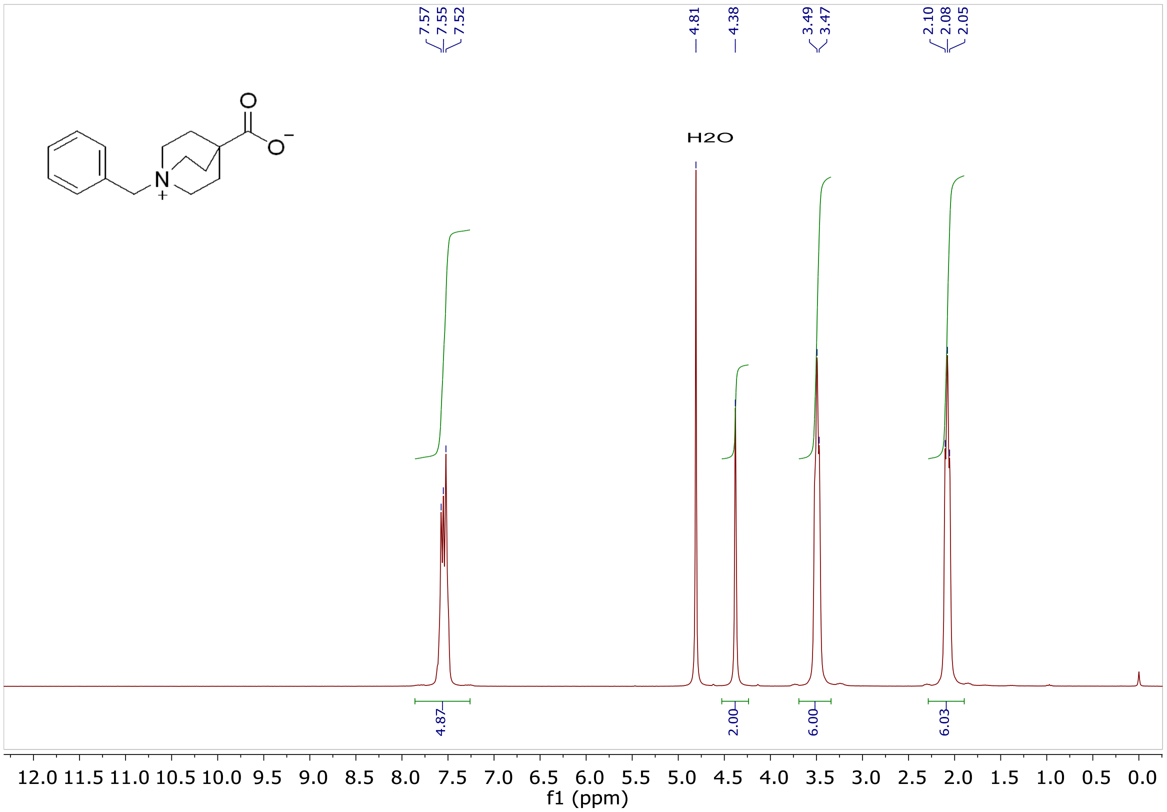

Figure S8. ^{1}H NMR (300 MHz, D_2O) of **5'**.

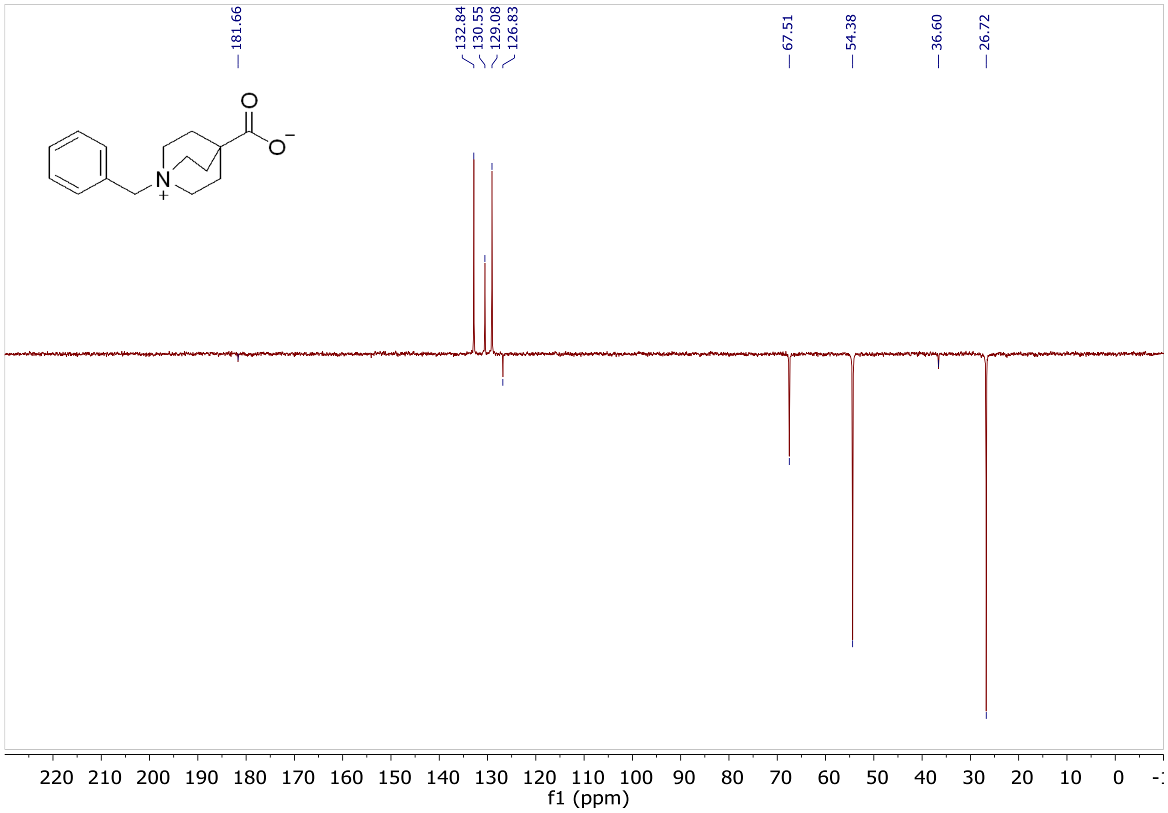

Figure S9. ^{13}C NMR (75 MHz, APT, D_2O) of **5'**.

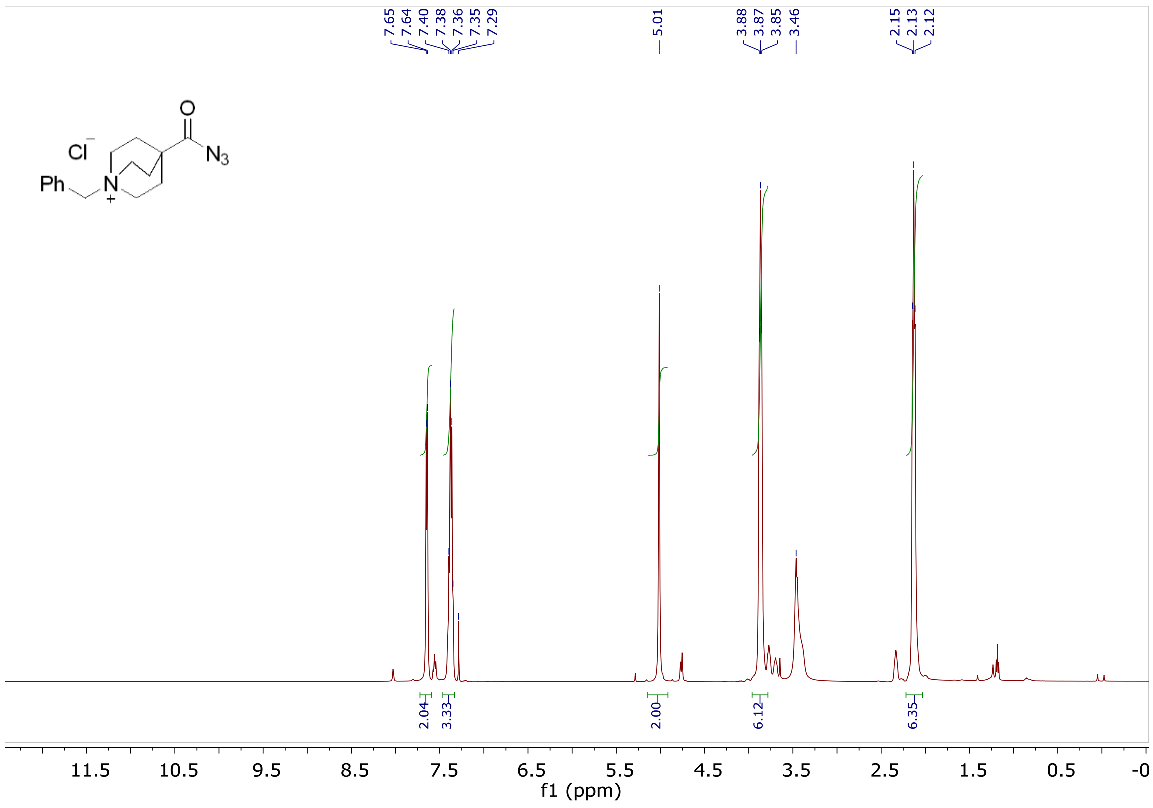

Figure S10. ^{1}H NMR (500 MHz, $CDCl_3$) of **9**.

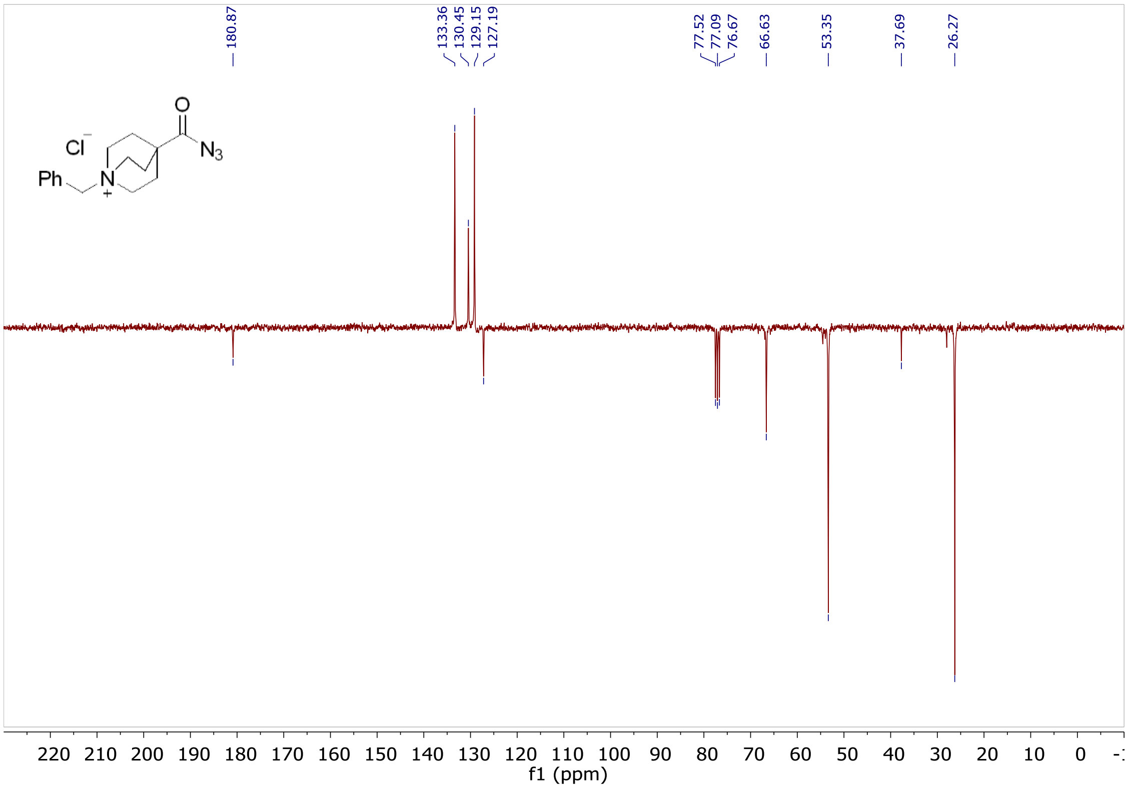

Figure S11. ^{13}C NMR (75 MHz, APT, $CDCl_3$) of **9**.

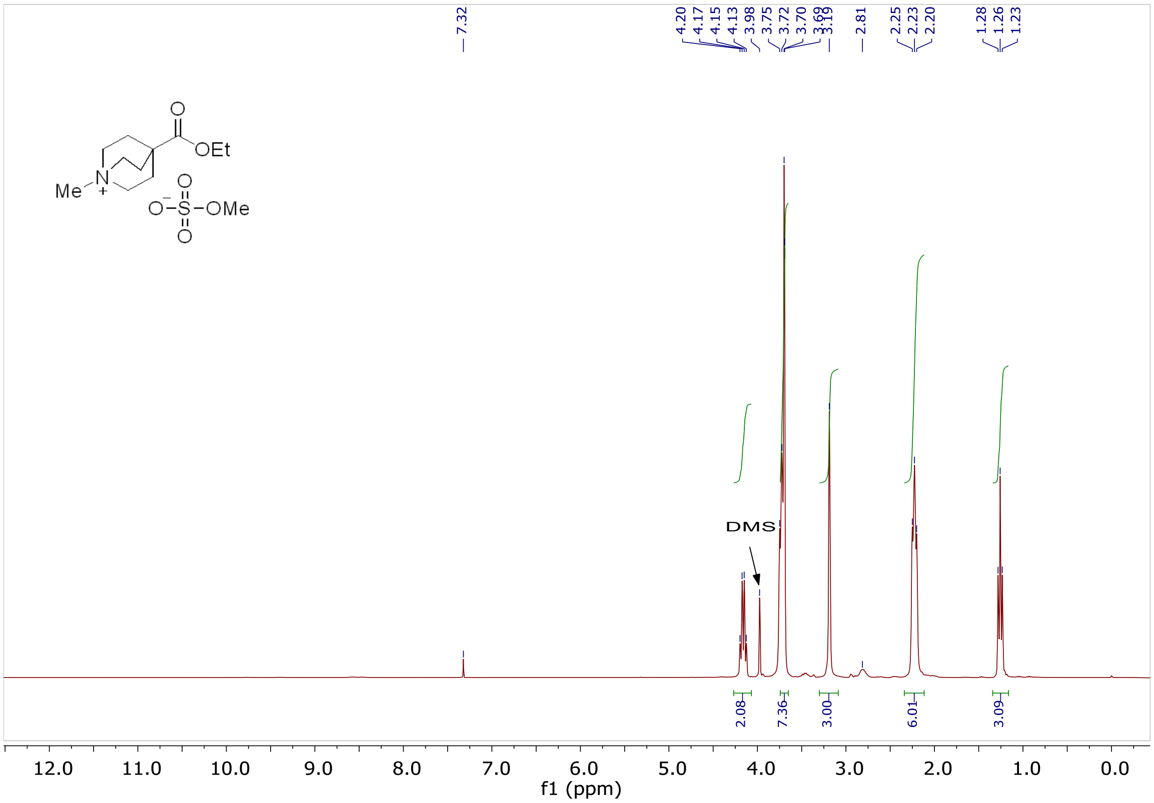

Figure S12. ^{1}H NMR (300 MHz, $CDCl_3$) of **4**.

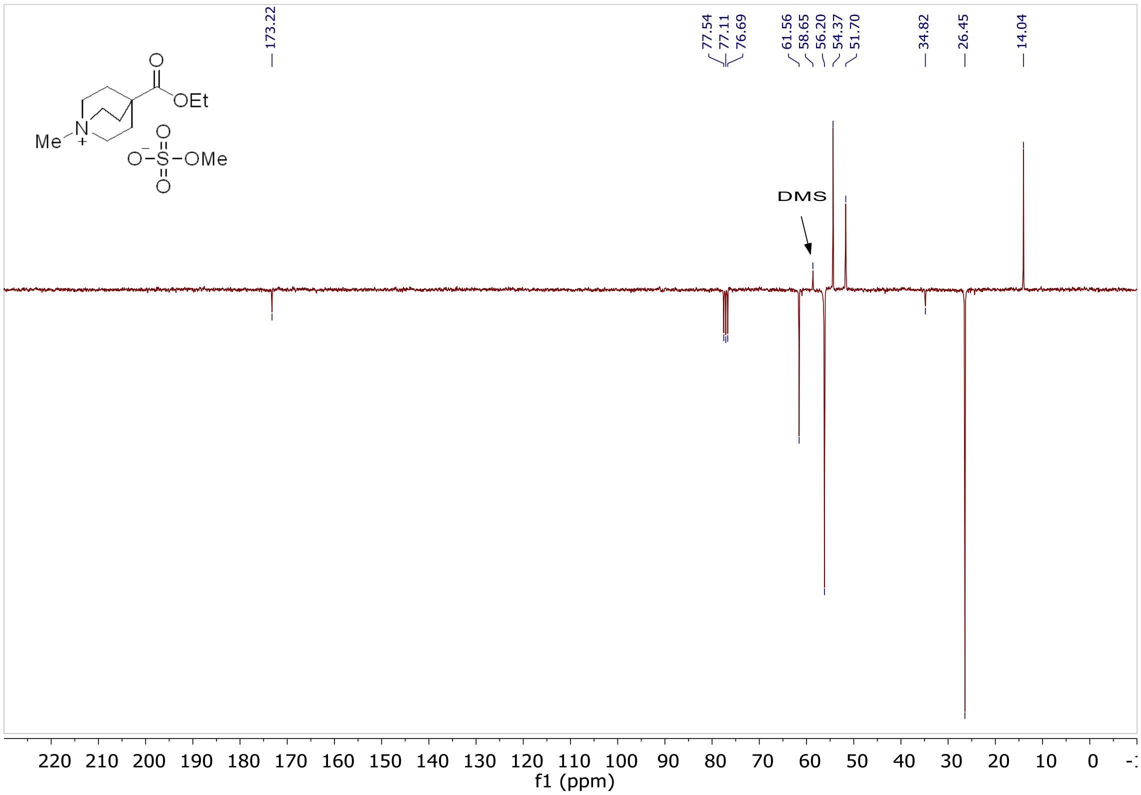

Figure S13. ^{13}C NMR (75 MHz, APT, $CDCl_3$) of **4**.

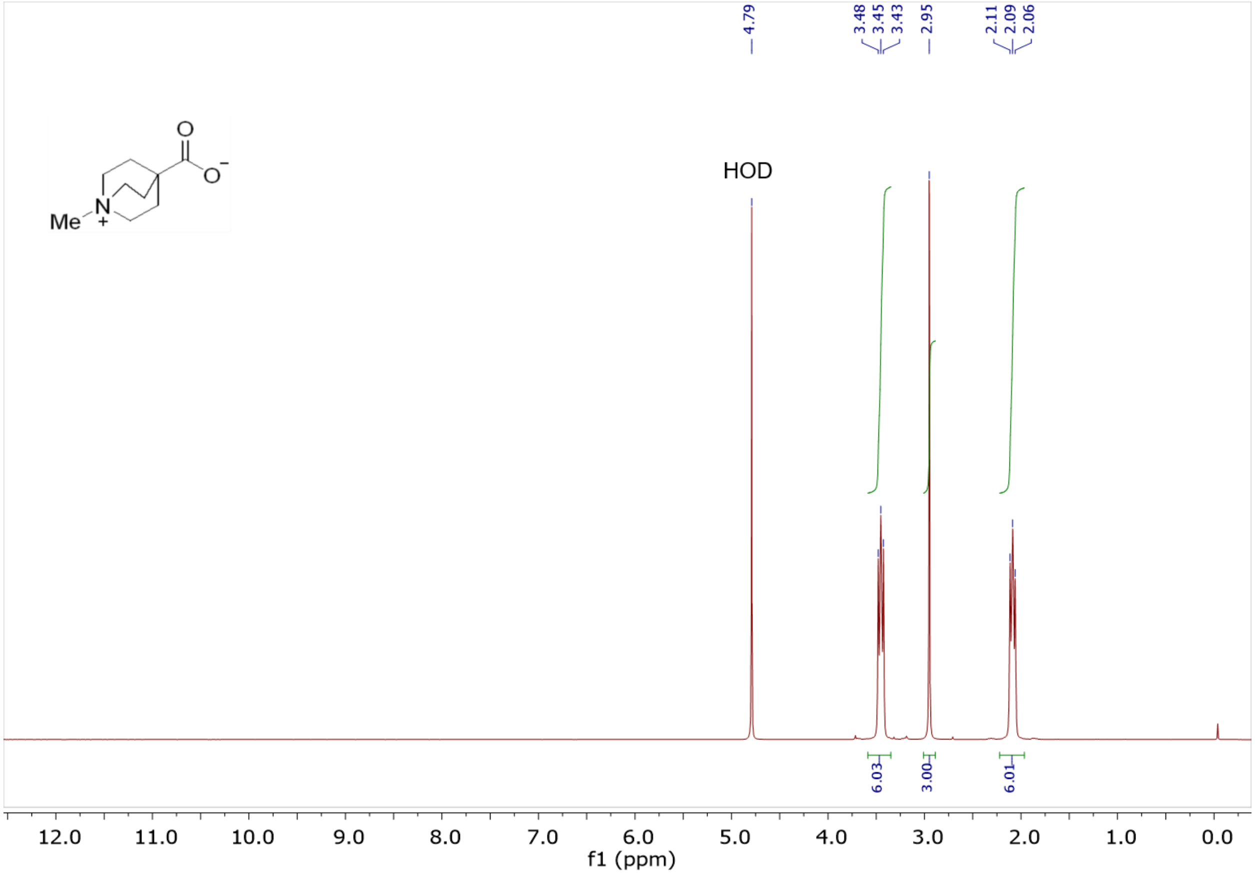

Figure S14. ^{1}H NMR (300 MHz, D_2O) of **5**.

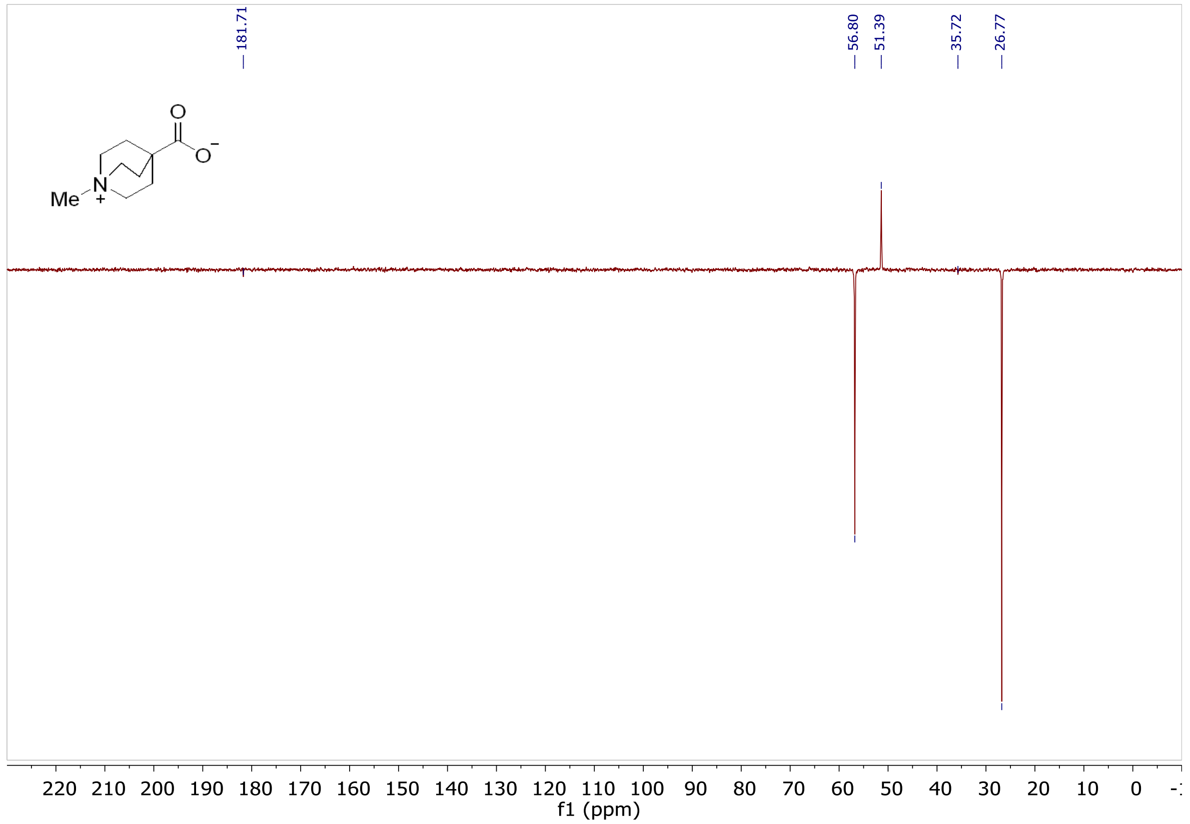

Figure S15. ^{13}C NMR (75 MHz, APT, D_2O) of **5**.

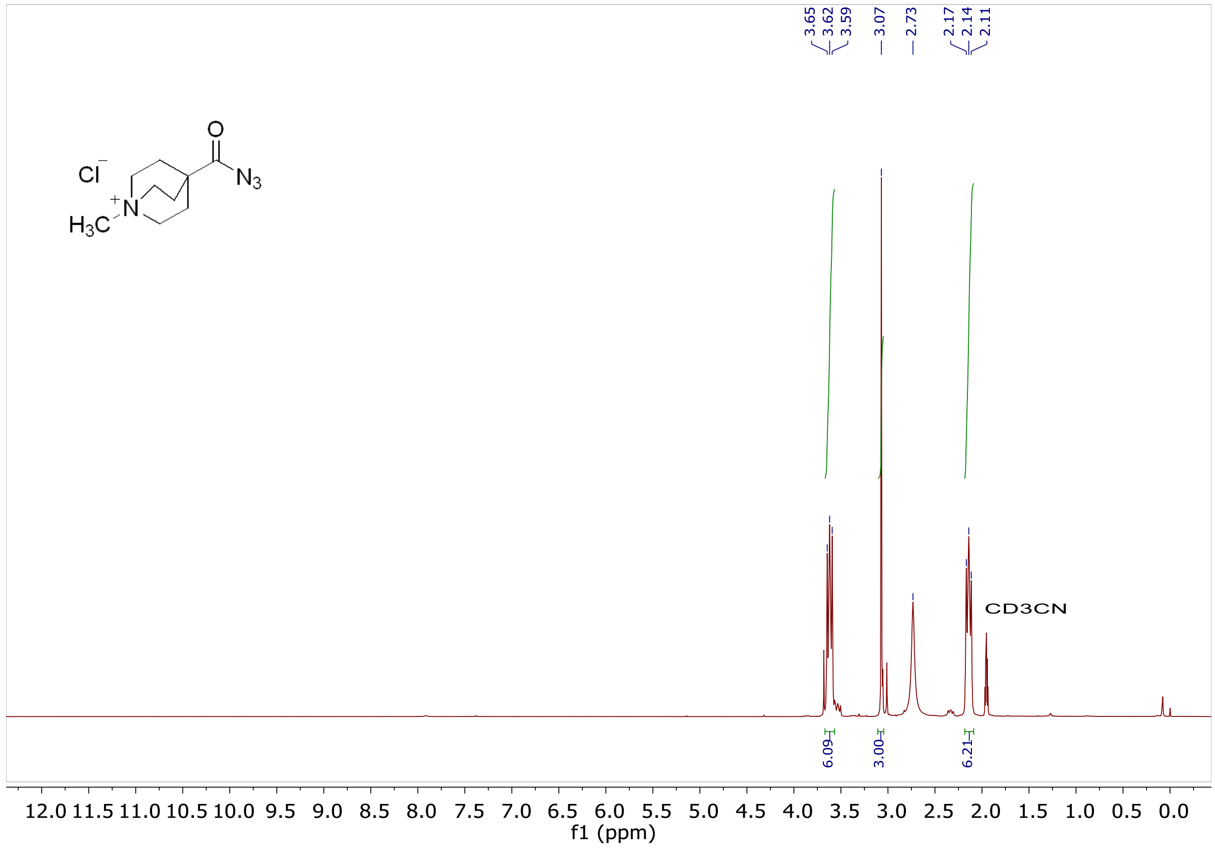

Figure S16. ^{1}H NMR (300 MHz, CD_3CN) of **8**.

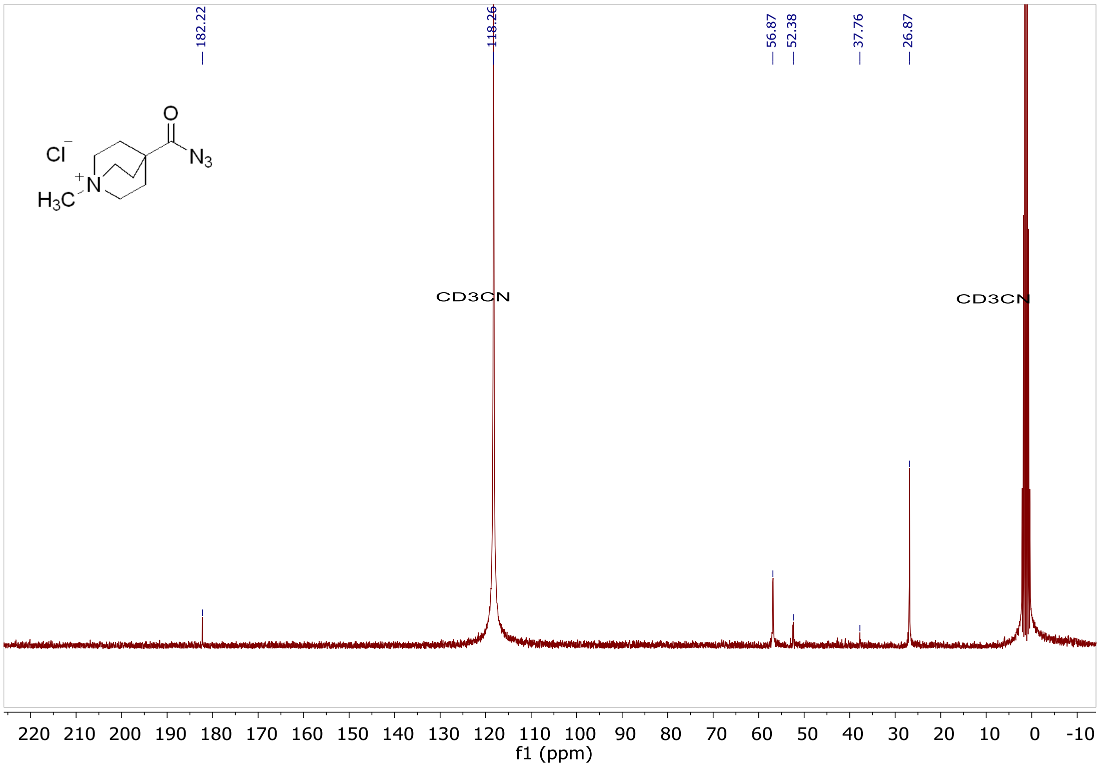

Figure S17. ^{13}C NMR (75 MHz, CD_3CN) of **8**.

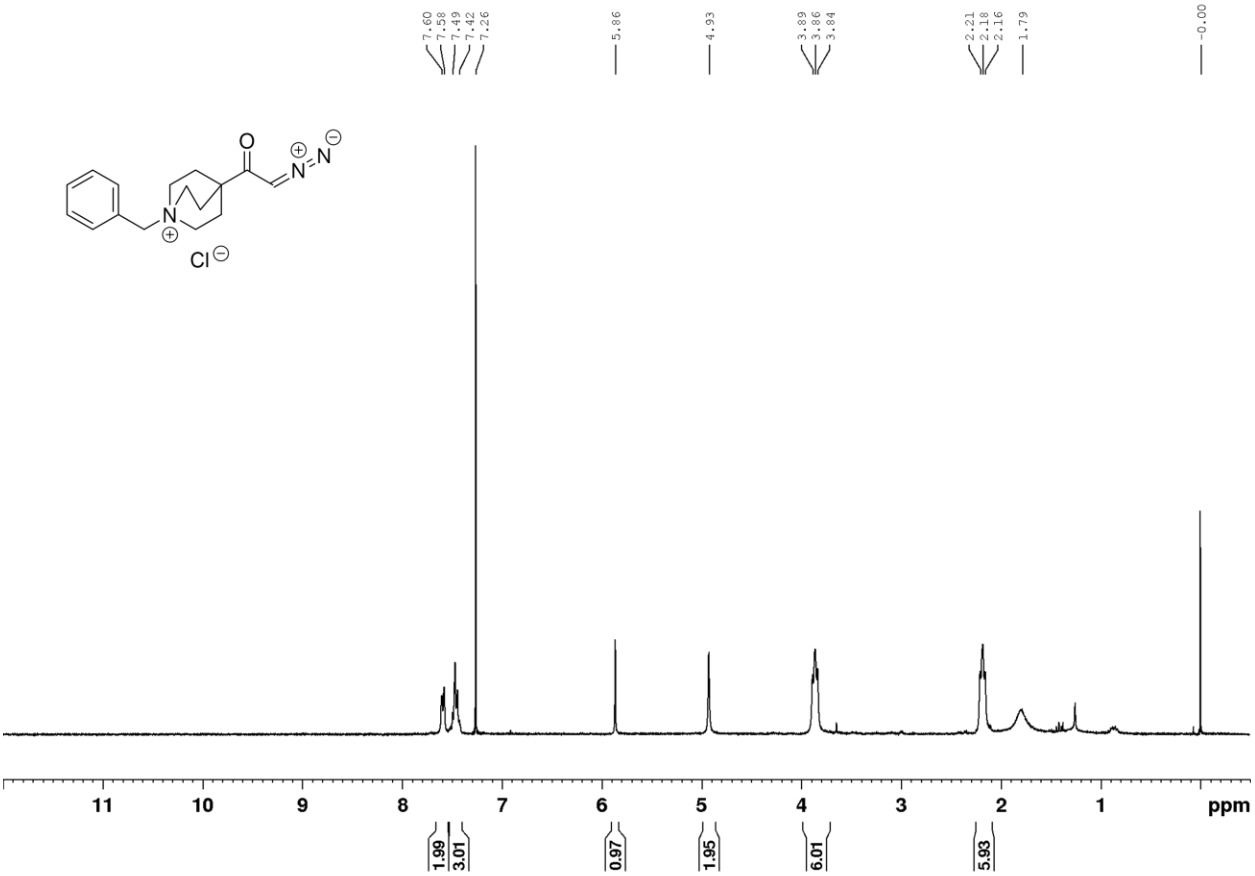

Figure S18. ^{1}H NMR (300 MHz, $CDCl_3$) of **10**.

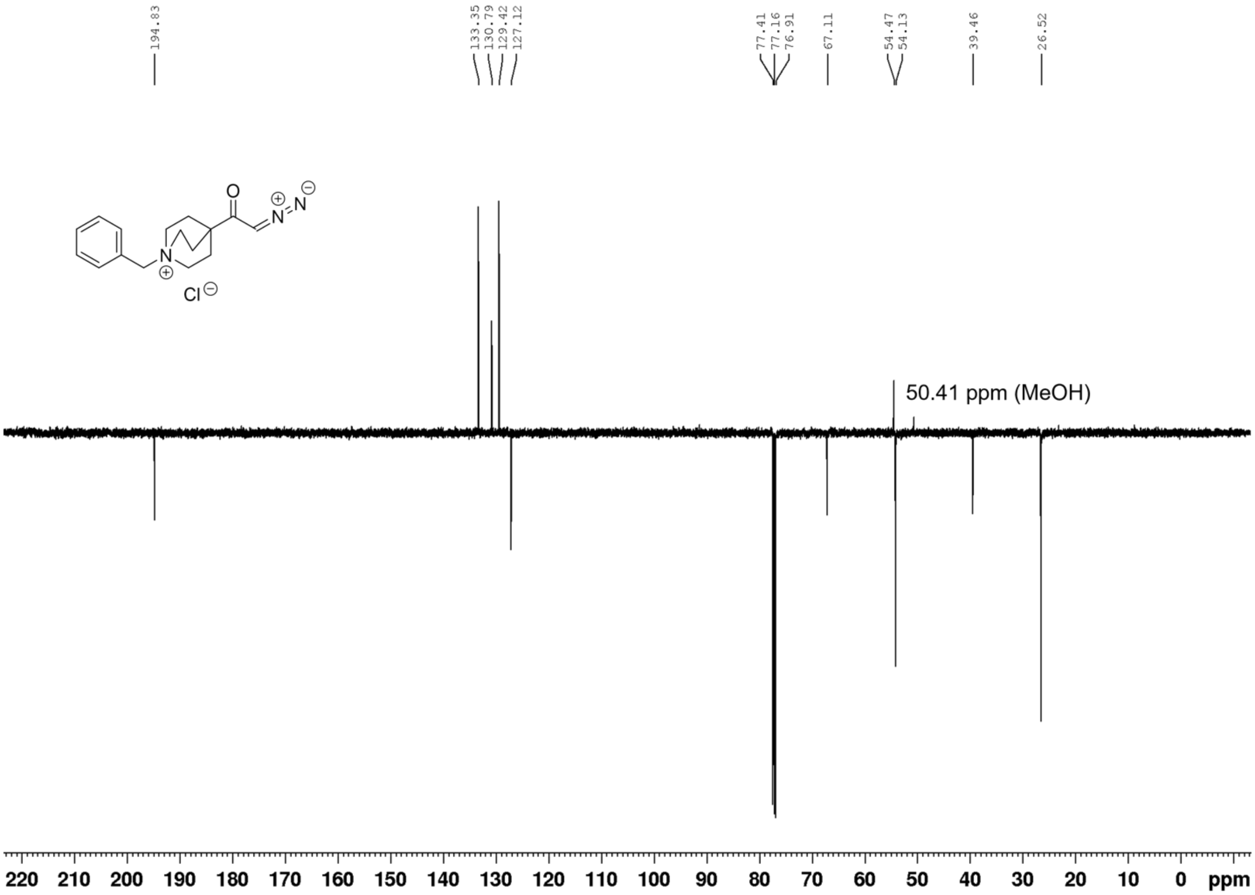

Figure S19. ^{13}C NMR (126 MHz, $CDCl_3$) of **10**.

7. IR spectra (ATR)

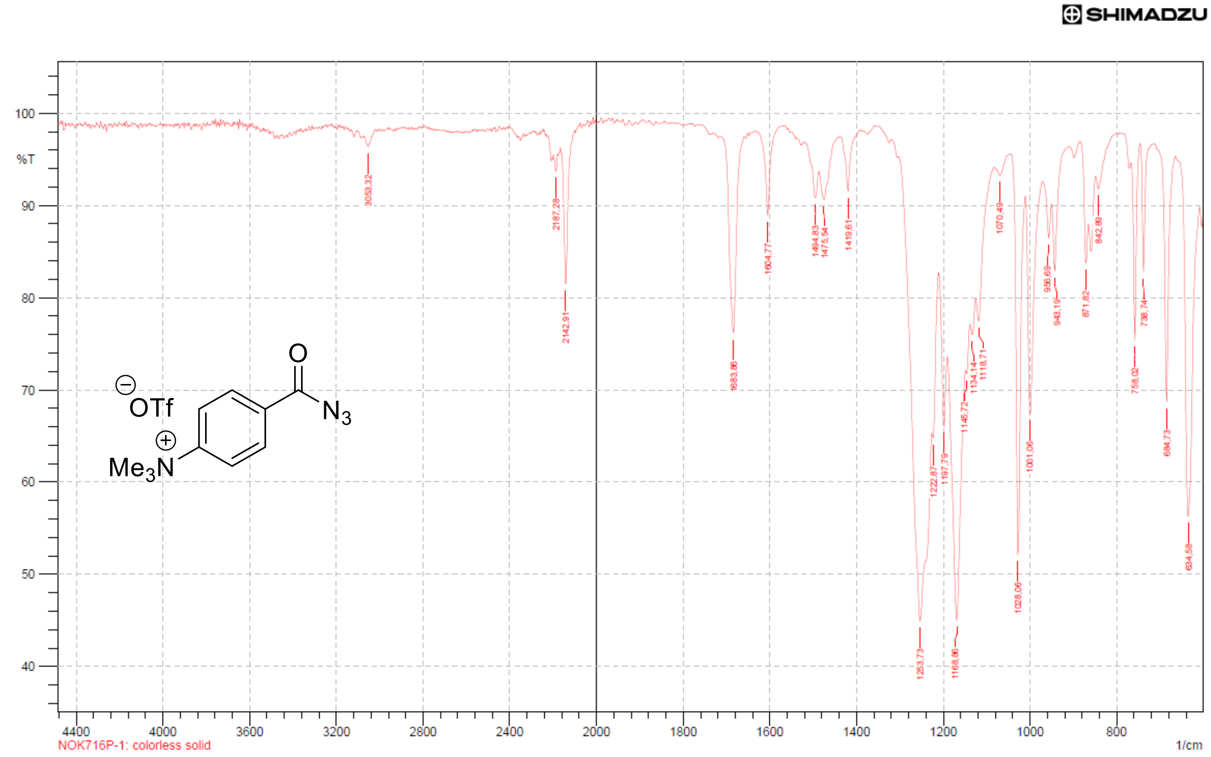

Figure S20. IR spectrum of **7.**

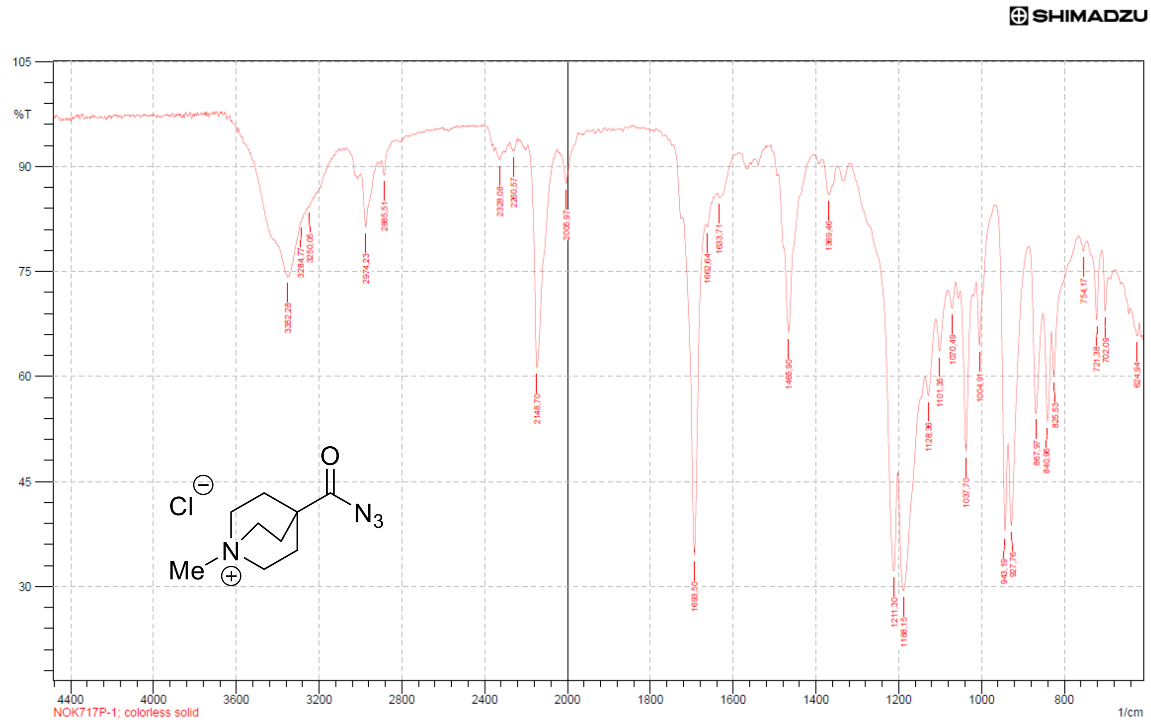

Figure S21. IR spectrum of **8.**

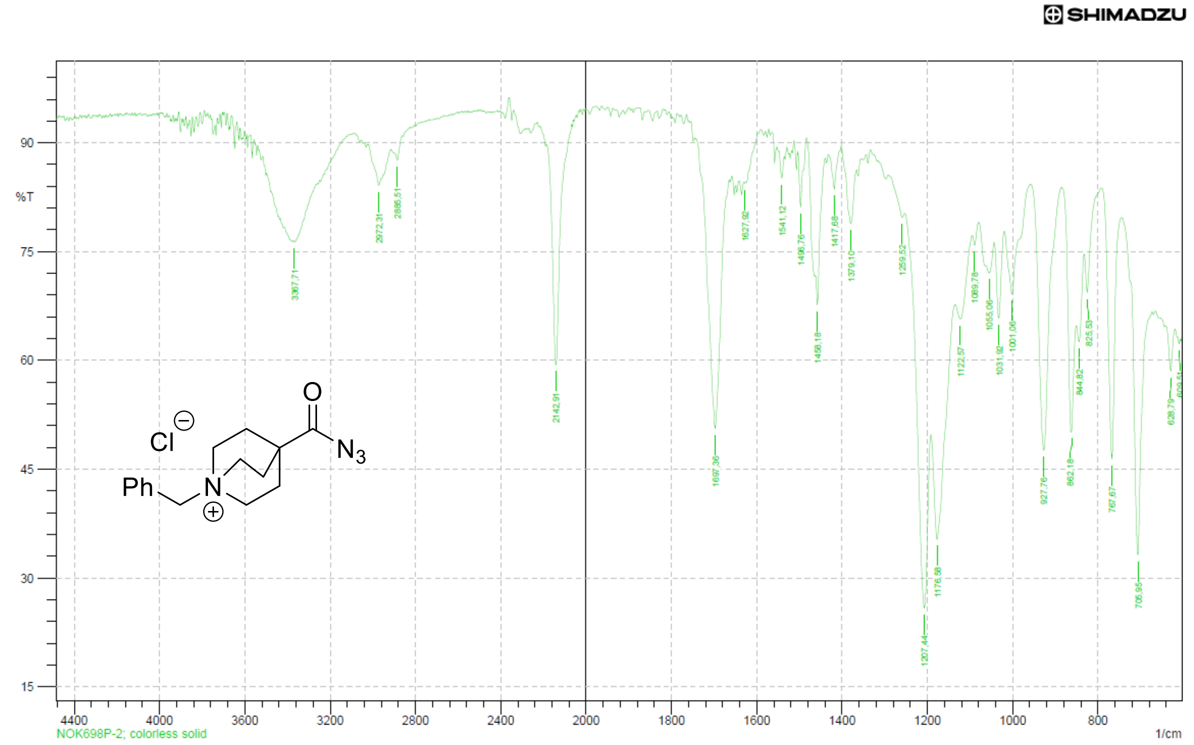

Figure S22. IR spectrum of **9.**

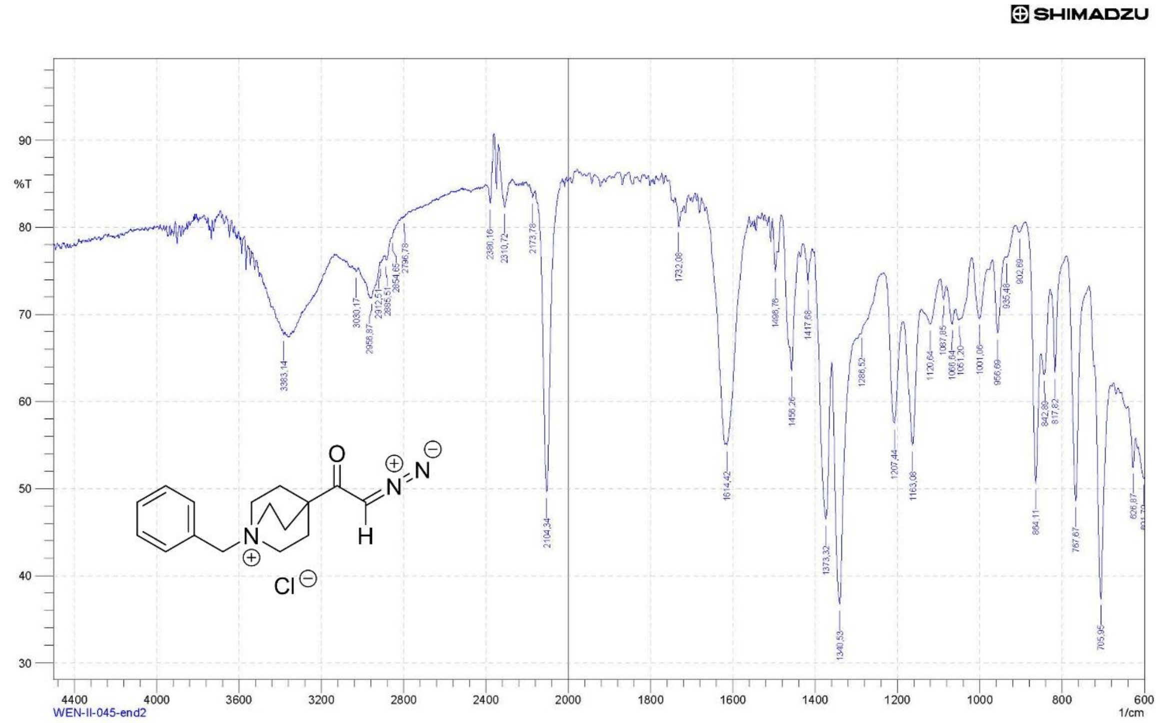

Figure S23. IR spectrum of **10.**

Supporting Information

to

***Gas-phase Curtius and Wolff* rearrangement reactions investigated by tandem-MS, IR ion spectroscopy and theory**

by

Wacharee Harnying, Hui-Chung Wen, Jonathan Martens, Giel Berden, Jos Oomens, Jana Roithová, Albrecht Berkessel, Mathias Schäfer and Anthony J.H.M. Meijer

Part II
Mass Spectrometry

Table S1. (+)ESI-MS Accurate ion mass measurements of the precursor ions analyzed with IRIS.

Sample	Nominal Ion Mass [m/z]	Theo. mass [u]	Accurate Ion Mass measured [u]	Composition	Error (ppm)
**7** 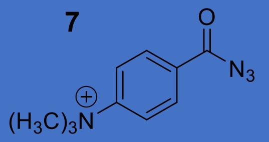	205	205.1084	205.1082	$[C_{10}H_{13}ON_4]^+$	-0.95
$[7\text{-}N_2]^+$	177	177.1022	177.1021	$[C_{10}H_{13}ON_2]^+$	-0.6
**8** 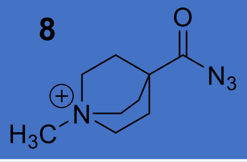	195	195.1240	195.1224	$[C_9H_{15}ON_4]^+$	-0.2
$[8\text{-}N_2]^+$	167	167.1179	167.1180	$[C_9H_{15}ON_2]^+$	0.5
$[8\text{-}N_2\text{-}C_2H_4]^+$	139	139.0866	139.0876	$[C_7H_{11}ON_2]^+$	1.0
**9** 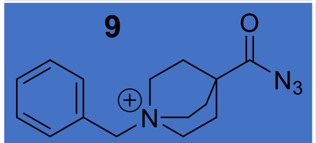	271	271.1553	271.1556	$[C_{15}H_{19}ON_4]^+$	1.3
$[9\text{-}N_2]^+$	243	243.1492	243.1502	$[C_{15}H_{19}ON_2]^+$	1.0
$[9\text{-}N_2–C_2H_4]^+$	139	139.0866	139.0875	$[C_7H_{11}ON_2]^+$	6.8
**10** 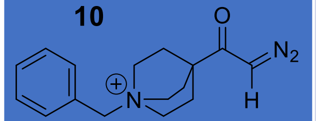	270	270.1601	270.1601	$[C_{16}H_{20}ON_3]^+$	-0.03
$[10\text{-}N_2]^+$	242	242.1539	242.1534	$[C_{16}H_{20}ON_2]^+$	-0.5

Table S2. Tandem-MS and Photofragmentation of the selected precursor ions used for IRIS. All ions are characterized with accurate ion masses that match the respective compositions and confirm the identity of the respective ions.

Sample	Precursor Ion [m/z]	MS^2 CID Product Ions [m/z]	Photofragments at Laser Attenuation	
			0dB [m/z]	3dB [m/z]
**7**	205	[**7**-N_2]$^+$ *m/z* 177<br>[**7**-N_2-$^{\bullet}CH_3$]$^+$ *m/z* 162	177, 163, 162, 161, 146, 145, 135, 134, 133, 119, 118	
**[7-N_2]$^+$**	177	[**7**-N_2-$^{\bullet}CH_3$]$^+$ *m/z* 162	162, 161, 146, 145, 135, 134, 133, 119, 118, 117, 116, 107, 106, 104, 93, 92, 91, 90, 79, 77	162, 161, 146, 135, 134, 133, 119, 118, 117, 116, 107, 106, 91, 90,
**8**	195	[**8**-N_2]$^+$ *m/z* 167	167, 139, 96, 81, 80, 71	
**[8-N_2]$^+$**	167	[**8**-N_2 - C_2H_4]$^+$ *m/z* 139	139, 114, 110, 97, 95, 94, 82, 80, 71, 70, 69, 59, 54, 53 (4 Laser Pulses / data point)	139, 96, 80, 71, 53
	139		114, 97, 96, 95, 94, 84, 83, 71, 70, 69, 55, 54, 53 (4 Laser Pulses / data point)	97, 96, 71, 69, 55, 53
**9**	271	[**9**-N_2]$^+$ *m/z* 243	243, 151, 110, 92, 91	244, 243, 151, 110, 91
**[9-N_2]$^+$**	243	[C_7H_7]$^+$ *m/z* 91	110, 91, 90	
10	270	[**10**-N_2]$^+$ *m/z* 242<br>[C_7H_7]$^+$ *m/z* 91	242, 214, 123, 122, 110, 95, 93, 91	242, 214, 123, 122, 110, 95, 93, 91
**[10-N_2]$^+$**	242	[C_7H_7]$^+$ *m/z* 91		

Figure S24. (+)ESI-MS^2 of the molecular ion of analyte **8** at *m/z* 195. Collision activation performed in a linear quadrupole ion trap with He as inert collision gas and with normalized collision energy, NCE of 12%.

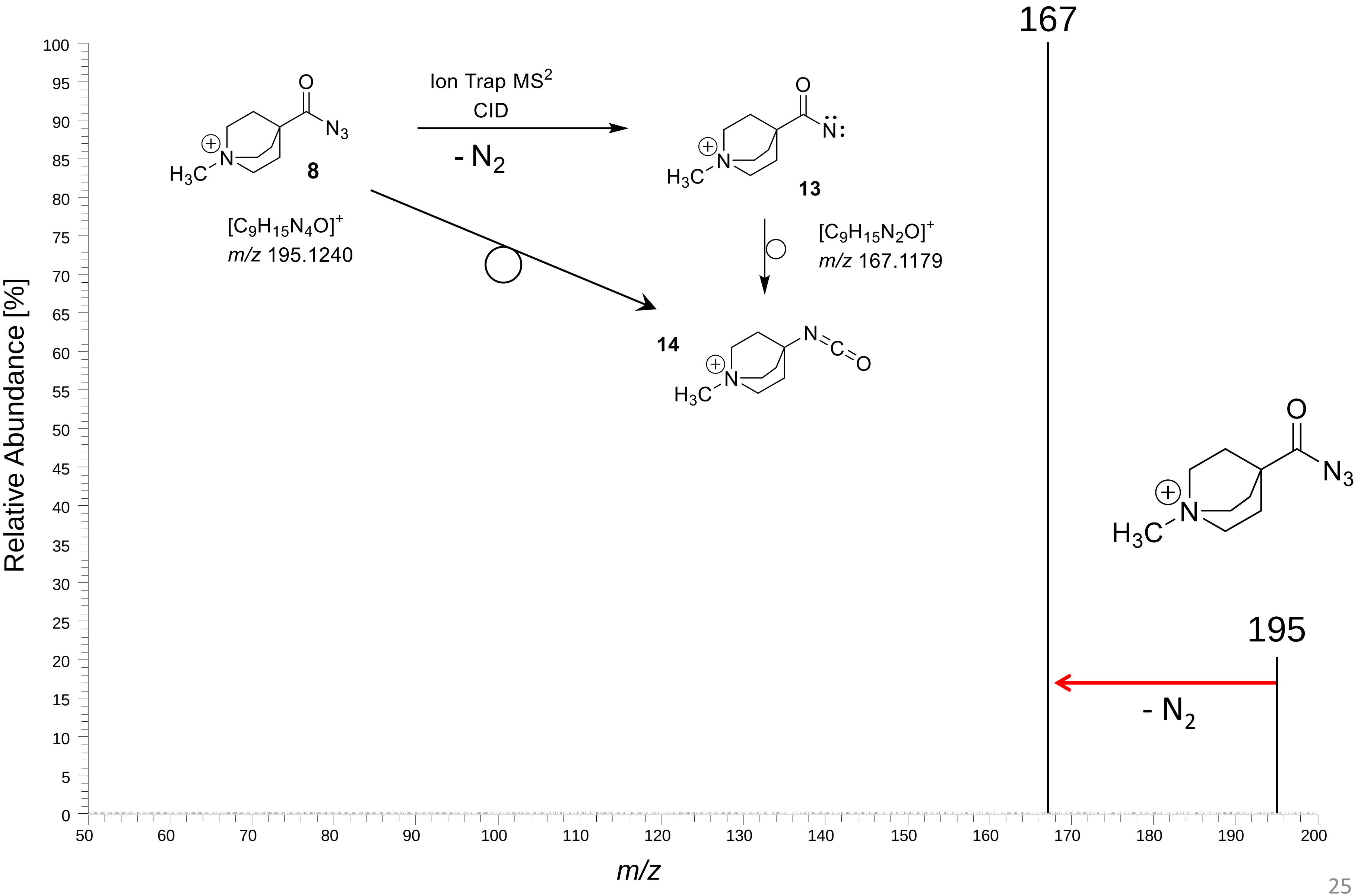

Figure S25. (+)ESI-MS^3 of the product ion at *m/z* 167 activated with NCE 26 formed from the molecular ion of analyte **8** at *m/z* 195. Collision activation performed in a linear quadrupole ion trap with He as inert collision gas and with normalized collision energy NCE of 12%.

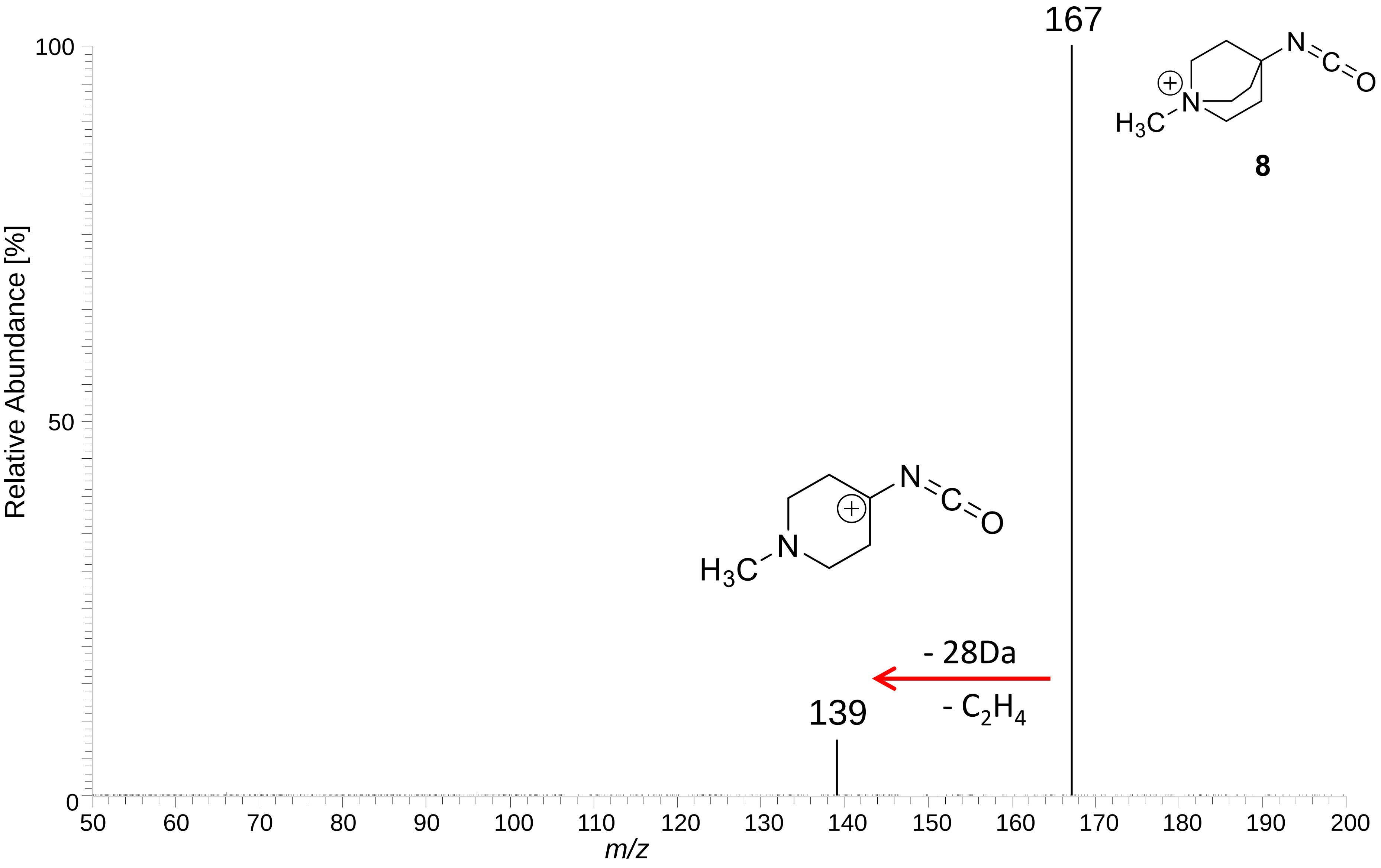

Figure S26. (+)ESI-MS[2] of the molecular ion of analyte **9** at *m/z* 271. Collision activation performed in a linear quadrupole ion trap with He as inert collision gas and with normalized collision energy NCE of 13%.

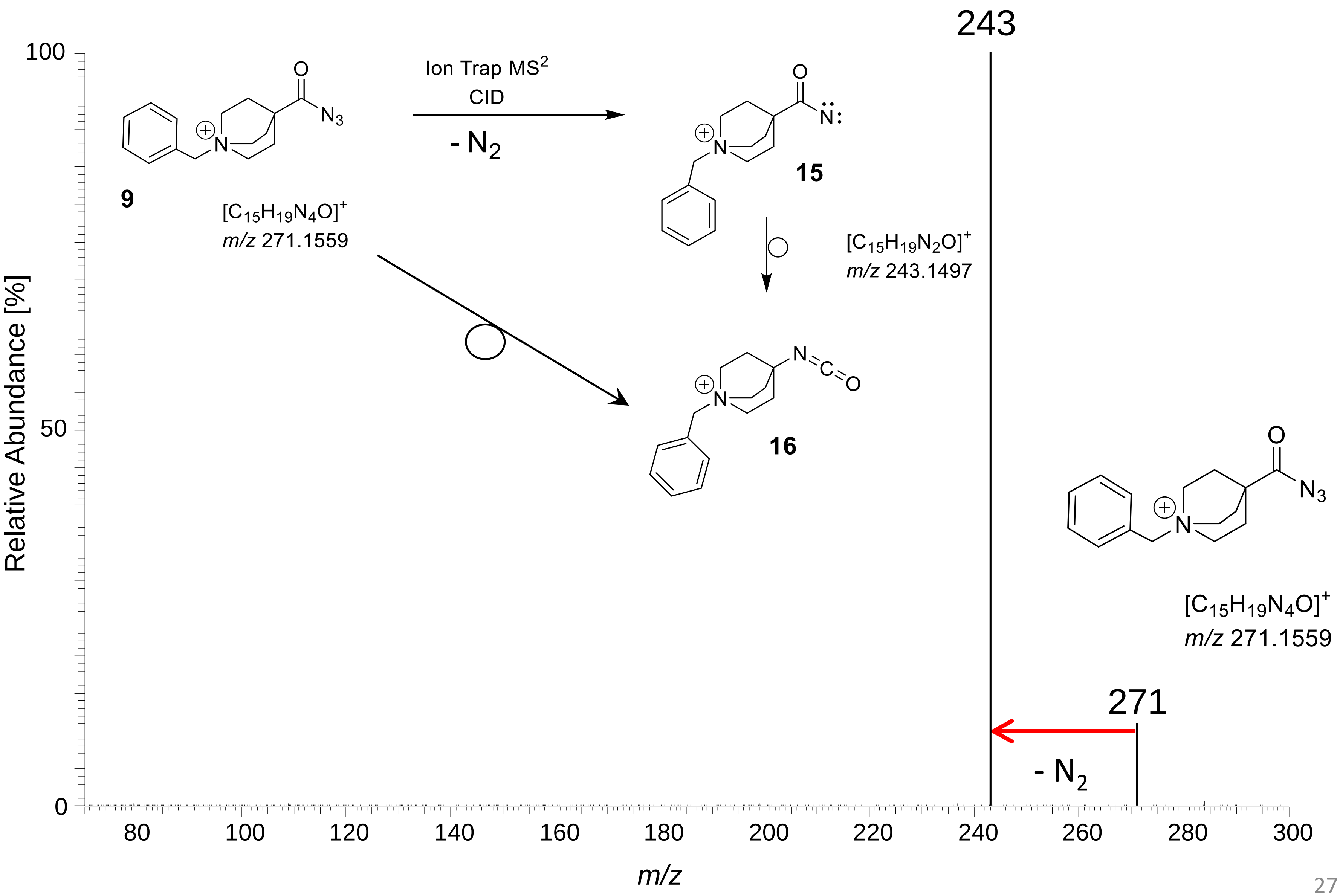

Figure S27. (+)ESI-MS^3 of the product ion at *m/z* 243 activated with NCE 24 formed from the molecular ion of analyte **9** at *m/z* 271. Collision activation performed in a linear quadrupole ion trap with He as inert collision gas and with normalized collision energy NCE of 13%.

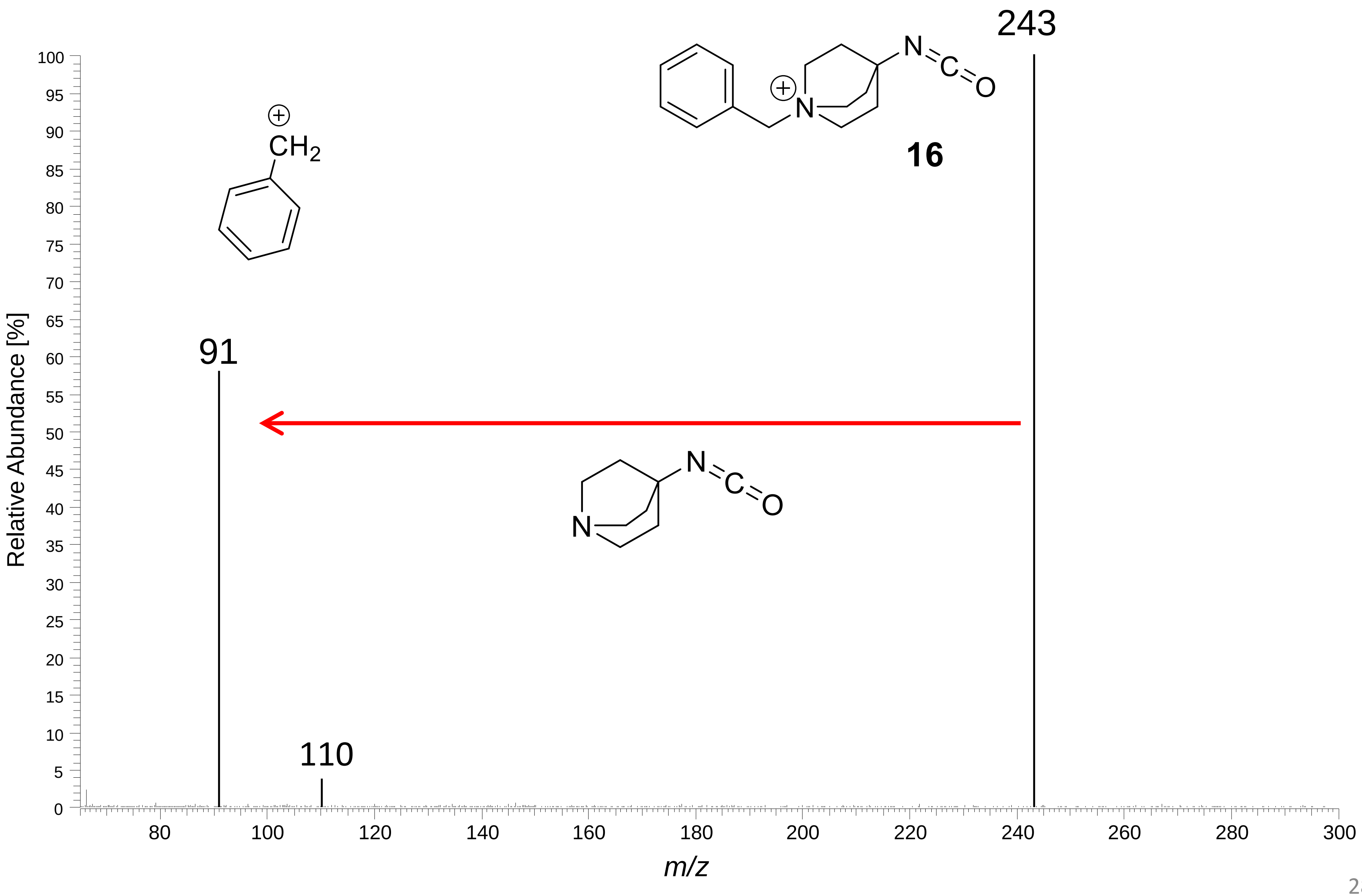

Figure S28. (+)ESI-MS[2] of the molecular ion of analyte **10** at *m/z* 270. Collision activation performed in a linear quadrupole ion trap with He as inert collision gas and with normalized collision energy NCE of 20%.

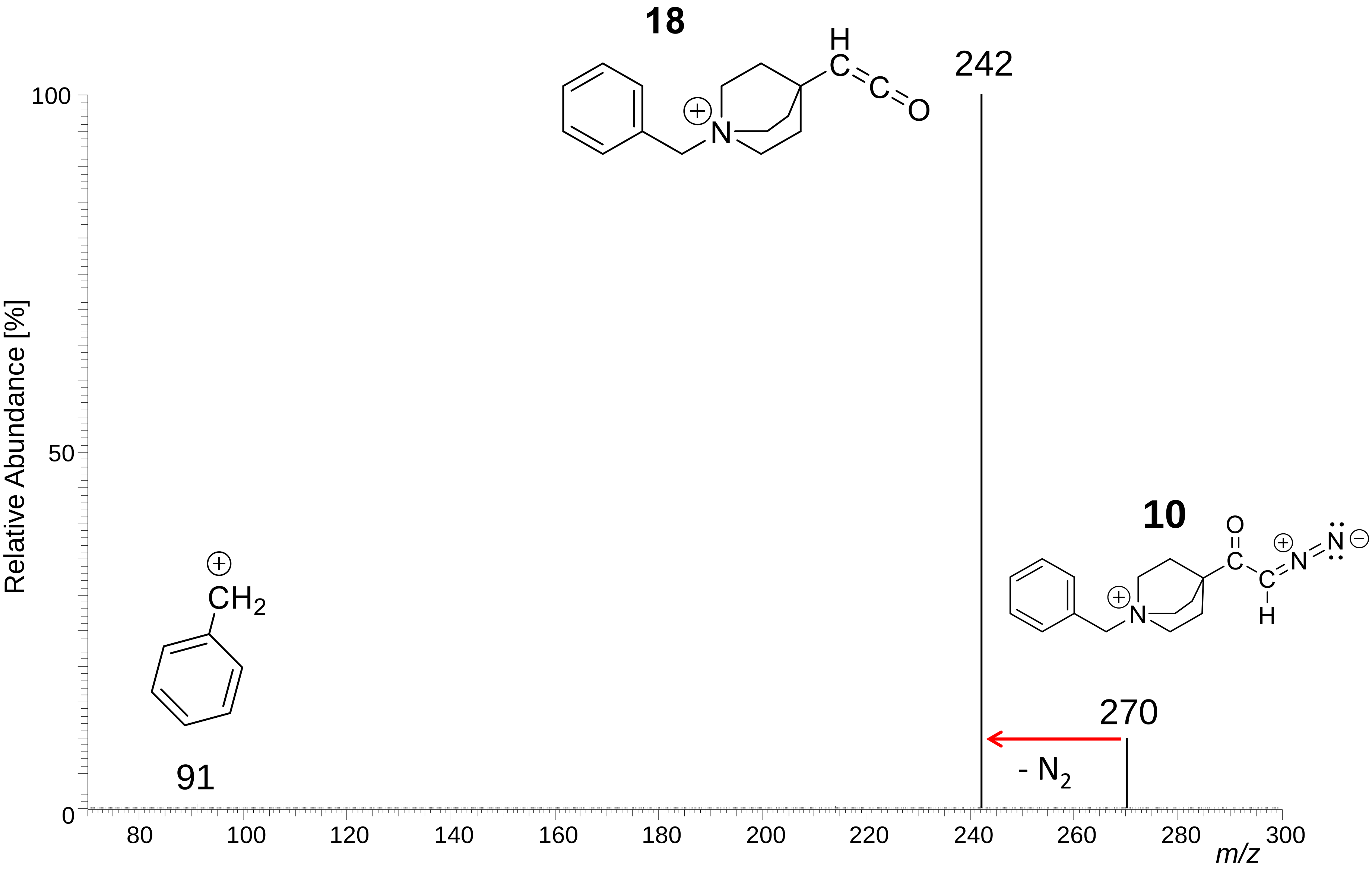

Supporting Information

to

***Gas-phase Curtius and Wolff* rearrangement reactions**
investigated by tandem-MS, IR ion spectroscopy and theory

by

Wacharee Harnying, Hui-Chung Wen, Jonathan Martens, Giel Berden, Jos Oomens,
Jana Roithová, Albrecht Berkessel, Mathias Schäfer and Anthony J.H.M. Meijer

Part III
IR Ion Spectroscopy IRIS

Table S3. Photofragmentation of the selected precursor ions used for IRIS. All ions are characterized by accurate ion masses that match the respective compositions and confirm the identity of the respective precursor ions (see Table S1).

Compound and precursor ion	Precursor Ion [m/z]	Photofragments at Laser Attenuation	
		0dB [m/z]	3dB [m/z]
**7** 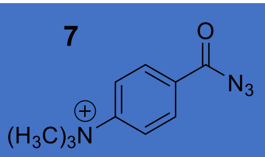	205	177, 163, 162, 161, 146, 145, 135, 134, 133, 119, 118	
$[7\text{-}N_2]^+$	177	162, 161, 146, 145, 135, 134, 133, 119, 118, 117, 116, 107, 106, 104, 93, 92, 91, 90, 79, 77	162, 161, 146, 135, 134, 133, 119, 118, 117, 116, 107, 106, 91, 90,
**8** 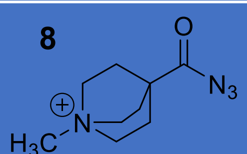	195	167, 139, 96, 81, 80, 71	
$[8\text{-}N_2]^+$	167	139, 114, 110, 97, 95, 94, 82, 80, 71, 70, 69, 59, 54, 53 (4 Laser Pulses / data point)	139, 96, 80, 71, 53
**9** 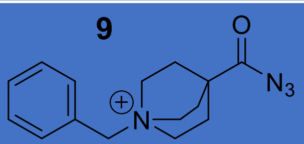	271	243, 151, 110, 92, 91	244, 243, 151, 110, 91
$[9\text{-}N_2]^+$	243	110, 91, 90	
$[8\text{-}N_2\text{–}C_2H_4]^+$	139	114, 97, 96, 95, 94, 84, 83, 71, 70, 69, 55, 54, 53 (4 Laser Pulses / data point)	97, 96, 71, 69, 55, 53
**10** 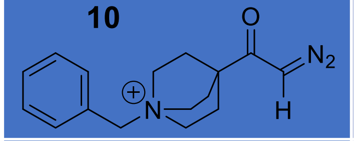	270	242, 214, 123, 122, 110, 95, 93, 91	242, 214, 123, 122, 110, 95, 93, 91
$[10\text{-}N_2]^+$	242	91	91

Figure S29. IR ion spectrum of the 4-(trimethylammonio) benzoyl azide precursor **7** at *m/z* 205 (blue shadowed trace) compared with the calculated, linear IR spectra of three structural alternatives: (c) isomer 3 harm. computed IR spectrum (39.6 kJ mol^{-1}); (b) isomer 2 harm. computed IR spectrum (0.2 kJ mol^{-1}); (a) isomer 1 harm. computed IR spectrum (0.0 kJ mol^{-1}). The harmonic IR spectra (green traces) are scaled by a factor of 0.97 below 2000cm^{-1} and by 0.95 above 2000 cm^{-1}. The signals are broadened by a factor of 25 in computed IR spectra.

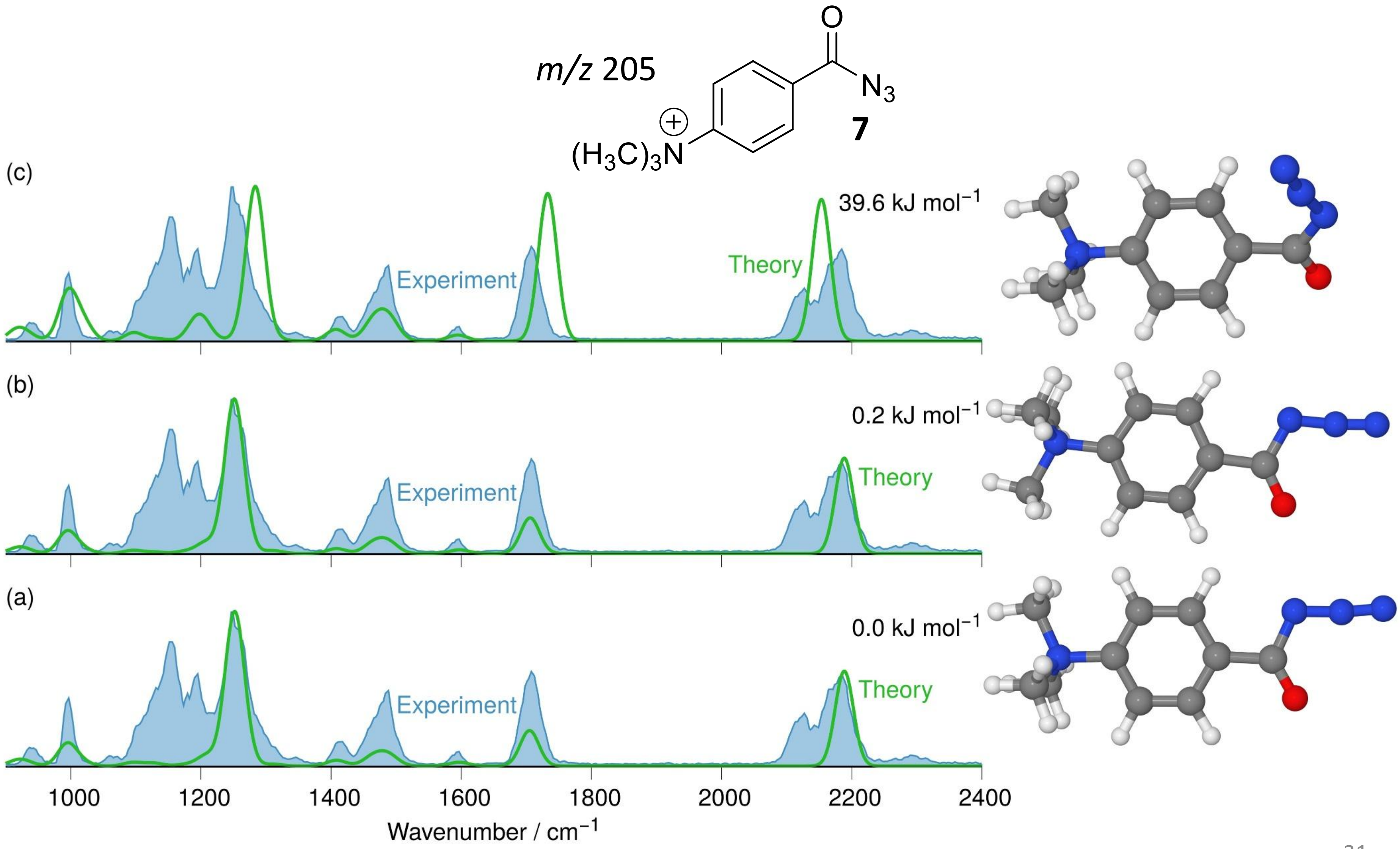

Figure S30. IR ion spectrum of the N_2-loss product ion at *m/z* 177 of precursor ion **7** (blue shadowed trace) compared with calculated IR spectra of the singlet isocyanate **12s** (0.0 kJ mol^{-1}). The anharm. computed spectrum (orange trace) is scaled by a factor 0.99 below 2000 cm^{-1} and by a factor of 0.955 above 2000 cm^{-1}. The harm. computed spectrum (green trace) is scaled by a factor 0.97 below 2000cm^{-1} and by a factor of 0.95 above 2000 cm^{-1}.

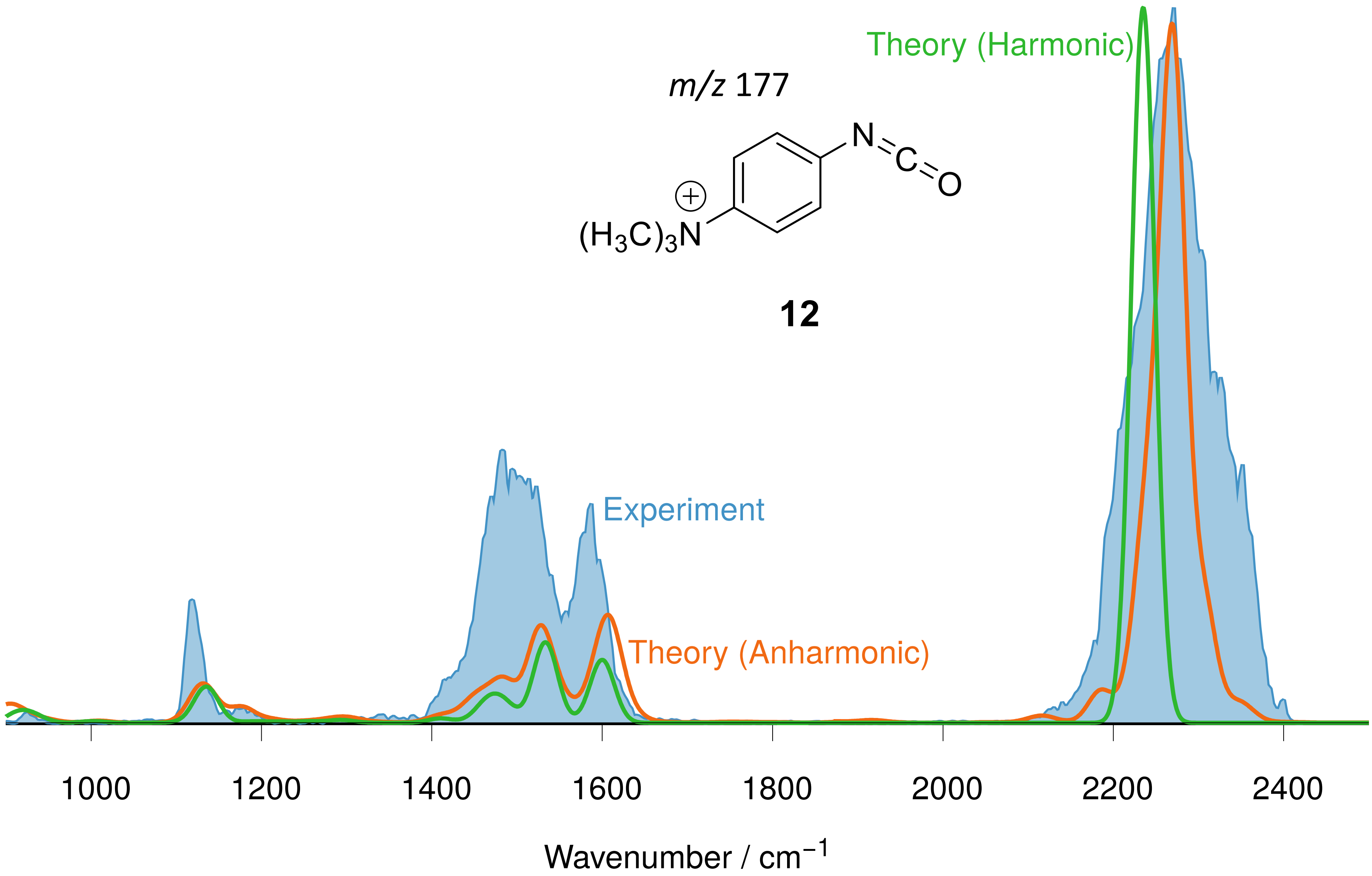

Figure S31. Isomer population analysis at the $\nu_{as\ R\text{-}N=C=O}$ band of the isocyanate ions **12** at *m/z* 177 formed by (+)ESI-MS^2 of the molecular ion of analyte **7** at *m/z* 205. With increasing number of laser pulses at 2250 cm^{-1} the signal is completely depleted (black trace: 2 laser pulses; blue trace: 4 laser pulses). This finding evidences that all ions present at *m/z* 177 absorb at that photon energy. This in turn documents that exclusively isocyanate ions **12** are present.

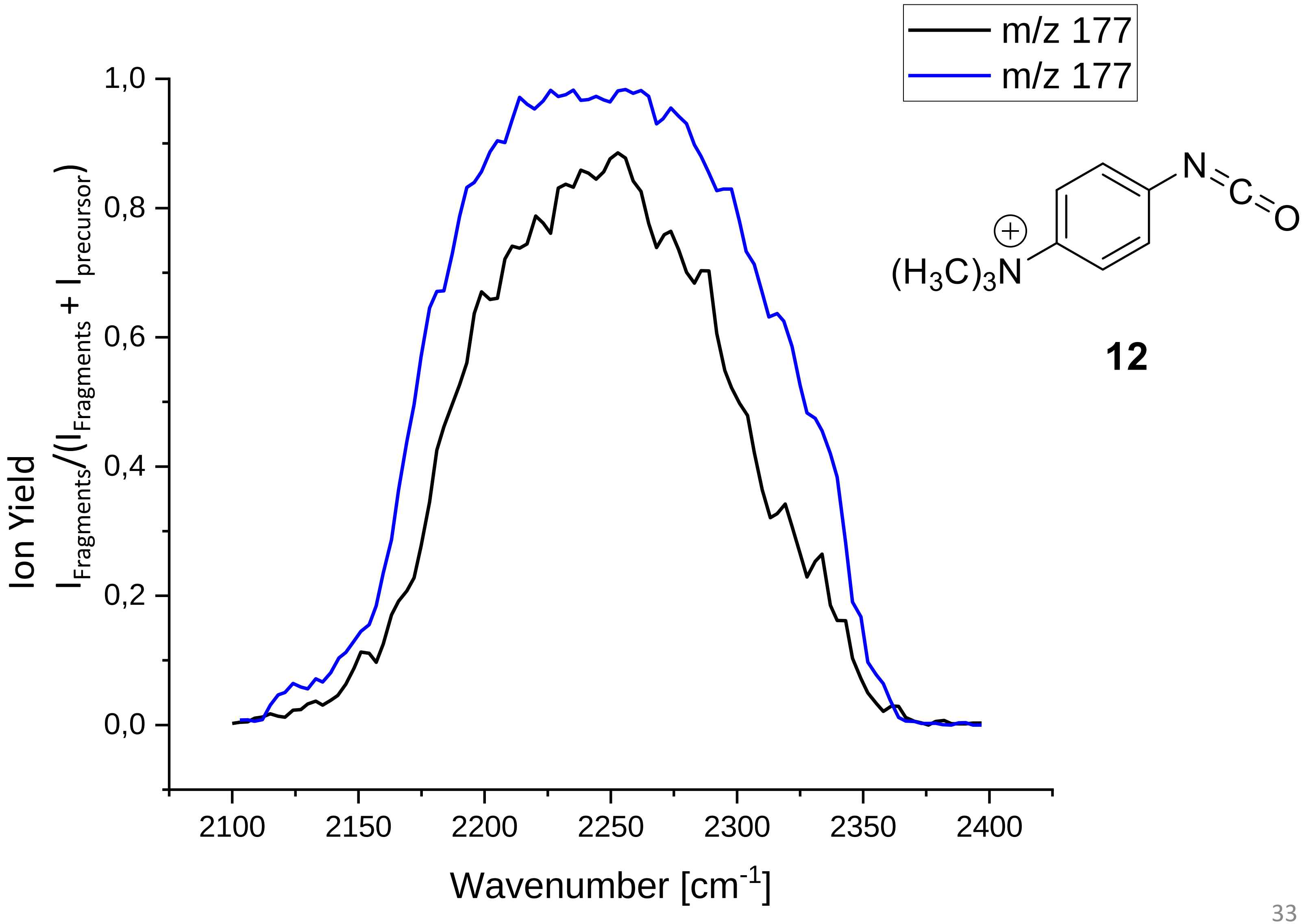

Figure S32. (+)ESI-MS IR ion spectrum of the N-methyl-4-quinuclidinium acyl azide precursor ion **8** at *m/z* 195 (blue shadowed trace) compared with the calculated, linear IR spectrum of the most stable ion structure (0.0 $kJmol^{-1}$) proposed by theory. The anharm. computed spectrum are scaled by a factor 0.99 below 2000cm^{-1} and by a factor of 0.955 above 2000 cm^{-1}. The signals are broadened by a factor of 25 in computed IR spectrum.

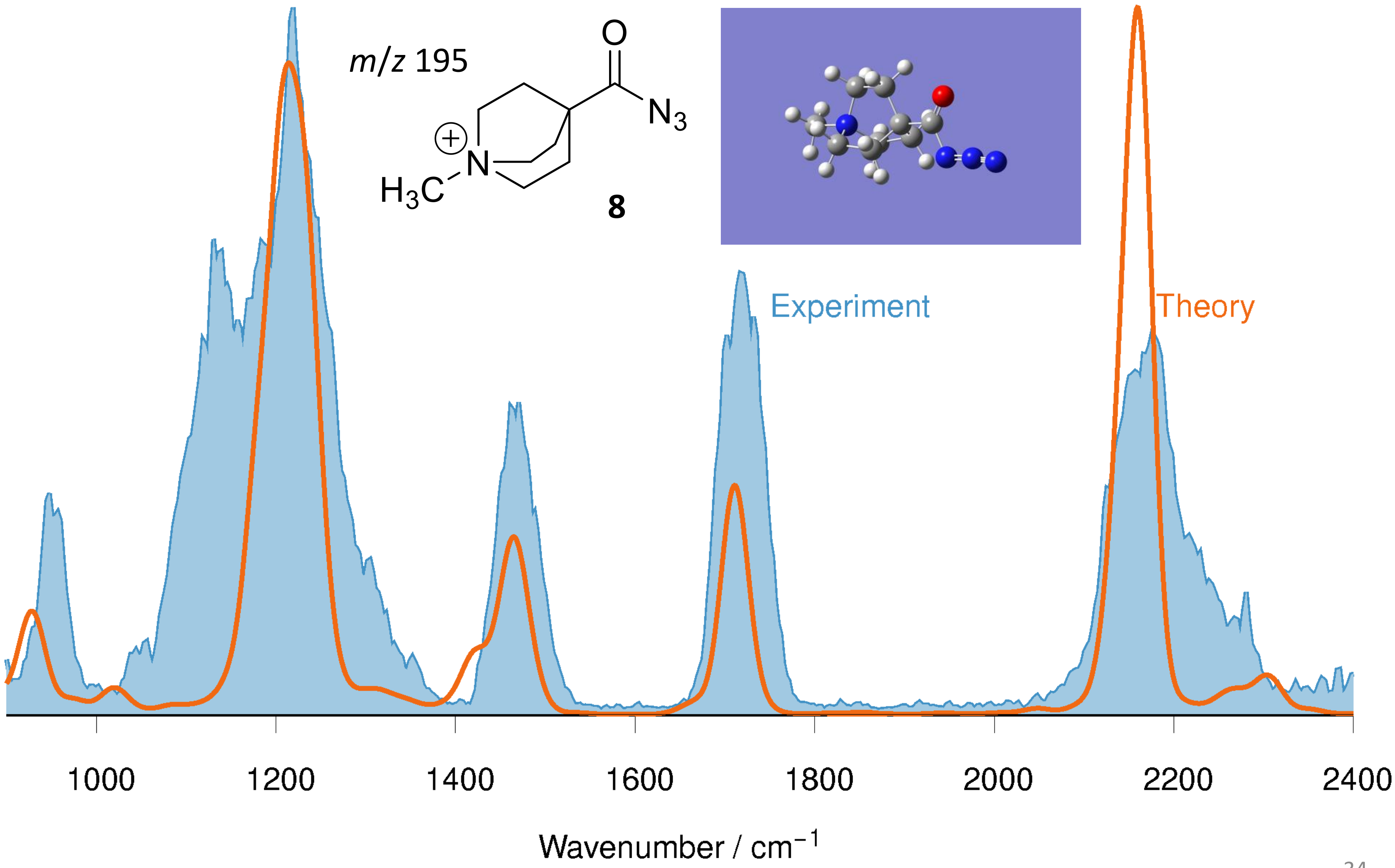

Figure S33. Isomer population analysis at the $\nu_{as\ R\text{-}N=C=O}$ band of the isocyanate ions at *m/z* 167 formed by (+)ESI-MS^2 of the molecular ion of analyte **8** at *m/z* 195. With increasing number of laser pulses at 2250 cm^{-1} the signal is nearly completely depleted after 2 laser pulses. This finding evidences that all ions present at *m/z* 167 absorb at that photon energy. This in turn documents that exclusively isocyanate ions **14** are present.

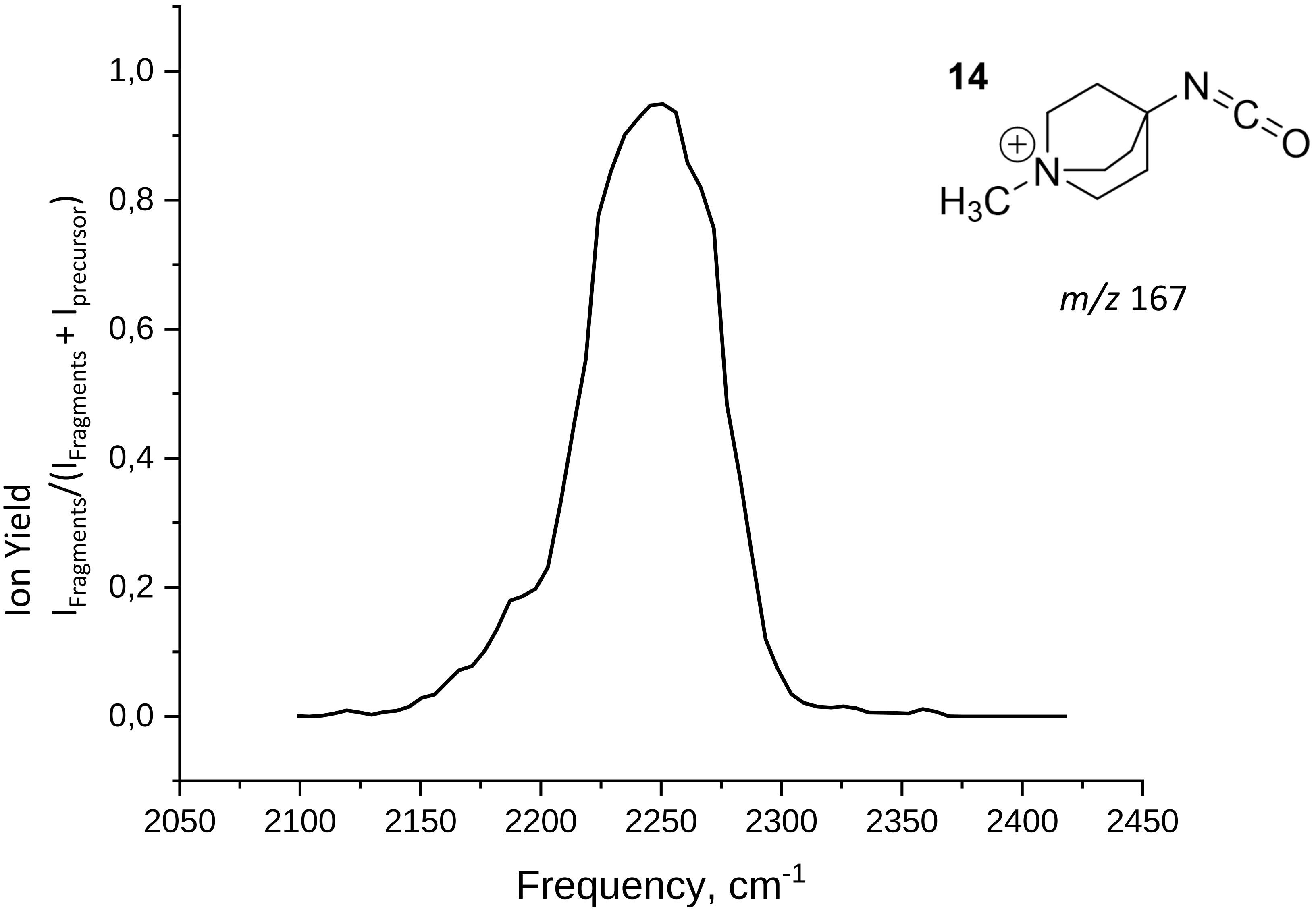

Figure S34. (+)ESI-MS IR ion spectrum of the N-benzyl-4-quinuclidinium acyl azide precursor ion **9** at *m/z* 271 (blue shadowed trace) compared with the calculated, linear IR spectrum of the most stable ion structure (0.0 kJmol^{-1}) proposed by theory. The anharm. computed spectrum are scaled by a factor 0.99 below 2000cm^{-1} and by a factor of 0.955 above 2000 cm^{-1}. The signals are broadened by a factor of 25 in computed IR spectrum.

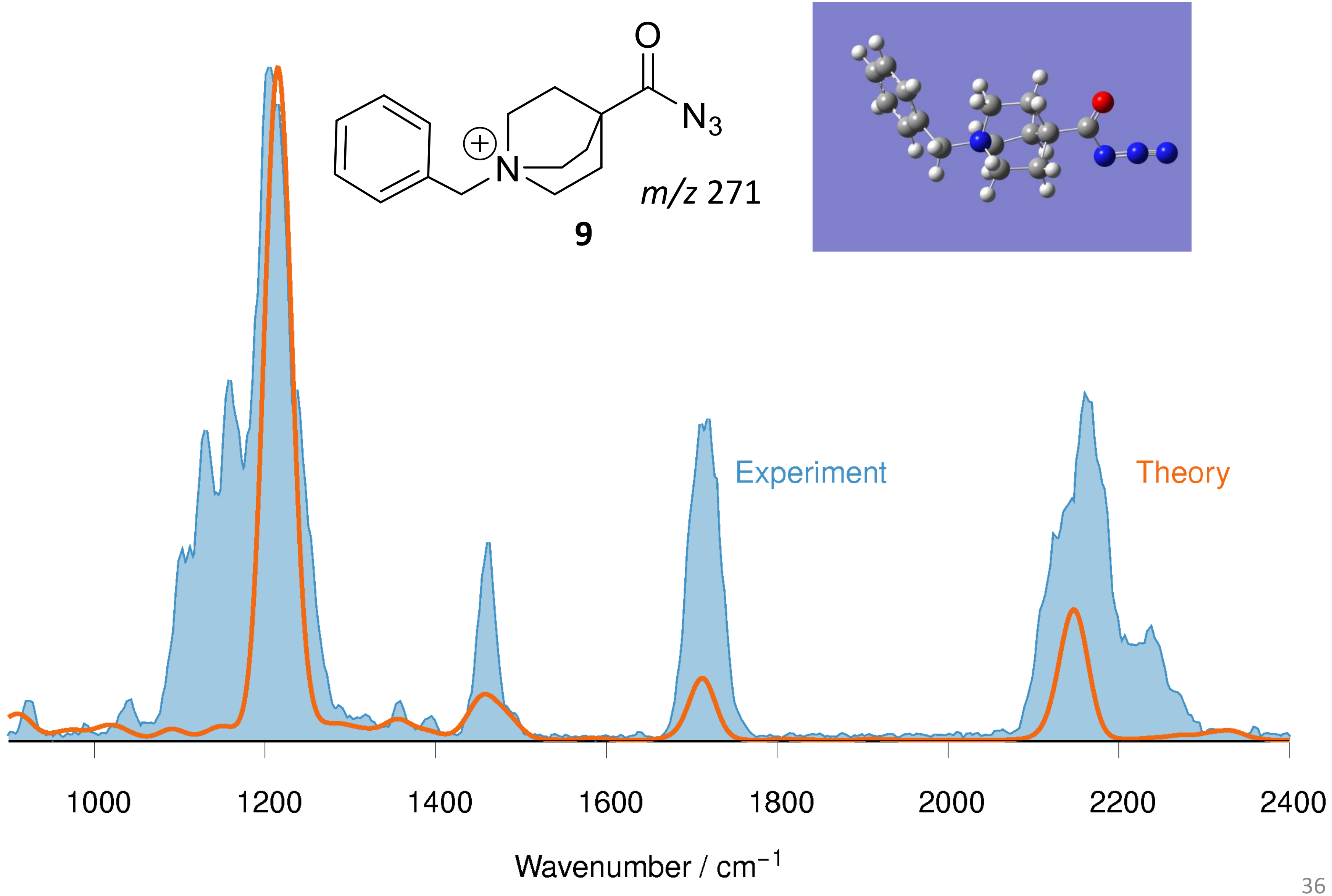

Figure S35. Isomer population analysis at the $\nu_{as\ R\text{-}N=C=O}$ band of the isocyanate ions at *m/z* 243 formed by (+)ESI-MS^2 of the molecular ion of analyte **9** at *m/z* 271. With increasing number of laser pulses at 2245 cm^{-1} the signal is completely depleted (black trace: 2 laser pulses; blue trace: 4 laser pulses). This finding evidences that all ions present at *m/z* 243 absorb at that photon energy. This in turn documents that exclusively isocyanate ions **16** are present.

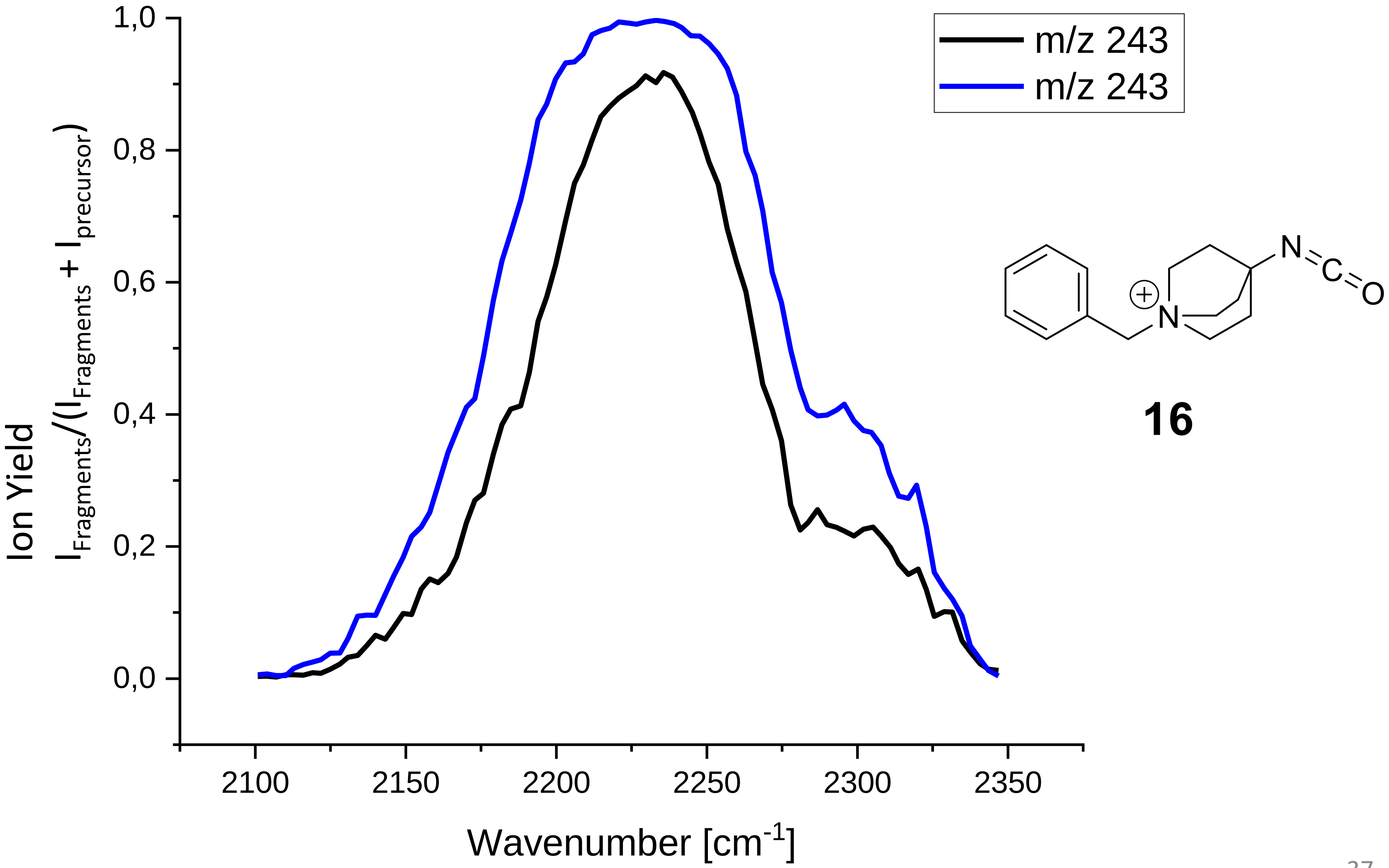

16

Figure S36. IR ion spectrum of the molecular ion of the charge-tagged benzyl quinuclidinium α-diazo carbonyl compound **10** at *m/z* 270 (blue shaddowed trace) compared with the harm. calculated IR spectra of two ion structures proposed by theory: (a) isomer 2 (+11.2 kJ mol^{-1}); (b) isomer 1 (0.0 kJ mol^{-1}). The harm. computed spectra are scaled by 0.97 in the range 800-2000cm^{-1} and by 0.95 in the range 2000-2400cm^{-1}.

10

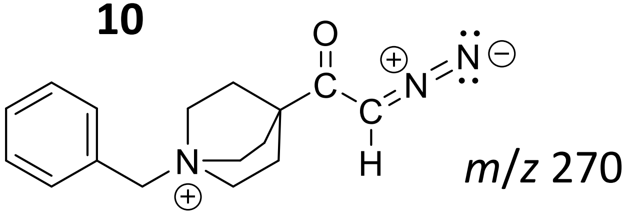

m/z 270

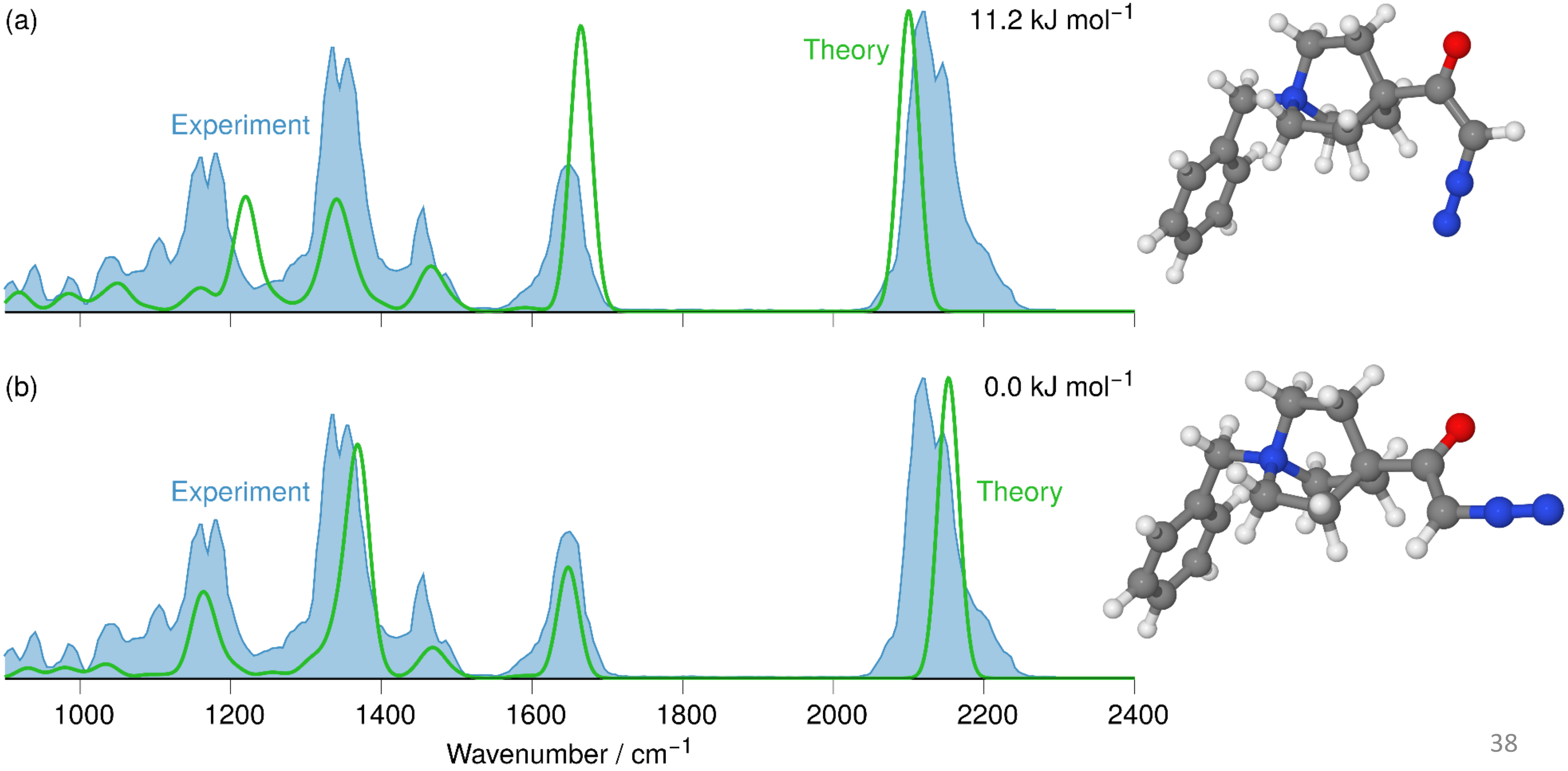

Table S4: Mode descriptions of the harmonic calculated vibrational modes of the ground structure (i.e. isomer 1; 0.0 kJmol^{-1}) of the 4-(trimethylammonio) benzoyl azide **7.** Harmonic scaling 0.97 (<2000) / 0.95 (>2000).

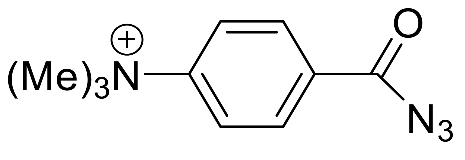

7

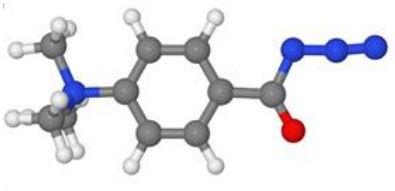

Mode #	Mode description	scaled ν_{vib} [cm^{-1}]
ν_1	Aromatic out-of-plane C-C bend.	536
ν_2	Out-of-plane C-CO-C bend., aromatic out-of-plane C-C bend.	679
ν_3	In-plane C-C bend.	730
ν_4	Aromatic out-of-plane C-C bend.	756
ν_5	$(CH_3)_3$N-C stretch.	819
ν_6	OC-N-N_2 bend.	859
ν_7	$(CH_3)_3$N-C stretch.	918
ν_8	$(CH_3)_3$N-C stretch.	933
ν_9	Aromatic in-plane C-C bend., OC-N_3 stretch.	995
ν_{10}	Aromatic in-plane C-C stretch., aromatic in-plane C-H bend.	1017
ν_{11}	$(CH_3)_3$N-C stretch., aromatic C-C stretch.	1095
ν_{12}	Methyl C-H rock., aromatic in-plane C-H bend.	1128
ν_{13}	Aromatic ring in-plane C-H bend., C-CON_3 stretch., N-N_2 stretch.	1209
ν_{14}	$(CH_3)_3$N-C stretch., C-CO-N_3 stretch.	1250
ν_{15}	$(CH_3)_3$N-C stretch., C-CO-N_3 stretch.	1260
ν_{16}	C-H_3 bend., In-plane C-H bend.	1409
ν_{17}	Methyl C-H twist.	1462
ν_{18}	Methyl C-H wagg. bend.	1468
ν_{19}	Methyl C-H_3 wagg. bend.	1485

ν_{20}	in-plane C-H bend.	1497
ν_{21}	Aromatic ring C=C stretch.	1598
ν_{22}	C-O stretch.	1705
ν_{23}	N_3 stretch.	2189

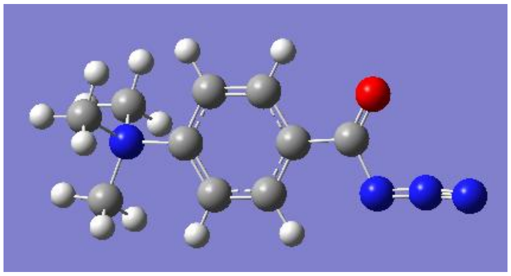

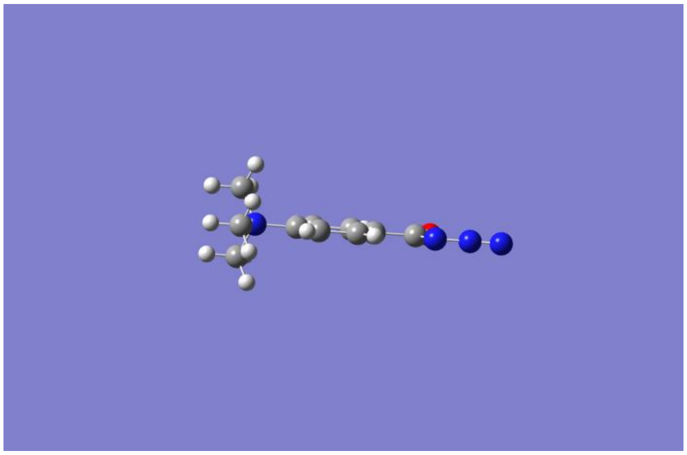

Table S5: Mode descriptions of the harmonic calculated vibrational modes of the ground structure (i.e. isomer 1; 0.0 kJmol^{-1}) of the N-methyl-4-quinuclidinium acyl azide **8.** Harmonic scaling 0.97 (<2000) / 0.95 (>2000).

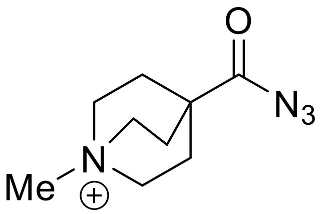

8

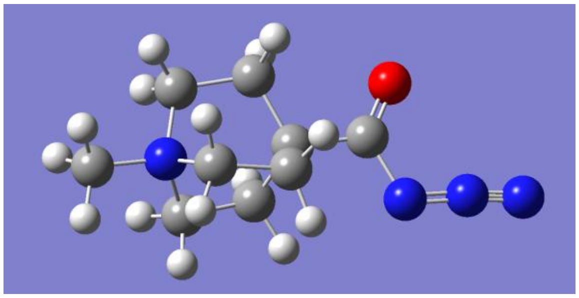

Mode #	Mode description	scaled ν_{vib} [cm^{-1}]
ν_1	CH_3-N stretch., OC-N-N_2 stretch.	857
ν_2	CH_3-N stretch., $(CH_2)_3$C-CO stretch., OC-N-N_2 stretch.	934
ν_3	CH_2-CH_2 stretch.	1021
ν_4	C-CO stretch.; C-H bend.	1157
ν_5	$(H\text{-}C\text{-}H)_2$ bend.	1226
ν_6	Methyl C-H twist.	1247
ν_7	H-C-H bend.	1469
ν_8	H-C-H bend.	1470
ν_9	C-O stretch.	1720
ν_{10}	N_3 stretch.	2189

Table S6: Mode descriptions of the harmonic calculated vibrational modes of the ground structure (i.e. isomer 1; 0.0 kJmol^{-1}) of the N-benzyl-4-quinuclidinium acyl azide **9.** Harmonic scaling 0.97 (<2000) / 0.95 (>2000).

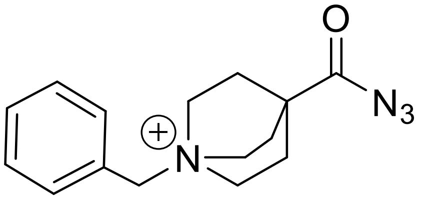

9

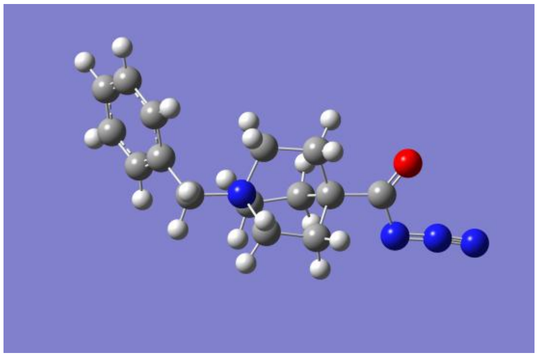

Mode #	Mode description	scaled ν_{vib} [cm^{-1}]
ν_1	Arom. out-of-plane C-C bend.	703
ν_2	Arom. out-of-plane C-H bend.	763
ν_3	Arom. out-of-plane C-H bend., $C_6H_5CH_2$-N stretch.	844
ν_4	$C_6H_5CH_2$-N stretch., CO-N-N_2 stretch.	914
ν_5	H_2C-CH_2 stretch., CO-N-N_2 stretch.	1017
ν_6	C-CO stretch.; C-H bend.	1147
ν_7	H_5C_6-CH_2 stretch.	1203
ν_8	H-C-H bend.	1218
ν_9	CO-N-N_2 stretch., H-C-H twist.	1227
ν_{10}	H-C-H twist.	1249
ν_{11}	H-C-H bend.	1471
ν_{12}	C-O stretch.	1720
ν_{13}	N_3 stretch.	2188

Table S7: Mode descriptions of the harmonic calculated vibrational modes of the ground structure (i.e. isomer 1; 0.0 kJmol^{-1}) of the N-benzyl-4-quinuclidinium diazo carbonyl compound compound **10.** Harmonic scaling 0.97 (<2000) / 0.95 (>2000).

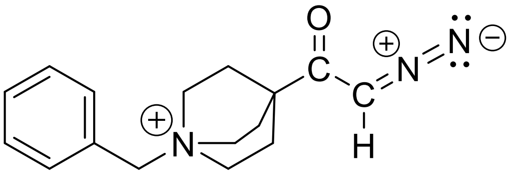

10

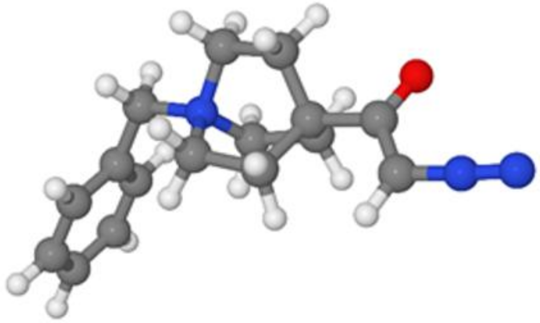

Mode #	Mode description	scaled ν_{vib} [cm^{-1}]
ν_1	OC-C-HN_2 bend.	493
ν_2	Arom. out-of-plane C-H bend.	703
ν_3	Arom. out-of-plane C-H bend.	763
ν_4	$C_6H_5CH_2$-N stretch., C-CON_3 stretch.	844
ν_5	OC-C-HN_2 bend., arom. in-plane C-H bend.	1160
ν_6	OC-C-HN_2 bend., H-C-H twist.	1175
ν_7	H_5C_6-CH_2 stretch., arom. C-C stretch.	1203
ν_8	H-C-H wagg., OC-C-HN_2 bend.	1345
ν_9	H_5C_6(H-C-H) bend., OC-C-HN_2 bend., H-C-H wagg.	1364
ν_{10}	OC-CHN_2 stretch., OCC-HN_2 bend.,	1371
ν_{11}	H-C-H bend.	1472
ν_{12}	C-O stretch.	1648
ν_{13}	CON-N stretch.	2153

Table S8: Mode descriptions of the harmonic calculated vibrational modes of the charge-tagged aromatic singlet nitrene **11s** (320.4 kJ mol^{-1}). The modes are uniformly scaled 0.97.

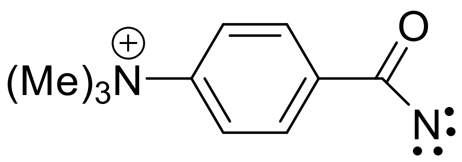

11s

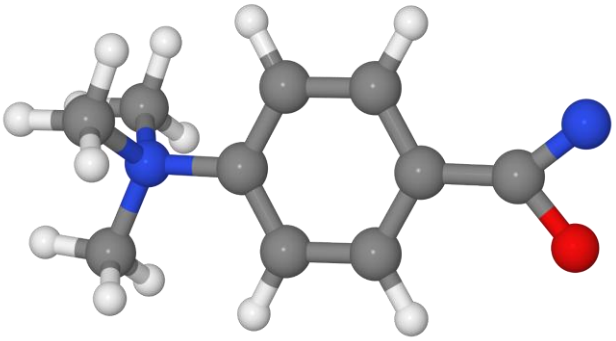

Mode #	Mode description	scaled ν_{vib} [cm^{-1}]
ν_1	Arom. out-of-plane C-C bend., out-of-plane C-C bend. O=C-N	601
ν_2	$(CH_3)_3$N-C stretch., Arom. C-C stretch.	821
ν_3	Arom. out-of-plane C-H bend.	841
ν_4	$(CH_3)_3$N-C stretch.	915
ν_5	$(CH_3)_3$N-C stretch.	930
ν_6	Aromatic in-plane C-C stretch., NC=O stretch.	1243
ν_7	Aromatic in-plane C-C stretch.	1414
ν_8	$(CH_3)_3$N-C bend.	1463
ν_9	$(CH_3)_3$N-C bend.	1469
ν_{10}	$(CH_3)_3$N-C bend.	1485
ν_{11}	Aromatic in-plane C-H bend.	1500
ν_{12}	N=CO stretch.	1763

Table S9: Mode descriptions of the harmonic calculated vibrational modes of the charge-tagged aromatic triplet nitrene **11t** (307.3 kJ mol^{-1}). The modes are uniformly scaled 0.97.

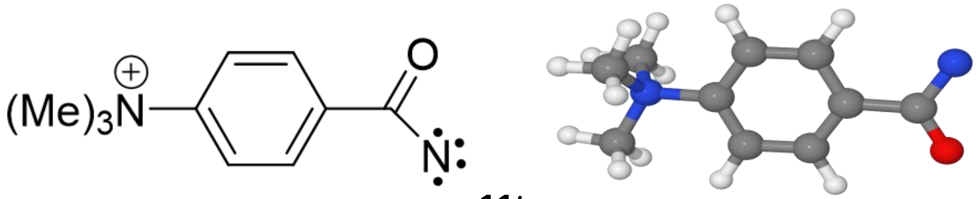

11t

Mode #	Mode description	scaled ν_{vib} [cm^{-1}]
ν_1	$(CH_3)_3N$ bend., O=C-N bend.	544
ν_2	$(CH_3)_3N$ bend., O=C-N bend.	624
ν_3	Arom. out-of-plane C-C bend., out-of-plane O=C-N bend.	631
ν_4	$(CH_3)_3N$-C stretch.	820
ν_5	Aromatic out-of-plane C-H bend.	844
ν_6	$(CH_3)_3N$-C stretch.	917
ν_7	$(CH_3)_3N$-C stretch.	931
ν_8	Aromatic in-plane HC-C-CH stretch.	1005
ν_9	O=C-N stretch., Aromatic in-plane HC-C-CH stretch.	1051
ν_{10}	$(CH_3)_3N$-C stretch., Aromatic in-plane HC-C-CH stretch.	1096
ν_{11}	Aromatic in-plane C-H bend.	1178
ν_{12}	ONC-C_6H_5 stretch., Aromatic in-plane C-H bend.	1201
ν_{13}	Aromatic in-plane C-CH-CH-C stretch.	1307
ν_{14}	Methyl C-H bend., Aromatic in-plane C-H bend.	1462
ν_{15}	Methyl C-H bend.	1468
ν_{16}	NC=O stretch.	1476
ν_{17}	Methyl C-H bend.	1485
ν_{18}	Aromatic in-plane CH-CH stretch.	1597

Table S10: Mode descriptions of the harmonic calculated vibrational modes of the charge-tagged aromatic singlet isocyanate **12s** (0.0 kJmol^{-1})**.** Harmonic scaling 0.97 (<2000) / 0.95 (>2000).

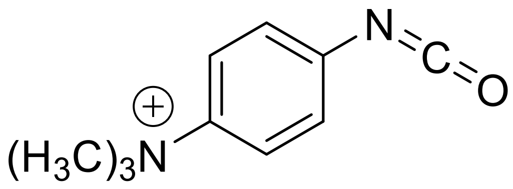

12

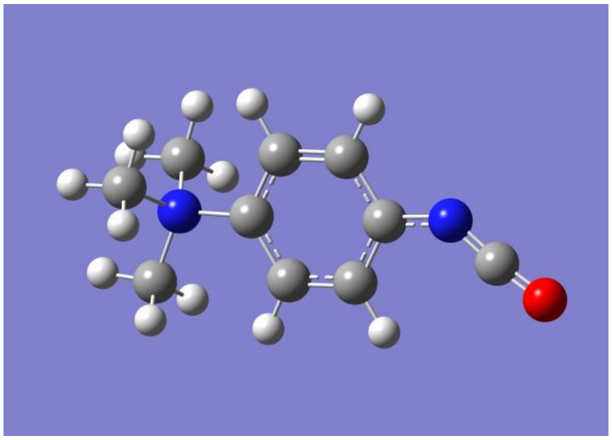

Mode #	Mode description	scaled ν_{vib} [cm^{-1}]
ν_1	Arom. out-of-plane C-C & C-H bend.	542
ν_2	N=C=O bend.	576
ν_3	Arom. in-plane C-C stretch., $C_6H_5CH_2$-N stretch.	824
ν_4	Arom. Out-of-plane C-H bend.,	837
ν_5	H_2C-CH_2 stretch., CO-N-N_2 stretch.	914
ν_6	Arom. in-plane C-H bend.	1131
ν_7	Arom. in-plane C-H bend.	1136
ν_8	$(CH_3)_3$N-C bend.	1485
ν_9	H_5C_6-NCO stretch., H_5C_6-N$(CH_3)_3$ stretch., CH-C-CH stretch.	1533
ν_{10}	Arom. CH-C-CH, CH-CH stretch.	1599
ν_{11}	N=C=O stretch.	2235

Table S11: Mode descriptions of the harmonic calculated vibrational modes of the charge-tagged quinuclidinium singlet nitrene **13s** (0.0 $kJmol^{-1}$)**.** The modes are uniformly scaled 0.97.

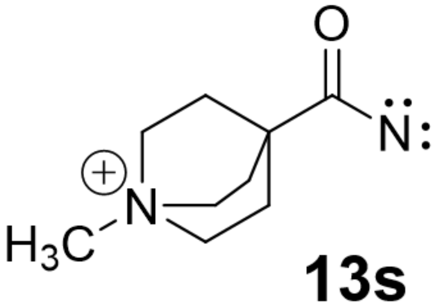

13s

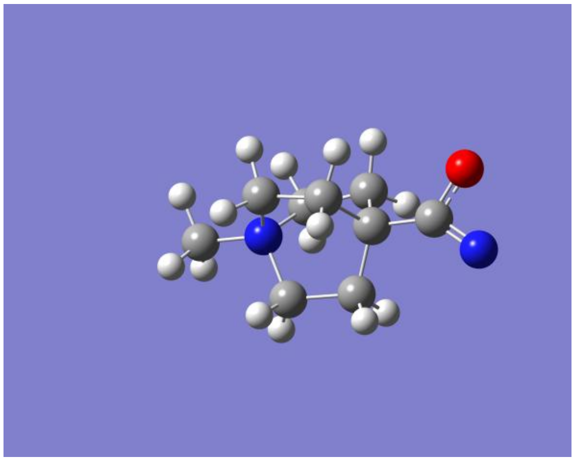

Mode #	Mode description	scaled ν_{vib} [cm^{-1}]
ν_1	H-C-H bend.	814
ν_2	CH_2-C-CH_2 stretch.	838
ν_3	CH_2-CH_2 stretch.	973
ν_4	CH_3-N stretch.	1121
ν_5	$(H\text{-}C\text{-}H)_2$ bend.; CON stretch.	1240
ν_6	H-C-H & CH_3 bend. wagg.	1470
ν_7	C-CNO stretch	1768

Table S12: Mode descriptions of the harmonic calculated vibrational modes of the charge-tagged quinuclidinium triplet nitrene **13t** (+309.9 kJ mol^{-1}). The modes are uniformly scaled 0.97.

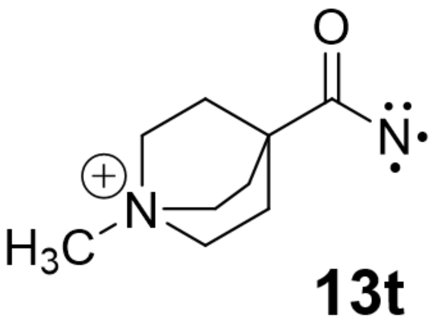

13t

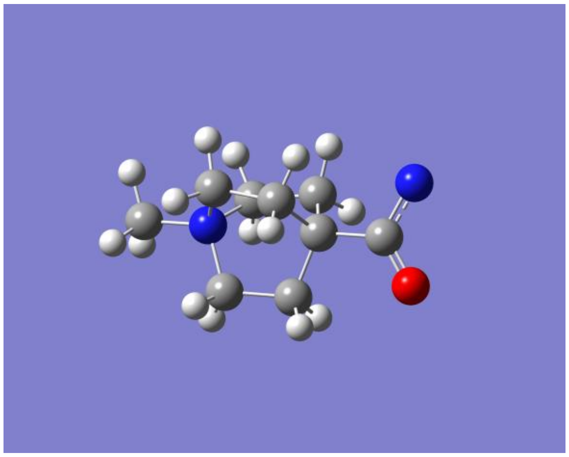

Mode #	Mode description	scaled ν_{vib} [cm^{-1}]
ν_1	CNO bend., CH_3-N stretch.	604
ν_2	CH_2 bend.	813
ν_3	CH_2-CH_2 stretch.	834
ν_4	CH_3-N stretch., Methyl C-H bend.	1116
ν_5	CH_3-N stretch., Methyl C-H bend.	1138
ν_6	H-C-H & CH_3 bend. wagg.	1194
ν_7	H-C-H & CH_3 bend. wagg.	1469
$\nu 8$	H-C-H & CH_3 bend. wagg.	1470
ν_9	H-C-H bend., CNO stretch.	1492
ν_{10}	C-CNO stretch	1495

Table S13: Mode descriptions of the harmonic calculated vibrational modes of the charge-tagged aromatic singlet isocyanate **14s** (0.0 kJmol^{-1}). Harmonic scaling 0.97 (<2000) / 0.95 (>2000).

14

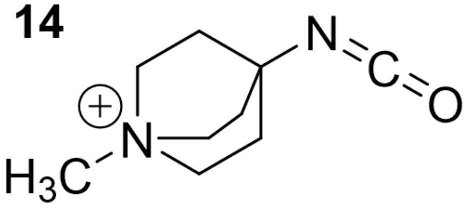

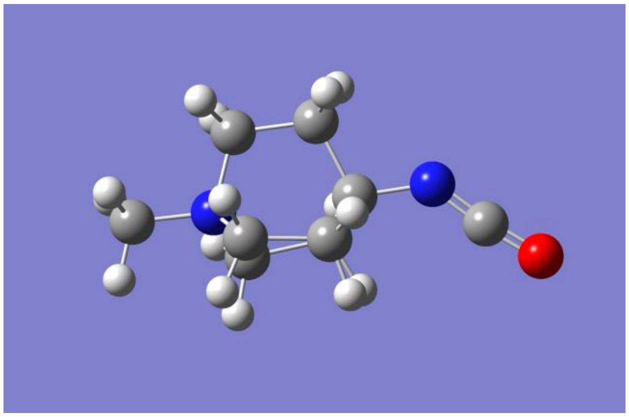

Mode #	Mode description	scaled ν_{vib} [cm^{-1}]
ν_1	CNO bend.	586
ν_2	CH_3-N-$(CH_2)_3$ stretch.	664
ν_3	CH_2-CH_2 stretch.	978
ν_4	CH_3-N stretch., CH_2-CH_2 stretch.	980
ν_5	CH_3-N stretch., CH_2-CH_2 stretch.	1044
ν_6	H-C-H wagg.	1346
ν_7	CNO stretch.	1444
$\nu 8$	H-C-H & CH_3 bend.	1469
ν_9	H-C-H & CH_3 bend.	1470
ν_{10}	C-CNO stretch	2232

Table S14: Mode descriptions of the harmonic calculated vibrational modes of the charge-tagged benzyl quinuclidinium singlet nitrene **15s** (+337.1 kJmol^{-1}). The modes are uniformly scaled 0.97.

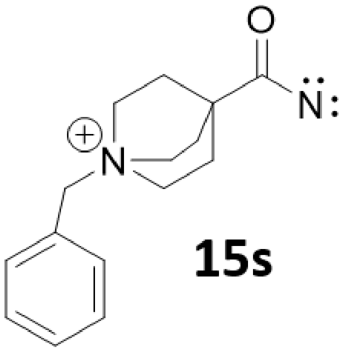

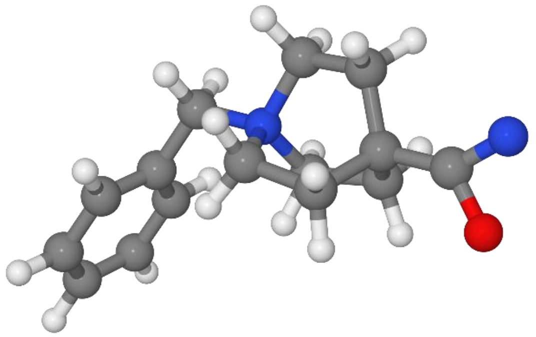

Mode #	Mode description	scaled ν_{vib} [cm^{-1}]
ν_1	Arom. out-of-plane C-C bend.	703
ν_2	Arom. out-of-plane C-H bend.	763
ν_3	C-CNO stretch., $C_6H_5CH_2$-N stretch.	940
ν_4	$C_6H_5CH_2$-N stretch.,	1036
ν_5	H_5C_6-CH_2N stretch.	1203
ν_6	H-C-H bend., NC=O stretch.	1237
ν_7	H_5C_6-HC-H bend., NH-C-H bend	1361
ν_8	H-C-H bend.	1468
ν_9	C-C=NO stretch.	1768

Table S15: Mode descriptions of the harmonic calculated vibrational modes of the charge-tagged benzyl quinuclidinium triplet nitrene **15t** (+313.5 kJmol^{-1}). The modes are uniformly scaled 0.97.

15t

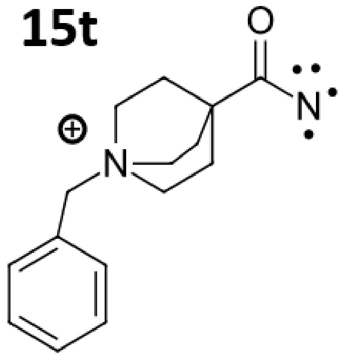

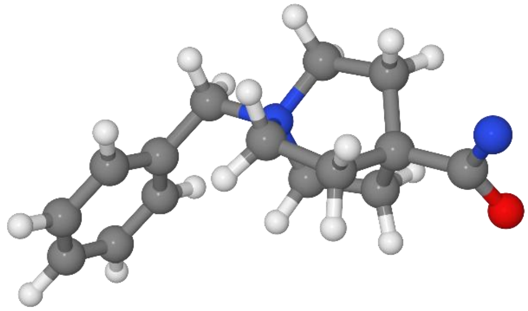

Mode #	Mode description	scaled ν_{vib} [cm^{-1}]
ν_1	Arom. in-plane C-C bend.	586
ν_2	Arom. out-of-plane C-H bend., H_5C_6-CH_2-N bend.	630
ν_3	Arom. out-of-plane C-H bend.	703
ν_4	Arom. out-of-plane C-H bend.	763
ν_5	H-C-H bend., H_5C_6-CH_2N stretch.	823
ν_6	H_5C_6-CH_2N stretch., H_2C-CH_2 stretch., Arom. out-of-plane C-H bend.	923
ν_7	H_2C-CH_2 stretch.	1034
ν_8	C-CNO stretch.	1121
ν_9	Arom. in-plane C-H bend.	1178
ν_{10}	H_5C_6-CH_2N stretch.	1204
ν_{11}	H-C-H bend., H_5C_6H-C-HN bend.	1363
ν_{12}	H-C-H bend.	1470
ν_{13}	NC=O stretch.	1495

Table S16: Mode descriptions of the harmonic calculated vibrational modes of the charge-tagged benzyl quinuclidinium singlet isocyanate **16s** (0.0 kJmol^{-1}). Harmonic scaling 0.97 (<2000) / 0.95 (>2000).

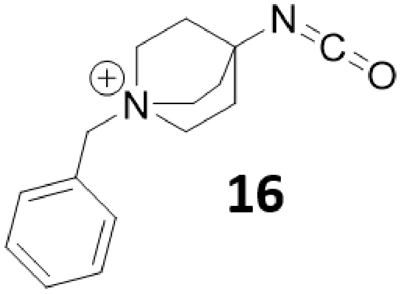

16

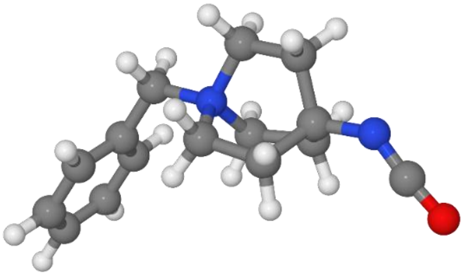

Mode #	Mode description	scaled ν_{vib} [cm^{-1}]
ν_1	N=C=O bend.	586
ν_2	N=C=O bend.	666
ν_3	Arom. out-of-plane C-H bend.	703
ν_4	Arom. out-of-plane C-H bend.	763
ν_5	$C_6H_5CH_2$-N stretch., C-NCO stretch.	939
ν_6	H-C-H bend.	1034
ν_7	C-N=C=O stretch.	1442
ν_8	H-C-H bend.	1468
ν_9	N=C=O stretch.	2232

Table S17: Mode descriptions of the harmonic calculated vibrational modes of the charge-tagged benzyl quinuclidinium carbonyl singlet carbene **17s** (+294.1 kJmol^{-1}). The modes are uniformly scaled 0.97.

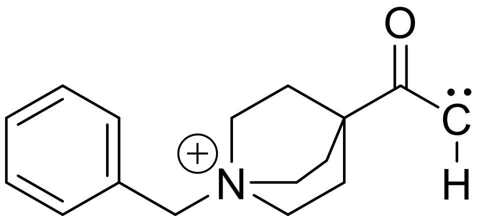

17s

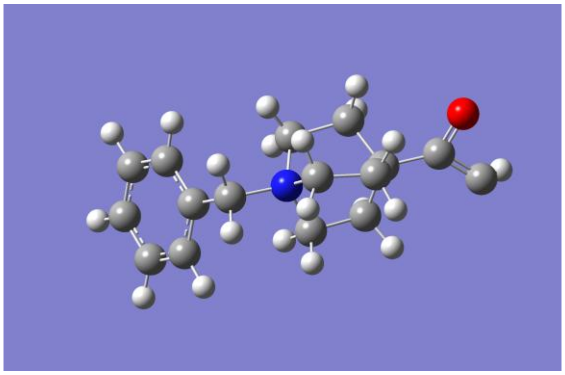

Mode #	Mode description	scaled ν_{vib} [cm^{-1}]
ν_1	COC-H bend.	465
ν_2	COC-H bend.	494
ν_3	COC-H bend.	664
ν_4	Arom. out-of-plane C-H bend.	703
ν_5	Arom. out-of-plane C-H bend.	763
ν_6	H-C-H bend.	815
ν_7	H_5C_6CH2-N stretch., H_2C-CH_2 stretch.	936
ν_8	H_5C_6CH2-N stretch., H_2C-CH_2 stretch.	1036
ν_9	C-COCH stretch.	1092
ν_{10}	H_5C_6-CH_2 stretch.	1203
ν_{11}	H-C-H bend.	1327
ν_{12}	O=C-CH stretch.	1388
ν_{13}	H-C-H bend.	1461
ν_{14}	C-COCH stretch.	1576

Table S18: Mode descriptions of the harmonic calculated vibrational modes of the charge-tagged benzyl quinuclidinium carbonyl triplet carbene **17t** (+288.4 kJmol^{-1}). The modes are uniformly scaled 0.97.

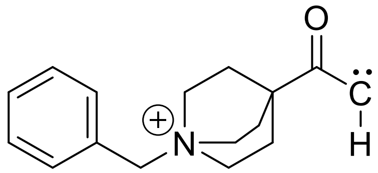

17t

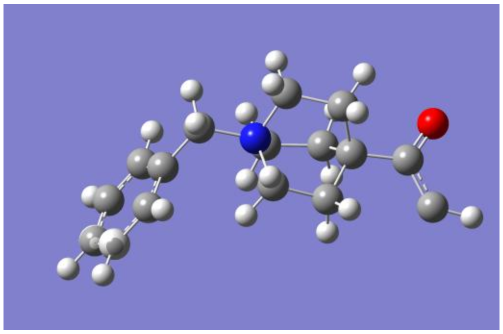

Mode #	Mode description	scaled ν_{vib} [cm^{-1}]
ν_1	in-plane C-C bend., CH_2-N-C_3 bend.	583
ν_2	COC-H bend., H_5C_6-CH_2 bend.	627
ν_3	Arom. out-of-plane C-H bend.	703
ν_4	Arom. out-of-plane C-H bend.	763
ν_5	$H_5C_6CH_2$-N stretch., H-C-H bend.	825
ν_6	$H_5C_6CH_2$-N stretch.	894
ν_7	$H_5C_6CH_2$-N stretch.	935
ν_8	$H_5C_6CH_2$-N stretch., H_2C-CH_2 stretch.	1035
ν_9	H_5C_6-CH_2 stretch.	1203
ν_{10}	H-C-H bend.	1238
ν_{11}	H-C-H bend.	1271
ν_{12}	H-C-H bend.	1289
ν_{13}	H-C-H bend.	1326
ν_{14}	H-C-H bend.	1361
ν_{15}	H-C-H bend.	1395
ν_{16}	H-C-H bend.	1460
ν_{17}	C=O stretch.	1483

Table S19: Mode descriptions of the harmonic calculated vibrational modes of the charge-tagged benzyl quinuclidinium singlet ketene **18s** (0.0 kJmol^{-1}). Harmonic scaling 0.97 (<2000) / 0.95 (>2000).

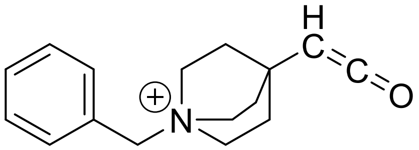

18

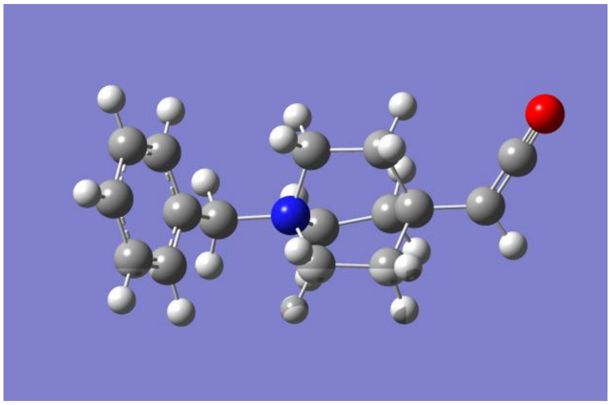

Mode #	Mode description	scaled ν_{vib} [cm^{-1}]
ν_1	H-CCCO bend.	582
ν_2	Arom. out-of-plane C-H bend.	703
ν_3	Arom. out-of-plane C-H bend.	763
ν_4	$H_5C_6CH_2$-N stretch., H-C-H bend.	834
ν_5	$H_5C_6CH_2$-N stretch.	940
ν_6	H_5C_6-CH_2 stretch.	1204
ν_7	H-C-H bend.	1327
ν_8	H-C-H bend.	1361
ν_9	H-C-H bend.	1466
ν_{10}	H-C-H bend.	1495
ν_{11}	HC=C=O stretch.	2113

Table S20: Mode descriptions of the harmonic calculated vibrational modes of the 1-methylene-1-piperidinium isocyanate cation **B** (+15.8 kJmol^{-1}). Harmonic scaling 0.97 (<2000) / 0.95 (>2000).

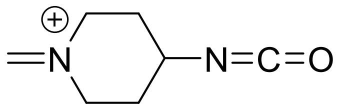

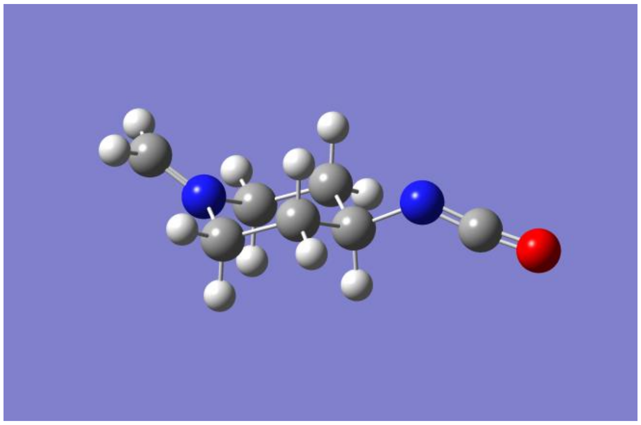

Mode #	Mode description	scaled ν_{vib} [cm^{-1}]
ν_1	CNO bend.	578
ν_2	CH_2=N-(CH_2)$_3$ bend.	620
ν_3	CH_2=N-(CH_2)$_3$ bend., CNCO bend.	655
ν_4	C=N=O stretch.	1441
ν_5	H-C-HN bend., H-C-H bend.	1468
ν_6	N=C=O stretch.	2238

Table S21: Mode descriptions of the harmonic calculated vibrational modes of the 1-azabi-cyclo[2.2.1]heptanium isocyanate ion **C** (0.0 kJmol^{-1}). Harmonic scaling 0.97 (<2000) / 0.95 (>2000).

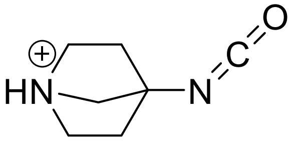

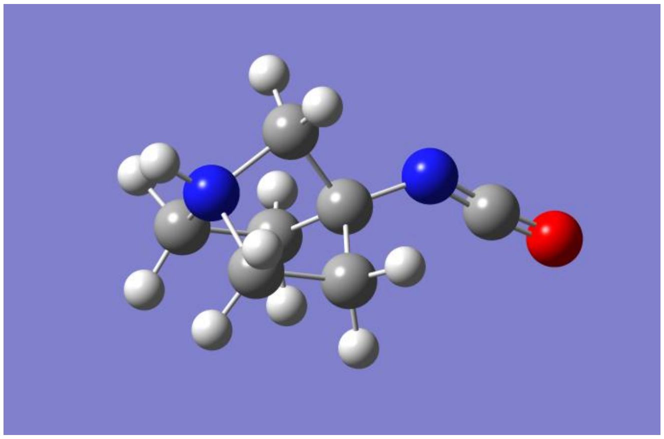

Mode #	Mode description	scaled ν_{vib} [cm^{-1}]
ν_1	CNO bend.	665
ν_2	C-NCO stretch.	1078
ν_3	H-C-H bend., C-NCO stretch.	1469
ν_4	H-C-H bend.	1499
ν_5	N=C=O stretch.	2242

Table S22. Mode descriptions of the significant anharmonic absorption bands of isomer 1 (0.0 kJmol^{-1}) of the charge-tagged aromatic carbonyl azide precursor ion **7**. Anharmonic scaling: 0.99 below 2000cm^{-1} and by 0.955 above 2000 cm^{-1}.

**IRIS Bands of the ions at *m/z* 205** (cm^{-1})	**IR Modes of isomer 1 of** 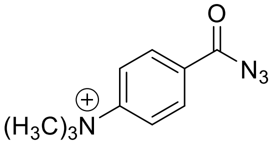 **7**	**Anharmonic Mode description**
2127	2124	Combination band of OC-N_3 stretch. mode and $(CH_3)_3$NC-CH bend. modes with aromatic C-H bend. Modes with C-CON_3 stretch. mode
	2134	Fundamental N=N=N stretch. mode
2185	2165	Combination band of OC-N_3 stretch. mode and $(CH_3)_3$NC-CH bend. modes with C-CON_3 stretch. mode and $(CH_3)_3$NC-CH stretch. modes
	2178	Combination band of OC-N_3 stretch. mode and $(CH_3)_3$NC-CH bend. modes with C-CON_3 stretch. mode and $(CH_3)_3$NC-CH stretch. modes

Table S23. Mode descriptions of the significant anharmonic absorption bands of isomer 1 (0.0 kJmol^{-1}) of the charge-tagged methyl quinuclidinium carbonyl azide precursor ion **8**. Anharmonic scaling: 0.99 below 2000cm^{-1} and by 0.955 above 2000 cm^{-1}.

**IRIS Bands of the ions at *m/z* 195** (cm^{-1})	**IR Modes of isomer 1 of** 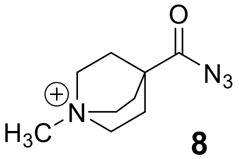 **8**	**Anharmonic Mode description**
2156	2138	Combination band of OC-N_3 stretch. mode and $(CH_3)_3$NC-CH bend. modes with quinuclidine C-H bend. modes
	2148	Combination band of N-CH_3 stretch. and quinuclidine core CH_2 bend. modes with C-CO stretch. and quinuclidine C-H bend. modes
2280	2163	Combination band of quinuclidine core CH_2-CH_2 bend. modes with the C=O stretch. mode

Table S24. Mode descriptions of the significant anharmonic absorption bands of isomer 1 (0.0 kJmol^{-1}) of the charge-tagged benzyl quinuclidinium carbonyl azide precursor ion **9**. Anharmonic scaling: 0.99 below 2000cm^{-1} and by 0.955 above 2000 cm^{-1}.

**IRIS Bands of the ions at *m/z* 271** (cm^{-1})	**IR Modes of isomer 1 of** **9** 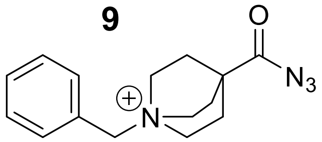	**Anharmonic Mode description**
2160	2134	Combination band of $C_6H_5H_2C$-N stretch. mode with aromatic in-plane CH bend. modes
	2146	Combination band of quinuclidine core CH_2-CH_2 stretch. and quinuclidine N-CH_2 stretch. modes with quinuclidine and benzylic CH_2 bend. and OCN=N_2 stretch. modes
	2155	Fundamental N=N=N stretch. mode
2230		

Table S25. Mode descriptions of the significant anharmonic absorption bands of the singlet aromatic isocyanate **12s** (0.0 $kJmol^{-1}$) important for the depletion scan. Anharmonic scaling: 0.99 below 2000cm^{-1} and by 0.955 above 2000 cm^{-1}.

**IRIS Modes of the ions at *m/z* 177** (cm^{-1})	**IR Modes of singlet isocyanate of** 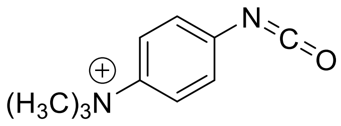 **12**	**Anharmonic Mode description**
2271	2275	Combination band of methyl C-H bend. and aromat. C-H bending modes
2303	2305	Combination band of $(CH_3)_3$N-C bend., aromat. C-CH bend. modes with aromat. C-H bend. and Methyl C-H bend. modes
2319	2341	Combination band of N-CH_3 stretch., C-NCO stretch. and aromat. C-CH bend. modes with other C-NCO stretch. and aromat. C-CH bend. modes

Table S26. Mode descriptions of the significant anharmonic absorption bands of the methyl quinuclidinium singlet isocyanate **14s** (0.0 $kJmol^{-1}$) important for the depletion scan. Anharmonic scaling: 0.99 below 2000cm^{-1} and by 0.955 above 2000 cm^{-1}.

**IRIS Modes of the ions at *m/z* 167** (cm^{-1})	**IR Modes of** **14** 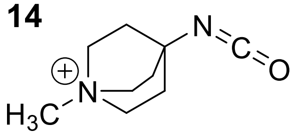	**Anharmonic Mode description**
2255	2188	Fundamental N=C=O stretch. mode
2195	2204	Combination band of CH_3 bend. quinuclidine core CH_2 bend. modes with other quinuclidine core CH_2 bend. modes

Table S27. Mode descriptions of the significant anharmonic absorption bands of the rearranged C_2H_4-loss product at *m/z* 139, the piperidinium isocyanate **B** (+15.8 kJmol^{-1}). Anharmonic scaling: 0.99 below 2000cm^{-1} and by 0.955 above 2000 cm^{-1}.

**IRIS Modes of the ions at *m/z* 139** (cm^{-1})	**IR Modes of C_2H_4-loss product B** 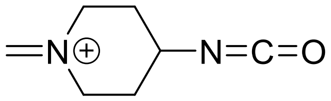	**Anharmonic Mode description**
2207	2183	Combination band of H_2C=N bend. and CH_2-C-CH_2 stretch. modes with C-H bend. modes of the NCH_2 group and C-H bend. modes of quinuclidine methylene CH_2 groups
2227	2189	Combination band of C-H bend. modes of quinuclidine methylene CH_2 groups with other C-H bend. modes of quinuclidine methylene CH_2 groups and C-NCO stretch mode
2247	2196	Combination band of HC-NCO stretch modes and OCNCH-CH_2 stretch. modes with a OCNC-H bending mode
2301	2211	Fundamental N=C=O stretch. mode

Table S28. Mode descriptions of the significant anharmonic absorption bands of the rearranged C_2H_4-loss product at *m/z* 139, the isocyanate **C** (0.0 kJmol^{-1}). Anharmonic scaling: 0.99 below 2000cm^{-1} and by 0.955 above 2000 cm^{-1}.

**IRIS Modes of the ions at *m/z* 139** (cm^{-1})	**IR Modes of C_2H_4-loss product C** 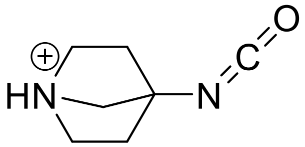	**Anharmonic Mode description**
2207	2186	Combination band of H_2C-CH_2 stretch. and methylene H-C-H wagging modes
2227	2201	Overtone CH_2 twisting modes
2247	2214	Fundamental N=C=O stretch. mode
2301		

Table S29. Mode descriptions of the significant anharmonic absorption bands of the singlet benzyl quinuclidinium isocyanate **16s** (0.0 kJmol^{-1}) important for the depletion scan. Anharmonic scaling: 0.99 below 2000cm^{-1} and by 0.955 above 2000 cm^{-1}.

**IRIS Modes of the ions at *m/z* 243** (cm^{-1})	**IR Modes of isomer 1 of 16** 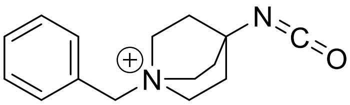	**Anharmonic Mode description**
2237	2186	$C_6H_5CH_2N$-$(CH_2)_3$ stretch. modes and C-H bending of quinuclidine methylene CH_2 groups with other C-H bending modes of quinuclidine methylene CH_2 groups
	2213	Fundamental N=C=O stretch. mode

PART IV

CALCULATIONS AND THEORY

TABLE OF CONTENTS

Figure S37: Potential energy surface (PES) of the N_2-loss reaction of the aromatic carbonyl azide **7** via the respective nitrenes **11s/11t** in either a stepwise or a concerted Curtius rearrangement reaction to the isocyanate **12**. All calculations at B3LYP-D3(BJ)//cc-pVTZ level of theory. 70

Figure S38: Potential energy surface (PES) of the N_2-loss reaction of the aromatic carbonyl azide **7** via the respective nitrenes **11s/11t** in either a stepwise or a concerted Curtius rearrangement reaction to the isocyanate **12**. Optimization at B3LYP-D3(BJ)//cc-pVTZ level of theory with energies at CASPT2//cc-pVTZ. 71

Figure S39: Potential energy surface (PES) of the N_2-loss reaction of the methyl quinuclidinium carbonyl azide **8** via the respective nitrenes **13s/14t** in either a stepwise or a concerted Curtius rearrangement reaction to the isocyanate **14**. All optimizations at B3LYP-D3(BJ)//cc-pVTZ level of theory. Singlet energies: CCSD(T)-F12b//cc-pVDZ-F12. Triplet energies: CASPT2//cc-pVTZ 72

Figure S40: Potential energy surface (PES) of the N_2-loss reaction of the benzyl quinuclidinium carbonyl azide **9** via the respective nitrenes **15s/15t** in either a stepwise or a concerted Curtius rearrangement reaction to the isocyanate **16**. All calculations at the B3LYP-D3(BJ)//cc-pVTZ level of theory. 73

S1. Calculations on **7** (isomer 1) 74
- Cartesian Co-ordinates (XYZ format) 74
- Frequencies 75

S2. Calculations on **7** (isomer 2) 77
- Cartesian Co-ordinates (XYZ format) 77
- Frequencies 78

S3. Calculations on **7** (isomer 3) 80
- Cartesian Co-ordinates (XYZ format) 80
- Frequencies 81

S4. Calculations on **7** (TS isomer 1 $\longrightarrow$ isomer 3) 83
- Cartesian Co-ordinates (XYZ format) 83
- Frequencies 84

S5. Calculations on **11s** 86
- Cartesian Co-ordinates (XYZ format) 86
- Frequencies 88

S6. Calculations on TS **11s** $\longrightarrow$ **12s** 90
- Cartesian Co-ordinates (XYZ format) 90
- Frequencies 91

S7. Calculations on **12s** 93
- Cartesian Co-ordinates (XYZ format) 93
- Frequencies 95

S8. Calculations on TS **7** (isomer 1) $\longrightarrow$ **12s** 97

Cartesian Co-ordinates (XYZ format) 97
Frequencies 98

S9. Calculations on **7** (isomer 1; triplet) 100
Cartesian Co-ordinates (XYZ format) 100
Frequencies 101

S10. Calculations on TS **7** (isomer 1; triplet) $\longrightarrow$ **11t** 103
Cartesian Co-ordinates (XYZ format) 103
Frequencies 104

S11. Calculations on **11t** 106
Cartesian Co-ordinates (XYZ format) 106
Frequencies 107

S12. Calculations on TS **11t** $\longrightarrow$ **12t** 109
Cartesian Co-ordinates (XYZ format) 109
Frequencies 110

S13. Calculations on **12t** 112
Cartesian Co-ordinates (XYZ format) 112
Frequencies 113

S14. Calculations on **8** (Isomer 1) 115
Cartesian Co-ordinates (XYZ format) 115
Frequencies 116

S15. Calculations on **8** (Isomer 2) 118
Cartesian Co-ordinates (XYZ format) 118
Frequencies 120

S16. Calculations on **8** (TS isomer 1 $\longrightarrow$ isomer 2) 122
Cartesian Co-ordinates (XYZ format) 122
Frequencies 124

S17. Calculations on **13s** 126
Cartesian Co-ordinates (XYZ format) 126
Frequencies 128

S18. Calculations on TS **13s** $\longrightarrow$ **14s** 130
Cartesian Co-ordinates (XYZ format) 130
Frequencies 131

S19. Calculations on **14s** 133
Cartesian Co-ordinates (XYZ format) 133
Frequencies 134

S20. Calculations on TS **8** (Isomer 1) $\longrightarrow$ **14s** 136
Cartesian Co-ordinates (XYZ format) 136
Frequencies 137

S21. Calculations on **8** (isomer 1; Triplet) 139
Cartesian Co-ordinates (XYZ format) 139
Frequencies 140

S22. Calculations on TS **8** (isomer 1; Triplet) $\longrightarrow$ **13t** 142
Cartesian Co-ordinates (XYZ format) 142
Frequencies 143

S23. Calculations on **13t** 145
Cartesian Co-ordinates (XYZ format) 145
Frequencies 146

S24. Calculations on TS **13t** $\longrightarrow$ **14t** 148
Cartesian Co-ordinates (XYZ format) 148
Frequencies 149

S25. Calculations on **14t** 151
Cartesian Co-ordinates (XYZ format) 151
Frequencies 152

S26. Calculations on $\mathbf{CH_3}$ 154
Cartesian Co-ordinates (XYZ format) 154
Frequencies 155

S27. Calculations on $\mathbf{14s - CH_3}$ 156
Cartesian Co-ordinates (XYZ format) 156
Frequencies 157

S28. Calculations on TS **14s** $\longrightarrow$ **A** 159
Cartesian Co-ordinates (XYZ format) 159
Frequencies 160

S29. Calculations on **A** 162
Cartesian Co-ordinates (XYZ format) 162
Frequencies 163

S30. Calculations on $\mathbf{A'}$ 164
Cartesian Co-ordinates (XYZ format) 164
Frequencies 165

S31. Calculations on TS $\mathbf{A'}$ $\longrightarrow$ **B** (intermediate) 166
Cartesian Co-ordinates (XYZ format) 166
Frequencies 167

S32. Calculations on **B** (intermediate) 168
Cartesian Co-ordinates (XYZ format) 168
Frequencies 169

S33. Calculations on **B** (final) 170
Cartesian Co-ordinates (XYZ format) 170

Frequencies 171

S34. Calculations on TS $\mathbf{A'} \longrightarrow \mathbf{C}$ 172
Cartesian Co-ordinates (XYZ format) 172
Frequencies 173

S35. Calculations on **C** 174
Cartesian Co-ordinates (XYZ format) 174
Frequencies 175

S36. Calculations on **9** (isomer 1) 176
Cartesian Co-ordinates (XYZ format) 176
Frequencies 178

S37. Calculations on **9** (isomer 2) 180
Cartesian Co-ordinates (XYZ format) 180
Frequencies 182

S38. Calculations on TS **9** (isomer 1) $\longrightarrow$ **9** (isomer 2) 184
Cartesian Co-ordinates (XYZ format) 184
Frequencies 186

S39. Calculations on **15s** 188
Cartesian Co-ordinates (XYZ format) 188
Frequencies 190

S40. Calculations on **15s** $\longrightarrow$ **16s** 192
Cartesian Co-ordinates (XYZ format) 192
Frequencies 194

S41. Calculations on TS **9** $\longrightarrow$ **16s** 196
Cartesian Co-ordinates (XYZ format) 196
Frequencies 198

S42. Calculations on **16s** 200
Cartesian Co-ordinates (XYZ format) 200
Frequencies 202

S43. Calculations on **9** (isomer 1; Triplet) 204
Cartesian Co-ordinates (XYZ format) 204
Frequencies 206

S44. Calculations on TS **9** (isomer 1; triplet) $\longrightarrow$ **15t** 208
Cartesian Co-ordinates (XYZ format) 208
Frequencies 210

S45. Calculations on **15t** 212
Cartesian Co-ordinates (XYZ format) 212
Frequencies 214

S46. Calculations on TS **15t** $\longrightarrow$ **16t** 216

Cartesian Co-ordinates (XYZ format) 216
Frequencies 218

S47. Calculations on **16t** 220
Cartesian Co-ordinates (XYZ format) 220
Frequencies 222

S48. Calculations on **10** (isomer 1) 224
Cartesian Co-ordinates (XYZ format) 224
Frequencies 226

S49. Calculations on **10** (isomer 2) 228
Cartesian Co-ordinates (XYZ format) 228
Frequencies 230

S50. Calculations on TS **10** (isomer 1) $\longrightarrow$ **10** (isomer 2) 232
Cartesian Co-ordinates (XYZ format) 232
Frequencies 234

S51. Calculations on **17s** 236
Cartesian Co-ordinates (XYZ format) 236
Frequencies 238

S52. Calculations on TS **17s** $\longrightarrow$ **18s** 240
Cartesian Co-ordinates (XYZ format) 240
Frequencies 242

S53. Calculations on TS **10** (isomer 1) $\longrightarrow$ **18s** 244
Cartesian Co-ordinates (XYZ format) 244
Frequencies 246

S54. Calculations on **18s** 248
Cartesian Co-ordinates (XYZ format) 248
Frequencies 250

S55. Calculations on **10** (isomer 1; Triplet) 252
Cartesian Co-ordinates (XYZ format) 252
Frequencies 254

S56. Calculations on TS **10** (isomer 1; Triplet) $\longrightarrow$ **17t** 256
Cartesian Co-ordinates (XYZ format) 256
Frequencies 258

S57. Calculations on **17t** 260
Cartesian Co-ordinates (XYZ format) 260
Frequencies 262

S58. Calculations on TS **17t** $\longrightarrow$ **18t** 264
Cartesian Co-ordinates (XYZ format) 264
Frequencies 266

S59. Calculations on **18t** 268
Cartesian Co-ordinates (XYZ format) 268
Frequencies 270

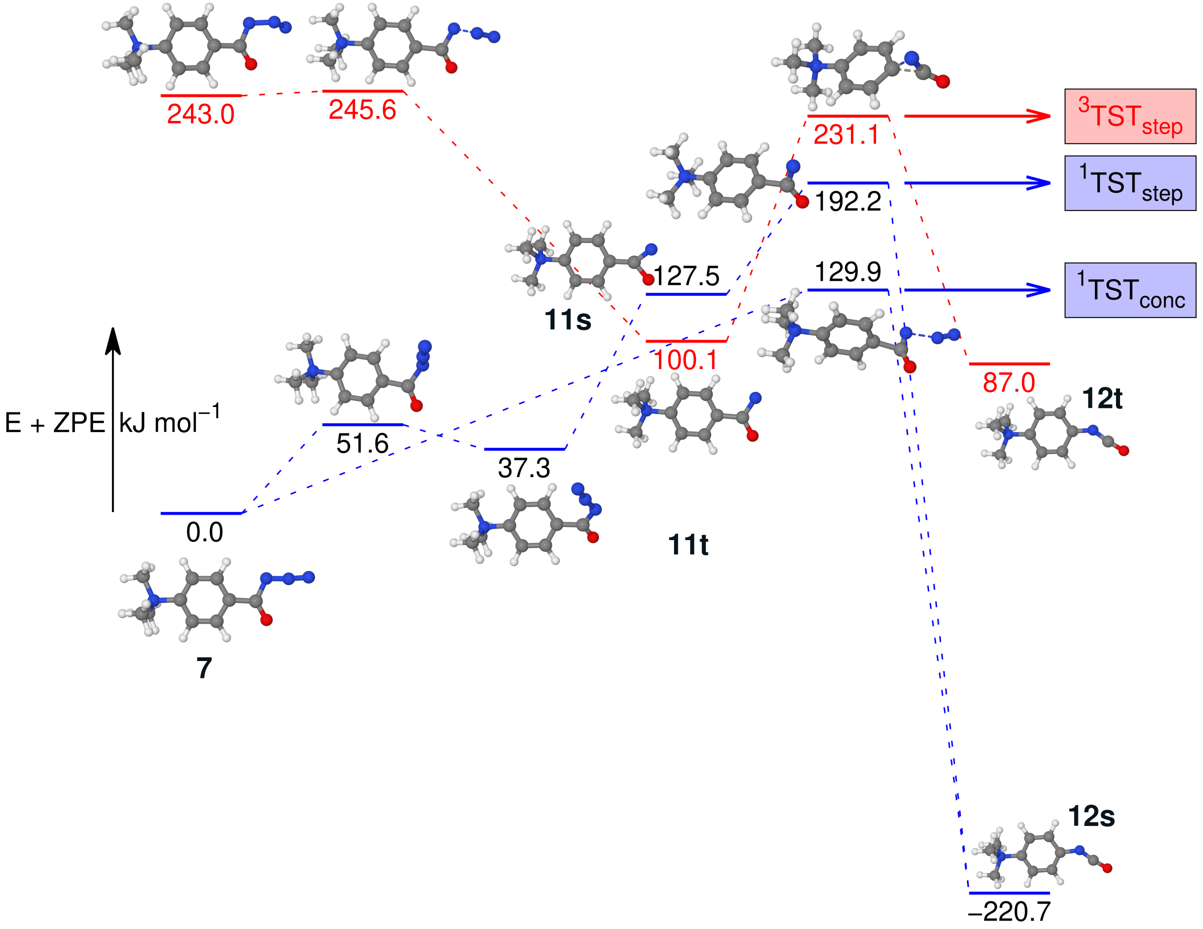

FIG. **S37**. Potential energy surface (PES) of the N_2-loss reaction of the aromatic carbonyl azide **7** via the respective nitrenes **11s**/**11t** in either a stepwise or a concerted Curtius rearrangement reaction to the isocyanate **12**. All calculations at B3LYP-D3(BJ)//cc-pVTZ level of theory.

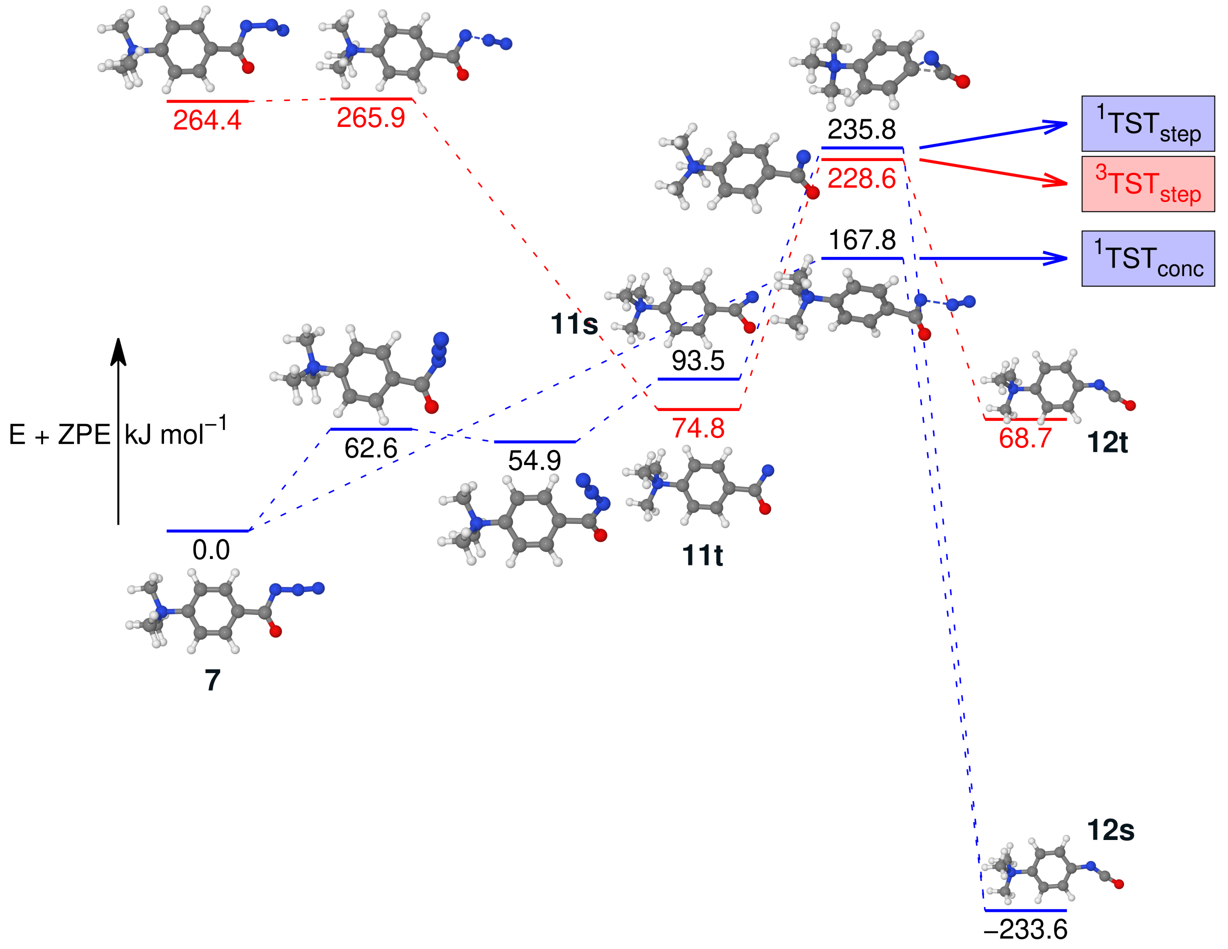

FIG. **S38**. Potential energy surface (PES) of the N_2-loss reaction of the aromatic carbonyl azide **7** via the respective nitrenes **11s**/**11t** in either a stepwise or a concerted Curtius rearrangement reaction to the isocyanate **12**. Optimization at B3LYP-D3(BJ)//cc-pVTZ level of theory with energies at CASPT2//cc-pVTZ.

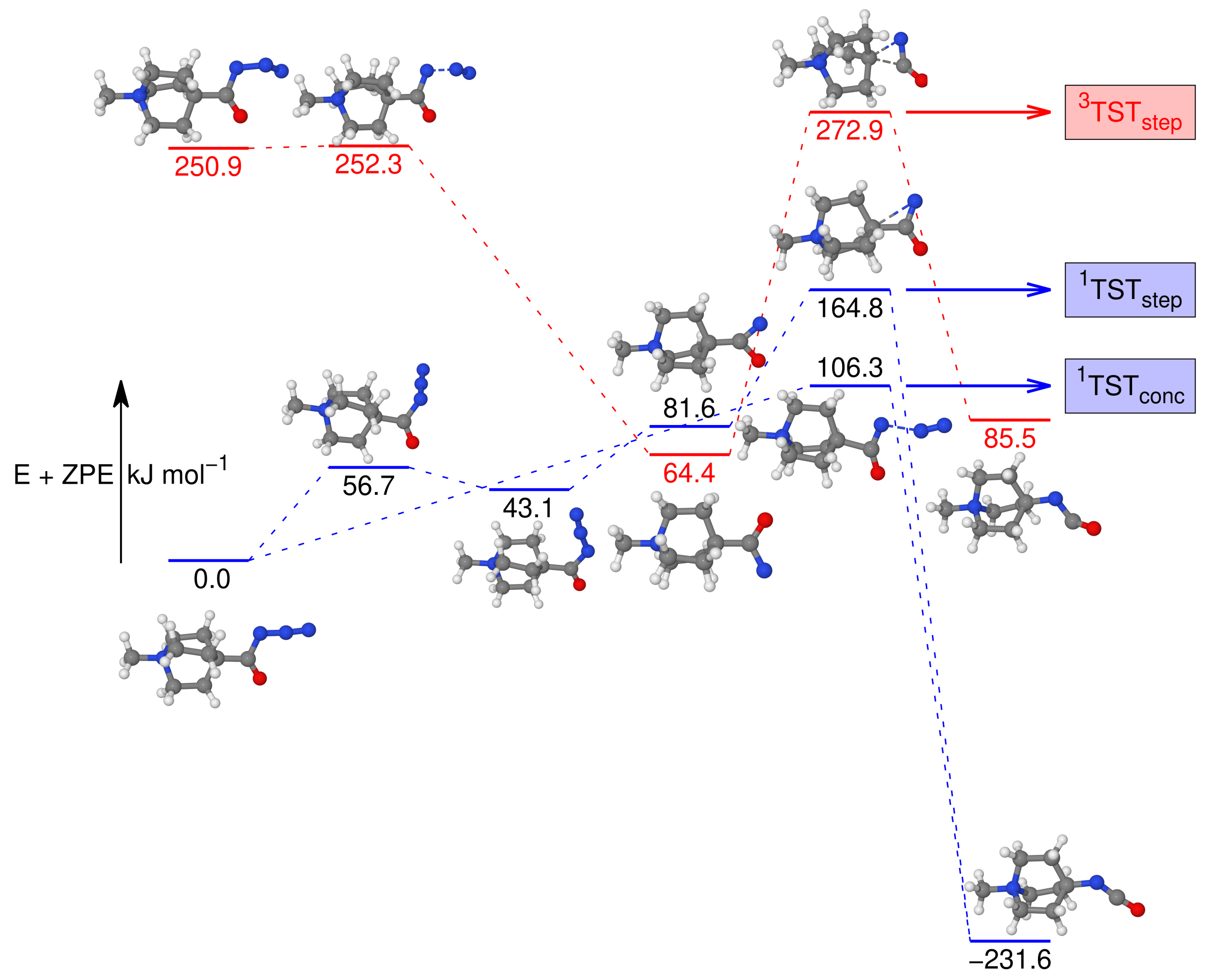

FIG. **S39**. Potential energy surface (PES) of the N_2-loss reaction of the methyl quinuclidinium carbonyl azide **8** via the respective nitrenes **13s**/**14t** in either a stepwise or a concerted Curtius rearrangement reaction to the isocyanate **14**. All optimizations at B3LYP-D3(BJ)//cc-pVTZ level of theory. Singlet energies: CCSD(T)-F12b//cc-pVDZ-F12. Triplet energies: CASPT2//cc-pVTZ

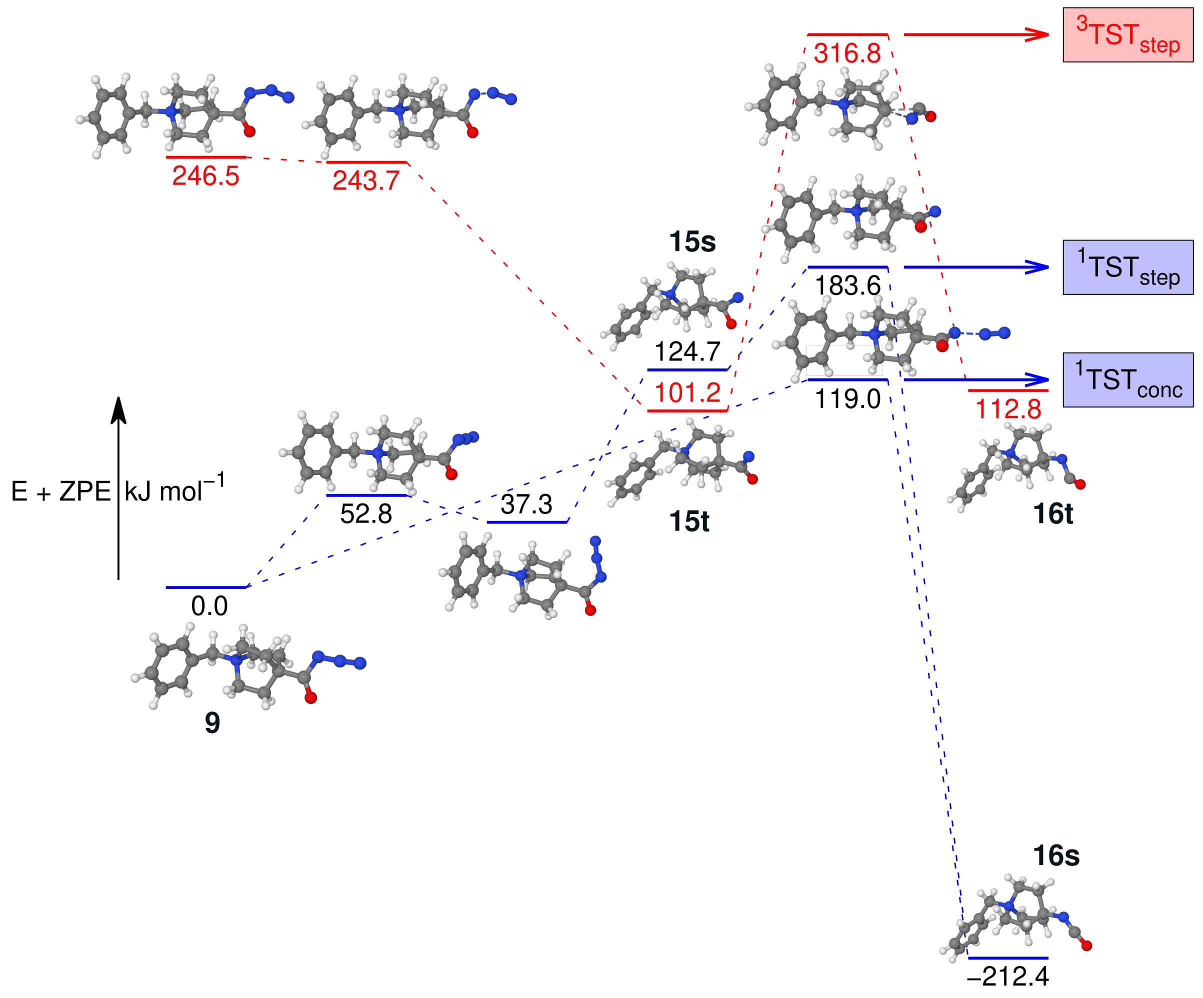

FIG. **S40**. Potential energy surface (PES) of the N_2-loss reaction of the benzyl quinuclidinium carbonyl azide **9** via the respective nitrenes **15s**/**15t** in either a stepwise or a concerted Curtius rearrangement reaction to the isocyanate **16**. All calculations at the B3LYP-D3(BJ)//cc-pVTZ level of theory.

S1. CALCULATIONS ON 7 (ISOMER 1)

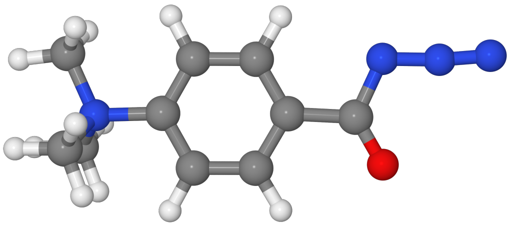

Route	:	# opt freq b3lyp/cc-pvtz empiricaldispersion=gd3bj pop=regular geom=connectivity int=ultrafine
SMILES	:	C[N](C)(C)c1ccc(cc1)C(=O)N=[N+]=[N-]
Formula	:	$C_{10}H_{13}N_4O^+$
Charge	:	1
Multiplicity	:	1
Energy	:	-683.11746068 a.u.
Gibbs Energy	:	-682.92955700 a.u.
CASPT2 Energy	:	-681.47517678 a.u.
CCSD(T)-F12b Energy	:	-681.83714673 a.u.

Cartesian Co-ordinates (XYZ format)

```
28

C  -3.73331499 -0.53415698  1.23425400
H  -3.31400299 -0.04265100  2.10620689
H  -4.81495905 -0.43684301  1.22273505
H  -3.45350599 -1.58032894  1.22893798
C  -3.66754699  1.55943501  0.00011800
H  -3.31144500  2.06205893 -0.89232898
H  -4.75232887  1.53484297  0.00011800
H  -3.31144190  2.06193209  0.89263600
C  -3.73333406 -0.53398699 -1.23430598
H  -3.45352793 -1.58015895 -1.22913694
H  -4.81497812 -0.43667200 -1.22275805
H  -3.31403399 -0.04236100 -2.10619712
C  -0.88234699  1.16797400  0.00006300
C  -1.11322904 -1.23651898 -0.00008700
C   0.50117898  1.02506804  0.00004400
H  -1.29477000  2.16237497  0.00012900
C   0.26317701 -1.36774194 -0.00010500
H  -1.72085595 -2.12862706 -0.00013800
C   1.07937300 -0.23776300 -0.00004100
H   1.12830102  1.90322495  0.00009400
H   0.72469699 -2.34412003 -0.00017000
C   2.55882597 -0.45266500 -0.00006500
O   3.05843711 -1.55073202 -0.00012200
N   4.51400280  0.62598902  0.00002100
N   5.62922001  0.60540003  0.00002200
N   3.27176404  0.76361400  0.00002200
N  -3.18045712  0.13805200  0.00001600
C  -1.67874002  0.03451600 -0.00000200
```

Frequencies

Mode	IR frequency	IR intensity	Raman intensity
1	36.02740000	1.78200000	0.00000000
2	57.61270000	0.00070000	0.00000000
3	68.27000000	0.21890000	0.00000000
4	94.53910000	0.64250000	0.00000000
5	113.28130000	0.00340000	0.00000000
6	192.60330000	1.67150000	0.00000000
7	203.44970000	0.44400000	0.00000000
8	231.27390000	2.01120000	0.00000000
9	240.70140000	0.09700000	0.00000000
10	271.86730000	0.98370000	0.00000000
11	301.99400000	5.61390000	0.00000000
12	346.42130000	0.12870000	0.00000000
13	356.66000000	0.08380000	0.00000000
14	372.09060000	1.18210000	0.00000000
15	422.15270000	0.00090000	0.00000000
16	430.36660000	3.19080000	0.00000000
17	430.71220000	0.05380000	0.00000000
18	466.38930000	0.76780000	0.00000000
19	501.78600000	3.47250000	0.00000000
20	531.23430000	1.11940000	0.00000000
21	553.04960000	18.32600000	0.00000000
22	593.63950000	0.71560000	0.00000000
23	607.53080000	1.25460000	0.00000000
24	650.13650000	1.61910000	0.00000000
25	700.46870000	32.84610000	0.00000000
26	752.75800000	15.73180000	0.00000000
27	779.83650000	10.11580000	0.00000000
28	843.86520000	22.52140000	0.00000000
29	844.11990000	0.17530000	0.00000000
30	879.33100000	10.71510000	0.00000000
31	886.03490000	40.49700000	0.00000000
32	946.35380000	27.45440000	0.00000000
33	961.71790000	15.01670000	0.00000000
34	1003.24550000	0.12080000	0.00000000
35	1022.16460000	0.11460000	0.00000000
36	1025.51200000	120.97990000	0.00000000
37	1048.46260000	21.10120000	0.00000000
38	1077.57370000	0.02140000	0.00000000
39	1128.83780000	18.56570000	0.00000000
40	1132.72670000	0.62750000	0.00000000
41	1140.60900000	0.51310000	0.00000000
42	1162.39180000	15.53170000	0.00000000
43	1214.60150000	8.71990000	0.00000000
44	1245.94090000	55.50630000	0.00000000
45	1258.15990000	1.62040000	0.00000000
46	1258.26490000	2.62100000	0.00000000
47	1288.38230000	664.21490000	0.00000000
48	1299.08880000	223.56220000	0.00000000
49	1349.28910000	2.64960000	0.00000000
50	1352.61310000	7.18490000	0.00000000
51	1447.79050000	7.24550000	0.00000000
52	1452.29360000	4.82270000	0.00000000
53	1452.96550000	19.48640000	0.00000000
54	1480.11110000	0.00160000	0.00000000
55	1489.46600000	0.38060000	0.00000000
56	1493.89320000	2.18160000	0.00000000
57	1496.05620000	1.27260000	0.00000000
58	1507.57820000	26.27610000	0.00000000
59	1513.68440000	24.30720000	0.00000000
60	1531.22010000	48.60420000	0.00000000

61	1542.93060000	15.99750000	0.00000000
62	1634.20120000	2.70450000	0.00000000
63	1647.51750000	18.24980000	0.00000000
64	1757.69600000	194.09520000	0.00000000
65	2303.76820000	519.84540000	0.00000000
66	3080.60660000	0.27940000	0.00000000
67	3081.48030000	1.00580000	0.00000000
68	3088.29280000	0.82770000	0.00000000
69	3168.32940000	0.00580000	0.00000000
70	3168.76630000	0.51990000	0.00000000
71	3175.95960000	4.98760000	0.00000000
72	3183.31330000	0.12960000	0.00000000
73	3189.08960000	0.01840000	0.00000000
74	3189.36640000	2.01740000	0.00000000
75	3201.98880000	0.96810000	0.00000000
76	3214.67310000	7.80670000	0.00000000
77	3216.43980000	1.33730000	0.00000000
78	3238.79270000	1.60340000	0.00000000

S2. CALCULATIONS ON 7 (ISOMER 2)

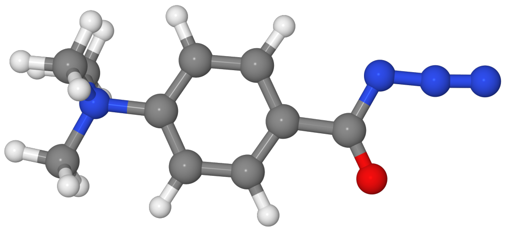

Route	:	# opt freq b3lyp/cc-pvtz empiricaldispersion=gd3bj pop=regular geom=connectivity int=ultrafine
SMILES	:	C[N](C)(C)c1ccc(cc1)C(=O)N=[N+]=[N-]
Formula	:	$C_{10}H_{13}N_4O^+$
Charge	:	1
Multiplicity	:	1
Energy	:	-683.11739044 a.u.
Gibbs Energy	:	-682.92949500 a.u.
CCSD(T)-F12b Energy	:	-681.83707211 a.u.

Cartesian Co-ordinates (XYZ format)

```
28

C  -3.58250499 -1.00600696 -1.15512502
H  -3.12902999 -1.98175502 -1.03230906
H  -4.66500902 -1.09320104 -1.14216101
H  -3.24598193 -0.55476600 -2.08294010
C  -3.60239196 -0.71965802  1.29662097
H  -3.27965808 -0.06616900  2.10067296
H  -4.68471909 -0.80930603  1.28688705
H  -3.14880991 -1.69661796  1.40902901
C  -3.88363099  1.19709098 -0.17236800
H  -3.61070299  1.64595902 -1.12101495
H  -4.94836712  0.98868698 -0.15693200
H  -3.62554789  1.85294604  0.65171403
C  -0.89005601 -1.10274303  0.12072000
C  -1.07374299  1.28942001 -0.15952501
C   0.49113700 -0.99795699  0.12012600
H  -1.33610797 -2.07945490  0.23078400
C   0.31137699  1.38353503 -0.15885200
H  -1.65003395  2.19200802 -0.26960599
C   1.09955394  0.24744800 -0.01984000
H   1.09818494 -1.88347602  0.22838600
H   0.79467702  2.34333897 -0.26663199
C   2.58382893  0.42699799 -0.02816700
O   3.11071110  1.50528300 -0.14901200
N   4.51164484 -0.69072300  0.11804900
N   5.62698221 -0.69737500  0.12834699
N   3.26642108 -0.79805601  0.11997100
N  -3.16194391 -0.11145300 -0.01358700
C  -1.66447699  0.04295900 -0.01927600
```

Frequencies

Mode	IR frequency	IR intensity	Raman intensity
1	37.82850000	1.61860000	0.00000000
2	51.29580000	0.07180000	0.00000000
3	74.06850000	0.27940000	0.00000000
4	94.22250000	0.59600000	0.00000000
5	112.22720000	0.00510000	0.00000000
6	190.30940000	0.74820000	0.00000000
7	204.12510000	0.43810000	0.00000000
8	240.76260000	0.08890000	0.00000000
9	244.31740000	2.91550000	0.00000000
10	269.72510000	4.75950000	0.00000000
11	285.23410000	0.01220000	0.00000000
12	355.73040000	0.11110000	0.00000000
13	357.51900000	2.82670000	0.00000000
14	372.22630000	1.13010000	0.00000000
15	421.92700000	0.02850000	0.00000000
16	427.13160000	1.44870000	0.00000000
17	430.69000000	0.08610000	0.00000000
18	472.26530000	1.64380000	0.00000000
19	498.59550000	4.29080000	0.00000000
20	530.40180000	1.17420000	0.00000000
21	552.99630000	18.21460000	0.00000000
22	593.70790000	0.74150000	0.00000000
23	607.17650000	1.26740000	0.00000000
24	650.44210000	1.38780000	0.00000000
25	700.35210000	32.93400000	0.00000000
26	752.24230000	16.33950000	0.00000000
27	779.49230000	10.06100000	0.00000000
28	843.87090000	23.18300000	0.00000000
29	844.10260000	0.01780000	0.00000000
30	878.16990000	11.10540000	0.00000000
31	885.73840000	41.16920000	0.00000000
32	946.47820000	27.05160000	0.00000000
33	961.31070000	13.62540000	0.00000000
34	1007.99480000	0.11940000	0.00000000
35	1017.53970000	0.24440000	0.00000000
36	1025.52030000	118.73180000	0.00000000
37	1049.69960000	23.17800000	0.00000000
38	1077.56320000	0.02450000	0.00000000
39	1129.20110000	16.08570000	0.00000000
40	1132.47190000	0.51320000	0.00000000
41	1140.69930000	0.50410000	0.00000000
42	1163.16850000	8.28190000	0.00000000
43	1213.46030000	12.62610000	0.00000000
44	1244.62850000	55.29090000	0.00000000
45	1258.24500000	1.64270000	0.00000000
46	1258.60160000	5.87230000	0.00000000
47	1287.78680000	669.51780000	0.00000000
48	1298.95240000	214.59870000	0.00000000
49	1349.00690000	4.08250000	0.00000000
50	1352.08990000	11.83330000	0.00000000
51	1447.60170000	3.74980000	0.00000000
52	1452.16520000	4.80360000	0.00000000
53	1452.78180000	17.99000000	0.00000000
54	1480.20050000	0.00470000	0.00000000
55	1489.41360000	0.34560000	0.00000000
56	1493.72060000	2.04240000	0.00000000
57	1495.60250000	0.41980000	0.00000000
58	1507.90030000	28.43910000	0.00000000
59	1513.68180000	24.33930000	0.00000000
60	1531.03940000	49.78200000	0.00000000

61	1542.79630000	16.27940000	0.00000000
62	1633.72700000	0.95710000	0.00000000
63	1647.12190000	19.36460000	0.00000000
64	1758.64720000	194.15690000	0.00000000
65	2303.69940000	518.99640000	0.00000000
66	3080.51260000	0.27850000	0.00000000
67	3081.43950000	1.00070000	0.00000000
68	3088.23560000	0.81290000	0.00000000
69	3168.35690000	0.01160000	0.00000000
70	3168.85590000	0.59420000	0.00000000
71	3176.03740000	4.89300000	0.00000000
72	3183.40260000	0.10490000	0.00000000
73	3188.44390000	0.01770000	0.00000000
74	3188.74970000	2.63380000	0.00000000
75	3202.38780000	1.56050000	0.00000000
76	3210.05040000	5.05890000	0.00000000
77	3220.61220000	2.78420000	0.00000000
78	3238.61940000	1.84930000	0.00000000

S3. CALCULATIONS ON 7 (ISOMER 3)

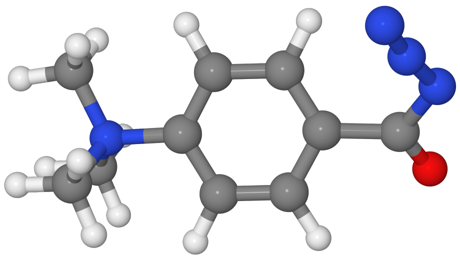

Route	:	# opt freq b3lyp/cc-pvtz empiricaldispersion=gd3bj pop=regular geom=connectivity int=ultrafine
SMILES	:	C[N](C)(C)c1ccc(cc1)C(=O)N=[N+]=[N-]
Formula	:	$C_{10}H_{13}N_4O^+$
Charge	:	1
Multiplicity	:	1
Energy	:	-683.10299776 a.u.
Gibbs Energy	:	-682.91545200 a.u.
CASPT2 Energy	:	-681.45398241 a.u.
CCSD(T)-F12b Energy	:	-681.82181402 a.u.

Cartesian Co-ordinates (XYZ format)

```
28

C  -3.33202100 -0.32641801 -1.46679497
H  -2.80261207 -1.17828000 -1.88129401
H  -4.40486193 -0.49309799 -1.49218595
H  -3.08201599  0.57042700 -2.02001595
C  -3.35539603 -1.38516402  0.72204298
H  -3.09832406 -1.28055704  1.77035499
H  -4.43237019 -1.46139705  0.61444801
H  -2.88057303 -2.26127911  0.29416800
C  -3.62019491  1.02822006  0.57745802
H  -3.36974812  1.91955101  0.01556400
H  -4.69002819  0.84691900  0.53230602
H  -3.29281211  1.13428199  1.60667205
C  -0.60283899 -0.81052601  0.74461102
C  -0.89156401  1.16034901 -0.61758602
C   0.76836300 -0.57496899  0.77552801
H  -0.98679799 -1.66374898  1.27697897
C   0.46954700  1.39620101 -0.56431001
H  -1.51663005  1.84980798 -1.16437602
C   1.31271994  0.52241600  0.12165900
H   1.39301801 -1.24832702  1.34326005
H   0.89127499  2.26674509 -1.04441500
C   2.77166200  0.88208097  0.18900900
O   3.11799908  2.02494407  0.30117700
N   3.42249703 -1.27134597 -0.26541200
N   3.27214408 -2.32251406 -0.62904900
N   3.74285889 -0.14629500  0.14251000
C  -1.42203903  0.05533800  0.04091100
N  -2.90932012 -0.16050200 -0.02612400
```

Frequencies

Mode	IR frequency	IR intensity	Raman intensity
1	32.79470000	0.46570000	0.00000000
2	55.07030000	0.12970000	0.00000000
3	67.38720000	0.25790000	0.00000000
4	102.91650000	1.42340000	0.00000000
5	120.81690000	0.22710000	0.00000000
6	161.64510000	1.85500000	0.00000000
7	201.71700000	2.31930000	0.00000000
8	236.22520000	1.27800000	0.00000000
9	258.98370000	1.04330000	0.00000000
10	271.83900000	0.77220000	0.00000000
11	298.55300000	3.55260000	0.00000000
12	348.52170000	1.46460000	0.00000000
13	356.22780000	0.39460000	0.00000000
14	359.93540000	2.07410000	0.00000000
15	417.56250000	0.75160000	0.00000000
16	422.15070000	0.30650000	0.00000000
17	436.01910000	1.59610000	0.00000000
18	473.55280000	1.72430000	0.00000000
19	495.42140000	4.80490000	0.00000000
20	566.93590000	7.69140000	0.00000000
21	572.78750000	3.52440000	0.00000000
22	583.23280000	9.39780000	0.00000000
23	639.34760000	3.91220000	0.00000000
24	652.31980000	3.78280000	0.00000000
25	681.36330000	29.46170000	0.00000000
26	706.06480000	21.91540000	0.00000000
27	776.15050000	10.40140000	0.00000000
28	781.67190000	2.87290000	0.00000000
29	842.76810000	0.23090000	0.00000000
30	846.17000000	30.19290000	0.00000000
31	873.72250000	9.54500000	0.00000000
32	944.56810000	25.84170000	0.00000000
33	960.97110000	15.76610000	0.00000000
34	989.81910000	1.64350000	0.00000000
35	1009.70570000	0.15060000	0.00000000
36	1026.68040000	126.39510000	0.00000000
37	1051.30090000	44.42160000	0.00000000
38	1077.49620000	0.02540000	0.00000000
39	1130.87980000	20.68010000	0.00000000
40	1134.45790000	1.11820000	0.00000000
41	1141.07460000	0.45230000	0.00000000
42	1169.60770000	5.80050000	0.00000000
43	1226.10360000	32.22700000	0.00000000
44	1239.92720000	45.49770000	0.00000000
45	1257.73670000	1.48950000	0.00000000
46	1257.97980000	1.18570000	0.00000000
47	1296.04920000	6.13890000	0.00000000
48	1323.39410000	404.29180000	0.00000000
49	1344.53850000	3.12940000	0.00000000
50	1357.54420000	3.89350000	0.00000000
51	1446.68870000	8.55910000	0.00000000
52	1452.59220000	17.39110000	0.00000000
53	1452.68410000	4.91840000	0.00000000
54	1480.48930000	0.00220000	0.00000000
55	1489.43980000	0.27060000	0.00000000
56	1494.04780000	2.02690000	0.00000000
57	1495.69340000	1.08140000	0.00000000
58	1508.03170000	26.12260000	0.00000000
59	1514.16300000	24.53690000	0.00000000
60	1531.16810000	47.98350000	0.00000000

61	1544.09480000	18.30720000	0.00000000
62	1632.14550000	3.35790000	0.00000000
63	1646.39630000	13.24050000	0.00000000
64	1786.47540000	388.04690000	0.00000000
65	2266.75710000	372.53750000	0.00000000
66	3080.88330000	0.20990000	0.00000000
67	3081.58420000	0.82290000	0.00000000
68	3088.19110000	0.53120000	0.00000000
69	3168.91280000	0.03790000	0.00000000
70	3169.34140000	0.32540000	0.00000000
71	3176.40550000	3.89030000	0.00000000
72	3182.70510000	0.11220000	0.00000000
73	3188.50370000	0.22340000	0.00000000
74	3189.43740000	1.41530000	0.00000000
75	3202.30590000	0.79020000	0.00000000
76	3204.11520000	0.28430000	0.00000000
77	3214.81820000	4.63160000	0.00000000
78	3238.94940000	0.68820000	0.00000000

S4. CALCULATIONS ON 7 (TS ISOMER 1 ⟶ ISOMER 3)

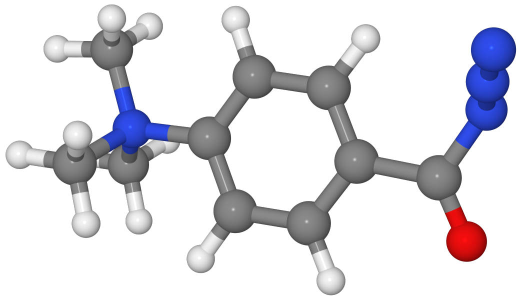

Route	:	# opt=(calcfc,qst3) freq b3lyp/cc-pvtz empiricaldispersion=gd3bj pop=regular geom=connectivity int=ultrafine
SMILES	:	C[N](C)(C)c1ccc(cc1)C(=O)N=[N+]=[N-]
Formula	:	$C_{10}H_{13}N_4O^+$
Charge	:	1
Multiplicity	:	1
Energy	:	-683.09730712 a.u.
Gibbs Energy	:	-682.90914500 a.u.
CASPT2 Energy	:	-681.45082832 a.u.
CCSD(T)-F12b Energy	:	-681.81623713 a.u.

Cartesian Co-ordinates (XYZ format)

```
28

C  -3.33943510  0.20295000  1.45725095
H  -2.80366397  0.99680197  1.96766901
H  -4.40778303  0.39788899  1.46161497
H  -3.13294005 -0.74837101  1.93177700
C  -3.25749302  1.46075499 -0.62248200
H  -2.96229291  1.44802499 -1.66588402
H  -4.33578682  1.55501401 -0.54711199
H  -2.77975106  2.28010106 -0.09668100
C  -3.58678293 -0.94718999 -0.71414798
H  -3.38026309 -1.89356196 -0.22982299
H  -4.65255022 -0.74005002 -0.68928403
H  -3.22439408 -0.96662998 -1.73691797
C  -0.52022898  0.81770802 -0.59832799
C  -0.90973002 -1.26940596  0.55422902
C   0.84394097  0.54375601 -0.60975999
H  -0.86311501  1.73240900 -1.05057502
C   0.44626200 -1.53458297  0.53306299
H  -1.57311797 -1.98758602  1.01156795
C   1.33399904 -0.62677503 -0.04638800
H   1.52022099  1.24252796 -1.07810295
H   0.83513498 -2.44634509  0.96184802
C   2.79183602 -0.96874702 -0.04026600
O   3.19868588 -2.04508305  0.28369501
N   4.05045700  0.93132299  0.21830900
N   4.45909023  1.77266598  0.84286499
N   3.66830897  0.05327700 -0.55422598
C  -1.38603699 -0.09122600 -0.01424600
N  -2.86910009  0.15975600  0.02226700
```

Frequencies

Mode	IR frequency	IR intensity	Raman intensity
1	-97.06010000	1.33880000	0.00000000
2	29.96600000	0.32530000	0.00000000
3	59.05180000	0.05730000	0.00000000
4	66.35280000	0.01930000	0.00000000
5	138.35800000	1.16610000	0.00000000
6	153.12450000	1.11410000	0.00000000
7	200.33680000	0.02900000	0.00000000
8	236.29400000	0.00300000	0.00000000
9	252.66860000	1.85390000	0.00000000
10	270.99960000	0.80420000	0.00000000
11	300.35880000	3.59690000	0.00000000
12	350.50520000	0.92210000	0.00000000
13	354.89740000	0.14990000	0.00000000
14	367.97840000	0.91280000	0.00000000
15	421.53520000	0.02140000	0.00000000
16	427.35740000	0.18750000	0.00000000
17	441.20040000	1.64300000	0.00000000
18	465.03550000	1.57320000	0.00000000
19	496.72140000	2.29460000	0.00000000
20	546.32570000	9.78590000	0.00000000
21	560.75020000	13.75480000	0.00000000
22	567.93870000	6.91310000	0.00000000
23	605.69020000	1.02340000	0.00000000
24	649.65720000	1.90920000	0.00000000
25	665.78010000	9.69510000	0.00000000
26	730.17330000	25.78630000	0.00000000
27	773.02250000	11.92390000	0.00000000
28	809.75930000	28.60880000	0.00000000
29	845.12680000	25.44610000	0.00000000
30	845.24890000	2.39080000	0.00000000
31	887.28630000	3.61410000	0.00000000
32	945.55870000	26.22300000	0.00000000
33	960.23950000	14.30740000	0.00000000
34	987.83310000	269.40580000	0.00000000
35	1000.78280000	4.43260000	0.00000000
36	1021.68110000	1.81430000	0.00000000
37	1039.91010000	2.48040000	0.00000000
38	1077.39220000	0.02430000	0.00000000
39	1128.54160000	20.63210000	0.00000000
40	1133.30320000	0.03580000	0.00000000
41	1140.45370000	0.49290000	0.00000000
42	1164.14450000	6.08580000	0.00000000
43	1210.02480000	97.64470000	0.00000000
44	1240.53980000	73.18750000	0.00000000
45	1257.53980000	1.56630000	0.00000000
46	1257.84920000	0.90740000	0.00000000
47	1296.18060000	0.22650000	0.00000000
48	1348.16980000	20.39440000	0.00000000
49	1350.64810000	22.45080000	0.00000000
50	1358.30050000	317.76800000	0.00000000
51	1446.81370000	9.67060000	0.00000000
52	1452.34540000	5.25470000	0.00000000
53	1452.67480000	17.85520000	0.00000000
54	1480.22590000	0.00240000	0.00000000
55	1489.24560000	0.35260000	0.00000000
56	1493.87580000	1.81150000	0.00000000
57	1495.52770000	1.42180000	0.00000000
58	1507.78580000	26.15710000	0.00000000
59	1513.90100000	24.61080000	0.00000000
60	1530.86760000	47.73590000	0.00000000

61	1540.40750000	16.14870000	0.00000000
62	1634.09130000	7.75890000	0.00000000
63	1645.35180000	23.29750000	0.00000000
64	1800.05360000	284.00190000	0.00000000
65	2286.39720000	591.28350000	0.00000000
66	3081.12840000	0.19730000	0.00000000
67	3081.65230000	0.74060000	0.00000000
68	3088.28320000	0.52110000	0.00000000
69	3169.08190000	0.04390000	0.00000000
70	3169.32500000	0.27500000	0.00000000
71	3176.39320000	3.93330000	0.00000000
72	3183.01530000	0.10190000	0.00000000
73	3189.00300000	0.15280000	0.00000000
74	3189.54020000	1.55760000	0.00000000
75	3202.43710000	0.78770000	0.00000000
76	3212.62580000	0.73060000	0.00000000
77	3214.73760000	7.33310000	0.00000000
78	3240.09480000	1.25790000	0.00000000

S5. CALCULATIONS ON 11s

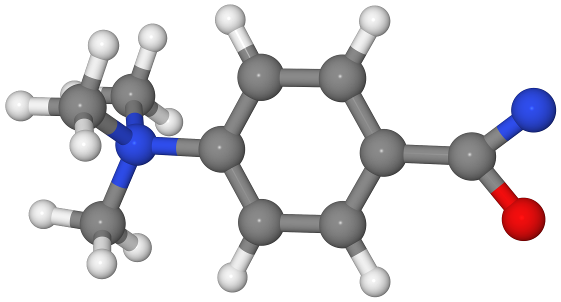

Route	:	# opt freq b3lyp/cc-pvtz empiricaldispersion=gd3bj pop=regular geom=connectivity int=ultrafine
SMILES	:	C[N](C)(C)c1ccc(cc1)[C]2[N]O2
Formula	:	$C_{10}H_{13}N_2O^+$
Charge	:	1
Multiplicity	:	1
Energy	:	-573.49348369 a.u.
Gibbs Energy	:	-573.31478700 a.u.
CASPT2 Energy (incl. N_2 at $R = \infty$)	:	-681.43333919 a.u.
CCSD(T)-F12b Energy	:	-572.39769135 a.u.

Cartesian Co-ordinates (XYZ format)

26

C	3.06652689	-1.40274405	0.00066900
H	2.74758005	-1.92957401	-0.89195400
H	4.14673901	-1.30120504	0.00040200
H	2.74792910	-1.92848206	0.89405900
C	2.98607802	0.69050997	-1.23575604
H	2.63731694	1.71580803	-1.23064899
H	4.07167721	0.66634101	-1.22365403
H	2.60160804	0.17113300	-2.10766912
C	2.98607206	0.69183999	1.23486197
H	2.60170794	0.17332201	2.10733294
H	4.07167292	0.66778499	1.22272599
H	2.63718295	1.71709096	1.22870898
C	0.32416400	1.21127105	-0.00023400
C	0.26295400	-1.20718002	0.00019300
C	-1.05768096	1.25056696	-0.00019400
H	0.86990702	2.14216805	-0.00039700
C	-1.12615895	-1.16231704	0.00022800
H	0.74528402	-2.16942501	0.00034700
C	-1.78360701	0.06049100	0.00003300
H	-1.57186103	2.20102906	-0.00033700
H	-1.69920003	-2.07852292	0.00040700
C	-3.23629403	0.07446600	0.00007500
O	-4.10609102	-0.89769298	0.00027600
N	-4.20647717	0.87030798	-0.00003800
C	0.97641701	-0.01928800	-0.00004600

N 2.48072100 -0.01781000 -0.00006600

Frequencies

Mode	IR frequency	IR intensity	Raman intensity
1	48.31220000	0.02650000	0.00000000
2	66.32780000	0.22840000	0.00000000
3	76.65840000	0.13790000	0.00000000
4	119.35150000	2.45170000	0.00000000
5	182.95030000	2.24810000	0.00000000
6	231.56660000	0.24010000	0.00000000
7	248.56070000	1.96330000	0.00000000
8	268.53130000	1.05840000	0.00000000
9	294.30590000	2.10840000	0.00000000
10	349.57790000	1.84240000	0.00000000
11	352.43530000	2.59310000	0.00000000
12	356.88470000	0.00350000	0.00000000
13	417.23300000	0.02670000	0.00000000
14	422.58580000	0.03490000	0.00000000
15	423.01900000	1.90000000	0.00000000
16	469.00170000	1.53600000	0.00000000
17	520.80160000	0.74510000	0.00000000
18	534.10840000	5.40140000	0.00000000
19	536.23630000	3.86190000	0.00000000
20	620.15630000	44.16550000	0.00000000
21	638.65640000	3.09330000	0.00000000
22	649.22870000	0.19490000	0.00000000
23	753.90170000	0.05070000	0.00000000
24	796.75780000	0.53900000	0.00000000
25	843.38800000	0.07550000	0.00000000
26	846.89190000	35.70300000	0.00000000
27	867.35690000	19.85640000	0.00000000
28	943.79420000	28.82900000	0.00000000
29	958.96380000	16.00340000	0.00000000
30	996.35100000	0.01130000	0.00000000
31	1003.81690000	0.08570000	0.00000000
32	1038.36970000	9.44440000	0.00000000
33	1077.26570000	0.02830000	0.00000000
34	1127.37730000	8.45520000	0.00000000
35	1132.40340000	0.51990000	0.00000000
36	1140.46620000	0.47470000	0.00000000
37	1164.43150000	7.88440000	0.00000000
38	1183.99820000	2.39860000	0.00000000
39	1228.50010000	1.29200000	0.00000000
40	1256.81800000	1.71210000	0.00000000
41	1256.85770000	0.65000000	0.00000000
42	1280.93060000	25.50730000	0.00000000
43	1296.22900000	0.59370000	0.00000000
44	1351.98750000	6.14210000	0.00000000
45	1359.89060000	2.48680000	0.00000000
46	1450.49650000	1.25850000	0.00000000
47	1452.68260000	4.91350000	0.00000000
48	1458.09150000	25.72830000	0.00000000
49	1480.51880000	0.00170000	0.00000000
50	1489.14470000	0.35350000	0.00000000
51	1494.13230000	1.65280000	0.00000000
52	1495.72210000	1.13490000	0.00000000
53	1508.11600000	26.77940000	0.00000000
54	1514.29670000	24.90370000	0.00000000
55	1531.01010000	49.62080000	0.00000000
56	1546.24080000	36.08690000	0.00000000
57	1634.53550000	5.75960000	0.00000000
58	1651.45680000	4.48650000	0.00000000
59	1816.92220000	86.37120000	0.00000000
60	3081.48820000	0.11550000	0.00000000

61	3081.93090000	0.61030000	0.00000000
62	3088.49830000	0.32950000	0.00000000
63	3169.54940000	0.01640000	0.00000000
64	3169.80820000	0.29200000	0.00000000
65	3176.93280000	3.20540000	0.00000000
66	3183.09250000	0.06280000	0.00000000
67	3189.41310000	0.00340000	0.00000000
68	3190.27090000	1.43470000	0.00000000
69	3198.88660000	0.45310000	0.00000000
70	3202.82610000	3.18680000	0.00000000
71	3212.10610000	2.64300000	0.00000000
72	3242.08450000	1.33860000	0.00000000

S6. CALCULATIONS ON TS 11s ⟶ 12s

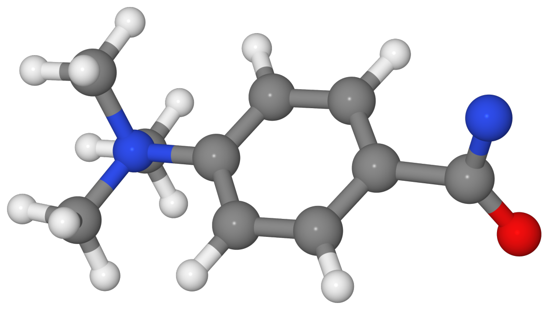

Route	:	# opt=qst2 freq b3lyp/cc-pvtz empiricaldispersion=gd3bj pop=regular geom=connectivity int=ultrafine
SMILES	:	C[N](C)(C)c1ccc(cc1)C(=O)[N]
Formula	:	$C_{10}H_{13}N_2O^+$
Charge	:	1
Multiplicity	:	1
Energy	:	-573.46683707 a.u.
Gibbs Energy	:	-573.29063000 a.u.
CASPT2 Energy (incl. N_2 at $R = \infty$)	:	-681.37714329 a.u.
CCSD(T)-F12b Energy	:	-572.36165384 a.u.

Cartesian Co-ordinates (XYZ format)

26

C	3.00527596	1.41804802	-0.11904900
H	2.69203401	2.00454307	0.73768997
H	4.08648920	1.32852995	-0.12650900
H	2.66894007	1.87389505	-1.04382098
C	2.96776700	-0.58156401	1.26676595
H	2.63356805	-1.60906994	1.34023499
H	4.05262089	-0.54399002	1.23720002
H	2.58794808	-0.00554200	2.10452890
C	2.93443203	-0.76156700	-1.19842803
H	2.52898788	-0.31386501	-2.10016298
H	4.01955318	-0.72031498	-1.20512104
H	2.60177898	-1.78856099	-1.11162305
C	0.30028000	-1.22220099	0.11040100
C	0.20459700	1.19262803	-0.06929100
C	-1.08439803	-1.27749002	0.12384900
H	0.85821301	-2.14351606	0.17603099
C	-1.18052399	1.13521695	-0.04777700
H	0.67803901	2.15661407	-0.14220400
C	-1.82388496	-0.09950200	0.02093500
H	-1.58993602	-2.22891593	0.19687501
H	-1.76209402	2.04360199	-0.11053700
C	-3.33286810	-0.13470800	-0.07276900
O	-4.04108715	0.17440400	-1.04061198
N	-3.76365304	-0.51095301	1.07409894
N	2.43700790	0.02965800	-0.00986600
C	0.93285298	0.01117800	0.00984000

Frequencies

Mode	IR frequency	IR intensity	Raman intensity
1	-717.14870000	66.62610000	0.00000000
2	34.55120000	0.88390000	0.00000000
3	56.40850000	0.97210000	0.00000000
4	60.78600000	0.27030000	0.00000000
5	135.47640000	2.44570000	0.00000000
6	162.33570000	2.34940000	0.00000000
7	223.66350000	0.47980000	0.00000000
8	259.04480000	2.09970000	0.00000000
9	270.54810000	0.11610000	0.00000000
10	301.49880000	1.04350000	0.00000000
11	326.69510000	3.52470000	0.00000000
12	356.14500000	1.34860000	0.00000000
13	358.23420000	0.10720000	0.00000000
14	404.42210000	1.60080000	0.00000000
15	417.48290000	0.35290000	0.00000000
16	451.90240000	0.46710000	0.00000000
17	477.24340000	2.91300000	0.00000000
18	514.26350000	9.42810000	0.00000000
19	584.81470000	22.50840000	0.00000000
20	622.29950000	18.65700000	0.00000000
21	635.46450000	0.39230000	0.00000000
22	713.07540000	7.07940000	0.00000000
23	754.29710000	1.14520000	0.00000000
24	777.60000000	1.13190000	0.00000000
25	837.99150000	1.79880000	0.00000000
26	843.32740000	36.86530000	0.00000000
27	860.07800000	24.82370000	0.00000000
28	942.66860000	29.21660000	0.00000000
29	958.26480000	16.63760000	0.00000000
30	992.38500000	0.10980000	0.00000000
31	1002.95610000	0.03690000	0.00000000
32	1037.22300000	11.43440000	0.00000000
33	1077.47320000	0.02850000	0.00000000
34	1123.44120000	33.24900000	0.00000000
35	1132.53050000	0.09520000	0.00000000
36	1140.41940000	0.49010000	0.00000000
37	1151.09720000	15.93730000	0.00000000
38	1164.41270000	4.05380000	0.00000000
39	1227.94410000	0.24710000	0.00000000
40	1256.04590000	0.54530000	0.00000000
41	1256.23500000	1.73990000	0.00000000
42	1295.53760000	0.94760000	0.00000000
43	1313.41160000	6.81330000	0.00000000
44	1344.56820000	20.55260000	0.00000000
45	1352.35750000	4.06330000	0.00000000
46	1439.40760000	9.56260000	0.00000000
47	1451.96400000	9.69550000	0.00000000
48	1452.96910000	5.03550000	0.00000000
49	1480.56500000	0.00330000	0.00000000
50	1488.68410000	0.33150000	0.00000000
51	1493.54620000	1.45680000	0.00000000
52	1495.20770000	1.25780000	0.00000000
53	1507.88050000	29.98970000	0.00000000
54	1514.51600000	24.19500000	0.00000000
55	1524.06030000	51.22400000	0.00000000
56	1531.12770000	45.18170000	0.00000000
57	1531.78220000	6.74850000	0.00000000
58	1616.40750000	5.55810000	0.00000000
59	1626.73290000	81.92710000	0.00000000
60	3081.40360000	0.10140000	0.00000000

61	3081.87610000	0.48720000	0.00000000
62	3088.39150000	0.16430000	0.00000000
63	3169.76440000	0.02510000	0.00000000
64	3170.01850000	0.25250000	0.00000000
65	3177.24240000	3.00270000	0.00000000
66	3183.24970000	0.04220000	0.00000000
67	3189.03870000	0.03560000	0.00000000
68	3190.08910000	1.39220000	0.00000000
69	3204.21390000	0.89530000	0.00000000
70	3205.47820000	3.37950000	0.00000000
71	3215.96990000	6.37990000	0.00000000
72	3240.77410000	1.50520000	0.00000000

S7. CALCULATIONS ON 12s

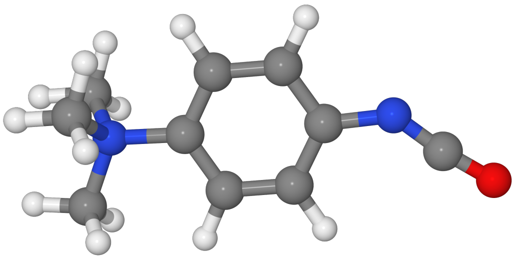

Route	: # opt freq b3lyp/cc-pvtz empiricaldispersion=gd3bj pop=regular geom=connectivity int=ultrafine
SMILES	: C[N](C)(C)c1ccc(cc1)[N][C]=O
Formula	: $C_{10}H_{13}N_2O^+$
Charge	: 1
Multiplicity	: 1
Energy	: -573.62825777 a.u.
Gibbs Energy	: -573.44748500 a.u.
CASPT2 Energy (incl. N_2 at $R = \infty$)	: -681.56008115 a.u.
CCSD(T)-F12b Energy	: -572.52143929 a.u.

Cartesian Co-ordinates (XYZ format)

```
26

C  -3.11697412  0.52187097 -1.23319805
H  -2.68694401  0.04038900 -2.10538793
H  -4.19610119  0.39873099 -1.21995294
H  -2.86274791  1.57464695 -1.22938204
C  -2.99072790 -1.56814206 -0.00001700
H  -2.62018800 -2.06027007  0.89243001
H  -4.07577896 -1.57508695 -0.00000500
H  -2.62020993 -2.06029105 -0.89246202
C  -3.11703205  0.52192402  1.23306501
H  -2.86282611  1.57470405  1.22920597
H  -4.19615602  0.39876401  1.21978402
H  -2.68702698  0.04049300  2.10529590
C  -0.21476200 -1.10158205  0.00009600
C  -0.50694501  1.28931904 -0.00009600
C   1.16115201 -0.92817098  0.00011700
H  -0.59694099 -2.10837293  0.00016600
C   0.86366200  1.46101296 -0.00007600
H  -1.13341701  2.16859508 -0.00018300
C   1.71584499  0.35287499  0.00003100
H   1.80517995 -1.79570496  0.00020200
H   1.29084802  2.45227790 -0.00014300
N   3.07700109  0.57852602  0.00005000
C   4.08101702 -0.09924000  0.00010600
O   5.10628796 -0.63917601  0.00014300
C  -1.04727900  0.00749300 -0.00001000
```

N -2.54382396 -0.13417900 -0.00003900

Frequencies

Mode	IR frequency	IR intensity	Raman intensity
1	39.89680000	0.29970000	0.00000000
2	61.32740000	0.00250000	0.00000000
3	81.04530000	1.70670000	0.00000000
4	108.04440000	0.07790000	0.00000000
5	217.37090000	0.13520000	0.00000000
6	222.29900000	2.77780000	0.00000000
7	263.93480000	0.04060000	0.00000000
8	277.73740000	0.12620000	0.00000000
9	313.35770000	0.51710000	0.00000000
10	343.49350000	3.38270000	0.00000000
11	355.55030000	0.09060000	0.00000000
12	394.84520000	1.28620000	0.00000000
13	421.59480000	5.44010000	0.00000000
14	427.94600000	0.04070000	0.00000000
15	459.85510000	0.14430000	0.00000000
16	482.36480000	2.88230000	0.00000000
17	506.57650000	0.14450000	0.00000000
18	558.75360000	19.41940000	0.00000000
19	593.80560000	29.37650000	0.00000000
20	621.65250000	7.44740000	0.00000000
21	652.59370000	1.16910000	0.00000000
22	687.19530000	20.37230000	0.00000000
23	739.58580000	0.72570000	0.00000000
24	809.21620000	12.49080000	0.00000000
25	828.16220000	1.30610000	0.00000000
26	849.67240000	25.43190000	0.00000000
27	862.66340000	36.04970000	0.00000000
28	942.57860000	26.30020000	0.00000000
29	962.70860000	16.54860000	0.00000000
30	976.41890000	0.02830000	0.00000000
31	995.11670000	0.02940000	0.00000000
32	1033.75740000	4.62540000	0.00000000
33	1077.41740000	0.02320000	0.00000000
34	1131.76180000	0.18890000	0.00000000
35	1133.90790000	0.36410000	0.00000000
36	1140.62920000	0.47610000	0.00000000
37	1166.39790000	18.50330000	0.00000000
38	1171.03930000	79.41750000	0.00000000
39	1226.80800000	3.82070000	0.00000000
40	1258.31470000	1.95160000	0.00000000
41	1258.59660000	1.40010000	0.00000000
42	1295.50720000	4.14670000	0.00000000
43	1332.75940000	7.69060000	0.00000000
44	1356.17920000	0.54300000	0.00000000
45	1449.39500000	3.23570000	0.00000000
46	1451.73140000	4.73050000	0.00000000
47	1458.82960000	4.29170000	0.00000000
48	1480.07430000	0.00440000	0.00000000
49	1489.32460000	0.39700000	0.00000000
50	1492.05640000	1.99180000	0.00000000
51	1494.86020000	0.84420000	0.00000000
52	1504.11290000	6.54230000	0.00000000
53	1509.50080000	22.45790000	0.00000000
54	1513.62380000	24.09540000	0.00000000
55	1530.90650000	46.12130000	0.00000000
56	1580.71830000	216.28820000	0.00000000
57	1621.60130000	0.44880000	0.00000000
58	1649.40270000	169.10150000	0.00000000
59	2352.68080000	1922.56660000	0.00000000
60	3079.73380000	0.41030000	0.00000000

61	3081.00530000	1.46580000	0.00000000
62	3087.87320000	1.13040000	0.00000000
63	3167.59880000	0.00570000	0.00000000
64	3168.33250000	0.81360000	0.00000000
65	3175.47310000	5.74250000	0.00000000
66	3182.63250000	0.16930000	0.00000000
67	3187.96730000	0.04320000	0.00000000
68	3188.03750000	2.69930000	0.00000000
69	3196.72090000	1.54690000	0.00000000
70	3200.46200000	1.76540000	0.00000000
71	3216.57430000	0.61680000	0.00000000
72	3234.32080000	0.40480000	0.00000000

S8. CALCULATIONS ON TS 7 (ISOMER 1) ⟶ 12s

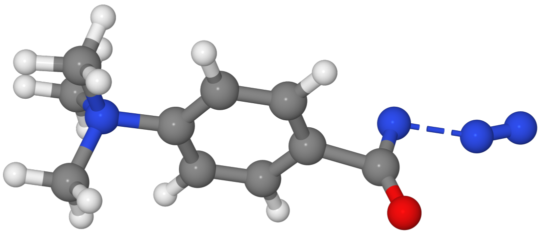

Route	:	# opt=(calcall,qst3) freq b3lyp/cc-pvtz empiricaldispersion=gd3bj pop=regular geom=connectivity int=ultrafine
SMILES	:	C[N](C)(C)c1ccc(cc1)C(=O)N=[N+]=[N-]
Formula	:	$C_{10}H_{13}N_4O^+$
Charge	:	1
Multiplicity	:	1
Energy	:	-683.06304418 a.u.
Gibbs Energy	:	-682.88216800 a.u.
CASPT2 Energy	:	-681.40634226 a.u.
CCSD(T)-F12b Energy	:	-681.78742261 a.u.

Cartesian Co-ordinates (XYZ format)

28

C	-3.73209095	0.90428698	-1.09156406
H	-3.40273094	0.50880700	-2.04698992
H	-4.81549501	0.97236001	-1.05750799
H	-3.29221392	1.87957096	-0.92376500
C	-3.98285794	-1.35230100	-0.22292800
H	-3.70242691	-2.04656291	0.56148702
H	-5.05029202	-1.16218996	-0.18096100
H	-3.71790791	-1.74569201	-1.19811797
C	-3.70801592	0.48768201	1.34189701
H	-3.26860690	1.46482003	1.49974000
H	-4.79160118	0.56000900	1.35315299
H	-3.36229110	-0.20205700	2.10506701
C	-1.17281604	-1.39871299	-0.26086301
C	-1.02246594	0.97985601	0.14575601
C	0.21548200	-1.47085798	-0.29445100
H	-1.73649395	-2.30523109	-0.40023899
C	0.35919899	0.90060902	0.11010500
H	-1.48255301	1.93963301	0.32520401
C	0.97491002	-0.32302299	-0.13034999
H	0.69822001	-2.42303109	-0.45792800
H	0.95508403	1.78923905	0.25762400
N	2.86015296	-0.63332301	0.77726400
C	2.51474094	-0.35582799	-0.44462401
O	2.96681499	-0.15061601	-1.54426599
C	-1.78005099	-0.17155001	-0.04460500
N	-3.27897811	-0.04258800	-0.00536900
N	5.67037010	-0.88031501	1.11519301
N	4.66546106	-0.73536003	0.71149802

Frequencies

Mode	IR frequency	IR intensity	Raman intensity
1	-517.26020000	275.92510000	0.00000000
2	24.53130000	2.17720000	0.00000000
3	41.67810000	1.97950000	0.00000000
4	57.04160000	0.06990000	0.00000000
5	66.22290000	0.00820000	0.00000000
6	91.61790000	0.73890000	0.00000000
7	147.34010000	0.08120000	0.00000000
8	165.23220000	2.09580000	0.00000000
9	219.95260000	0.02220000	0.00000000
10	230.01320000	1.01230000	0.00000000
11	258.76730000	0.60050000	0.00000000
12	265.62600000	37.91500000	0.00000000
13	283.93910000	0.15360000	0.00000000
14	303.92900000	0.72900000	0.00000000
15	339.78180000	0.18530000	0.00000000
16	355.89010000	0.10300000	0.00000000
17	379.66840000	4.12490000	0.00000000
18	393.15040000	0.77430000	0.00000000
19	414.09170000	0.07320000	0.00000000
20	422.00730000	1.10670000	0.00000000
21	459.54210000	0.15970000	0.00000000
22	480.47000000	2.78920000	0.00000000
23	565.10690000	26.07500000	0.00000000
24	589.02990000	3.11370000	0.00000000
25	641.05030000	0.10520000	0.00000000
26	716.31310000	14.53370000	0.00000000
27	735.88830000	3.67130000	0.00000000
28	747.57540000	1.69780000	0.00000000
29	812.82620000	15.25900000	0.00000000
30	832.50560000	0.06790000	0.00000000
31	844.73640000	35.89880000	0.00000000
32	864.72290000	41.30910000	0.00000000
33	943.86830000	24.52350000	0.00000000
34	961.16200000	16.47500000	0.00000000
35	982.17590000	0.71190000	0.00000000
36	993.62320000	0.12300000	0.00000000
37	1037.38140000	12.54570000	0.00000000
38	1077.25870000	0.02330000	0.00000000
39	1122.73840000	20.00990000	0.00000000
40	1132.54790000	0.20250000	0.00000000
41	1138.71400000	0.62780000	0.00000000
42	1140.59450000	0.50950000	0.00000000
43	1165.12730000	2.79870000	0.00000000
44	1197.86590000	8.10020000	0.00000000
45	1226.04840000	0.07390000	0.00000000
46	1257.78570000	0.95380000	0.00000000
47	1258.03940000	1.54050000	0.00000000
48	1295.43450000	1.49510000	0.00000000
49	1340.89200000	5.76370000	0.00000000
50	1351.88240000	2.18960000	0.00000000
51	1441.76270000	5.10770000	0.00000000
52	1451.62230000	9.57300000	0.00000000
53	1452.24370000	4.91210000	0.00000000
54	1480.28600000	0.00330000	0.00000000
55	1489.52570000	0.30250000	0.00000000
56	1493.66050000	2.23420000	0.00000000
57	1495.56400000	0.39130000	0.00000000
58	1507.61500000	27.53580000	0.00000000
59	1513.75670000	24.30080000	0.00000000
60	1531.07190000	55.62700000	0.00000000

61	1533.77500000	18.66260000	0.00000000
62	1626.91670000	3.85530000	0.00000000
63	1630.25230000	3.18210000	0.00000000
64	1849.43050000	316.10650000	0.00000000
65	2410.04590000	9.48020000	0.00000000
66	3080.80980000	0.24880000	0.00000000
67	3081.49780000	1.02290000	0.00000000
68	3088.22540000	0.83570000	0.00000000
69	3168.54810000	0.04600000	0.00000000
70	3168.90510000	0.47150000	0.00000000
71	3176.00600000	5.31430000	0.00000000
72	3183.02740000	0.11460000	0.00000000
73	3189.21860000	0.35000000	0.00000000
74	3189.38720000	2.21110000	0.00000000
75	3200.08530000	0.58110000	0.00000000
76	3205.68160000	0.87580000	0.00000000
77	3211.46950000	3.54580000	0.00000000
78	3238.84500000	1.18190000	0.00000000

S9. CALCULATIONS ON 7 (ISOMER 1; TRIPLET)

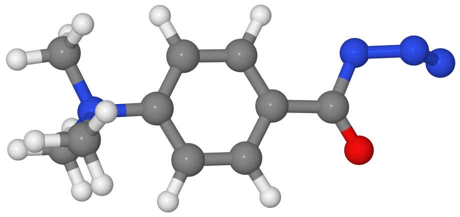

Route	:	# opt freq ub3lyp/cc-pvtz empiricaldispersion=gd3bj pop=regular geom=connectivity int=ultrafine
SMILES	:	C[N](C)(C)c1ccc(cc1)C(=O)N=[N+]=[N-]
Formula	:	$C_{10}H_{13}N_4O^{+,3}$
Charge	:	1
Multiplicity	:	3
Energy	:	-683.01966312 a.u.
Gibbs Energy	:	-682.83841900 a.u.
CASPT2 Energy	:	-681.37022961 a.u.

Cartesian Co-ordinates (XYZ format)

28

C	-3.69818807	0.54549003	-1.26371300
H	-3.26513910	0.04259600	-2.12239099
H	-4.78044415	0.45574999	-1.27307606
H	-3.41158891	1.58986902	-1.26237297
C	-3.67408204	-1.53726399	-0.00859100
H	-3.34167504	-2.03396606	0.89626801
H	-4.75833797	-1.50395000	-0.03211400
H	-3.30340004	-2.05112791	-0.88867199
C	-3.74884391	0.56893498	1.20513594
H	-3.46187592	1.61317897	1.19568300
H	-4.83061409	0.47894499	1.17190194
H	-3.35140395	0.08263000	2.09018493
C	-0.88716698	-1.16602802	0.04890000
C	-1.09891999	1.24047506	0.01732300
C	0.49712101	-1.03435898	0.07992000
H	-1.30714703	-2.15717912	0.05230300
C	0.27739301	1.36245894	0.04737500
H	-1.69988501	2.13669491	-0.00622500
C	1.08479500	0.22532800	0.07944900
H	1.11521304	-1.91849601	0.11204500
H	0.74297798	2.33701205	0.04820500
C	2.56401706	0.41959700	0.11028800
O	3.08749008	1.50965297	0.16931400
N	4.59789801	-0.65663499	-0.33415300
N	5.47947693	-0.33862600	0.37479299
N	3.29025888	-0.79271799	0.13828900
N	-3.17588592	-0.11921400	-0.01168200
C	-1.67437899	-0.02689800	0.01859800

Frequencies

Mode	IR frequency	IR intensity	Raman intensity
1	30.47340000	0.71740000	0.00000000
2	44.33520000	0.28470000	0.00000000
3	65.89000000	0.09080000	0.00000000
4	66.96650000	0.08150000	0.00000000
5	89.93900000	0.29540000	0.00000000
6	131.07670000	0.27190000	0.00000000
7	194.02140000	0.63350000	0.00000000
8	220.35870000	2.58290000	0.00000000
9	235.25880000	0.30100000	0.00000000
10	266.07820000	0.63560000	0.00000000
11	289.49480000	0.46480000	0.00000000
12	320.50010000	6.01380000	0.00000000
13	341.79090000	1.19600000	0.00000000
14	356.86860000	0.08830000	0.00000000
15	372.13540000	1.38260000	0.00000000
16	407.08600000	1.74280000	0.00000000
17	420.80740000	0.08410000	0.00000000
18	431.15200000	0.18320000	0.00000000
19	465.67170000	1.26860000	0.00000000
20	498.66490000	2.31420000	0.00000000
21	550.89550000	11.00500000	0.00000000
22	580.84940000	3.78960000	0.00000000
23	604.94320000	1.04880000	0.00000000
24	648.78570000	0.94080000	0.00000000
25	650.20580000	1.76540000	0.00000000
26	660.12020000	32.17470000	0.00000000
27	733.49620000	6.14860000	0.00000000
28	770.31080000	3.76840000	0.00000000
29	813.79840000	8.38000000	0.00000000
30	845.48490000	9.88590000	0.00000000
31	846.74230000	19.70210000	0.00000000
32	878.51480000	11.58980000	0.00000000
33	945.50290000	28.37490000	0.00000000
34	960.62450000	15.67610000	0.00000000
35	1004.80630000	0.33730000	0.00000000
36	1020.78830000	0.08590000	0.00000000
37	1029.02910000	131.24610000	0.00000000
38	1050.62250000	62.69510000	0.00000000
39	1077.41360000	0.02290000	0.00000000
40	1128.77530000	22.03120000	0.00000000
41	1133.31860000	0.07380000	0.00000000
42	1140.52980000	0.50420000	0.00000000
43	1164.77890000	5.72270000	0.00000000
44	1217.67450000	52.75830000	0.00000000
45	1254.87720000	133.80720000	0.00000000
46	1257.60670000	1.75760000	0.00000000
47	1258.05270000	11.52200000	0.00000000
48	1296.44330000	0.25300000	0.00000000
49	1350.69760000	9.56690000	0.00000000
50	1351.42860000	3.87910000	0.00000000
51	1448.00140000	6.14990000	0.00000000
52	1452.46080000	4.97810000	0.00000000
53	1453.20040000	18.94490000	0.00000000
54	1480.27770000	0.00220000	0.00000000
55	1489.27140000	0.37370000	0.00000000
56	1494.02680000	2.14580000	0.00000000
57	1495.82990000	1.05590000	0.00000000
58	1507.72820000	26.95920000	0.00000000
59	1513.92510000	24.56220000	0.00000000
60	1531.19760000	50.30190000	0.00000000

61	1542.11550000	17.89780000	0.00000000
62	1628.67600000	10.65650000	0.00000000
63	1645.92440000	19.12600000	0.00000000
64	1662.11180000	71.64570000	0.00000000
65	1747.08890000	6.99570000	0.00000000
66	3080.93880000	0.19720000	0.00000000
67	3081.62410000	0.80360000	0.00000000
68	3088.33460000	0.53700000	0.00000000
69	3168.82730000	0.01510000	0.00000000
70	3169.19900000	0.43420000	0.00000000
71	3176.38480000	4.38920000	0.00000000
72	3183.17520000	0.09830000	0.00000000
73	3189.03780000	0.21010000	0.00000000
74	3189.65400000	1.67120000	0.00000000
75	3202.38740000	0.87180000	0.00000000
76	3214.03450000	7.51360000	0.00000000
77	3214.78640000	2.27010000	0.00000000
78	3240.12550000	1.67520000	0.00000000

S10. CALCULATIONS ON TS 7 (ISOMER 1; TRIPLET) ⟶ 11t

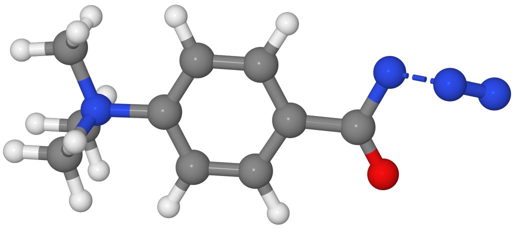

Route	:	# opt=(calcfc,ts,modredundant,maxstep=10,noeigentest) freq ub3lyp/cc-pvtz empiricaldispersion=gd3bj pop=regular geom=connectivity int=ultrafine
SMILES	:	C[N](C)(C)c1ccc(cc1)C(=O)N=[N+]=[N-]
Formula	:	$C_{10}H_{13}N_4O^{+,3}$
Charge	:	1
Multiplicity	:	3
Energy	:	-683.01932728 a.u.
Gibbs Energy	:	-682.83939000 a.u.
CASPT2 Energy	:	-681.3683248 a.u.

Cartesian Co-ordinates (XYZ format)

28

C	3.67648792	0.49947199	1.31404495
H	3.24366808	-0.04585700	2.14653611
H	4.75941992	0.41843101	1.32486999
H	3.38094211	1.54037702	1.35909700
C	3.67575908	-1.52379799	-0.03502600
H	3.35199594	-1.98123705	-0.96341503
H	4.75957012	-1.48292804	-0.00417900
H	3.30494905	-2.08035588	0.81864297
C	3.73910499	0.63619298	-1.15083599
H	3.44311500	1.67647898	-1.09543705
H	4.82142782	0.55381900	-1.11579704
H	3.35035205	0.18743999	-2.05926895
C	0.88628697	-1.17298806	-0.09026800
C	1.07783794	1.23137999	0.05210500
C	-0.49877000	-1.05089295	-0.12340700
H	1.31456804	-2.15949297	-0.13673700
C	-0.29933199	1.34368896	0.02038500
H	1.67133999	2.13035107	0.11982800
C	-1.09672499	0.20265800	-0.06827500
H	-1.10857797	-1.93829298	-0.20130900
H	-0.77335000	2.31331396	0.06219300
C	-2.57855105	0.38407499	-0.09551700
O	-3.11141300	1.47336602	-0.10003800
N	-4.61108017	-0.72773200	0.48384899
N	-5.55273008	-0.34840500	-0.07029900
N	-3.30209088	-0.81793803	-0.21178900
N	3.16584301	-0.11143800	0.03021300
C	1.66377902	-0.02995500	-0.00377900

Frequencies

Mode	IR frequency	IR intensity	Raman intensity
1	-372.83090000	19.99740000	0.00000000
2	31.08320000	0.73380000	0.00000000
3	43.19310000	0.31180000	0.00000000
4	63.86150000	0.10750000	0.00000000
5	65.73180000	0.03130000	0.00000000
6	83.57880000	0.30110000	0.00000000
7	147.82500000	0.32080000	0.00000000
8	195.21970000	0.92940000	0.00000000
9	233.97020000	0.27750000	0.00000000
10	237.74540000	3.83890000	0.00000000
11	264.79000000	0.13470000	0.00000000
12	285.23790000	0.22970000	0.00000000
13	314.54500000	9.49820000	0.00000000
14	348.12500000	0.77990000	0.00000000
15	356.74890000	0.08810000	0.00000000
16	372.60600000	1.53610000	0.00000000
17	420.14710000	0.07050000	0.00000000
18	425.50140000	2.59530000	0.00000000
19	432.58020000	1.13180000	0.00000000
20	466.98910000	1.02940000	0.00000000
21	505.18840000	1.49760000	0.00000000
22	549.16950000	9.12250000	0.00000000
23	566.98840000	4.72480000	0.00000000
24	607.11660000	5.04130000	0.00000000
25	647.44680000	18.35280000	0.00000000
26	649.14700000	9.08250000	0.00000000
27	727.29570000	9.33990000	0.00000000
28	769.39030000	4.37470000	0.00000000
29	809.44400000	8.15090000	0.00000000
30	845.25370000	7.62070000	0.00000000
31	846.39980000	22.75670000	0.00000000
32	879.40360000	11.71130000	0.00000000
33	945.42470000	28.50790000	0.00000000
34	960.62460000	15.70440000	0.00000000
35	1003.12680000	0.26420000	0.00000000
36	1020.44830000	0.11330000	0.00000000
37	1030.12520000	120.01190000	0.00000000
38	1051.34120000	72.97250000	0.00000000
39	1077.30750000	0.02250000	0.00000000
40	1128.84690000	22.39900000	0.00000000
41	1133.22820000	0.10530000	0.00000000
42	1140.54210000	0.50670000	0.00000000
43	1164.35430000	6.31110000	0.00000000
44	1216.74300000	55.62360000	0.00000000
45	1252.37110000	130.95520000	0.00000000
46	1257.62680000	1.71380000	0.00000000
47	1257.88140000	2.08880000	0.00000000
48	1296.35820000	0.09410000	0.00000000
49	1350.49220000	9.70640000	0.00000000
50	1351.70910000	4.13500000	0.00000000
51	1448.03900000	6.33810000	0.00000000
52	1452.42550000	4.92950000	0.00000000
53	1453.19590000	19.12920000	0.00000000
54	1480.28780000	0.00230000	0.00000000
55	1489.26470000	0.37110000	0.00000000
56	1494.06830000	2.10780000	0.00000000
57	1495.70420000	1.03920000	0.00000000
58	1507.71980000	27.01380000	0.00000000
59	1513.96030000	24.54730000	0.00000000
60	1531.17200000	49.99000000	0.00000000

61	1541.90560000	18.47580000	0.00000000
62	1626.13600000	19.04770000	0.00000000
63	1645.32080000	20.82430000	0.00000000
64	1650.82920000	49.22550000	0.00000000
65	1814.84190000	19.94930000	0.00000000
66	3080.98790000	0.20010000	0.00000000
67	3081.62230000	0.81280000	0.00000000
68	3088.32550000	0.54960000	0.00000000
69	3168.86350000	0.01210000	0.00000000
70	3169.21700000	0.44620000	0.00000000
71	3176.39590000	4.43180000	0.00000000
72	3183.10550000	0.09970000	0.00000000
73	3189.06530000	0.32450000	0.00000000
74	3189.58090000	1.61190000	0.00000000
75	3201.97350000	0.85040000	0.00000000
76	3210.85610000	1.98040000	0.00000000
77	3213.55660000	7.66810000	0.00000000
78	3239.98690000	1.47270000	0.00000000

S11. CALCULATIONS ON 11t

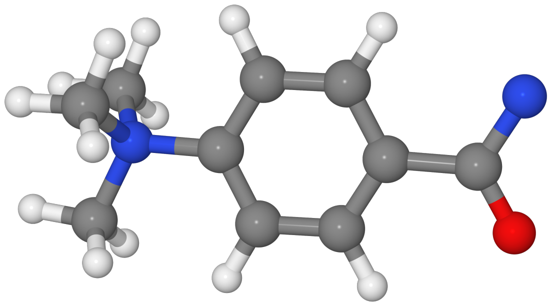

Route	:	# opt freq ub3lyp/cc-pvtz empiricaldispersion=gd3bj pop=regular geom=connectivity int=ultrafine
SMILES	:	C[N](C)(C)c1ccc(cc1)C(=O)[N]
Formula	:	$C_{10}H_{13}N_2O^{+,3}$
Charge	:	1
Multiplicity	:	3
Energy	:	-573.50315335 a.u.
Gibbs Energy	:	-573.32625000 a.u.
CASPT2 Energy (incl. N_2 at $R = \infty$)	:	-681.43967358 a.u.

Cartesian Co-ordinates (XYZ format)

26

C	3.04184794	1.39788401	0.00000500
H	2.72632098	1.92637706	0.89282000
H	4.12140989	1.28922701	-0.00017300
H	2.72602010	1.92646801	-0.89265299
C	2.94664788	-0.69503701	1.23486698
H	2.58955693	-1.71745503	1.22916496
H	4.03246689	-0.67928398	1.22321796
H	2.56586504	-0.17332800	2.10698891
C	2.94685507	-0.69513100	-1.23467398
H	2.56600094	-0.17364100	-2.10689592
H	4.03267002	-0.67912501	-1.22297001
H	2.59001303	-1.71762896	-1.22883797
C	0.27980900	-1.19238997	0.00060200
C	0.23562799	1.22386706	-0.00067400
C	-1.10510302	-1.21766901	0.00061000
H	0.81609702	-2.12893891	0.00114200
C	-1.15204799	1.18888402	-0.00067200
H	0.72523600	2.18261003	-0.00117500
C	-1.82737005	-0.02561000	-0.00003800
H	-1.62069798	-2.16716194	0.00112500
H	-1.72072601	2.10744905	-0.00116400
C	-3.32244706	-0.01719300	-0.00005500
O	-3.98390007	1.01930499	-0.00063000
N	-3.99021602	-1.21516502	0.00055500
N	2.44665504	0.01737500	0.00002900
C	0.94178802	0.02973900	-0.00005700

Frequencies

Mode	IR frequency	IR intensity	Raman intensity
1	41.72410000	0.05990000	0.00000000
2	62.87010000	0.01840000	0.00000000
3	72.57630000	0.07680000	0.00000000
4	148.81410000	0.65520000	0.00000000
5	191.65930000	1.21530000	0.00000000
6	235.38120000	0.18390000	0.00000000
7	260.63670000	3.46130000	0.00000000
8	266.52120000	0.98660000	0.00000000
9	291.52550000	1.36140000	0.00000000
10	355.33680000	2.29810000	0.00000000
11	356.02360000	0.31840000	0.00000000
12	364.25130000	2.21800000	0.00000000
13	417.72170000	0.12200000	0.00000000
14	419.87320000	6.68330000	0.00000000
15	427.59960000	0.00020000	0.00000000
16	471.42860000	2.09180000	0.00000000
17	498.80780000	3.75750000	0.00000000
18	548.79000000	6.74320000	0.00000000
19	560.83180000	16.58850000	0.00000000
20	643.24930000	18.91950000	0.00000000
21	648.23360000	0.37240000	0.00000000
22	650.81120000	53.90340000	0.00000000
23	764.14180000	2.20000000	0.00000000
24	786.38600000	1.38270000	0.00000000
25	845.87760000	38.04360000	0.00000000
26	846.16200000	0.15250000	0.00000000
27	870.29370000	14.04180000	0.00000000
28	945.25880000	27.56490000	0.00000000
29	959.90310000	16.11590000	0.00000000
30	1003.99190000	0.00580000	0.00000000
31	1016.11690000	0.15020000	0.00000000
32	1036.49120000	17.97350000	0.00000000
33	1077.32510000	0.02710000	0.00000000
34	1083.81010000	29.64480000	0.00000000
35	1129.91490000	16.98800000	0.00000000
36	1133.71900000	0.93300000	0.00000000
37	1140.54920000	0.48840000	0.00000000
38	1164.90310000	3.93170000	0.00000000
39	1214.74590000	46.03800000	0.00000000
40	1239.03550000	40.32830000	0.00000000
41	1257.40460000	1.68420000	0.00000000
42	1257.58210000	2.01590000	0.00000000
43	1295.83810000	0.25130000	0.00000000
44	1347.90240000	13.12560000	0.00000000
45	1355.26860000	7.19380000	0.00000000
46	1449.03920000	4.18420000	0.00000000
47	1452.52100000	4.85620000	0.00000000
48	1453.96840000	20.62830000	0.00000000
49	1480.34180000	0.00110000	0.00000000
50	1489.33220000	0.33320000	0.00000000
51	1493.81770000	1.87670000	0.00000000
52	1495.40450000	1.28330000	0.00000000
53	1507.72920000	31.91270000	0.00000000
54	1514.07560000	24.70590000	0.00000000
55	1521.80240000	131.80960000	0.00000000
56	1531.06120000	37.25590000	0.00000000
57	1545.13410000	2.92200000	0.00000000
58	1634.50190000	3.61000000	0.00000000
59	1645.89480000	20.67970000	0.00000000
60	3081.14300000	0.17600000	0.00000000

61	3081.75530000	0.72180000	0.00000000
62	3088.37300000	0.49940000	0.00000000
63	3169.03220000	0.01160000	0.00000000
64	3169.38740000	0.35740000	0.00000000
65	3176.50170000	3.66390000	0.00000000
66	3183.15150000	0.08080000	0.00000000
67	3189.23040000	0.01010000	0.00000000
68	3189.68800000	1.68840000	0.00000000
69	3198.04870000	0.89460000	0.00000000
70	3206.28420000	4.53540000	0.00000000
71	3210.18050000	3.24650000	0.00000000
72	3239.40030000	1.49830000	0.00000000

S12. CALCULATIONS ON TS 11t ⟶ 12t

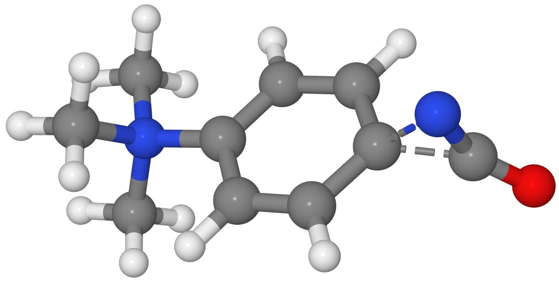

Route	:	# opt=qst3 freq b3lyp/cc-pvtz empiricaldispersion=gd3bj pop=regular geom=connectivity int=ultrafine
SMILES	:	C[N](C)(C)C1=C[CH]C2(C=C1)C(=O)[N]2
Formula	:	$C_{10}H_{13}N_2O^{+,3}$
Charge	:	1
Multiplicity	:	3
Energy	:	-573.44929645 a.u.
Gibbs Energy	:	-573.27601600 a.u.
CASPT2 Energy (incl. N_2 at $R = \infty$)	:	-681.37714329 a.u.

Cartesian Co-ordinates (XYZ format)

26

C	-2.84953189	-0.59404701	1.16440797
H	-2.53681207	-1.63078499	1.14063704
H	-3.93229294	-0.53050297	1.10500801
H	-2.48623896	-0.11761100	2.06913304
C	-2.77977204	1.54331195	0.00743600
H	-2.40337300	2.08355188	-0.85422498
H	-3.86298704	1.49583399	-0.03314600
H	-2.46692610	2.02212596	0.92882198
C	-2.75250196	-0.50675303	-1.30022597
H	-2.44135690	-1.54397595	-1.32395697
H	-3.83661294	-0.44273701	-1.32291698
H	-2.31895089	0.03004100	-2.13772798
C	0.01989400	1.22038198	0.09570000
C	1.19140399	-1.34264898	0.07426700
C	1.38707805	1.13136804	0.12484700
H	-0.41418299	2.20659304	0.09896600
C	-0.15765899	-1.21265900	0.01531300
H	-0.75506097	-2.11078501	-0.04047200
C	2.05248189	-0.17164500	0.14172301
H	1.98970795	2.02615404	0.16574600
H	1.65063798	-2.31974101	0.07134700
N	3.31500196	-0.27943900	1.04962695
C	3.54205489	-0.07349000	-0.24081700
O	4.33598995	0.04170800	-1.12435603
N	-2.25999594	0.13368300	-0.02232200
C	-0.76731998	0.06673400	0.03529800

Frequencies

Mode	IR frequency	IR intensity	Raman intensity
1	-473.20470000	100.60630000	0.00000000
2	43.53590000	0.40610000	0.00000000
3	52.19640000	0.20210000	0.00000000
4	132.48980000	1.25620000	0.00000000
5	145.67470000	1.96340000	0.00000000
6	215.15110000	0.24000000	0.00000000
7	222.08200000	0.33950000	0.00000000
8	244.72040000	0.51110000	0.00000000
9	277.44350000	0.17420000	0.00000000
10	314.58380000	0.76350000	0.00000000
11	336.48090000	1.01510000	0.00000000
12	355.09930000	0.71050000	0.00000000
13	363.04540000	1.98130000	0.00000000
14	384.94170000	5.12800000	0.00000000
15	421.21310000	2.12210000	0.00000000
16	437.87860000	1.61340000	0.00000000
17	477.11390000	2.69510000	0.00000000
18	483.03560000	6.35410000	0.00000000
19	546.48970000	15.94060000	0.00000000
20	579.08400000	5.34310000	0.00000000
21	623.61900000	1.01230000	0.00000000
22	632.09800000	4.71420000	0.00000000
23	690.69360000	21.06340000	0.00000000
24	737.69500000	0.45910000	0.00000000
25	757.63740000	20.08560000	0.00000000
26	791.54270000	21.35240000	0.00000000
27	833.23370000	20.20130000	0.00000000
28	930.54040000	13.53660000	0.00000000
29	950.18050000	12.20080000	0.00000000
30	956.99960000	18.14870000	0.00000000
31	961.24000000	41.16440000	0.00000000
32	968.95540000	55.47520000	0.00000000
33	981.03960000	13.20510000	0.00000000
34	1022.06120000	56.61370000	0.00000000
35	1076.28230000	0.02200000	0.00000000
36	1117.04880000	6.65860000	0.00000000
37	1129.86640000	0.09760000	0.00000000
38	1136.29710000	0.41980000	0.00000000
39	1153.77290000	4.43570000	0.00000000
40	1228.65730000	6.24930000	0.00000000
41	1249.89890000	1.73220000	0.00000000
42	1254.35680000	1.01810000	0.00000000
43	1282.46300000	2.41460000	0.00000000
44	1294.80750000	3.47790000	0.00000000
45	1325.52810000	0.53390000	0.00000000
46	1416.22980000	2.88550000	0.00000000
47	1450.10010000	4.81620000	0.00000000
48	1451.31240000	6.15680000	0.00000000
49	1471.52680000	2.16000000	0.00000000
50	1479.59270000	0.01420000	0.00000000
51	1488.88230000	0.38690000	0.00000000
52	1493.49150000	1.97590000	0.00000000
53	1495.20170000	1.38260000	0.00000000
54	1505.13330000	29.19870000	0.00000000
55	1513.37080000	23.83040000	0.00000000
56	1516.27900000	24.14070000	0.00000000
57	1530.38040000	45.54340000	0.00000000
58	1592.46740000	112.49800000	0.00000000
59	1684.69880000	248.47160000	0.00000000
60	3079.21110000	0.31210000	0.00000000

61	3080.66890000	1.28110000	0.00000000
62	3087.51690000	0.72270000	0.00000000
63	3167.27010000	0.02250000	0.00000000
64	3168.38160000	1.27000000	0.00000000
65	3175.73630000	5.46760000	0.00000000
66	3182.36080000	0.13900000	0.00000000
67	3185.18510000	2.55990000	0.00000000
68	3187.87490000	0.02470000	0.00000000
69	3196.13340000	1.31860000	0.00000000
70	3204.79470000	2.72350000	0.00000000
71	3211.65230000	2.91310000	0.00000000
72	3228.81240000	1.21960000	0.00000000

S13. CALCULATIONS ON 12t

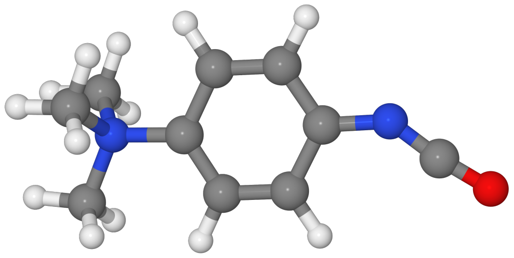

Route	:	# opt freq ub3lyp/cc-pvtz empiricaldispersion=gd3bj pop=regular geom=connectivity int=ultrafine
SMILES	:	C[N](C)(C)c1ccc(cc1)[N][C]=O
Formula	:	$C_{10}H_{13}N_2O^{+,3}$
Charge	:	1
Multiplicity	:	3
Energy	:	-573.50493542 a.u.
Gibbs Energy	:	-573.33386200 a.u.
CASPT2 Energy (incl. N_2 at $R = \infty$)	:	-681.43877889 a.u.

Cartesian Co-ordinates (XYZ format)

```
26

C  -3.07932210 -0.52310503  1.23567903
H  -2.68609500 -0.00978700  2.10710907
H  -4.16245604 -0.45001999  1.20192695
H  -2.77403092 -1.56208205  1.24893606
C  -2.98919296  1.57273495 -0.00006700
H  -2.62715793  2.07129097 -0.89251900
H  -4.07413292  1.55934000 -0.00005000
H  -2.62713194  2.07139111  0.89231801
C  -3.07934809 -0.52324200 -1.23557901
H  -2.77404809 -1.56221902 -1.24873102
H  -4.16248178 -0.45016399 -1.20180798
H  -2.68614793 -0.01001600 -2.10707498
C  -0.19305900  1.14694297 -0.00002100
C  -0.47931501 -1.35008097  0.00000400
C   1.16030002  0.98465598 -0.00002000
H  -0.58752799  2.15000606 -0.00003400
C   0.86326897 -1.51301301  0.00000700
H  -1.12631595 -2.21085596  0.00000300
C   1.74260104 -0.39481300 -0.00000200
H   1.82485700  1.83510804 -0.00003300
H   1.29711199 -2.50269794  0.00001500
N   3.07401991 -0.57541299  0.00000200
C   4.09888983  0.08499600 -0.00000500
O   5.14015293  0.60032201 -0.00000900
N  -2.51405692  0.14826301  0.00000600
C  -1.03759897  0.02581100 -0.00000600
```

Frequencies

Mode	IR frequency	IR intensity	Raman intensity
1	8.63780000	0.36520000	0.00000000
2	56.07750000	0.40430000	0.00000000
3	75.95210000	1.32360000	0.00000000
4	81.68590000	0.04930000	0.00000000
5	175.36680000	0.90300000	0.00000000
6	209.73860000	0.12340000	0.00000000
7	215.27680000	2.13490000	0.00000000
8	258.79800000	0.20970000	0.00000000
9	292.73970000	2.41080000	0.00000000
10	301.77840000	1.62660000	0.00000000
11	335.06170000	4.46370000	0.00000000
12	345.96040000	0.40660000	0.00000000
13	379.77630000	9.64150000	0.00000000
14	394.78270000	0.00200000	0.00000000
15	413.56640000	4.82990000	0.00000000
16	452.96890000	4.46670000	0.00000000
17	459.65510000	13.53580000	0.00000000
18	467.06460000	7.21800000	0.00000000
19	482.63410000	0.43660000	0.00000000
20	575.96760000	9.65630000	0.00000000
21	585.38940000	5.15290000	0.00000000
22	603.51860000	9.71810000	0.00000000
23	625.41720000	14.13690000	0.00000000
24	650.40580000	18.04710000	0.00000000
25	663.42180000	37.22340000	0.00000000
26	748.21510000	6.61130000	0.00000000
27	805.46230000	15.46090000	0.00000000
28	880.24970000	2.42370000	0.00000000
29	910.12550000	9.48770000	0.00000000
30	913.93500000	8.31780000	0.00000000
31	930.68390000	6.87340000	0.00000000
32	962.14480000	2.31460000	0.00000000
33	969.24410000	27.45010000	0.00000000
34	1074.61080000	0.00710000	0.00000000
35	1087.66140000	39.32010000	0.00000000
36	1126.42390000	2.21160000	0.00000000
37	1126.89380000	0.47160000	0.00000000
38	1141.80390000	8.72570000	0.00000000
39	1171.95900000	31.57460000	0.00000000
40	1243.68400000	2.28780000	0.00000000
41	1246.92480000	0.52420000	0.00000000
42	1264.09560000	6.17410000	0.00000000
43	1293.11000000	13.35810000	0.00000000
44	1355.46020000	8.64430000	0.00000000
45	1371.37480000	3.73540000	0.00000000
46	1445.06600000	4.49210000	0.00000000
47	1447.69660000	19.01890000	0.00000000
48	1448.14140000	3.91580000	0.00000000
49	1476.10970000	0.00610000	0.00000000
50	1480.64350000	2.09850000	0.00000000
51	1488.56870000	0.74090000	0.00000000
52	1490.74430000	6.82990000	0.00000000
53	1495.06260000	4.39340000	0.00000000
54	1503.48910000	30.16370000	0.00000000
55	1510.94090000	22.45100000	0.00000000
56	1529.46210000	40.87360000	0.00000000
57	1548.84970000	23.96790000	0.00000000
58	1590.35020000	99.08820000	0.00000000
59	2314.56730000	1473.75200000	0.00000000
60	3079.05650000	0.40780000	0.00000000

61	3080.60710000	1.11630000	0.00000000
62	3087.30480000	0.91530000	0.00000000
63	3167.39930000	0.03570000	0.00000000
64	3168.56990000	0.44680000	0.00000000
65	3175.56760000	4.00910000	0.00000000
66	3181.95140000	0.28260000	0.00000000
67	3188.88560000	0.00030000	0.00000000
68	3190.44390000	0.76530000	0.00000000
69	3200.66640000	0.34720000	0.00000000
70	3207.11600000	0.15770000	0.00000000
71	3225.92320000	0.53210000	0.00000000
72	3228.03340000	0.50630000	0.00000000

S14. CALCULATIONS ON 8 (ISOMER 1)

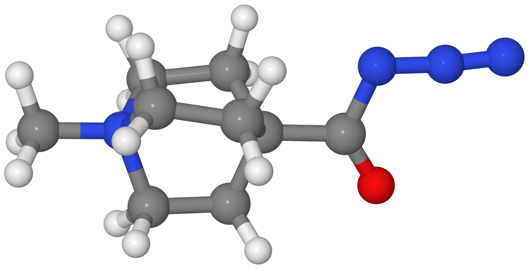

Route	:	# opt freq b3lyp/cc-pvtz empiricaldispersion=gd3bj pop=regular geom=connectivity int=ultrafine
SMILES	:	C[N]12CCC(CC1)(CC2)C(=O)N=[N+]=[N-]
Formula	:	$C_9H_{15}N_4O^+$
Charge	:	1
Multiplicity	:	1
Energy	:	-646.21797877 a.u.
Gibbs Energy	:	-646.00665100 a.u.
ZPE (B3LYP)	:	0.2504 a.u.
CASPT2 Energy	:	-644.66428265 a.u.
CCSD(T)-F12b Energy	:	-645.02438336 a.u.

Cartesian Co-ordinates (XYZ format)

```
29

C  -0.13344000  0.54658598 -1.21179295
C   0.25312200 -0.31992099  0.00249600
C  -1.66520202  0.61734998 -1.30545795
H   0.29550999  1.54024601 -1.10915303
H   0.26259801  0.11524700 -2.12905002
H  -2.02208710  1.62688696 -1.49112904
H  -2.06548095 -0.03623200 -2.07663989
C  -0.15951701  0.44086999  1.27740300
H  -0.04345100 -0.20740101  2.14458895
H   0.48134100  1.30585098  1.42584801
C  -1.61865997  0.89936799  1.13785601
H  -2.20320106  0.69971198  2.03183699
H  -1.70103300  1.95778894  0.90259701
C  -2.02912593 -1.32401705  0.16465400
H  -2.35835791 -1.57452095  1.17035902
H  -2.67246103 -1.83851004 -0.54398203
C  -0.54284400 -1.62853003 -0.06216400
H  -0.18572100 -2.32460499  0.69174999
H  -0.38806701 -2.10718989 -1.02688205
N  -2.28760695  0.16085599 -0.00247700
C  -3.75300407  0.43411601 -0.00615700
H  -4.19784689 -0.03674000 -0.87815899
H  -4.19071913  0.02443000  0.89969897
H  -3.91312909  1.50791097 -0.04248200
C   1.74677002 -0.61914003  0.00648300
O   2.20399404 -1.73248601  0.00849200
N   2.50149703  0.57261401  0.00474600
N   3.74130392  0.39561799  0.00389600
N   4.85449886  0.33684200  0.00216800
```

Frequencies

Mode	IR frequency	IR intensity	Raman intensity
1	33.11470000	1.47000000	0.00000000
2	75.96740000	0.10480000	0.00000000
3	100.06110000	0.41910000	0.00000000
4	116.55290000	0.04660000	0.00000000
5	188.53350000	1.32050000	0.00000000
6	197.74000000	1.34900000	0.00000000
7	261.90360000	0.03170000	0.00000000
8	264.54120000	0.72370000	0.00000000
9	269.41840000	0.15070000	0.00000000
10	281.52160000	4.54610000	0.00000000
11	345.36380000	7.14920000	0.00000000
12	362.73080000	0.63830000	0.00000000
13	422.56590000	0.10330000	0.00000000
14	423.74590000	0.04090000	0.00000000
15	471.11470000	2.85410000	0.00000000
16	520.45300000	0.29690000	0.00000000
17	539.40910000	0.11540000	0.00000000
18	544.62260000	0.64250000	0.00000000
19	581.31010000	0.17990000	0.00000000
20	590.44780000	5.49440000	0.00000000
21	699.72360000	1.05290000	0.00000000
22	736.67190000	3.84830000	0.00000000
23	801.70570000	1.25440000	0.00000000
24	836.24430000	7.39230000	0.00000000
25	837.87450000	6.79950000	0.00000000
26	840.26910000	6.92990000	0.00000000
27	883.50570000	48.79370000	0.00000000
28	929.82040000	3.20750000	0.00000000
29	931.90430000	4.67970000	0.00000000
30	962.97350000	66.76770000	0.00000000
31	989.67540000	1.85890000	0.00000000
32	1004.46100000	1.89500000	0.00000000
33	1010.07480000	5.14890000	0.00000000
34	1047.64850000	3.33450000	0.00000000
35	1052.35360000	16.29030000	0.00000000
36	1058.61950000	2.62440000	0.00000000
37	1120.47510000	7.00880000	0.00000000
38	1149.46670000	0.40030000	0.00000000
39	1153.60490000	2.26990000	0.00000000
40	1192.58660000	22.88770000	0.00000000
41	1205.52690000	0.29660000	0.00000000
42	1210.77160000	0.77430000	0.00000000
43	1226.56550000	13.08730000	0.00000000
44	1264.04840000	679.14860000	0.00000000
45	1286.03750000	77.19550000	0.00000000
46	1296.36800000	9.98400000	0.00000000
47	1305.80190000	3.46530000	0.00000000
48	1333.24390000	3.68090000	0.00000000
49	1336.33720000	2.29220000	0.00000000
50	1356.25070000	4.16870000	0.00000000
51	1360.46640000	3.68940000	0.00000000
52	1392.53140000	2.29670000	0.00000000
53	1393.72840000	1.96010000	0.00000000
54	1399.07900000	0.82900000	0.00000000
55	1416.32580000	1.07970000	0.00000000
56	1475.36570000	1.56260000	0.00000000
57	1494.10730000	5.15260000	0.00000000
58	1494.42640000	4.27560000	0.00000000
59	1505.34480000	1.42480000	0.00000000
60	1508.76820000	1.96190000	0.00000000

61	1514.40070000	24.48150000	0.00000000
62	1515.57080000	26.80250000	0.00000000
63	1515.93600000	24.27660000	0.00000000
64	1539.85970000	3.83090000	0.00000000
65	1773.12710000	200.88640000	0.00000000
66	2305.59560000	443.55070000	0.00000000
67	3070.05930000	0.09830000	0.00000000
68	3071.68040000	5.46280000	0.00000000
69	3076.57670000	4.03810000	0.00000000
70	3081.95990000	2.69480000	0.00000000
71	3083.75220000	5.43110000	0.00000000
72	3083.87140000	3.89690000	0.00000000
73	3088.09660000	9.93760000	0.00000000
74	3116.40150000	0.99080000	0.00000000
75	3120.29780000	0.76180000	0.00000000
76	3123.39660000	0.82540000	0.00000000
77	3136.10570000	0.10890000	0.00000000
78	3140.90260000	4.32230000	0.00000000
79	3141.22700000	4.71100000	0.00000000
80	3160.94350000	1.06920000	0.00000000
81	3161.09220000	1.09190000	0.00000000

S15. CALCULATIONS ON 8 (ISOMER 2)

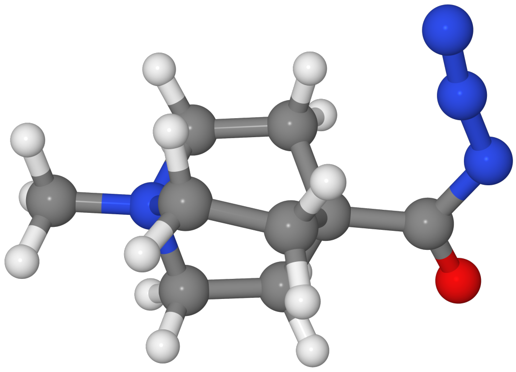

Route	:	# opt freq b3lyp/cc-pvtz empiricaldispersion=gd3bj pop=regular geom=connectivity int=ultrafine
SMILES	:	C[N]12CCC(CC1)(CC2)C(=O)N=[N+]=[N-]
Formula	:	$C_9H_{15}N_4O^+$
Charge	:	1
Multiplicity	:	1
Energy	:	-646.20328784 a.u.
Gibbs Energy	:	-645.99095400 a.u.
ZPE (B3LYP)	:	0.250518 a.u.
CASPT2 Energy	:	-644.64436915 a.u.
CCSD(T)-F12b Energy	:	-645.00809895 a.u.

Cartesian Co-ordinates (XYZ format)

29

C	0.30359301	-0.65197802	-1.23294401
C	-0.15243100	0.17467301	-0.01117200
C	1.83795297	-0.61016500	-1.32075799
H	-0.02754600	-1.68396199	-1.16146505
H	-0.11878400	-0.23805600	-2.14635491
H	2.26338196	-1.58788896	-1.52986896
H	2.19206905	0.08790200	-2.07504702
C	0.31346399	-0.53688598	1.27629006
H	0.18636100	0.13568699	2.12280798
H	-0.26220301	-1.43159103	1.49295294
C	1.78823996	-0.93591601	1.11752105
H	2.36662507	-0.73901403	2.01594806
H	1.90747297	-1.98437297	0.85459399
C	2.07014203	1.31924105	0.18122099
H	2.36274910	1.57318795	1.19734597
H	2.69216108	1.88662899	-0.50573701
C	0.57321697	1.52980995	-0.07344800
H	0.16263001	2.20875597	0.66775501
H	0.40488300	1.99131000	-1.04377306
N	2.42227697	-0.14278799	-0.00515200
C	3.90189290	-0.32915300	-0.00041400
H	4.32351398	0.17618500	-0.86460203
H	4.30761385	0.09722400	0.91258401
H	4.12572718	-1.39121199	-0.04573300
C	-1.66844106	0.43687099	-0.02305500

O	-2.10848308	1.55091906	-0.04922300
N	-2.58095908	-0.64481300	-0.01414200
N	-2.20870900	-1.82120299	0.03053600
N	-2.02302289	-2.92919707	0.07137500

Frequencies

Mode	IR frequency	IR intensity	Raman intensity
1	62.77170000	0.67650000	0.00000000
2	95.11270000	0.20290000	0.00000000
3	106.89790000	0.34190000	0.00000000
4	135.80380000	0.17660000	0.00000000
5	162.44490000	2.02680000	0.00000000
6	194.28930000	4.06900000	0.00000000
7	263.04160000	0.00440000	0.00000000
8	266.70020000	0.41410000	0.00000000
9	273.38900000	0.16660000	0.00000000
10	321.33670000	3.63430000	0.00000000
11	352.09530000	5.18790000	0.00000000
12	363.13160000	1.49370000	0.00000000
13	422.62160000	0.18300000	0.00000000
14	427.11430000	0.02490000	0.00000000
15	460.65970000	3.82500000	0.00000000
16	537.78020000	0.33930000	0.00000000
17	541.28570000	0.12510000	0.00000000
18	556.15600000	1.10800000	0.00000000
19	580.40860000	6.13300000	0.00000000
20	628.90880000	3.12430000	0.00000000
21	674.70590000	16.87780000	0.00000000
22	706.39770000	0.71650000	0.00000000
23	719.83920000	2.71280000	0.00000000
24	795.14520000	0.22760000	0.00000000
25	837.52860000	7.79100000	0.00000000
26	838.05180000	6.74900000	0.00000000
27	857.42560000	5.13990000	0.00000000
28	931.37390000	3.89400000	0.00000000
29	932.39950000	3.67430000	0.00000000
30	960.72800000	72.59250000	0.00000000
31	991.33780000	1.73420000	0.00000000
32	1004.80760000	2.30230000	0.00000000
33	1009.16360000	6.45120000	0.00000000
34	1048.04140000	13.35960000	0.00000000
35	1050.78790000	17.65190000	0.00000000
36	1058.32180000	1.51820000	0.00000000
37	1114.47860000	17.72930000	0.00000000
38	1149.11700000	0.94370000	0.00000000
39	1152.45320000	2.60080000	0.00000000
40	1176.39040000	101.09190000	0.00000000
41	1209.58480000	1.35250000	0.00000000
42	1211.18650000	0.68040000	0.00000000
43	1220.18610000	7.36540000	0.00000000
44	1285.21050000	10.06270000	0.00000000
45	1297.92720000	1.73100000	0.00000000
46	1307.57980000	4.58540000	0.00000000
47	1335.31530000	2.46340000	0.00000000
48	1339.44120000	355.38730000	0.00000000
49	1343.14960000	5.49530000	0.00000000
50	1360.96310000	2.31440000	0.00000000
51	1367.75070000	4.63160000	0.00000000
52	1393.43750000	1.69120000	0.00000000
53	1394.42190000	4.73280000	0.00000000
54	1401.22520000	2.82710000	0.00000000
55	1417.58620000	1.34820000	0.00000000
56	1475.60200000	1.53950000	0.00000000
57	1494.69250000	5.19230000	0.00000000
58	1494.77250000	5.61010000	0.00000000
59	1503.79000000	2.37470000	0.00000000
60	1509.00050000	2.66660000	0.00000000

61	1513.94930000	19.29460000	0.00000000
62	1515.65840000	26.04720000	0.00000000
63	1516.59900000	26.08800000	0.00000000
64	1540.01960000	6.64140000	0.00000000
65	1792.40670000	380.79070000	0.00000000
66	2257.36680000	364.41430000	0.00000000
67	3070.22760000	0.01490000	0.00000000
68	3078.23590000	4.22750000	0.00000000
69	3081.24550000	2.01100000	0.00000000
70	3083.38400000	2.80000000	0.00000000
71	3085.17900000	5.79120000	0.00000000
72	3087.62340000	4.18900000	0.00000000
73	3090.13680000	5.12900000	0.00000000
74	3122.59150000	0.56370000	0.00000000
75	3127.62560000	0.97510000	0.00000000
76	3128.16300000	1.09160000	0.00000000
77	3139.63220000	0.17340000	0.00000000
78	3143.86460000	3.67720000	0.00000000
79	3144.60100000	3.66210000	0.00000000
80	3161.23850000	0.85790000	0.00000000
81	3161.85230000	0.75280000	0.00000000

S16. CALCULATIONS ON 8 (TS ISOMER 1 ⟶ ISOMER 2)

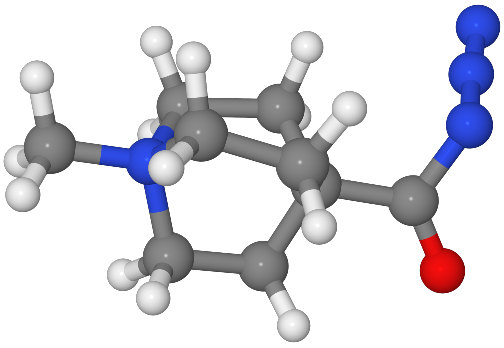

Route	: # opt=qst3 freq b3lyp/cc-pvtz empiricaldispersion=gd3bj pop=regular geom=connectivity int=ultrafine
SMILES	: C[N]12CCC(CC1)(CC2)C(=O)N=[N+]=[N-]
Formula	: $C_9H_{15}N_4O^+$
Charge	: 1
Multiplicity	: 1
Energy	: -646.19697691 a.u.
Gibbs Energy	: -645.98506200 a.u.
ZPE (B3LYP)	: 0.2500258 a.u.
CASPT2 Energy	: -644.64189655 a.u.
CCSD(T)-F12b Energy	: -645.00242753 a.u.

Cartesian Co-ordinates (XYZ format)

29

C	0.21576899	-0.60781902	-1.22452998
C	-0.18435501	0.22524101	0.00790800
C	1.74774694	-0.60676199	-1.34644794
H	-0.15010400	-1.62764800	-1.12872195
H	-0.21064800	-0.18975300	-2.13444996
H	2.14340711	-1.59350300	-1.57099795
H	2.10233212	0.08667700	-2.10485411
C	0.27812400	-0.54286599	1.26478195
H	0.15813901	0.09027700	2.14212704
H	-0.33304101	-1.42940295	1.41478896
C	1.74725699	-0.95268601	1.08832300
H	2.33923793	-0.75921899	1.97868097
H	1.85821199	-2.00135708	0.82301998
C	2.06429505	1.30781996	0.16611400
H	2.39111900	1.54405999	1.17597699
H	2.68298292	1.86367202	-0.53309900
C	0.56634301	1.56243205	-0.04304200
H	0.19093899	2.23124599	0.72597498
H	0.38738799	2.05035591	-0.99960703
N	2.37338305	-0.16241500	-0.04178900
C	3.84784794	-0.38343099	-0.07328300
H	4.26214600	0.12185700	-0.94107199
H	4.28429317	0.02213400	0.83500201
H	4.04502916	-1.44986200	-0.13554101
C	-1.69004405	0.46735701	0.09810700
O	-2.16443992	1.53010905	0.35704100

N	-2.50499892	-0.72060502	0.02939900
N	-2.77663112	-1.20294797	-1.06862104
N	-3.09162498	-1.71431506	-2.02003503

Frequencies

Mode	IR frequency	IR intensity	Raman intensity
1	-90.03150000	2.89940000	0.00000000
2	26.33900000	0.27950000	0.00000000
3	115.95740000	1.33660000	0.00000000
4	127.92510000	0.07280000	0.00000000
5	156.03080000	1.16820000	0.00000000
6	181.91230000	0.27600000	0.00000000
7	263.12240000	0.00140000	0.00000000
8	270.01100000	0.19200000	0.00000000
9	271.61590000	0.12880000	0.00000000
10	308.34290000	4.02470000	0.00000000
11	344.36040000	6.09630000	0.00000000
12	360.29630000	0.84310000	0.00000000
13	422.65010000	0.07000000	0.00000000
14	423.98330000	0.02010000	0.00000000
15	465.16140000	0.77620000	0.00000000
16	539.30980000	0.14110000	0.00000000
17	542.52380000	0.44110000	0.00000000
18	551.91030000	10.91360000	0.00000000
19	553.30860000	1.47740000	0.00000000
20	593.02240000	1.55460000	0.00000000
21	655.40500000	5.25040000	0.00000000
22	703.19210000	2.07210000	0.00000000
23	786.56760000	11.42730000	0.00000000
24	802.02540000	0.80870000	0.00000000
25	840.78370000	8.22670000	0.00000000
26	841.10460000	8.08520000	0.00000000
27	856.27170000	8.31090000	0.00000000
28	923.97690000	86.92750000	0.00000000
29	932.93550000	6.34790000	0.00000000
30	939.13400000	72.50850000	0.00000000
31	988.98750000	1.59230000	0.00000000
32	1003.99300000	1.65110000	0.00000000
33	1011.07410000	3.33210000	0.00000000
34	1039.57920000	32.36260000	0.00000000
35	1048.21620000	3.03650000	0.00000000
36	1058.16740000	3.27820000	0.00000000
37	1108.71120000	19.10160000	0.00000000
38	1149.53570000	1.64400000	0.00000000
39	1153.82160000	3.50050000	0.00000000
40	1174.45120000	108.17860000	0.00000000
41	1206.94600000	0.99100000	0.00000000
42	1211.24370000	1.45140000	0.00000000
43	1224.25120000	14.34560000	0.00000000
44	1286.20340000	1.96970000	0.00000000
45	1296.18230000	6.18020000	0.00000000
46	1305.32560000	2.17680000	0.00000000
47	1336.76490000	3.57550000	0.00000000
48	1337.45650000	5.12030000	0.00000000
49	1358.87450000	94.59910000	0.00000000
50	1359.27940000	165.36120000	0.00000000
51	1361.41880000	37.69810000	0.00000000
52	1392.73030000	2.44560000	0.00000000
53	1394.46230000	1.90910000	0.00000000
54	1398.08150000	1.21900000	0.00000000
55	1416.94330000	1.55100000	0.00000000
56	1476.00230000	1.78890000	0.00000000
57	1494.64630000	5.52570000	0.00000000
58	1494.88920000	4.05040000	0.00000000
59	1506.74890000	0.81150000	0.00000000
60	1509.80810000	3.48770000	0.00000000

61	1514.83490000	25.00850000	0.00000000
62	1516.15200000	26.28150000	0.00000000
63	1516.73780000	23.73650000	0.00000000
64	1540.33640000	3.52130000	0.00000000
65	1820.84240000	290.45590000	0.00000000
66	2281.79040000	526.05290000	0.00000000
67	3070.26370000	0.24290000	0.00000000
68	3071.86440000	4.94670000	0.00000000
69	3074.26450000	3.36890000	0.00000000
70	3077.86290000	3.70690000	0.00000000
71	3083.88520000	2.47180000	0.00000000
72	3084.96560000	3.45470000	0.00000000
73	3088.94470000	8.95940000	0.00000000
74	3112.43540000	1.76150000	0.00000000
75	3118.53450000	0.99120000	0.00000000
76	3126.25840000	0.94590000	0.00000000
77	3137.20630000	0.07280000	0.00000000
78	3141.58600000	3.42330000	0.00000000
79	3142.46340000	3.54410000	0.00000000
80	3161.30880000	0.88520000	0.00000000
81	3161.76430000	0.74810000	0.00000000

S17. CALCULATIONS ON 13s

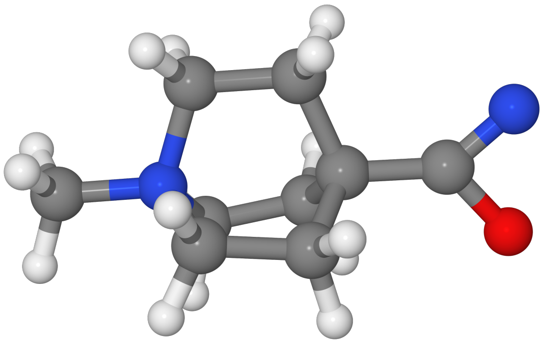

Route	:	# opt freq b3lyp/cc-pvtz empiricaldispersion=gd3bj pop=regular geom=connectivity int=ultrafine
SMILES	:	C[N]12CCC(CC1)(CC2)[C]3[N]O3
Formula	:	$C_9H_{15}N_2O^+$
Charge	:	1
Multiplicity	:	1
Energy	:	-536.59425916 a.u.
Gibbs Energy	:	-536.39187700 a.u.
ZPE (B3LYP)	:	0.238783 a.u.
CASPT2 Energy (incl. N_2 at $R = \infty$)	:	-644.6279031 a.u.
CCSD(T)-F12b Energy	:	-535.58494545 a.u.

Cartesian Co-ordinates (XYZ format)

27

C	-0.10462300	0.58930099	-1.14242601
C	0.22686701	-0.31165200	0.05454300
C	-1.63215697	0.67475700	-1.28118396
H	0.32547101	1.57800996	-0.99379498
H	0.32345101	0.18390800	-2.05701208
H	-1.97660995	1.69306803	-1.43801796
H	-2.01141596	0.05557300	-2.09032106
C	-0.21778999	0.39646000	1.35008502
H	-0.12085500	-0.28265101	2.19428706
H	0.41810599	1.25573301	1.55158806
C	-1.67157197	0.86062598	1.17750704
H	-2.28615499	0.62352699	2.04153395
H	-1.74574494	1.92805600	0.98512602
C	-2.05966496	-1.32032597	0.10437800
H	-2.42925096	-1.60840297	1.08540201
H	-2.67340398	-1.80451298	-0.65010399
C	-0.56648898	-1.62758803	-0.07486900
H	-0.24089199	-2.34066010	0.67899603
H	-0.37766600	-2.07470703	-1.04905295
N	-2.30069089	0.17114300	-0.01638300
C	-3.76518703	0.45405301	-0.06139100
H	-4.18108988	0.01912000	-0.96570301
H	-4.23667908	0.01402900	0.81266099
H	-3.91719007	1.52959394	-0.06301100
C	1.67360997	-0.61367500	0.12492900
O	2.35624599	-1.29911494	0.99858898

N 2.74231195 -0.40690801 -0.49342999

Frequencies

Mode	IR frequency	IR intensity	Raman intensity
1	50.29130000	0.06380000	0.00000000
2	110.92130000	0.10570000	0.00000000
3	124.77210000	3.22710000	0.00000000
4	130.18780000	3.98860000	0.00000000
5	260.44090000	0.00050000	0.00000000
6	265.15750000	0.25670000	0.00000000
7	268.34680000	0.22190000	0.00000000
8	313.41270000	7.21400000	0.00000000
9	329.15010000	2.47250000	0.00000000
10	342.82440000	1.16700000	0.00000000
11	421.16160000	0.04850000	0.00000000
12	422.10120000	0.02820000	0.00000000
13	498.11430000	0.92670000	0.00000000
14	510.47140000	3.03890000	0.00000000
15	537.75030000	0.09440000	0.00000000
16	540.30500000	0.09340000	0.00000000
17	615.15380000	6.09840000	0.00000000
18	634.84700000	3.07210000	0.00000000
19	705.64500000	0.83730000	0.00000000
20	803.81860000	0.07750000	0.00000000
21	836.72280000	8.08190000	0.00000000
22	838.73880000	8.68670000	0.00000000
23	863.60450000	12.69900000	0.00000000
24	928.68500000	2.65110000	0.00000000
25	931.91400000	4.17030000	0.00000000
26	990.01120000	0.34300000	0.00000000
27	999.56010000	1.99740000	0.00000000
28	1003.30120000	8.71330000	0.00000000
29	1011.05710000	1.78830000	0.00000000
30	1049.77700000	2.14510000	0.00000000
31	1056.72760000	1.24760000	0.00000000
32	1096.64140000	7.77940000	0.00000000
33	1149.19470000	0.44230000	0.00000000
34	1151.02650000	1.80350000	0.00000000
35	1155.98250000	14.00730000	0.00000000
36	1206.04370000	0.23400000	0.00000000
37	1209.21440000	6.92780000	0.00000000
38	1210.89730000	0.82650000	0.00000000
39	1277.90540000	16.73800000	0.00000000
40	1286.41860000	1.87780000	0.00000000
41	1304.55010000	7.02210000	0.00000000
42	1312.79500000	5.24790000	0.00000000
43	1337.13420000	3.55460000	0.00000000
44	1338.83000000	6.92980000	0.00000000
45	1358.41690000	3.24130000	0.00000000
46	1360.88980000	4.44460000	0.00000000
47	1393.28470000	2.19570000	0.00000000
48	1395.33350000	2.77900000	0.00000000
49	1407.18970000	5.16630000	0.00000000
50	1417.73550000	1.71270000	0.00000000
51	1476.56840000	2.38690000	0.00000000
52	1493.95780000	5.01240000	0.00000000
53	1494.24570000	4.83820000	0.00000000
54	1507.57840000	0.97960000	0.00000000
55	1508.19160000	0.88590000	0.00000000
56	1515.26640000	29.39250000	0.00000000
57	1515.61440000	28.79820000	0.00000000
58	1515.91050000	23.55150000	0.00000000
59	1540.11860000	3.66770000	0.00000000
60	1822.56540000	61.12210000	0.00000000

61	3070.85800000	0.03010000	0.00000000
62	3073.32880000	2.49680000	0.00000000
63	3075.42860000	2.71850000	0.00000000
64	3078.83630000	1.96180000	0.00000000
65	3086.12330000	1.75360000	0.00000000
66	3086.55170000	1.82290000	0.00000000
67	3090.55440000	8.34210000	0.00000000
68	3112.59520000	1.05090000	0.00000000
69	3117.21590000	0.70250000	0.00000000
70	3118.25640000	0.54780000	0.00000000
71	3138.29150000	0.07600000	0.00000000
72	3142.89800000	2.55720000	0.00000000
73	3143.10840000	2.05810000	0.00000000
74	3162.40460000	0.61910000	0.00000000
75	3162.52480000	0.65540000	0.00000000

S18. CALCULATIONS ON TS 13s ⟶ 14s

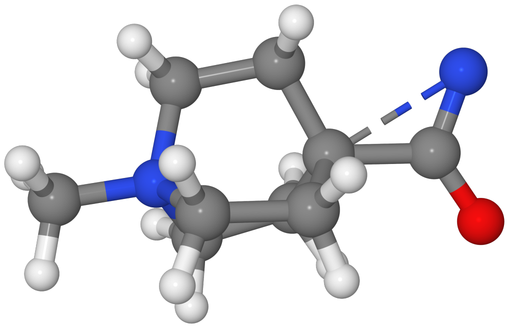

Route	:	# opt=(calcfc,qst3) freq b3lyp/cc-pvtz empiricaldispersion=gd3bj pop=regular geom=connectivity int=ultrafine
SMILES	:	C[N]12CCC(CC1)(CC2)C(=O)[N]
Formula	:	$C_9H_{15}N_2O^+$
Charge	:	1
Multiplicity	:	1
Energy	:	-536.56959732 a.u.
Gibbs Energy	:	-536.36912200 a.u.
ZPE (B3LYP)	:	0.236523 a.u.
CASPT2 Energy (incl. N_2 at $R = \infty$)	:	-644.57029557 a.u.
CCSD(T)-F12b Energy	:	-535.55099429 a.u.

Cartesian Co-ordinates (XYZ format)

27

C	0.22988801	1.53669202	0.06169200
C	0.94579703	0.17773800	0.01115800
C	-1.27291906	1.29058504	-0.16706000
H	0.39345399	2.01841998	1.02358902
H	0.60036403	2.20909309	-0.70872498
H	-1.89144301	1.83280206	0.54265600
H	-1.58694398	1.55909503	-1.17256200
C	0.52542597	-0.64828497	1.23719704
H	0.92799997	-1.65419805	1.15271294
H	0.92646801	-0.20949499	2.14833689
C	-1.01089597	-0.66470599	1.30765295
H	-1.40242004	-1.66333997	1.48151195
H	-1.39844704	-0.00224800	2.07757711
C	-0.94972301	-0.95424998	-1.13551199
H	-1.07272005	-2.00679708	-0.89288098
H	-1.51752400	-0.73721999	-2.03616691
C	0.52782100	-0.55385202	-1.27309799
H	1.14236498	-1.43932998	-1.41511703
H	0.67353499	0.09647700	-2.13358307
N	-1.59647298	-0.18071701	-0.00282600
C	-3.07399607	-0.38901600	-0.01492900
H	-3.50747108	0.10260400	0.85124803
H	-3.48314309	0.03643200	-0.92676800
H	-3.27947998	-1.45509100	0.02193300
C	2.51325989	0.18437800	0.00891500
O	3.20258188	-0.84502900	-0.09318600
N	2.96056294	1.36490703	0.14019400

Frequencies

Mode	IR frequency	IR intensity	Raman intensity
1	-947.40100000	56.14130000	0.00000000
2	49.71010000	0.76160000	0.00000000
3	114.58350000	0.00480000	0.00000000
4	140.92200000	1.51230000	0.00000000
5	163.31850000	2.25160000	0.00000000
6	260.79310000	0.00140000	0.00000000
7	266.98210000	0.29820000	0.00000000
8	268.72810000	0.17890000	0.00000000
9	315.88570000	5.92390000	0.00000000
10	330.51870000	1.02280000	0.00000000
11	358.56520000	0.71630000	0.00000000
12	420.87530000	0.02570000	0.00000000
13	421.94570000	0.16730000	0.00000000
14	484.43120000	3.40340000	0.00000000
15	533.35820000	0.10610000	0.00000000
16	539.73840000	0.07540000	0.00000000
17	579.73760000	3.09220000	0.00000000
18	688.83080000	4.71630000	0.00000000
19	699.22600000	0.03830000	0.00000000
20	805.64420000	0.36250000	0.00000000
21	828.19450000	6.38990000	0.00000000
22	837.84730000	8.09120000	0.00000000
23	842.54190000	8.36140000	0.00000000
24	928.82940000	2.88720000	0.00000000
25	931.43130000	4.04360000	0.00000000
26	959.71140000	19.81690000	0.00000000
27	988.42710000	1.23780000	0.00000000
28	991.09480000	0.65490000	0.00000000
29	997.26150000	3.24670000	0.00000000
30	1041.77030000	3.60350000	0.00000000
31	1045.09810000	2.44120000	0.00000000
32	1048.90090000	0.97580000	0.00000000
33	1132.31220000	10.84810000	0.00000000
34	1147.22070000	0.48450000	0.00000000
35	1150.03010000	1.24690000	0.00000000
36	1202.20200000	2.80740000	0.00000000
37	1206.05030000	1.06780000	0.00000000
38	1206.72840000	0.81710000	0.00000000
39	1267.22000000	10.42820000	0.00000000
40	1280.83190000	4.16850000	0.00000000
41	1304.66980000	8.66430000	0.00000000
42	1306.56140000	5.57930000	0.00000000
43	1334.26760000	2.28660000	0.00000000
44	1336.78150000	2.69950000	0.00000000
45	1354.48460000	5.67560000	0.00000000
46	1359.11690000	4.90430000	0.00000000
47	1387.93730000	14.52510000	0.00000000
48	1389.01610000	3.91240000	0.00000000
49	1392.17020000	3.50780000	0.00000000
50	1414.77230000	5.22250000	0.00000000
51	1475.29170000	4.43380000	0.00000000
52	1492.95960000	4.09440000	0.00000000
53	1493.81180000	3.64990000	0.00000000
54	1508.07030000	2.43420000	0.00000000
55	1508.33990000	2.92570000	0.00000000
56	1513.84850000	30.96470000	0.00000000
57	1515.02820000	35.45740000	0.00000000
58	1515.80000000	22.84950000	0.00000000
59	1527.18180000	40.49770000	0.00000000
60	1539.57060000	0.99750000	0.00000000

61	3070.70720000	0.02640000	0.00000000
62	3074.53960000	1.37130000	0.00000000
63	3074.87960000	3.29440000	0.00000000
64	3081.40590000	0.31530000	0.00000000
65	3085.37510000	2.06990000	0.00000000
66	3086.51070000	2.09920000	0.00000000
67	3090.21920000	9.46980000	0.00000000
68	3113.64120000	0.94950000	0.00000000
69	3121.45610000	1.33160000	0.00000000
70	3123.60980000	0.54300000	0.00000000
71	3139.31540000	0.00660000	0.00000000
72	3143.48950000	2.58110000	0.00000000
73	3144.58100000	1.58800000	0.00000000
74	3161.97800000	0.56960000	0.00000000
75	3162.55450000	0.64710000	0.00000000

S19. CALCULATIONS ON 14s

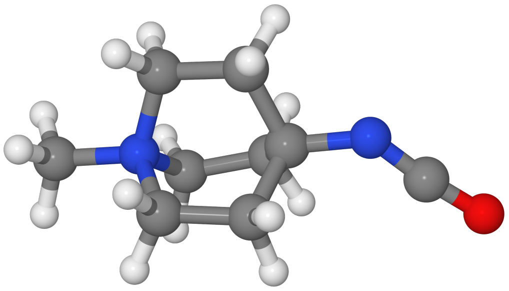

Route	:	# opt freq b3lyp/cc-pvtz empiricaldispersion=gd3bj pop=regular geom=connectivity int=ultrafine
SMILES	:	C[N]12CCC(CC1)(CC2)[N][C]=O
Formula	:	$C_9H_{15}N_2O^+$
Charge	:	1
Multiplicity	:	1
Energy	:	-536.72520272 a.u.
Gibbs Energy	:	-536.52141100 a.u.
ZPE (B3LYP)	:	0.240816 a.u.
CASPT2 Energy (incl. N_2 at $R = \infty$)	:	-644.75383496 a.u.
CCSD(T)-F12b Energy	:	-535.70627759 a.u.

Cartesian Co-ordinates (XYZ format)

27

C	-0.12979600	0.63141900	-1.11984706
C	0.23233899	-0.22705901	0.10441500
C	-1.65698195	0.67963302	-1.27566195
H	0.27785200	1.63375199	-0.99609798
H	0.31998500	0.20139600	-2.01150799
H	-2.02719903	1.68788600	-1.43994105
H	-2.01210904	0.04797000	-2.08609891
C	-0.26747799	0.49917001	1.36553895
H	-0.14088900	-0.14908400	2.23029804
H	0.32701001	1.39417005	1.53728199
C	-1.74170899	0.88936102	1.17890501
H	-2.35374808	0.62640703	2.03728390
H	-1.86867702	1.95071304	0.98016202
C	-2.02694106	-1.31591296	0.12113400
H	-2.41589093	-1.61233699	1.09225094
H	-2.60073495	-1.82677495	-0.64717698
C	-0.51809400	-1.56194305	-0.00895400
H	-0.18061000	-2.23384404	0.77620399
H	-0.27825299	-2.02960491	-0.96180600
N	-2.32479596	0.16384400	-0.01666600
C	-3.79760599	0.38885000	-0.08669600
H	-4.18317413	-0.07596900	-0.98955703
H	-4.26461506	-0.05564500	0.78749597
H	-3.99153090	1.45744097	-0.10781500
N	1.63945103	-0.49740401	0.16727801
C	2.63304591	0.18498300	0.26013100
O	3.65171504	0.73684901	0.34845200

Frequencies

Mode	IR frequency	IR intensity	Raman intensity
1	23.06730000	0.12930000	0.00000000
2	84.94760000	1.97130000	0.00000000
3	99.20280000	0.08910000	0.00000000
4	255.76790000	1.74460000	0.00000000
5	258.76530000	1.84740000	0.00000000
6	261.55120000	0.02090000	0.00000000
7	276.57150000	2.16420000	0.00000000
8	279.46720000	2.24990000	0.00000000
9	401.82910000	7.20670000	0.00000000
10	422.54260000	0.18090000	0.00000000
11	423.34630000	0.41520000	0.00000000
12	429.12910000	1.83210000	0.00000000
13	444.08630000	3.87950000	0.00000000
14	532.90260000	0.42950000	0.00000000
15	535.36080000	0.11590000	0.00000000
16	599.33930000	1.66240000	0.00000000
17	603.67010000	20.53070000	0.00000000
18	684.16120000	17.19340000	0.00000000
19	714.79240000	7.24990000	0.00000000
20	809.13070000	0.02140000	0.00000000
21	837.33730000	6.32400000	0.00000000
22	837.92200000	5.60050000	0.00000000
23	868.44830000	1.08320000	0.00000000
24	928.96040000	2.05010000	0.00000000
25	931.11090000	1.82600000	0.00000000
26	983.13950000	2.58830000	0.00000000
27	1006.10640000	9.49760000	0.00000000
28	1008.41620000	22.19290000	0.00000000
29	1010.39600000	15.60780000	0.00000000
30	1048.89540000	0.38620000	0.00000000
31	1052.55830000	2.49070000	0.00000000
32	1076.53360000	44.97090000	0.00000000
33	1140.82390000	1.84770000	0.00000000
34	1149.54280000	1.74650000	0.00000000
35	1151.96930000	3.09200000	0.00000000
36	1199.95290000	0.01690000	0.00000000
37	1217.05720000	3.48510000	0.00000000
38	1219.72630000	7.42090000	0.00000000
39	1278.16780000	4.33880000	0.00000000
40	1298.30660000	5.62950000	0.00000000
41	1303.49760000	6.93130000	0.00000000
42	1330.63980000	1.82710000	0.00000000
43	1333.40220000	1.00760000	0.00000000
44	1356.18080000	6.81320000	0.00000000
45	1356.52970000	6.77370000	0.00000000
46	1387.52920000	27.06140000	0.00000000
47	1395.61870000	0.89250000	0.00000000
48	1396.80400000	1.42100000	0.00000000
49	1417.18350000	2.60900000	0.00000000
50	1476.04960000	4.17600000	0.00000000
51	1488.74910000	56.31170000	0.00000000
52	1493.44100000	4.60770000	0.00000000
53	1493.81550000	4.80970000	0.00000000
54	1506.06750000	0.80760000	0.00000000
55	1506.77150000	0.61990000	0.00000000
56	1514.83410000	28.41420000	0.00000000
57	1515.37620000	29.42850000	0.00000000
58	1515.97290000	9.70490000	0.00000000
59	1540.86680000	13.20350000	0.00000000
60	2350.00110000	1267.16900000	0.00000000

61	3068.73890000	4.99180000	0.00000000
62	3070.69170000	0.09240000	0.00000000
63	3073.12140000	5.93330000	0.00000000
64	3078.32540000	5.70460000	0.00000000
65	3084.83090000	2.72160000	0.00000000
66	3084.95300000	2.28800000	0.00000000
67	3089.74690000	8.92170000	0.00000000
68	3113.04040000	1.54880000	0.00000000
69	3115.83980000	1.68580000	0.00000000
70	3119.79110000	1.33050000	0.00000000
71	3136.14230000	0.20310000	0.00000000
72	3141.01590000	3.99870000	0.00000000
73	3141.49710000	4.24950000	0.00000000
74	3161.76140000	0.96160000	0.00000000
75	3161.98790000	0.91760000	0.00000000

S20. CALCULATIONS ON TS 8 (ISOMER 1) ⟶ 14s

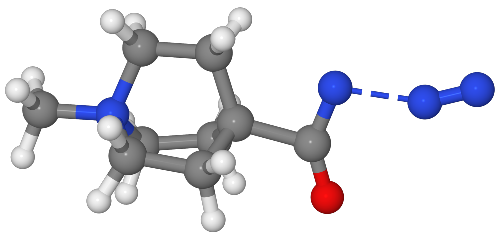

Route	:	# opt=(calcfc,ts,noeigentest) freq b3lyp/cc-pvtz empiricaldispersion=gd3bj pop=regular geom=connectivity int=ultrafine
SMILES	:	C[N]12CCC(CC1)(CC2)C(=O)[N].[N]=[N]
Formula	:	$C_9H_{15}N_4O^+$
Charge	:	1
Multiplicity	:	1
Energy	:	-646.16804409 a.u.
Gibbs Energy	:	-645.96232800 a.u.
ZPE (B3LYP)	:	0.245858 a.u.
CASPT2 Energy	:	-644.60562707 a.u.
CCSD(T)-F12b Energy	:	-644.97936476 a.u.

Cartesian Co-ordinates (XYZ format)

29

C	-0.18465200	-0.80513602	1.12808502
C	0.18794200	0.11472500	-0.04369800
C	-1.72029197	-0.83208102	1.25806403
H	0.20098400	-1.80721200	0.95883501
H	0.24427000	-0.44602099	2.05851793
H	-2.10686994	-1.84105897	1.37395406
H	-2.07553792	-0.23012801	2.09095597
C	-0.28644800	-0.54054701	-1.34790695
H	-0.14547700	0.15375499	-2.17193699
H	0.30439100	-1.42891002	-1.56110597
C	-1.76505804	-0.93251997	-1.19799900
H	-2.35906196	-0.62941200	-2.05609107
H	-1.90073705	-2.00018811	-1.04416895
C	-2.04272699	1.22752905	-0.04926500
H	-2.38688898	1.56838703	-1.02271700
H	-2.63773990	1.71537697	0.71796900
C	-0.53549302	1.45406699	0.14813399
H	-0.17465800	2.17819405	-0.57688802
H	-0.33243799	1.84434199	1.14384198
N	-2.36256003	-0.25258800	0.01614300
C	-3.83847094	-0.46044999	0.04912100
H	-4.23468590	-0.03604600	0.96714699
H	-4.28358412	0.03329100	-0.81008297
H	-4.04640198	-1.52600801	0.01335200
C	1.74721897	0.52283400	-0.34727201
O	2.00470400	1.28327096	-1.24849796
N	2.27942896	-0.19410001	0.57691199
N	4.09425688	0.11763100	0.37591299
N	5.15915489	0.02724500	0.60030800

Frequencies

Mode	IR frequency	IR intensity	Raman intensity
1	-481.06390000	247.34850000	0.00000000
2	35.89630000	2.29740000	0.00000000
3	62.91890000	1.57680000	0.00000000
4	80.02600000	0.09280000	0.00000000
5	116.59150000	0.06210000	0.00000000
6	159.75220000	0.17910000	0.00000000
7	170.01730000	0.58020000	0.00000000
8	239.68840000	2.21130000	0.00000000
9	259.91480000	21.55490000	0.00000000
10	261.00400000	1.11090000	0.00000000
11	267.54710000	7.42970000	0.00000000
12	273.61620000	6.71660000	0.00000000
13	301.17350000	2.27470000	0.00000000
14	314.04210000	2.60370000	0.00000000
15	398.53630000	3.18800000	0.00000000
16	406.40650000	1.82330000	0.00000000
17	423.45850000	0.02270000	0.00000000
18	423.56260000	0.02170000	0.00000000
19	535.78050000	0.03630000	0.00000000
20	542.13990000	0.27020000	0.00000000
21	558.22270000	2.41890000	0.00000000
22	694.12620000	0.73610000	0.00000000
23	717.82540000	5.49890000	0.00000000
24	739.87590000	11.18130000	0.00000000
25	808.24870000	0.05830000	0.00000000
26	833.86530000	7.56410000	0.00000000
27	839.81180000	7.37870000	0.00000000
28	842.79810000	8.58850000	0.00000000
29	930.06060000	3.35160000	0.00000000
30	930.95370000	3.35030000	0.00000000
31	953.72570000	20.67670000	0.00000000
32	992.16180000	0.16430000	0.00000000
33	999.18830000	3.82820000	0.00000000
34	1000.96070000	4.55250000	0.00000000
35	1038.60510000	4.12550000	0.00000000
36	1048.65310000	1.30920000	0.00000000
37	1051.96450000	1.58110000	0.00000000
38	1132.71610000	8.14130000	0.00000000
39	1148.77760000	2.16700000	0.00000000
40	1150.24230000	0.40810000	0.00000000
41	1189.64320000	11.46610000	0.00000000
42	1206.22590000	0.08240000	0.00000000
43	1210.64980000	0.70770000	0.00000000
44	1235.55520000	10.79490000	0.00000000
45	1279.08420000	4.56780000	0.00000000
46	1302.78910000	4.26370000	0.00000000
47	1305.02770000	7.32540000	0.00000000
48	1334.22240000	1.89310000	0.00000000
49	1335.24630000	2.66150000	0.00000000
50	1355.90600000	5.95480000	0.00000000
51	1357.20470000	5.06820000	0.00000000
52	1381.38520000	12.79230000	0.00000000
53	1391.57070000	1.66880000	0.00000000
54	1392.40620000	2.87200000	0.00000000
55	1414.41570000	6.89520000	0.00000000
56	1474.75280000	3.85730000	0.00000000
57	1493.80300000	4.27780000	0.00000000
58	1493.89630000	4.66110000	0.00000000
59	1505.51880000	1.14830000	0.00000000
60	1509.63710000	2.09410000	0.00000000

61	1514.05470000	26.08760000	0.00000000
62	1515.56840000	27.16020000	0.00000000
63	1516.48170000	25.13890000	0.00000000
64	1539.99730000	4.86550000	0.00000000
65	1867.02100000	302.40700000	0.00000000
66	2420.33620000	15.50600000	0.00000000
67	3070.08820000	0.01420000	0.00000000
68	3074.43080000	4.24480000	0.00000000
69	3079.95100000	0.55820000	0.00000000
70	3082.81200000	2.37120000	0.00000000
71	3084.11870000	4.63220000	0.00000000
72	3087.34640000	11.23600000	0.00000000
73	3090.42490000	4.48280000	0.00000000
74	3122.01410000	0.07120000	0.00000000
75	3125.33900000	2.55290000	0.00000000
76	3126.72370000	0.21990000	0.00000000
77	3138.70740000	0.16120000	0.00000000
78	3143.13690000	2.82300000	0.00000000
79	3144.56160000	4.51460000	0.00000000
80	3160.77300000	0.90260000	0.00000000
81	3161.61060000	1.09230000	0.00000000

S21. CALCULATIONS ON 8 (ISOMER 1; TRIPLET)

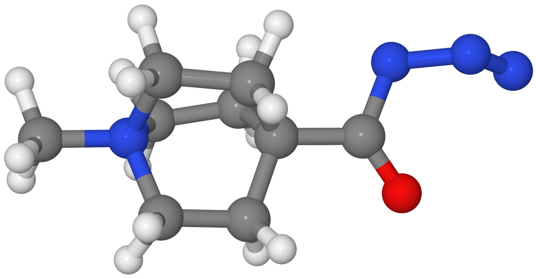

Route	:	# opt freq b3lyp/cc-pvtz empiricaldispersion=gd3bj pop=regular geom=connectivity int=ultrafine
SMILES	:	C[N]12CCC(CC1)(CC2)C(=O)N=[N+]=[N-]
Formula	:	$C_9H_{15}N_4O^{+,3}$
Charge	:	1
Multiplicity	:	3
Energy	:	-646.11934651 a.u.
Gibbs Energy	:	-645.91450900 a.u.
ZPE (B3LYP)	:	0.246116 a.u.
CASPT2 Energy	:	-644.56445301 a.u.

Cartesian Co-ordinates (XYZ format)

```
29

C   0.25597599 -0.64546901 -1.25442505
C  -0.19323701  0.20015600 -0.04418300
C   1.79118299 -0.65113997 -1.31267595
H  -0.13356601 -1.65656102 -1.16499197
H  -0.13736001 -0.22577700 -2.17811894
H   2.19328904 -1.64468896 -1.49164796
H   2.18075800  0.02019100 -2.07392502
C   0.22185799 -0.54690701  1.23784196
H   0.06006400  0.09172900  2.10493493
H  -0.38253999 -1.44107997  1.37274897
C   1.70168400 -0.94295198  1.12812304
H   2.25658989 -0.72248203  2.03587389
H   1.83338404 -1.99603403  0.89179599
C   2.03818297  1.30023801  0.16856800
H   2.33423209  1.56305099  1.18134797
H   2.67359209  1.84347796 -0.52552599
C   0.54516298  1.54272401 -0.09073500
H   0.14579999  2.22325206  0.65711498
H   0.39016399  2.01589489 -1.05782199
N   2.36328697 -0.17200001  0.00505100
C   3.83947992 -0.38211501  0.03422900
H   4.28297186  0.10982500 -0.82673001
H   4.23843384  0.04353100  0.95055401
H   4.04614401 -1.44797599 -0.00049700
C  -1.69763601  0.42464799 -0.08384700
O  -2.21356392  1.50566995 -0.23458500
N  -2.42406607 -0.78623003 -0.01671900
N  -3.73832107 -0.61895299  0.44159901
N  -4.61173677 -0.34686801 -0.29532000
```

Frequencies

Mode	IR frequency	IR intensity	Raman intensity
1	35.29900000	0.62600000	0.00000000
2	46.10960000	0.47730000	0.00000000
3	78.50210000	0.26990000	0.00000000
4	116.22340000	0.03480000	0.00000000
5	127.88870000	0.32070000	0.00000000
6	166.71550000	1.22890000	0.00000000
7	251.00620000	3.68060000	0.00000000
8	261.23430000	0.00140000	0.00000000
9	267.94650000	0.22170000	0.00000000
10	272.54760000	1.72810000	0.00000000
11	302.31430000	0.28640000	0.00000000
12	345.66170000	5.52980000	0.00000000
13	366.06140000	2.24800000	0.00000000
14	422.43320000	0.04650000	0.00000000
15	423.27000000	0.04140000	0.00000000
16	447.96380000	2.14600000	0.00000000
17	536.52620000	0.28320000	0.00000000
18	539.09520000	0.09040000	0.00000000
19	561.34010000	0.72260000	0.00000000
20	593.79600000	3.07910000	0.00000000
21	625.87370000	1.37920000	0.00000000
22	666.65080000	6.82430000	0.00000000
23	701.60170000	0.34140000	0.00000000
24	779.69350000	1.04390000	0.00000000
25	801.90660000	0.27330000	0.00000000
26	836.63270000	7.43640000	0.00000000
27	840.20540000	7.99120000	0.00000000
28	863.06240000	8.67170000	0.00000000
29	929.43020000	4.95370000	0.00000000
30	931.92820000	4.37440000	0.00000000
31	962.88290000	86.67990000	0.00000000
32	989.74550000	2.05960000	0.00000000
33	1000.89350000	2.27820000	0.00000000
34	1009.06220000	3.54510000	0.00000000
35	1047.62710000	3.01650000	0.00000000
36	1051.78970000	32.65820000	0.00000000
37	1058.29380000	1.38320000	0.00000000
38	1117.44210000	7.69860000	0.00000000
39	1148.62780000	0.59690000	0.00000000
40	1153.11520000	3.76890000	0.00000000
41	1188.70450000	107.25620000	0.00000000
42	1206.39240000	4.25740000	0.00000000
43	1209.31410000	0.88200000	0.00000000
44	1228.85430000	39.73840000	0.00000000
45	1284.04970000	0.01040000	0.00000000
46	1298.73880000	6.04780000	0.00000000
47	1307.59790000	4.20360000	0.00000000
48	1333.96470000	3.32390000	0.00000000
49	1336.80750000	2.41530000	0.00000000
50	1356.84140000	4.40770000	0.00000000
51	1360.81170000	3.77320000	0.00000000
52	1392.38650000	2.14620000	0.00000000
53	1393.93600000	2.38500000	0.00000000
54	1399.70320000	0.92590000	0.00000000
55	1416.43170000	3.12110000	0.00000000
56	1475.69770000	2.34160000	0.00000000
57	1493.89440000	5.08700000	0.00000000
58	1494.19230000	4.75860000	0.00000000
59	1504.50140000	1.64350000	0.00000000
60	1507.94620000	1.07210000	0.00000000

61	1514.06380000	22.84370000	0.00000000
62	1515.06250000	26.85130000	0.00000000
63	1515.82950000	26.30820000	0.00000000
64	1539.48360000	5.05450000	0.00000000
65	1680.70110000	80.79460000	0.00000000
66	1749.59200000	9.17550000	0.00000000
67	3068.18510000	4.85570000	0.00000000
68	3070.42480000	0.14070000	0.00000000
69	3077.47860000	2.50490000	0.00000000
70	3082.25500000	2.95930000	0.00000000
71	3084.79300000	2.85230000	0.00000000
72	3084.85710000	3.36780000	0.00000000
73	3089.06050000	9.06160000	0.00000000
74	3111.42510000	0.73470000	0.00000000
75	3117.55090000	0.74580000	0.00000000
76	3120.11180000	0.78540000	0.00000000
77	3136.48740000	0.18870000	0.00000000
78	3141.06430000	3.42030000	0.00000000
79	3141.75800000	3.36320000	0.00000000
80	3161.46510000	0.85210000	0.00000000
81	3161.75160000	0.83260000	0.00000000

S22. CALCULATIONS ON TS 8 (ISOMER 1; TRIPLET) ⟶ 13t

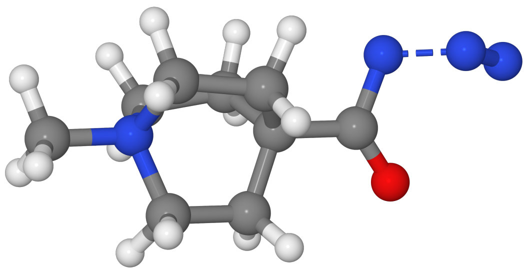

Route	:	# opt=(calcall,ts,noeigentest) freq ub3lyp/cc-pvtz empiricaldispersion=gd3bj pop=regular geom=connectivity int=ultrafine
SMILES	:	C[N]12CCC(CC1)(CC2)C(=O)N=[N+]=[N-]
Formula	:	$C_9H_{15}N_4O^{+,3}$
Charge	:	1
Multiplicity	:	3
Energy	:	-646.11909804 a.u.
Gibbs Energy	:	-645.91545500 a.u.
ZPE (B3LYP)	:	0.246116 a.u.
CASPT2 Energy	:	-644.56259419 a.u.

Cartesian Co-ordinates (XYZ format)

29

C	0.35704499	-0.43518201	-1.37809598
C	-0.10837800	0.20010300	-0.05085800
C	1.89268696	-0.41910401	-1.42310798
H	-0.02362800	-1.45060694	-1.45713305
H	-0.03362900	0.12541400	-2.22490597
H	2.30541897	-1.36688101	-1.75813305
H	2.28162003	0.37052199	-2.06122804
C	0.30326101	-0.74166900	1.09697199
H	0.12997700	-0.25315899	2.05468607
H	-0.29443699	-1.65051198	1.08108604
C	1.78697598	-1.10475194	0.93709701
H	2.33334804	-1.03122401	1.87337804
H	1.92902505	-2.10437202	0.53366899
C	2.11125207	1.26703501	0.35804799
H	2.39564610	1.36316001	1.40318298
H	2.74826097	1.92062199	-0.23159499
C	0.61863601	1.53797400	0.12601300
H	0.20723400	2.08697200	0.96942699
H	0.46792099	2.15901208	-0.75409597
N	2.45045304	-0.15665700	-0.03983800
C	3.92813396	-0.35786900	-0.03187500
H	4.37390184	0.27063301	-0.79752201
H	4.31633711	-0.08375700	0.94492698
H	4.14428377	-1.40239799	-0.23734701
C	-1.61589706	0.41682801	-0.06533700
O	-2.13777995	1.50739598	-0.06179300
N	-2.33964300	-0.78384000	-0.19371600
N	-3.66032791	-0.70221001	0.47339499
N	-4.58680582	-0.30897200	-0.09904500

Frequencies

Mode	IR frequency	IR intensity	Raman intensity
1	-358.83900000	15.85070000	0.00000000
2	37.72910000	0.62210000	0.00000000
3	42.78510000	0.45470000	0.00000000
4	73.64340000	0.23460000	0.00000000
5	118.23670000	0.04770000	0.00000000
6	137.79600000	0.73710000	0.00000000
7	171.00740000	1.45450000	0.00000000
8	261.01610000	0.00580000	0.00000000
9	265.14890000	0.68330000	0.00000000
10	268.43320000	0.25240000	0.00000000
11	279.64190000	2.44950000	0.00000000
12	297.30660000	7.46680000	0.00000000
13	343.41040000	6.00260000	0.00000000
14	365.14580000	2.57480000	0.00000000
15	422.26570000	0.10840000	0.00000000
16	422.86930000	0.04320000	0.00000000
17	470.28720000	1.43140000	0.00000000
18	536.70020000	0.17700000	0.00000000
19	538.95330000	0.08070000	0.00000000
20	555.34030000	1.22650000	0.00000000
21	595.68750000	1.78120000	0.00000000
22	649.67270000	7.51050000	0.00000000
23	701.46800000	0.45090000	0.00000000
24	777.02300000	1.28760000	0.00000000
25	802.01580000	0.25690000	0.00000000
26	836.50210000	7.22480000	0.00000000
27	840.37550000	7.83090000	0.00000000
28	861.80410000	9.03930000	0.00000000
29	929.61050000	5.35730000	0.00000000
30	932.24180000	4.25100000	0.00000000
31	962.62510000	81.85130000	0.00000000
32	989.57990000	1.65640000	0.00000000
33	1001.33700000	2.73370000	0.00000000
34	1007.95520000	3.26660000	0.00000000
35	1047.37020000	2.78480000	0.00000000
36	1053.16990000	31.56080000	0.00000000
37	1057.97730000	2.38600000	0.00000000
38	1116.62190000	8.76960000	0.00000000
39	1148.56020000	0.65010000	0.00000000
40	1152.80110000	4.46810000	0.00000000
41	1185.11070000	104.30320000	0.00000000
42	1206.21090000	2.61450000	0.00000000
43	1209.47170000	0.85990000	0.00000000
44	1228.53040000	35.39510000	0.00000000
45	1283.89380000	0.03070000	0.00000000
46	1298.73010000	6.33580000	0.00000000
47	1307.60230000	4.42980000	0.00000000
48	1333.97720000	3.44770000	0.00000000
49	1336.91020000	2.41800000	0.00000000
50	1357.14270000	4.41890000	0.00000000
51	1360.90390000	3.73970000	0.00000000
52	1392.34010000	2.08860000	0.00000000
53	1393.73450000	2.62110000	0.00000000
54	1399.21410000	1.01380000	0.00000000
55	1416.31310000	3.26570000	0.00000000
56	1475.53960000	2.36950000	0.00000000
57	1493.75920000	5.18080000	0.00000000
58	1494.04170000	4.60580000	0.00000000
59	1504.47690000	1.63500000	0.00000000
60	1507.84930000	1.04720000	0.00000000

61	1513.98290000	22.83430000	0.00000000
62	1514.94700000	26.28960000	0.00000000
63	1515.52760000	27.10750000	0.00000000
64	1539.07600000	4.98390000	0.00000000
65	1668.34680000	71.80560000	0.00000000
66	1810.29140000	9.53160000	0.00000000
67	3067.56970000	4.76480000	0.00000000
68	3070.35470000	0.09540000	0.00000000
69	3077.38410000	2.55780000	0.00000000
70	3082.40530000	2.79870000	0.00000000
71	3084.71230000	2.67510000	0.00000000
72	3084.98260000	3.51240000	0.00000000
73	3089.01620000	9.01410000	0.00000000
74	3109.54550000	0.87940000	0.00000000
75	3117.18720000	0.80920000	0.00000000
76	3120.06900000	0.78490000	0.00000000
77	3136.43910000	0.19490000	0.00000000
78	3141.02060000	3.31480000	0.00000000
79	3141.68430000	3.44660000	0.00000000
80	3161.48240000	0.84680000	0.00000000
81	3161.64510000	0.83440000	0.00000000

S23. CALCULATIONS ON 13t

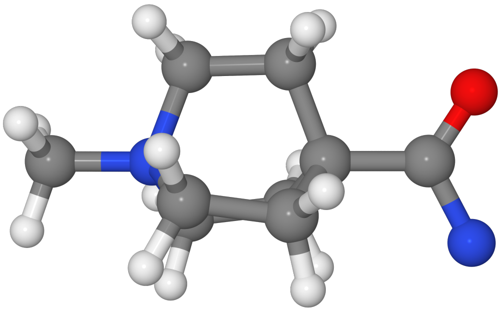

Route	:	# opt freq b3lyp/cc-pvtz empiricaldispersion=gd3bj pop=regular geom=connectivity int=ultrafine
SMILES	:	C[N]12CCC(CC1)(CC2)C(=O)[N]
Formula	:	$C_9H_{15}N_2O^{+,3}$
Charge	:	1
Multiplicity	:	3
Energy	:	-536.60260809 a.u.
Gibbs Energy	:	-536.40243800 a.u.
ZPE (B3LYP)	:	0.237727 a.u.
CASPT2 Energy (incl. N_2 at $R = \infty$)	:	-644.63265162 a.u.

Cartesian Co-ordinates (XYZ format)

27

C	-0.25977600	-1.45944595	-0.00139600
C	-0.82657498	-0.02511800	-0.01626300
C	1.26063097	-1.39427698	-0.21450099
H	-0.49558899	-1.94616604	0.94304198
H	-0.70806998	-2.05570197	-0.79371500
H	1.80310702	-2.02984691	0.48013899
H	1.54901695	-1.66632903	-1.22680104
C	-0.30397901	0.70574301	1.23700500
H	-0.56343001	1.76167500	1.18046904
H	-0.76539201	0.30569899	2.13737392
C	1.21939600	0.52681601	1.32198703
H	1.73342395	1.45925403	1.53848398
H	1.51044798	-0.20763500	2.06903601
C	1.21697497	0.89960802	-1.11255705
H	1.47584295	1.92031300	-0.84140801
H	1.76140404	0.63665199	-2.01537108
C	-0.29716599	0.69567502	-1.26073694
H	-0.79103398	1.65626800	-1.38225603
H	-0.52410698	0.11261700	-2.15104389
N	1.75632906	0.02066400	-0.00026700
C	3.24767590	0.04938600	0.00357900
H	3.61150599	-0.39294899	-0.91940600
H	3.58036995	1.08086395	0.07656300
H	3.61059809	-0.51862001	0.85540700
C	-2.35189700	-0.05180800	-0.01989300
O	-3.03463602	0.47976601	-0.88909698
N	-2.97782612	-0.70917100	1.00706995

Frequencies

Mode	IR frequency	IR intensity	Raman intensity
1	31.41450000	0.22730000	0.00000000
2	110.44620000	0.04390000	0.00000000
3	154.62510000	2.17700000	0.00000000
4	157.95110000	0.81120000	0.00000000
5	260.33550000	0.00070000	0.00000000
6	266.03550000	0.21500000	0.00000000
7	268.52590000	0.14640000	0.00000000
8	307.81600000	7.97930000	0.00000000
9	336.94750000	4.03040000	0.00000000
10	353.83000000	1.48200000	0.00000000
11	422.03430000	0.05000000	0.00000000
12	422.96200000	0.06920000	0.00000000
13	461.22890000	3.70230000	0.00000000
14	531.33640000	4.46120000	0.00000000
15	538.99110000	0.04010000	0.00000000
16	548.97180000	3.19810000	0.00000000
17	622.62210000	22.92260000	0.00000000
18	659.37840000	10.16730000	0.00000000
19	705.40560000	2.58720000	0.00000000
20	801.97470000	0.11110000	0.00000000
21	835.83740000	8.47500000	0.00000000
22	838.68730000	8.53230000	0.00000000
23	859.71300000	16.46050000	0.00000000
24	929.08010000	4.43040000	0.00000000
25	931.69170000	4.74460000	0.00000000
26	980.59040000	8.48300000	0.00000000
27	988.76340000	1.01150000	0.00000000
28	996.72190000	1.20980000	0.00000000
29	1003.34150000	1.82490000	0.00000000
30	1047.47180000	2.29440000	0.00000000
31	1056.07540000	2.36180000	0.00000000
32	1068.41040000	5.78110000	0.00000000
33	1116.64650000	0.47930000	0.00000000
34	1148.44270000	0.54230000	0.00000000
35	1150.65810000	12.08690000	0.00000000
36	1173.11500000	39.74910000	0.00000000
37	1206.07820000	0.28120000	0.00000000
38	1208.60080000	0.58590000	0.00000000
39	1230.57420000	11.62320000	0.00000000
40	1283.11940000	1.23670000	0.00000000
41	1298.91060000	4.58950000	0.00000000
42	1310.93050000	4.46340000	0.00000000
43	1334.95870000	2.93450000	0.00000000
44	1337.96810000	2.64840000	0.00000000
45	1358.16180000	4.27860000	0.00000000
46	1360.19370000	4.16230000	0.00000000
47	1392.53630000	2.36200000	0.00000000
48	1393.64990000	2.13410000	0.00000000
49	1399.73020000	0.60990000	0.00000000
50	1416.46510000	3.64240000	0.00000000
51	1475.74600000	3.00610000	0.00000000
52	1493.62550000	4.85320000	0.00000000
53	1493.89270000	5.17630000	0.00000000
54	1504.43130000	1.37250000	0.00000000
55	1507.07150000	0.94150000	0.00000000
56	1513.91480000	21.92280000	0.00000000
57	1514.93910000	28.34910000	0.00000000
58	1515.29590000	26.00100000	0.00000000
59	1537.68550000	62.91780000	0.00000000
60	1540.51070000	33.55020000	0.00000000

61	3069.24090000	3.80700000	0.00000000
62	3070.33500000	0.74750000	0.00000000
63	3071.44160000	2.79100000	0.00000000
64	3080.24490000	2.66310000	0.00000000
65	3084.45890000	2.43460000	0.00000000
66	3084.75150000	2.16120000	0.00000000
67	3088.94350000	8.89520000	0.00000000
68	3109.92420000	1.16430000	0.00000000
69	3110.89310000	1.17760000	0.00000000
70	3120.68130000	0.71870000	0.00000000
71	3136.34650000	0.09970000	0.00000000
72	3140.90370000	2.60950000	0.00000000
73	3141.45020000	3.52510000	0.00000000
74	3161.69780000	0.78020000	0.00000000
75	3161.86660000	0.78420000	0.00000000

S24. CALCULATIONS ON TS 13t ⟶ 14t

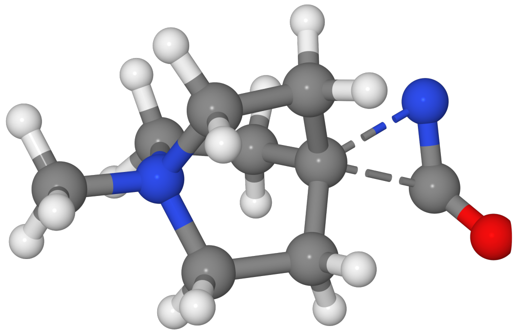

Route	:	# opt=(calcfc,ts,noeigentest) freq ub3lyp/cc-pvtz empiricaldispersion=gd3bj pop=regular geom=connectivity int=ultrafine
SMILES	:	C[N]12CC[C](CC1)CC2.[C](=O)[N]
Formula	:	$C_9H_{15}N_2O^{+,3}$
Charge	:	1
Multiplicity	:	3
Energy	:	-536.51772864 a.u.
Gibbs Energy	:	-536.31978800 a.u.
ZPE (B3LYP)	:	0.235187 a.u.
CASPT2 Energy (incl. N_2 at $R = \infty$)	:	-644.55072081 a.u.

Cartesian Co-ordinates (XYZ format)

27

C	0.19642501	-0.97582698	-1.15289295
C	0.80278498	-0.19561900	0.01083200
C	-1.29449403	-0.59616297	-1.26219702
H	0.30727699	-2.04267907	-0.98321497
H	0.70205897	-0.73804998	-2.08503199
H	-1.49656904	0.09295200	-2.07839704
H	-1.93024194	-1.46929705	-1.38116598
C	0.25107700	-0.69054800	1.33032095
H	0.55696201	-0.03310800	2.14041805
H	0.61185300	-1.69074297	1.55794001
C	-1.28854597	-0.71811700	1.20096099
H	-1.67384303	-1.72060704	1.03351605
H	-1.77750802	-0.29986501	2.07631111
C	-1.04080105	1.45545900	0.05329600
H	-1.50445294	2.07826090	-0.70695901
H	-1.26061904	1.87809896	1.03039706
C	0.47949699	1.28879595	-0.17354999
H	0.76273400	1.61697102	-1.17066801
H	1.01137698	1.91189897	0.53758901
N	-1.73027098	0.10816800	0.00560500
C	-3.21178007	0.28348699	0.02158100
H	-3.48934007	0.86687702	0.89487797
H	-3.68204188	-0.69478798	0.06363300
H	-3.51526403	0.80220199	-0.88326597
C	2.77098298	0.09331100	0.27905700
O	3.41888404	0.95021600	-0.26224300
N	2.54382801	-1.16545105	-0.08583800

Frequencies

Mode	IR frequency	IR intensity	Raman intensity
1	-866.77750000	136.62060000	0.00000000
2	49.56460000	0.15630000	0.00000000
3	115.69840000	0.04770000	0.00000000
4	143.52920000	1.41540000	0.00000000
5	205.28760000	0.73170000	0.00000000
6	219.36150000	1.73830000	0.00000000
7	261.84380000	0.00330000	0.00000000
8	264.08780000	0.03760000	0.00000000
9	274.84200000	0.25740000	0.00000000
10	322.06490000	0.35260000	0.00000000
11	376.31940000	0.53470000	0.00000000
12	406.70900000	0.30640000	0.00000000
13	420.24450000	0.04730000	0.00000000
14	426.24980000	0.07110000	0.00000000
15	518.26710000	9.88600000	0.00000000
16	531.64940000	1.58870000	0.00000000
17	533.28990000	4.27640000	0.00000000
18	552.68280000	14.64780000	0.00000000
19	685.76580000	1.92730000	0.00000000
20	772.90140000	4.85000000	0.00000000
21	805.53080000	0.03560000	0.00000000
22	835.36090000	6.50170000	0.00000000
23	841.96680000	7.42740000	0.00000000
24	916.74820000	21.20650000	0.00000000
25	923.19300000	10.97900000	0.00000000
26	926.74020000	7.90840000	0.00000000
27	978.76890000	18.68500000	0.00000000
28	988.16060000	1.39950000	0.00000000
29	991.21370000	5.16360000	0.00000000
30	1008.98920000	2.45940000	0.00000000
31	1030.82380000	2.53970000	0.00000000
32	1055.54810000	0.23540000	0.00000000
33	1127.39550000	8.87670000	0.00000000
34	1138.25740000	9.00990000	0.00000000
35	1152.56930000	0.98400000	0.00000000
36	1168.90940000	9.93300000	0.00000000
37	1202.33940000	0.18660000	0.00000000
38	1211.04870000	0.26860000	0.00000000
39	1216.36900000	1.75570000	0.00000000
40	1269.85050000	7.79330000	0.00000000
41	1285.78100000	6.85670000	0.00000000
42	1299.86910000	9.61460000	0.00000000
43	1325.52460000	1.36140000	0.00000000
44	1333.81000000	3.13850000	0.00000000
45	1352.53520000	6.24270000	0.00000000
46	1356.65600000	5.41120000	0.00000000
47	1361.98350000	10.51010000	0.00000000
48	1387.54540000	3.19040000	0.00000000
49	1390.32670000	1.03500000	0.00000000
50	1410.01030000	10.42420000	0.00000000
51	1473.78380000	4.66890000	0.00000000
52	1491.83500000	3.13680000	0.00000000
53	1492.80000000	4.44260000	0.00000000
54	1503.07390000	1.73900000	0.00000000
55	1504.82140000	1.41120000	0.00000000
56	1512.03280000	27.51470000	0.00000000
57	1512.42860000	28.08590000	0.00000000
58	1514.01760000	31.08990000	0.00000000
59	1537.34250000	3.89850000	0.00000000
60	1576.38470000	141.93500000	0.00000000

61	3070.51310000	0.01650000	0.00000000
62	3081.21660000	0.76800000	0.00000000
63	3083.99570000	1.52360000	0.00000000
64	3085.60130000	2.66820000	0.00000000
65	3089.21460000	7.89990000	0.00000000
66	3091.39490000	1.34370000	0.00000000
67	3093.39810000	4.40880000	0.00000000
68	3122.40190000	0.43820000	0.00000000
69	3128.61100000	0.06160000	0.00000000
70	3132.48420000	0.42620000	0.00000000
71	3142.99240000	0.50430000	0.00000000
72	3146.96090000	2.65690000	0.00000000
73	3149.88440000	1.74980000	0.00000000
74	3161.78200000	0.75870000	0.00000000
75	3162.16900000	0.68400000	0.00000000

S25. CALCULATIONS ON 14t

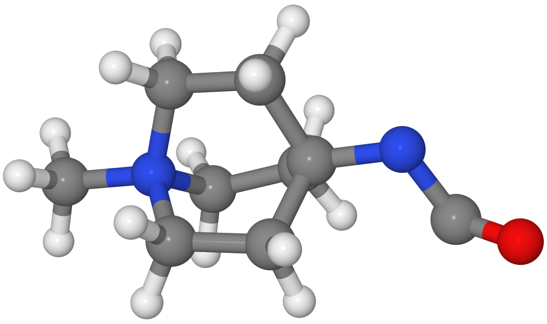

Route	:	# opt freq b3lyp/cc-pvtz empiricaldispersion=gd3bj pop=regular geom=connectivity int=ultrafine
SMILES	:	C[N]12CCC(CC1)(CC2)[N][C]=O
Formula	:	$C_9H_{15}N_2O^{+,3}$
Charge	:	1
Multiplicity	:	3
Energy	:	-536.59727988 a.u.
Gibbs Energy	:	-536.39793400 a.u.
ZPE (B3LYP)	:	0.237055 a.u.
CASPT2 Energy (incl. N_2 at $R = \infty$)	:	-644.62395321 a.u.

Cartesian Co-ordinates (XYZ format)

```
27

C   0.34840599 -0.49407601  1.19762397
C   0.71903998  0.33823299 -0.04960900
C  -1.18053806 -0.55584198  1.31656301
H   0.77600902 -1.49099505  1.10975099
H   0.76789802 -0.03286900  2.08902788
H  -1.53416395 -1.55664802  1.54912698
H  -1.56743503  0.12911700  2.06684804
C   0.29190600 -0.47016799 -1.30133903
H   0.39646599  0.15532400 -2.18513107
H   0.94127101 -1.33298397 -1.42500806
C  -1.16154397 -0.92816198 -1.11714804
H  -1.75863695 -0.76584601 -2.01035309
H  -1.23216701 -1.97744095 -0.84113902
C  -1.57484305  1.32828701 -0.21962699
H  -1.91618896  1.54147696 -1.22972298
H  -2.21209693  1.86516905  0.47753900
C  -0.08654900  1.64416397 -0.02080100
H   0.26347101  2.30582905 -0.80925900
H   0.08093300  2.15333700  0.92641902
N  -1.82078898 -0.15123799  0.00418000
C  -3.28526402 -0.43360001  0.03596100
H  -3.72175789  0.06403100  0.89724201
H  -3.73688102 -0.05774700 -0.87767297
H  -3.43721700 -1.50657594  0.11096900
N   2.11961603  0.65239602 -0.11201100
C   2.99095511 -0.43442801 -0.06150800
O   4.04748487 -0.39706701  0.47950500
```

Frequencies

Mode	IR frequency	IR intensity	Raman intensity
1	60.40690000	0.58740000	0.00000000
2	79.66450000	1.49430000	0.00000000
3	123.99050000	0.00920000	0.00000000
4	153.76820000	0.49290000	0.00000000
5	229.38760000	1.79620000	0.00000000
6	262.24020000	0.00030000	0.00000000
7	266.37310000	0.10840000	0.00000000
8	273.55870000	0.37360000	0.00000000
9	308.66010000	1.35180000	0.00000000
10	369.56700000	2.19830000	0.00000000
11	404.51340000	2.32720000	0.00000000
12	421.43360000	0.08750000	0.00000000
13	423.12800000	0.01630000	0.00000000
14	483.09940000	1.14390000	0.00000000
15	531.05110000	0.12950000	0.00000000
16	533.50470000	0.12800000	0.00000000
17	566.73540000	1.20610000	0.00000000
18	687.57200000	1.36050000	0.00000000
19	745.47340000	5.18660000	0.00000000
20	801.22450000	0.18060000	0.00000000
21	834.21010000	5.78330000	0.00000000
22	837.48030000	6.18130000	0.00000000
23	856.02210000	26.94740000	0.00000000
24	903.17400000	5.68820000	0.00000000
25	925.27640000	1.34990000	0.00000000
26	929.53940000	1.97810000	0.00000000
27	969.38840000	6.45030000	0.00000000
28	985.67210000	0.27920000	0.00000000
29	1008.89900000	10.93720000	0.00000000
30	1031.91370000	1.33530000	0.00000000
31	1040.85910000	2.30890000	0.00000000
32	1053.55860000	0.89220000	0.00000000
33	1102.21200000	14.21660000	0.00000000
34	1141.33120000	1.99650000	0.00000000
35	1147.14530000	6.39760000	0.00000000
36	1166.73850000	5.06770000	0.00000000
37	1198.99010000	0.96430000	0.00000000
38	1201.48100000	0.05370000	0.00000000
39	1220.08620000	6.93300000	0.00000000
40	1279.28290000	3.98080000	0.00000000
41	1286.28990000	4.81320000	0.00000000
42	1290.51460000	11.60610000	0.00000000
43	1328.16630000	2.18360000	0.00000000
44	1329.05070000	2.71710000	0.00000000
45	1351.28830000	3.46290000	0.00000000
46	1354.43150000	4.86430000	0.00000000
47	1378.26870000	9.09930000	0.00000000
48	1390.29960000	2.42990000	0.00000000
49	1395.67390000	4.13010000	0.00000000
50	1415.54660000	4.06330000	0.00000000
51	1475.56970000	2.81100000	0.00000000
52	1493.59090000	4.34260000	0.00000000
53	1493.93840000	5.07560000	0.00000000
54	1505.92360000	1.55230000	0.00000000
55	1506.83670000	1.71750000	0.00000000
56	1513.60880000	21.85860000	0.00000000
57	1514.57580000	26.52890000	0.00000000
58	1515.23990000	27.31140000	0.00000000
59	1539.28830000	5.27310000	0.00000000
60	1753.27950000	36.76180000	0.00000000

61	3070.42860000	0.00770000	0.00000000
62	3073.46730000	2.04160000	0.00000000
63	3077.28220000	3.34150000	0.00000000
64	3079.45730000	2.28380000	0.00000000
65	3084.83870000	2.12800000	0.00000000
66	3085.48880000	2.17540000	0.00000000
67	3089.61580000	8.95340000	0.00000000
68	3116.02940000	1.09110000	0.00000000
69	3120.05420000	0.81850000	0.00000000
70	3122.23440000	0.37090000	0.00000000
71	3137.61580000	0.01440000	0.00000000
72	3142.21500000	3.09120000	0.00000000
73	3142.77850000	2.78460000	0.00000000
74	3161.73620000	0.76510000	0.00000000
75	3161.91750000	0.71610000	0.00000000

S26. CALCULATIONS ON CH_3

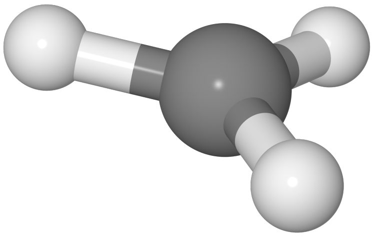

Route	:	# opt freq b3lyp/cc-pvtz empiricaldispersion=gd3bj pop=regular geom=connectivity int=ultrafine	
SMILES	:	[CH3]	
Formula	:	CH_3^2	
Charge	:	0	
Multiplicity	:	2	
Dipole	:	0.0005	Debye
Energy	:	-39.86005873	a.u.
Gibbs Energy	:	-39.84912900	a.u.
ZPE (B3LYP)	:	0.029672	a.u.
CCSD(T)-F12b Energy + ZPE	:	-39.73983973	a.u.

Cartesian Co-ordinates (XYZ format)

```
4

C  0.00000000  0.00000000 0.00007900
H -0.00000000  1.07770097 0.00000000
H -0.93331599 -0.53885001 0.00000000
H  0.93331599 -0.53885001 0.00000000
```

Frequencies

Mode	IR frequency	IR intensity	Raman intensity
1	523.99310000	76.20420000	0.00000000
2	1406.94920000	2.93150000	0.00000000

S27. CALCULATIONS ON 14s – CH_3

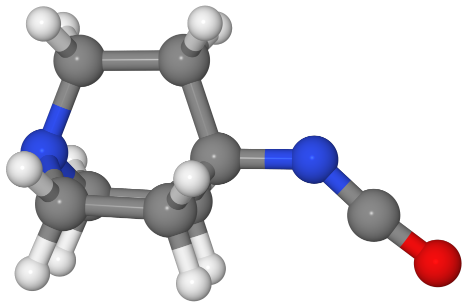

Route	:	# opt freq b3lyp/cc-pvtz empiricaldispersion=gd3bj pop=regular geom=connectivity int=ultrafine
SMILES	:	C1CN2CCC1(CC2)[N][C]=O
Formula	:	$C_8H_{12}N_2O^{+,2}$
Charge	:	1
Multiplicity	:	2
Energy	:	-496.72336202 a.u.
Gibbs Energy	:	-496.56301300 a.u.
ZPE (B3LYP)	:	0.197635 a.u.
CCSD(T)-F12b Energy + ZPE	:	-495.78251917 a.u.

Cartesian Co-ordinates (XYZ format)

23

C	-0.01601700	-0.54543197	1.25666296
C	0.33305600	0.27985001	-0.00020200
C	-1.55780196	-0.89530599	1.22293603
H	0.56151801	-1.46692896	1.27845001
H	0.20924900	0.02452500	2.15429807
H	-1.72452605	-1.96769094	1.18633103
H	-2.08504510	-0.46521500	2.06912398
C	-0.01454200	-0.55261701	-1.25271201
H	0.21075000	0.01262200	-2.15332294
H	0.56377703	-1.47373402	-1.26915205
C	-1.55600703	-0.90379298	-1.21788204
H	-2.08283710	-0.48051199	-2.06775999
H	-1.72171295	-1.97603703	-1.17349195
C	-2.05240107	1.15405500	-0.00498400
H	-2.56185699	1.50705600	-0.89658701
H	-2.56421494	1.51330900	0.88276798
C	-0.52604902	1.55997300	-0.00436900
H	-0.30499101	2.15441394	-0.88689601
H	-0.30678800	2.15891004	0.87556499
N	-2.04016900	-0.29217300	0.00006100
N	1.71235394	0.67267603	-0.00037200
C	2.76476407	0.07471000	0.00047000
O	3.82882094	-0.38825199	0.00106600

Frequencies

Mode	IR frequency	IR intensity	Raman intensity
1	22.97950000	0.09300000	0.00000000
2	32.94960000	0.02110000	0.00000000
3	91.69460000	2.23980000	0.00000000
4	261.44320000	0.20640000	0.00000000
5	268.41070000	2.70820000	0.00000000
6	361.51540000	8.34500000	0.00000000
7	367.26820000	3.30110000	0.00000000
8	427.69270000	1.55300000	0.00000000
9	428.55680000	0.39320000	0.00000000
10	461.84200000	1.62380000	0.00000000
11	490.53980000	1.34170000	0.00000000
12	493.22980000	2.96340000	0.00000000
13	602.33180000	22.95590000	0.00000000
14	654.53410000	20.44180000	0.00000000
15	700.78720000	16.88840000	0.00000000
16	782.93150000	0.05870000	0.00000000
17	816.51520000	0.00030000	0.00000000
18	838.64060000	3.27020000	0.00000000
19	848.70060000	0.38400000	0.00000000
20	859.67030000	4.72090000	0.00000000
21	864.58080000	6.17220000	0.00000000
22	874.46120000	20.84220000	0.00000000
23	946.29040000	3.36200000	0.00000000
24	977.57880000	0.00000000	0.00000000
25	1004.44800000	2.13170000	0.00000000
26	1007.92790000	1.61050000	0.00000000
27	1011.99160000	8.47340000	0.00000000
28	1017.54080000	14.46560000	0.00000000
29	1073.16910000	15.66660000	0.00000000
30	1158.38740000	0.35480000	0.00000000
31	1163.89570000	2.97530000	0.00000000
32	1165.35460000	0.34580000	0.00000000
33	1211.42450000	0.10110000	0.00000000
34	1248.27190000	25.09040000	0.00000000
35	1249.13570000	24.26400000	0.00000000
36	1286.68480000	2.81830000	0.00000000
37	1292.69660000	3.52050000	0.00000000
38	1318.15260000	0.68720000	0.00000000
39	1332.00670000	2.30100000	0.00000000
40	1332.44340000	1.53590000	0.00000000
41	1369.19950000	3.52580000	0.00000000
42	1369.54600000	2.76340000	0.00000000
43	1378.10120000	11.69090000	0.00000000
44	1483.49450000	2.12280000	0.00000000
45	1484.15690000	3.72790000	0.00000000
46	1487.38310000	43.13990000	0.00000000
47	1506.17420000	5.43810000	0.00000000
48	1506.61380000	5.39930000	0.00000000
49	1512.09690000	0.21800000	0.00000000
50	1549.21870000	12.17240000	0.00000000
51	2351.20100000	1248.45320000	0.00000000
52	3077.77540000	0.87620000	0.00000000
53	3080.10650000	3.19270000	0.00000000
54	3086.49420000	1.79500000	0.00000000
55	3093.77740000	0.01200000	0.00000000
56	3094.20390000	0.16170000	0.00000000
57	3100.25620000	0.94740000	0.00000000
58	3128.41300000	0.17660000	0.00000000
59	3131.32800000	1.61980000	0.00000000
60	3135.40740000	0.98240000	0.00000000

61	3154.39420000	0.00190000	0.00000000
62	3161.64440000	0.13440000	0.00000000
63	3162.10170000	0.11340000	0.00000000

S28. CALCULATIONS ON TS 14s ⟶ A

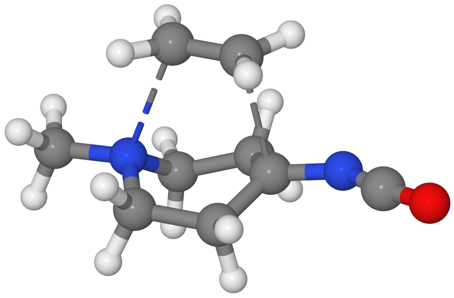

Route	:	# opt=(calcall,ts,noeigentest) freq b3lyp/cc-pvtz empiricaldispersion=gd3bj pop=regular geom=connectivity int=ultrafine
SMILES	:	CN1CC[C](CC1)[N][C]=O.C=C
Formula	:	$C_9H_{15}N_2O^+$
Charge	:	1
Multiplicity	:	1
Energy	:	-536.62820355 a.u.
Gibbs Energy	:	-536.43468800 a.u.
ZPE (B3LYP)	:	0.231684 a.u.
CCSD(T)-F12b Energy	:	-535.58476327 a.u.

Cartesian Co-ordinates (XYZ format)

```
27

C   0.23104300 -0.65624201  1.16140199
C   0.76223201 -0.12939100 -0.12478500
C  -1.31071103 -0.68514198  1.26374495
H   0.61220598 -1.68699598  1.17986095
H   0.68593699 -0.15526401  2.01336503
H  -1.61706805 -1.65933204  1.66419303
H  -1.64323103  0.07004400  1.97563601
C   0.08326700 -0.58116400 -1.37804699
H   0.08851300  0.22251500 -2.11162090
H   0.72643399 -1.36098194 -1.79881501
C  -1.32002795 -1.14477599 -1.10995197
H  -1.92862499 -1.03646803 -2.00429392
H  -1.26508796 -2.22130489 -0.89342701
C  -1.28105402  2.00521302 -0.14935701
H  -1.74172103  1.99350202 -1.12526405
H  -1.95447803  2.03520894  0.69274402
C   0.05759000  2.13176799  0.00207200
H   0.68811899  2.32691598 -0.85193402
H   0.48521900  2.30834293  0.97937101
N  -1.94856501 -0.41516399 -0.01837000
C  -3.39480710 -0.56450701  0.04330800
H  -3.79416108  0.06982600  0.83416498
H  -3.83640599 -0.25584400 -0.90231401
H  -3.69805789 -1.59893894  0.25047800
N   2.06108689  0.19544600 -0.24002901
C   2.97677994  0.46797001  0.52816302
O   3.91651511  0.74453598  1.12790000
```

Frequencies

Mode	IR frequency	IR intensity	Raman intensity
1	-266.69880000	329.70390000	0.00000000
2	64.26710000	0.97870000	0.00000000
3	86.30100000	0.73950000	0.00000000
4	102.46480000	0.99490000	0.00000000
5	126.49290000	14.46920000	0.00000000
6	155.36190000	1.10020000	0.00000000
7	183.60500000	3.39880000	0.00000000
8	201.30410000	2.81170000	0.00000000
9	231.68460000	0.33550000	0.00000000
10	240.83960000	0.64770000	0.00000000
11	297.91970000	9.38920000	0.00000000
12	333.40750000	1.91630000	0.00000000
13	365.57510000	14.35140000	0.00000000
14	376.50090000	4.68770000	0.00000000
15	411.45660000	52.04260000	0.00000000
16	440.08640000	5.92770000	0.00000000
17	512.26660000	2.53000000	0.00000000
18	528.11000000	11.61730000	0.00000000
19	586.98050000	10.05960000	0.00000000
20	614.80690000	16.13190000	0.00000000
21	664.75480000	28.35310000	0.00000000
22	726.80820000	58.09130000	0.00000000
23	758.33660000	16.42230000	0.00000000
24	830.61980000	43.98880000	0.00000000
25	854.13970000	0.61970000	0.00000000
26	928.15250000	77.12130000	0.00000000
27	961.21280000	44.53030000	0.00000000
28	971.03460000	42.19440000	0.00000000
29	989.96690000	2.74800000	0.00000000
30	1004.79930000	7.26820000	0.00000000
31	1021.97080000	10.42210000	0.00000000
32	1041.36940000	28.80190000	0.00000000
33	1071.40380000	11.27710000	0.00000000
34	1086.48080000	15.09320000	0.00000000
35	1135.40090000	60.85560000	0.00000000
36	1151.17970000	35.32410000	0.00000000
37	1180.04170000	10.49140000	0.00000000
38	1193.18840000	28.88150000	0.00000000
39	1221.40860000	13.09200000	0.00000000
40	1254.71560000	3.57740000	0.00000000
41	1264.08110000	31.22150000	0.00000000
42	1271.14070000	32.70950000	0.00000000
43	1295.13840000	15.96860000	0.00000000
44	1328.63580000	38.65310000	0.00000000
45	1337.51600000	78.65150000	0.00000000
46	1359.31900000	12.40760000	0.00000000
47	1389.23080000	4.14270000	0.00000000
48	1404.50320000	8.25390000	0.00000000
49	1423.15860000	6.56300000	0.00000000
50	1448.06280000	15.77080000	0.00000000
51	1452.60430000	4.02750000	0.00000000
52	1471.34050000	1.96960000	0.00000000
53	1483.29680000	4.22190000	0.00000000
54	1492.64120000	13.69290000	0.00000000
55	1505.46900000	3.40980000	0.00000000
56	1518.48470000	12.36590000	0.00000000
57	1530.08390000	3.72040000	0.00000000
58	1577.30620000	455.56900000	0.00000000
59	1612.03280000	14.17580000	0.00000000
60	2331.23740000	1789.57240000	0.00000000

61	2951.55150000	47.18740000	0.00000000
62	2973.82660000	31.83610000	0.00000000
63	2978.85200000	65.83530000	0.00000000
64	2986.20220000	10.51810000	0.00000000
65	3029.04720000	3.40960000	0.00000000
66	3067.54070000	9.67230000	0.00000000
67	3075.38890000	13.71210000	0.00000000
68	3095.56130000	4.42840000	0.00000000
69	3101.17150000	2.72260000	0.00000000
70	3109.58620000	1.48550000	0.00000000
71	3118.65270000	11.40870000	0.00000000
72	3143.17140000	1.24630000	0.00000000
73	3170.16020000	9.76660000	0.00000000
74	3231.50450000	0.31750000	0.00000000
75	3263.40950000	0.46180000	0.00000000

S29. CALCULATIONS ON A

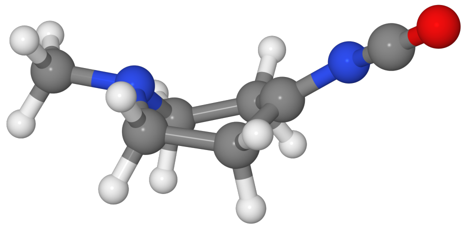

Route	:	# opt freq b3lyp/cc-pvtz empiricaldispersion=gd3bj pop=regular geom=connectivity int=ultrafine
SMILES	:	CN1CC[C](CC1)[N][C]=O
Formula	:	$C_7H_{11}N_2O^+$
Charge	:	1
Multiplicity	:	1
Energy	:	-458.00268513 a.u.
Gibbs Energy	:	-457.86186200 a.u.
CCSD(T)-F12b Energy	:	-457.13507737 a.u.

Cartesian Co-ordinates (XYZ format)

21

C	0.73531902	0.56291503	-0.35373601
C	0.30900800	-0.38860801	-1.39890802
H	0.38620001	0.18981700	-2.33309889
H	0.99505502	-1.22733200	-1.49294496
C	-1.15073895	-0.84287298	-1.19227600
H	-1.71374702	-0.75992399	-2.13258410
H	-1.14319098	-1.89300394	-0.90587503
C	-1.59498096	1.36148906	-0.31470701
H	-1.88367796	1.67381096	-1.32913601
H	-2.24054503	1.89597404	0.37685600
C	-0.14084400	1.69664097	-0.00510700
H	0.23133300	2.57166004	-0.56002802
H	0.00422900	1.93741906	1.04851305
N	-1.75617301	-0.07593000	-0.11817600
C	-3.13473701	-0.48191801	0.13380399
H	-3.51717901	0.04845400	1.00295603
H	-3.79122591	-0.27534899	-0.72197801
H	-3.16630602	-1.54953897	0.34187099
N	1.93098605	0.52098900	0.20618500
C	2.90205908	-0.24492501	0.14971299
O	3.86443400	-0.85600102	0.21124600

Frequencies

Mode	IR frequency	IR intensity	Raman intensity
1	35.27810000	0.90340000	0.00000000
2	75.19310000	0.52010000	0.00000000
3	110.29110000	0.83350000	0.00000000
4	127.60220000	1.30100000	0.00000000
5	173.98260000	3.53970000	0.00000000
6	220.91740000	0.20520000	0.00000000
7	339.34760000	3.55670000	0.00000000
8	355.91460000	5.74180000	0.00000000
9	368.30330000	0.81050000	0.00000000
10	443.49320000	28.62090000	0.00000000
11	456.24160000	3.34210000	0.00000000
12	538.97070000	5.10010000	0.00000000
13	584.37840000	16.05050000	0.00000000
14	624.42240000	22.52360000	0.00000000
15	667.13400000	16.57130000	0.00000000
16	739.24040000	7.25610000	0.00000000
17	767.48160000	15.94370000	0.00000000
18	823.76910000	13.64510000	0.00000000
19	926.85720000	18.98320000	0.00000000
20	967.09640000	12.26000000	0.00000000
21	978.38720000	3.71290000	0.00000000
22	1011.56930000	26.38700000	0.00000000
23	1063.59070000	0.47220000	0.00000000
24	1084.44370000	14.34500000	0.00000000
25	1136.31420000	65.96080000	0.00000000
26	1146.78410000	11.68030000	0.00000000
27	1154.52750000	10.25300000	0.00000000
28	1187.09780000	14.54350000	0.00000000
29	1213.17580000	3.17870000	0.00000000
30	1230.95590000	20.89060000	0.00000000
31	1274.73540000	11.91520000	0.00000000
32	1286.71170000	53.96560000	0.00000000
33	1307.55970000	59.41700000	0.00000000
34	1342.44490000	17.32060000	0.00000000
35	1388.74470000	0.64950000	0.00000000
36	1396.90440000	31.06410000	0.00000000
37	1410.85800000	8.06520000	0.00000000
38	1417.08690000	4.56700000	0.00000000
39	1442.94360000	12.91170000	0.00000000
40	1468.87490000	3.88130000	0.00000000
41	1493.74500000	15.32210000	0.00000000
42	1497.50120000	1.42270000	0.00000000
43	1511.80880000	17.65560000	0.00000000
44	1518.70700000	4.29320000	0.00000000
45	1627.34970000	664.41500000	0.00000000
46	2334.23320000	1650.91750000	0.00000000
47	2953.11080000	25.45720000	0.00000000
48	2960.92850000	55.70290000	0.00000000
49	2967.93650000	3.02750000	0.00000000
50	2975.23320000	62.58230000	0.00000000
51	2978.81560000	6.55670000	0.00000000
52	3080.71130000	4.93820000	0.00000000
53	3087.95930000	1.18580000	0.00000000
54	3089.77070000	14.10220000	0.00000000
55	3108.65510000	3.03450000	0.00000000
56	3117.19460000	0.85920000	0.00000000
57	3130.58200000	7.91950000	0.00000000

S30. CALCULATIONS ON A′

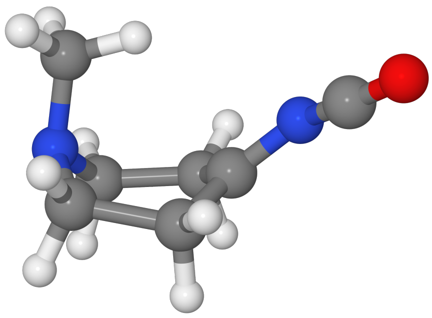

Route	:	# opt freq b3lyp/cc-pvtz empiricaldispersion=gd3bj pop=regular geom=connectivity int=ultrafine
SMILES	:	CN1CC[C](CC1)[N][C]=O
Formula	:	$C_7H_{11}N_2O^+$
Charge	:	1
Multiplicity	:	1
Energy	:	-458.00163446 a.u.
Gibbs Energy	:	-457.85999700 a.u.
CCSD(T)-F12b Energy	:	-457.13084746 a.u.

Cartesian Co-ordinates (XYZ format)

21

C	2.91140509	-0.25844699	2.08890605
C	2.32672095	1.03540599	1.76964605
H	1.43197894	1.15497398	2.38390803
H	2.99766493	1.86947203	1.95326996
C	1.73701000	1.04869199	0.22975200
H	0.70476502	1.37535298	0.31483099
H	2.30375600	1.78757799	-0.32805100
C	1.26529801	-1.25436699	0.42928201
H	0.24514200	-0.99620599	0.70238298
H	1.25421798	-2.20819592	-0.09063900
C	2.12362909	-1.45060694	1.77572894
H	1.37464499	-1.57076800	2.56561303
H	2.74861312	-2.33901191	1.77563703
N	1.80452704	-0.22233500	-0.40460399
C	3.10177207	-0.54048097	-0.97042400
H	3.39750409	0.22958100	-1.67841697
H	3.88616395	-0.61258698	-0.19772400
H	3.05528402	-1.49619997	-1.48729503
N	4.14475918	-0.41740799	2.56662607
C	5.03625202	0.33227199	2.96925211
O	5.94093513	0.90180099	3.38001704

Frequencies

Mode	IR frequency	IR intensity	Raman intensity
1	47.53550000	0.38800000	0.00000000
2	63.00190000	1.57850000	0.00000000
3	109.99510000	3.76660000	0.00000000
4	162.56740000	10.02700000	0.00000000
5	175.36790000	0.25030000	0.00000000
6	246.35580000	0.20730000	0.00000000
7	342.67920000	5.15730000	0.00000000
8	396.98900000	4.87100000	0.00000000
9	409.71460000	13.51140000	0.00000000
10	434.92470000	4.32380000	0.00000000
11	501.66400000	12.52290000	0.00000000
12	532.36820000	50.59210000	0.00000000
13	613.36570000	18.13920000	0.00000000
14	622.39570000	17.29040000	0.00000000
15	669.57650000	98.78290000	0.00000000
16	733.32750000	13.56890000	0.00000000
17	756.54680000	55.75560000	0.00000000
18	803.92900000	80.10340000	0.00000000
19	814.64310000	96.70510000	0.00000000
20	877.82010000	50.34860000	0.00000000
21	924.43680000	7.19650000	0.00000000
22	962.43450000	5.29920000	0.00000000
23	1024.63430000	1.86830000	0.00000000
24	1051.36950000	14.08620000	0.00000000
25	1103.58580000	137.65360000	0.00000000
26	1134.76670000	80.99170000	0.00000000
27	1142.06090000	21.88070000	0.00000000
28	1152.09350000	27.21490000	0.00000000
29	1172.94190000	50.76670000	0.00000000
30	1193.46300000	36.62820000	0.00000000
31	1268.86310000	15.49400000	0.00000000
32	1294.76430000	24.50310000	0.00000000
33	1328.21500000	34.99600000	0.00000000
34	1332.85560000	20.20100000	0.00000000
35	1377.99130000	5.33200000	0.00000000
36	1395.75440000	20.41750000	0.00000000
37	1402.78540000	22.10670000	0.00000000
38	1445.96850000	27.00440000	0.00000000
39	1463.40790000	15.13600000	0.00000000
40	1479.08940000	12.58960000	0.00000000
41	1489.93370000	23.44300000	0.00000000
42	1493.48220000	21.98540000	0.00000000
43	1499.43400000	3.83890000	0.00000000
44	1521.50640000	1.58880000	0.00000000
45	1612.69300000	484.95670000	0.00000000
46	2329.10590000	1789.94500000	0.00000000
47	2923.81230000	52.76230000	0.00000000
48	3028.19960000	23.36000000	0.00000000
49	3051.38490000	14.90210000	0.00000000
50	3084.15680000	8.60970000	0.00000000
51	3090.61110000	4.79810000	0.00000000
52	3093.37070000	11.23380000	0.00000000
53	3126.58460000	1.97920000	0.00000000
54	3130.29470000	5.75500000	0.00000000
55	3140.51440000	0.90990000	0.00000000
56	3143.66850000	3.04480000	0.00000000
57	3154.88230000	2.54550000	0.00000000

S31. CALCULATIONS ON TS A′ ⟶ B (INTERMEDIATE)

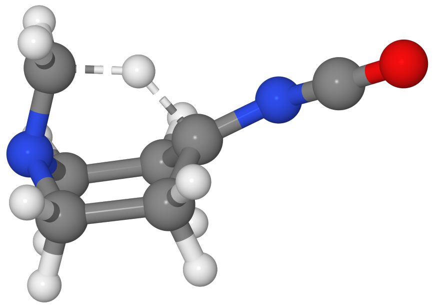

Route	:	# opt=(calcall,qst3) freq b3lyp/cc-pvtz empiricaldispersion=gd3bj pop=regular geom=connectivity int=ultrafine
SMILES	:	CN1CC[C](CC1)[N][C]=O
Formula	:	$C_7H_{11}N_2O^+$
Charge	:	1
Multiplicity	:	1
Energy	:	-457.98678649 a.u.
Gibbs Energy	:	-457.84490600 a.u.
CCSD(T)-F12b Energy	:	-457.12121319 a.u.

Cartesian Co-ordinates (XYZ format)

21

C	0.30912799	0.37108400	-0.01473800
C	0.01938700	-0.61998999	-1.11758900
H	0.17959800	-0.10843500	-2.06676006
H	0.70633298	-1.46234405	-1.06941795
C	-1.48551595	-1.07045305	-1.04761600
H	-2.04166007	-0.74145103	-1.91860294
H	-1.55937898	-2.15231800	-0.98096699
C	-2.13436389	0.99383301	0.04705000
H	-2.67374206	1.26152897	-0.85499001
H	-2.67354393	1.39428103	0.90101302
C	-0.65695399	1.52665305	0.02243800
H	-0.51424402	2.09723496	-0.89583302
H	-0.42643300	2.17983389	0.85953701
N	-2.08414197	-0.47026801	0.15117100
C	-1.42206502	-0.85554200	1.27067006
H	-1.18397403	-1.91016603	1.36063695
H	-0.21561700	-0.34640601	1.03654099
H	-1.67760301	-0.34301800	2.19202304
N	1.59620404	0.64533597	0.36271599
C	2.67908692	0.09526500	0.24372999
O	3.75498390	-0.31374300	0.19487700

Frequencies

Mode	IR frequency	IR intensity	Raman intensity
1	-888.21950000	2172.15870000	0.00000000
2	67.54810000	0.04490000	0.00000000
3	93.95640000	1.25730000	0.00000000
4	133.28200000	0.57160000	0.00000000
5	248.37430000	3.90840000	0.00000000
6	342.66540000	1.97490000	0.00000000
7	374.57940000	10.39990000	0.00000000
8	430.74400000	0.64600000	0.00000000
9	455.48440000	0.65220000	0.00000000
10	502.66600000	5.73090000	0.00000000
11	554.59570000	0.91350000	0.00000000
12	557.60080000	75.24250000	0.00000000
13	604.11980000	19.39960000	0.00000000
14	667.74020000	25.94550000	0.00000000
15	725.12660000	1.85820000	0.00000000
16	729.53590000	12.14540000	0.00000000
17	812.23950000	34.77800000	0.00000000
18	823.30140000	0.18080000	0.00000000
19	880.47990000	36.23750000	0.00000000
20	908.21940000	2.47130000	0.00000000
21	933.45440000	33.10300000	0.00000000
22	967.78750000	90.26300000	0.00000000
23	980.92020000	7.58120000	0.00000000
24	1004.52100000	5.36960000	0.00000000
25	1108.13310000	2.17240000	0.00000000
26	1139.75140000	13.41580000	0.00000000
27	1144.93730000	18.49770000	0.00000000
28	1198.42680000	15.13150000	0.00000000
29	1221.26620000	54.74140000	0.00000000
30	1230.68390000	24.13740000	0.00000000
31	1247.14220000	21.40180000	0.00000000
32	1298.93930000	8.29020000	0.00000000
33	1306.43520000	1.05530000	0.00000000
34	1332.03040000	1.36340000	0.00000000
35	1349.07850000	115.89880000	0.00000000
36	1350.35270000	55.31450000	0.00000000
37	1364.21300000	10.58660000	0.00000000
38	1382.10820000	1.69640000	0.00000000
39	1459.89380000	114.05140000	0.00000000
40	1475.04100000	4.65420000	0.00000000
41	1491.15950000	0.55650000	0.00000000
42	1505.30350000	11.90570000	0.00000000
43	1516.38130000	35.46380000	0.00000000
44	1539.65870000	57.16130000	0.00000000
45	1548.37580000	55.01560000	0.00000000
46	1609.69220000	40.60580000	0.00000000
47	2347.31570000	1560.44800000	0.00000000
48	3062.68760000	1.47510000	0.00000000
49	3068.55850000	5.60480000	0.00000000
50	3100.45300000	2.89040000	0.00000000
51	3100.85730000	8.26060000	0.00000000
52	3109.39450000	1.95050000	0.00000000
53	3112.24560000	4.25020000	0.00000000
54	3126.14740000	2.17600000	0.00000000
55	3154.90440000	0.24010000	0.00000000
56	3158.92730000	2.65900000	0.00000000
57	3206.34530000	1.35990000	0.00000000

S32. CALCULATIONS ON B (INTERMEDIATE)

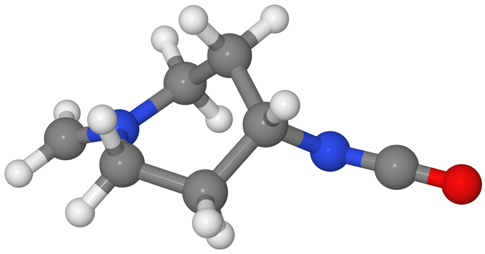

Route	: # opt freq b3lyp/cc-pvtz empiricaldispersion=gd3bj pop=regular geom=connectivity int=ultrafine
SMILES	: [CH2]N1CCC(CC1)[N][C]=O
Formula	: $C_7H_{11}N_2O^+$
Charge	: 1
Multiplicity	: 1
Energy	: -458.03471751 a.u.
Gibbs Energy	: -457.89119000 a.u.
CCSD(T)-F12b Energy	: -457.17538007 a.u.

Cartesian Co-ordinates (XYZ format)

```
21

C 3.53764701 0.05397400 1.96389604
C 2.77951407 1.19670904 1.23599505
H 1.83753705 1.37555099 1.75280595
H 3.34479690 2.12541103 1.28012097
C 2.49868298 0.87856197 -0.22434700
H 1.95429099 1.67613006 -0.72234201
H 3.41790390 0.69036102 -0.77943301
C 2.01828003 -1.44848502 0.64917201
H 1.30236304 -1.36548805 1.46793699
H 1.87295198 -2.40032196 0.14575000
C 3.42953110 -1.25776505 1.16995597
H 3.67659092 -2.09592390 1.81719899
H 4.14473391 -1.26892602 0.34696099
N 1.68197405 -0.36286300 -0.32675999
C 0.74991000 -0.49256501 -1.17886198
H 0.51524401 0.32113299 -1.85250294
H 0.19160999 -1.41872704 -1.22863102
N 2.94533896 -0.14257500 3.27123189
C 3.32212710 0.10197900 4.39568806
O 3.57823992 0.28965399 5.51212215
H 4.59131193 0.31269601 2.05835605
```

Frequencies

Mode	IR frequency	IR intensity	Raman intensity
1	21.75880000	2.08070000	0.00000000
2	24.63280000	1.16610000	0.00000000
3	112.36230000	0.83370000	0.00000000
4	162.88500000	8.40150000	0.00000000
5	247.73340000	6.74910000	0.00000000
6	339.83450000	2.70220000	0.00000000
7	375.97000000	2.66830000	0.00000000
8	417.55950000	1.63330000	0.00000000
9	462.39850000	4.00910000	0.00000000
10	493.60280000	2.07620000	0.00000000
11	549.40180000	8.17490000	0.00000000
12	593.38930000	1.89250000	0.00000000
13	597.12210000	20.42700000	0.00000000
14	657.72400000	18.72080000	0.00000000
15	707.48080000	0.37650000	0.00000000
16	732.35090000	0.87800000	0.00000000
17	776.28530000	7.20150000	0.00000000
18	834.82500000	5.25180000	0.00000000
19	897.29190000	4.05370000	0.00000000
20	918.01270000	11.18510000	0.00000000
21	987.62950000	6.66730000	0.00000000
22	1013.53660000	14.06350000	0.00000000
23	1026.22530000	19.34770000	0.00000000
24	1049.08350000	1.48620000	0.00000000
25	1104.10790000	29.64000000	0.00000000
26	1113.64710000	1.20260000	0.00000000
27	1119.02050000	3.86250000	0.00000000
28	1199.87600000	0.28460000	0.00000000
29	1225.54430000	4.55880000	0.00000000
30	1254.45600000	3.49640000	0.00000000
31	1289.43700000	10.93780000	0.00000000
32	1311.88250000	14.93720000	0.00000000
33	1349.29600000	18.86010000	0.00000000
34	1356.33130000	2.50910000	0.00000000
35	1377.29430000	17.56820000	0.00000000
36	1387.66320000	5.38150000	0.00000000
37	1397.42340000	5.48490000	0.00000000
38	1408.56060000	4.21370000	0.00000000
39	1473.42430000	60.16710000	0.00000000
40	1481.25490000	8.91530000	0.00000000
41	1485.25330000	14.25630000	0.00000000
42	1491.95280000	21.95230000	0.00000000
43	1497.43540000	29.70790000	0.00000000
44	1503.21350000	30.31690000	0.00000000
45	1739.93820000	13.01670000	0.00000000
46	2351.12270000	1359.97420000	0.00000000
47	3056.22830000	2.04680000	0.00000000
48	3065.05290000	5.52890000	0.00000000
49	3066.77020000	3.42850000	0.00000000
50	3071.49670000	5.25900000	0.00000000
51	3074.25670000	10.38210000	0.00000000
52	3117.39070000	2.25990000	0.00000000
53	3121.19890000	1.49090000	0.00000000
54	3132.08750000	1.29910000	0.00000000
55	3133.01410000	1.43830000	0.00000000
56	3140.09230000	4.81380000	0.00000000
57	3252.37550000	6.43410000	0.00000000

S33. CALCULATIONS ON B (FINAL)

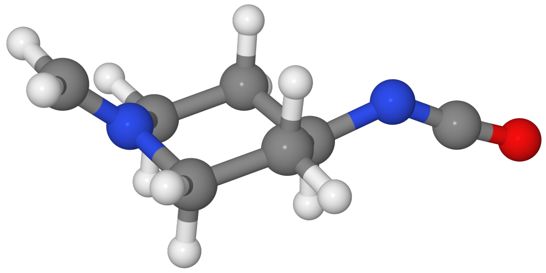

Route	:	# opt freq b3lyp/cc-pvtz empiricaldispersion=gd3bj pop=regular geom=connectivity int=ultrafine
SMILES	:	[CH2]N1CCC(CC1)[N][C]=O
Formula	:	$C_7H_{11}N_2O^+$
Charge	:	1
Multiplicity	:	1
Energy	:	-458.04055726 a.u.
Gibbs Energy	:	-457.89616100 a.u.
CCSD(T)-F12b Energy	:	-457.18114445 a.u.

Cartesian Co-ordinates (XYZ format)

21

C	-0.69046003	0.11274400	-0.16687600
C	-0.00147000	-1.16303098	0.33077100
H	0.05731400	-1.13497806	1.41981304
H	-0.58835000	-2.03964591	0.06089800
C	1.39334595	-1.32075500	-0.26609200
H	1.35121000	-1.41702402	-1.35193098
H	1.92006803	-2.17930698	0.14087801
C	1.59434295	1.15424001	-0.48588300
H	2.25751805	1.97518206	-0.22815000
H	1.55266201	1.06436706	-1.57228994
C	0.20093501	1.32887602	0.10953200
H	0.26996800	1.48281801	1.18739605
H	-0.24178299	2.22721195	-0.31790999
N	2.19989800	-0.10635800	0.00964600
C	3.29444790	-0.13812000	0.65299302
H	3.83538890	0.77973503	0.84215701
H	3.68407607	-1.08325803	1.00762200
N	-1.94423199	0.27561501	0.52052897
C	-3.09705496	0.33704099	0.15956201
O	-4.23294401	0.40879101	-0.07004500
H	-0.84920001	0.02977100	-1.24674797

Frequencies

Mode	IR frequency	IR intensity	Raman intensity
1	13.89170000	0.56720000	0.00000000
2	68.58250000	0.45560000	0.00000000
3	132.98450000	10.12010000	0.00000000
4	201.82410000	0.07810000	0.00000000
5	240.72990000	2.17590000	0.00000000
6	335.10890000	3.52390000	0.00000000
7	348.42550000	4.28980000	0.00000000
8	439.05180000	5.58380000	0.00000000
9	442.89510000	0.19940000	0.00000000
10	466.61000000	1.86620000	0.00000000
11	487.99540000	0.58870000	0.00000000
12	596.08450000	19.79480000	0.00000000
13	638.72160000	11.49810000	0.00000000
14	675.68820000	25.40240000	0.00000000
15	716.39860000	0.04760000	0.00000000
16	750.82770000	0.35530000	0.00000000
17	798.71510000	0.77450000	0.00000000
18	840.05900000	14.45730000	0.00000000
19	922.87640000	1.56260000	0.00000000
20	942.35360000	14.48420000	0.00000000
21	961.75320000	8.79640000	0.00000000
22	1020.94250000	2.22570000	0.00000000
23	1034.15740000	1.95040000	0.00000000
24	1042.50810000	25.81910000	0.00000000
25	1107.28670000	22.77990000	0.00000000
26	1115.18640000	2.59990000	0.00000000
27	1141.08960000	3.55830000	0.00000000
28	1185.94820000	1.74350000	0.00000000
29	1224.74880000	0.01000000	0.00000000
30	1275.07400000	19.08800000	0.00000000
31	1291.44590000	1.81930000	0.00000000
32	1326.05860000	13.37380000	0.00000000
33	1342.80850000	0.25470000	0.00000000
34	1346.81670000	13.81310000	0.00000000
35	1376.62390000	4.94460000	0.00000000
36	1388.75120000	4.27360000	0.00000000
37	1389.54300000	0.36930000	0.00000000
38	1410.38290000	17.04120000	0.00000000
39	1485.30810000	58.82900000	0.00000000
40	1485.42900000	6.37730000	0.00000000
41	1488.77830000	5.58760000	0.00000000
42	1490.57720000	22.74020000	0.00000000
43	1500.46410000	4.68780000	0.00000000
44	1512.85980000	58.76560000	0.00000000
45	1747.56840000	10.55750000	0.00000000
46	2356.26640000	1377.27010000	0.00000000
47	3006.04690000	18.66730000	0.00000000
48	3053.93510000	0.85110000	0.00000000
49	3058.69240000	4.12360000	0.00000000
50	3062.13300000	0.37410000	0.00000000
51	3066.91600000	6.43900000	0.00000000
52	3108.79610000	1.42330000	0.00000000
53	3110.72080000	4.12580000	0.00000000
54	3133.69350000	2.12630000	0.00000000
55	3135.30900000	3.18250000	0.00000000
56	3143.30490000	4.92110000	0.00000000
57	3255.74390000	6.19480000	0.00000000

S34. CALCULATIONS ON TS A′ ⟶ C

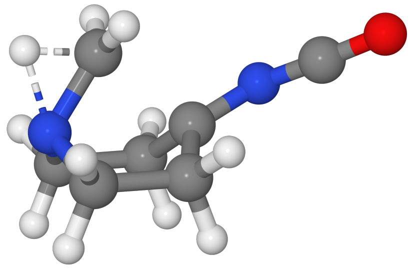

Route	:	# opt=(calcall,qst3) freq b3lyp/cc-pvtz empiricaldispersion=gd3bj pop=regular geom=connectivity int=ultrafine
SMILES	:	CN1CC[C](CC1)[N][C]=O
Formula	:	$C_7H_{11}N_2O^+$
Charge	:	1
Multiplicity	:	1
Energy	:	-457.90845152 a.u.
Gibbs Energy	:	-457.76862400 a.u.
CCSD(T)-F12b Energy	:	-457.04394044 a.u.

Cartesian Co-ordinates (XYZ format)

21

C	0.19252101	0.31979501	-0.02034300
C	-0.02116200	-0.58896500	-1.20471704
H	0.14325000	-0.00862100	-2.11402011
H	0.68718302	-1.41450405	-1.20095599
C	-1.49879801	-1.07522798	-1.15460801
H	-2.09577799	-0.76251101	-2.00650501
H	-1.54625499	-2.16011095	-1.09318805
C	-2.18857503	0.96914101	0.01817400
H	-2.77535892	1.24547601	-0.85320199
H	-2.71905899	1.31663895	0.90182298
C	-0.73013401	1.50738001	0.00132200
H	-0.56224102	2.08318400	-0.91076499
H	-0.50438797	2.14985394	0.84764701
N	-2.09008789	-0.49689800	0.06009100
C	-1.05912495	-0.76790601	1.14072394
H	-0.52923101	-1.70861101	1.11196005
H	-1.04950094	-0.12874600	2.01127195
H	-2.19485998	-1.21476698	1.26092696
N	1.42645204	0.49382499	0.52203900
C	2.50259805	-0.07986500	0.48387900
O	3.56518197	-0.52330601	0.53124100

Frequencies

Mode	IR frequency	IR intensity	Raman intensity
1	-1517.70600000	1762.46070000	0.00000000
2	70.17350000	0.16630000	0.00000000
3	94.10990000	1.52330000	0.00000000
4	168.84950000	0.80340000	0.00000000
5	214.13870000	2.81910000	0.00000000
6	304.56350000	10.20300000	0.00000000
7	365.13500000	6.76000000	0.00000000
8	406.13950000	0.84430000	0.00000000
9	447.49670000	12.44710000	0.00000000
10	464.08900000	10.19750000	0.00000000
11	501.79340000	4.02280000	0.00000000
12	570.27110000	2.09670000	0.00000000
13	603.90690000	19.14250000	0.00000000
14	604.34330000	3.21510000	0.00000000
15	661.17670000	25.21470000	0.00000000
16	729.15680000	6.87620000	0.00000000
17	765.35970000	61.01160000	0.00000000
18	810.05060000	0.44110000	0.00000000
19	819.16120000	10.97280000	0.00000000
20	893.88660000	42.69840000	0.00000000
21	926.01720000	34.83170000	0.00000000
22	932.17490000	2.90900000	0.00000000
23	963.32590000	5.71670000	0.00000000
24	968.41660000	0.58600000	0.00000000
25	994.10690000	34.34380000	0.00000000
26	996.79230000	13.51350000	0.00000000
27	1081.98050000	32.68980000	0.00000000
28	1099.31130000	2.00470000	0.00000000
29	1161.26770000	34.66790000	0.00000000
30	1187.72130000	0.71720000	0.00000000
31	1212.04970000	54.27920000	0.00000000
32	1233.01940000	34.93100000	0.00000000
33	1254.22140000	1.24740000	0.00000000
34	1292.31100000	2.72230000	0.00000000
35	1297.64220000	5.89640000	0.00000000
36	1309.01790000	11.51400000	0.00000000
37	1338.20430000	32.76860000	0.00000000
38	1360.57290000	4.89980000	0.00000000
39	1374.56430000	8.63830000	0.00000000
40	1447.53980000	3.50470000	0.00000000
41	1479.06030000	3.13560000	0.00000000
42	1493.86470000	18.41090000	0.00000000
43	1497.25880000	20.22090000	0.00000000
44	1517.28970000	4.44150000	0.00000000
45	1574.89320000	294.36050000	0.00000000
46	2213.02580000	249.15780000	0.00000000
47	2344.40500000	1571.30630000	0.00000000
48	3054.52540000	0.99850000	0.00000000
49	3062.23820000	3.68570000	0.00000000
50	3089.14850000	1.74160000	0.00000000
51	3090.07950000	4.46950000	0.00000000
52	3109.77990000	4.08730000	0.00000000
53	3125.19040000	1.38460000	0.00000000
54	3132.89560000	10.83340000	0.00000000
55	3139.95630000	0.78980000	0.00000000
56	3144.28570000	0.96200000	0.00000000
57	3271.73420000	7.21740000	0.00000000

S35. CALCULATIONS ON C

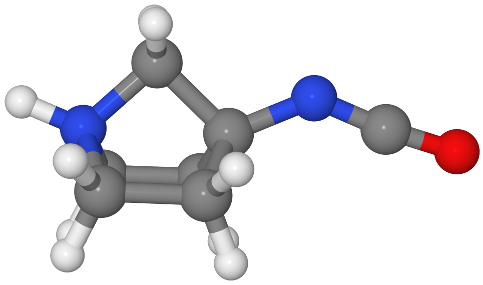

Route	:	# opt freq b3lyp/cc-pvtz empiricaldispersion=gd3bj pop=regular geom=connectivity int=ultrafine
SMILES	:	C1C[NH]2CCC1(C2)[N][C]=O
Formula	:	$C_7H_{11}N_2O^+$
Charge	:	1
Multiplicity	:	1
Energy	:	-458.04656187 a.u.
Gibbs Energy	:	-457.89711900 a.u.
CCSD(T)-F12b Energy	:	-457.19381917 a.u.

Cartesian Co-ordinates (XYZ format)

21

C	0.14588299	0.04672700	0.18919900
C	-0.01735300	-0.98547000	-0.95447099
H	0.33948800	-0.59439999	-1.90346503
H	0.54100901	-1.88820100	-0.72157103
C	-1.53642499	-1.25816500	-0.96196800
H	-2.04890203	-0.98364699	-1.87794495
H	-1.78696001	-2.28272605	-0.70322502
C	-1.98586702	1.08990502	-0.28559500
H	-2.46074200	1.16993999	-1.25776100
H	-2.54536295	1.67662799	0.43700799
C	-0.47234601	1.39128900	-0.26934800
H	-0.09834300	1.69472396	-1.24345803
H	-0.23651101	2.17176008	0.44911501
N	-2.03793597	-0.36371601	0.16269600
C	-0.92848003	-0.44288701	1.17436695
H	-0.79453403	-1.46433198	1.51775396
H	-1.11866999	0.22917500	2.00585699
N	1.43673897	0.12760700	0.76616597
C	2.52751589	0.45606899	0.35433200
O	3.61438990	0.74563098	0.07486900
H	-2.95571899	-0.63789201	0.50476700

Frequencies

Mode	IR frequency	IR intensity	Raman intensity
1	27.77350000	0.78520000	0.00000000
2	98.77250000	1.73050000	0.00000000
3	157.64210000	0.24620000	0.00000000
4	289.81180000	5.76950000	0.00000000
5	352.21440000	2.65960000	0.00000000
6	398.76840000	4.87830000	0.00000000
7	418.58420000	5.60680000	0.00000000
8	488.41270000	0.44950000	0.00000000
9	502.37900000	0.16460000	0.00000000
10	556.91180000	1.61980000	0.00000000
11	601.36680000	21.38150000	0.00000000
12	685.16260000	20.82610000	0.00000000
13	754.38600000	0.05390000	0.00000000
14	786.79050000	9.00600000	0.00000000
15	806.36860000	0.51680000	0.00000000
16	828.67250000	1.61440000	0.00000000
17	869.22640000	0.24840000	0.00000000
18	898.87380000	5.74640000	0.00000000
19	935.74050000	3.92210000	0.00000000
20	957.39680000	0.98410000	0.00000000
21	975.25450000	8.62620000	0.00000000
22	985.11820000	7.08700000	0.00000000
23	992.56610000	0.01760000	0.00000000
24	1029.69360000	2.11600000	0.00000000
25	1110.46330000	1.07610000	0.00000000
26	1111.39350000	32.59650000	0.00000000
27	1179.98690000	4.46000000	0.00000000
28	1205.51650000	0.04280000	0.00000000
29	1223.46600000	14.12440000	0.00000000
30	1232.16170000	5.30340000	0.00000000
31	1256.86880000	0.06810000	0.00000000
32	1278.97240000	0.14070000	0.00000000
33	1289.41410000	0.16080000	0.00000000
34	1296.02430000	3.97040000	0.00000000
35	1319.83930000	22.01780000	0.00000000
36	1323.98700000	10.12560000	0.00000000
37	1386.93190000	28.48890000	0.00000000
38	1398.18160000	9.30840000	0.00000000
39	1404.59450000	16.99330000	0.00000000
40	1501.48400000	0.00110000	0.00000000
41	1509.03010000	17.66880000	0.00000000
42	1512.53540000	11.10210000	0.00000000
43	1514.03300000	49.26330000	0.00000000
44	1519.47090000	27.35000000	0.00000000
45	1545.76510000	25.48120000	0.00000000
46	2359.24320000	1258.94060000	0.00000000
47	3089.46610000	1.01880000	0.00000000
48	3090.74870000	4.39360000	0.00000000
49	3101.14060000	2.17070000	0.00000000
50	3103.19010000	1.08170000	0.00000000
51	3105.84870000	2.06950000	0.00000000
52	3134.58630000	0.50430000	0.00000000
53	3137.56920000	1.42560000	0.00000000
54	3159.31030000	0.13600000	0.00000000
55	3165.09020000	0.97440000	0.00000000
56	3170.63600000	0.49150000	0.00000000
57	3494.79590000	77.54510000	0.00000000

S36. CALCULATIONS ON 9 (ISOMER 1)

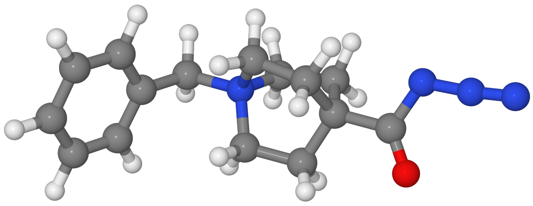

Route	:	# opt freq b3lyp/cc-pvtz empiricaldispersion=gd3bj pop=regular geom=connectivity int=ultrafine
SMILES	:	c1ccc(cc1)C[N]23CCC(CC2)(CC3)C(=O)N=[N+]=[N-]
Formula	:	$C_{15}H_{19}N_4O^+$
Charge	:	1
Multiplicity	:	1
Energy	:	-877.37718440 a.u.
Gibbs Energy	:	-877.09134100 a.u.

Cartesian Co-ordinates (XYZ format)

39

C	-0.12287700	0.49890599	-1.24604404
C	0.32547501	-0.31374499	-0.01674300
C	-1.65739095	0.56702000	-1.26154697
H	0.31020600	1.49547398	-1.20954502
H	0.22501600	0.02707400	-2.16305304
H	-2.02092290	1.56674802	-1.48312104
H	-2.09413505	-0.12295900	-1.97935295
C	-0.02259500	0.50458503	1.23996103
H	0.13233900	-0.10589900	2.12840295
H	0.62762302	1.37178600	1.32005894
C	-1.48535502	0.96546900	1.15257299
H	-2.01865792	0.81401199	2.08431697
H	-1.57247198	2.01205111	0.86953098
C	-1.95228899	-1.29349506	0.30980399
H	-2.23310089	-1.50384903	1.33659804
H	-2.62779808	-1.83746302	-0.34403101
C	-0.47965500	-1.61648798	0.01976800
H	-0.08766300	-2.27971506	0.78592402
H	-0.37663200	-2.14001703	-0.92862302
N	-2.21649098	0.18005200	0.08944600
C	-3.71608901	0.49114999	0.10159100
C	1.81496799	-0.61941898	-0.07645300
O	2.27224803	-1.73254597	-0.04928800
N	2.57348299	0.56859201	-0.16970900
N	3.81000495	0.38532099	-0.22665501
N	4.92175102	0.31931701	-0.28310600
H	-4.11798000	-0.01878500	-0.77125001
H	-3.78490901	1.56391203	-0.06446400
C	-4.43978977	0.08881100	1.34890401
C	-4.58359718	0.99217701	2.40320802
C	-5.01608992	-1.17752194	1.45730495
C	-5.26272106	0.62572002	3.55682611
H	-4.18306303	1.99393499	2.31589508
C	-5.69468307	-1.54520500	2.61033106

H	-4.95215797	-1.87472796	0.63226199
C	-5.81255007	-0.64608002	3.66391301
H	-5.37384415	1.33510303	4.36431694
H	-6.14177990	-2.52634406	2.68177605
H	-6.34654617	-0.93025702	4.55941391

Frequencies

Mode	IR frequency	IR intensity	Raman intensity
1	26.71190000	0.50670000	0.00000000
2	40.29470000	0.57650000	0.00000000
3	49.73910000	0.86490000	0.00000000
4	67.02800000	0.64320000	0.00000000
5	83.29180000	0.07090000	0.00000000
6	105.59410000	0.32370000	0.00000000
7	120.12790000	0.05860000	0.00000000
8	166.41400000	3.84270000	0.00000000
9	187.16690000	2.27420000	0.00000000
10	214.85030000	0.83760000	0.00000000
11	234.17880000	0.39890000	0.00000000
12	245.30220000	1.19970000	0.00000000
13	301.34310000	1.15740000	0.00000000
14	328.76220000	0.24920000	0.00000000
15	338.33790000	9.68840000	0.00000000
16	370.18580000	1.58620000	0.00000000
17	403.16810000	0.34260000	0.00000000
18	418.33390000	0.01760000	0.00000000
19	422.02840000	0.07970000	0.00000000
20	467.96160000	3.10650000	0.00000000
21	476.96710000	3.94630000	0.00000000
22	526.82830000	0.34650000	0.00000000
23	540.51960000	0.15640000	0.00000000
24	547.72640000	0.64990000	0.00000000
25	589.60520000	4.22980000	0.00000000
26	591.80450000	7.66880000	0.00000000
27	639.11510000	1.48150000	0.00000000
28	639.33790000	3.94490000	0.00000000
29	702.22490000	6.52420000	0.00000000
30	724.32720000	45.09600000	0.00000000
31	737.28930000	2.82070000	0.00000000
32	786.29880000	38.69100000	0.00000000
33	803.02360000	3.19860000	0.00000000
34	835.12340000	5.31820000	0.00000000
35	836.64280000	1.25950000	0.00000000
36	839.17300000	3.86270000	0.00000000
37	854.91590000	2.17380000	0.00000000
38	869.48450000	23.96400000	0.00000000
39	869.66510000	80.07840000	0.00000000
40	905.69880000	1.04500000	0.00000000
41	942.44900000	67.05550000	0.00000000
42	955.73210000	2.57660000	0.00000000
43	988.42380000	6.30800000	0.00000000
44	993.14710000	0.85030000	0.00000000
45	1002.30770000	7.92620000	0.00000000
46	1006.19720000	0.03600000	0.00000000
47	1014.23800000	3.69900000	0.00000000
48	1018.69640000	4.36820000	0.00000000
49	1028.39330000	3.77400000	0.00000000
50	1036.32960000	0.01950000	0.00000000
51	1048.36620000	18.39500000	0.00000000
52	1053.02270000	4.96560000	0.00000000
53	1056.68610000	2.13990000	0.00000000
54	1063.33290000	10.08450000	0.00000000
55	1073.84600000	8.33100000	0.00000000
56	1122.09850000	5.14690000	0.00000000
57	1182.24570000	35.44110000	0.00000000
58	1191.05960000	3.80810000	0.00000000
59	1196.26350000	0.16340000	0.00000000
60	1204.70050000	0.47280000	0.00000000

61	1214.04440000	1.60750000	0.00000000
62	1214.85240000	0.79490000	0.00000000
63	1240.47230000	26.41110000	0.00000000
64	1255.88380000	153.97570000	0.00000000
65	1264.31970000	555.09660000	0.00000000
66	1286.66550000	95.02090000	0.00000000
67	1297.69700000	7.62570000	0.00000000
68	1302.66350000	3.22750000	0.00000000
69	1330.06420000	3.26370000	0.00000000
70	1334.89850000	3.08170000	0.00000000
71	1347.17040000	0.57120000	0.00000000
72	1350.86720000	4.50730000	0.00000000
73	1362.59230000	5.89410000	0.00000000
74	1371.39860000	7.58860000	0.00000000
75	1376.95220000	0.57120000	0.00000000
76	1397.04600000	0.24680000	0.00000000
77	1404.57710000	12.30090000	0.00000000
78	1406.48410000	9.05350000	0.00000000
79	1438.40360000	13.31880000	0.00000000
80	1493.32620000	4.37170000	0.00000000
81	1496.96970000	6.96820000	0.00000000
82	1503.00850000	5.43970000	0.00000000
83	1505.23050000	2.37840000	0.00000000
84	1513.51090000	14.47600000	0.00000000
85	1514.60360000	10.76690000	0.00000000
86	1516.70150000	19.54540000	0.00000000
87	1538.33420000	3.24100000	0.00000000
88	1541.54770000	4.72090000	0.00000000
89	1630.71270000	2.42030000	0.00000000
90	1647.99170000	2.92780000	0.00000000
91	1772.81060000	193.25990000	0.00000000
92	2303.78330000	487.21600000	0.00000000
93	3069.57060000	6.44240000	0.00000000
94	3073.73870000	10.82350000	0.00000000
95	3075.96040000	0.81090000	0.00000000
96	3080.23600000	4.71210000	0.00000000
97	3084.25760000	3.48720000	0.00000000
98	3086.78280000	8.10400000	0.00000000
99	3097.48360000	7.24690000	0.00000000
100	3114.44310000	1.35390000	0.00000000
101	3120.67770000	2.27550000	0.00000000
102	3123.63840000	1.60580000	0.00000000
103	3127.68940000	0.42310000	0.00000000
104	3138.75090000	4.40870000	0.00000000
105	3151.58220000	1.65700000	0.00000000
106	3154.47110000	3.11690000	0.00000000
107	3166.57080000	6.07390000	0.00000000
108	3169.90830000	6.13050000	0.00000000
109	3188.15530000	0.93920000	0.00000000
110	3197.70230000	4.40330000	0.00000000
111	3206.81750000	1.68470000	0.00000000

S37. CALCULATIONS ON 9 (ISOMER 2)

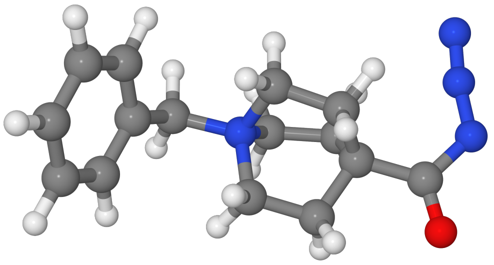

Route	:	# opt freq b3lyp/cc-pvtz empiricaldispersion=gd3bj pop=regular geom=connectivity int=ultrafine
SMILES	:	c1ccc(cc1)C[N]23CCC(CC2)(CC3)C(=O)N=[N+]=[N-]
Formula	:	$C_{15}H_{19}N_4O^+$
Charge	:	1
Multiplicity	:	1
Energy	:	-877.36310278 a.u.
Gibbs Energy	:	-877.07627600 a.u.

Cartesian Co-ordinates (XYZ format)

39

C	-1.73005998	1.37020302	-0.85158402
C	-1.76779699	0.18385100	0.13461700
C	-0.28021801	1.86541402	-0.97823900
H	-2.10376000	1.08576000	-1.83104002
H	-2.35610700	2.18298411	-0.48861700
H	-0.00883100	2.06689692	-2.01078510
H	-0.09904200	2.76437998	-0.39464399
C	-1.01840901	-1.00816405	-0.49371201
H	-0.85080498	-1.76693106	0.26887700
H	-1.57815301	-1.48196101	-1.29449797
C	0.31873301	-0.51205403	-1.06511903
H	1.13166404	-1.19575906	-0.84808803
H	0.27426299	-0.35589600	-2.14070296
C	0.50780702	0.72967702	1.04073405
H	1.09000599	-0.12648199	1.36561406
H	0.95234001	1.62333000	1.46894598
C	-0.98134398	0.60263801	1.38725305
H	-1.11580205	-0.13160700	2.17577791
H	-1.37413394	1.54270804	1.76839304
N	0.68146902	0.82192600	-0.45850199
C	2.10651898	1.23824596	-0.84085101
C	-3.20140290	-0.17484599	0.55581498
O	-3.54839993	-0.12980901	1.70185602
N	-4.15632296	-0.56055200	-0.41752699
N	-3.87625194	-0.64954501	-1.61630797
N	-3.77057409	-0.77544397	-2.72864008
H	2.21388006	2.25352192	-0.46525300
H	2.11070204	1.27051198	-1.92813301
C	3.18896198	0.34529299	-0.32030499
C	3.65452504	-0.72086698	-1.09181499
C	3.77506590	0.59092599	0.92221498
C	4.66439819	-1.54570603	-0.61631203

H	3.24374199	-0.89532000	-2.07798696
C	4.78428793	-0.23341900	1.39866805
H	3.45756888	1.43965399	1.51359200
C	5.22480488	-1.30665600	0.63274002
H	5.02215481	-2.36410499	-1.22443199
H	5.23537207	-0.03133100	2.35953808
H	6.01527977	-1.94472396	1.00128603

Frequencies

Mode	IR frequency	IR intensity	Raman intensity
1	28.49500000	0.27450000	0.00000000
2	58.42610000	1.21390000	0.00000000
3	63.57910000	0.54060000	0.00000000
4	76.04970000	1.68010000	0.00000000
5	96.61350000	0.03850000	0.00000000
6	105.81340000	0.40140000	0.00000000
7	135.43790000	1.32830000	0.00000000
8	145.21050000	3.83410000	0.00000000
9	181.47440000	3.79070000	0.00000000
10	222.96780000	1.26810000	0.00000000
11	237.99920000	0.80890000	0.00000000
12	247.82310000	0.27860000	0.00000000
13	321.15860000	0.37170000	0.00000000
14	336.66950000	1.88750000	0.00000000
15	348.56790000	6.88340000	0.00000000
16	369.84150000	1.42340000	0.00000000
17	405.09020000	0.35120000	0.00000000
18	418.57610000	0.02320000	0.00000000
19	423.47480000	0.15450000	0.00000000
20	461.18690000	2.51540000	0.00000000
21	472.96250000	5.53880000	0.00000000
22	539.16710000	0.68970000	0.00000000
23	545.90640000	0.65190000	0.00000000
24	574.29680000	4.03530000	0.00000000
25	581.66790000	5.64150000	0.00000000
26	615.29570000	3.66350000	0.00000000
27	638.99520000	0.05230000	0.00000000
28	644.06100000	12.80840000	0.00000000
29	677.85630000	20.80080000	0.00000000
30	708.56210000	1.33740000	0.00000000
31	720.68650000	6.57880000	0.00000000
32	724.61370000	39.49290000	0.00000000
33	786.53520000	35.77640000	0.00000000
34	796.42890000	1.24210000	0.00000000
35	834.84090000	6.90370000	0.00000000
36	839.85530000	4.09340000	0.00000000
37	848.04930000	25.48310000	0.00000000
38	852.60700000	2.86190000	0.00000000
39	869.23060000	0.07580000	0.00000000
40	906.99890000	1.84380000	0.00000000
41	931.87800000	93.23150000	0.00000000
42	955.64040000	1.35010000	0.00000000
43	991.45610000	4.01410000	0.00000000
44	992.60640000	2.27960000	0.00000000
45	1004.10880000	9.07470000	0.00000000
46	1006.52350000	0.19540000	0.00000000
47	1015.47680000	4.39440000	0.00000000
48	1018.06000000	3.68730000	0.00000000
49	1028.31650000	3.69530000	0.00000000
50	1037.03430000	0.05970000	0.00000000
51	1046.43280000	33.75210000	0.00000000
52	1054.50620000	2.79580000	0.00000000
53	1056.87090000	3.70080000	0.00000000
54	1060.63280000	5.21600000	0.00000000
55	1072.12120000	11.15670000	0.00000000
56	1122.51240000	5.30310000	0.00000000
57	1167.27480000	121.03950000	0.00000000
58	1190.94530000	2.03370000	0.00000000
59	1196.38900000	0.97890000	0.00000000
60	1200.65830000	8.66660000	0.00000000

61	1211.01210000	2.15250000	0.00000000
62	1214.97300000	0.65080000	0.00000000
63	1240.77400000	17.52600000	0.00000000
64	1257.11420000	0.71150000	0.00000000
65	1285.32550000	7.36930000	0.00000000
66	1299.63350000	3.10370000	0.00000000
67	1303.87600000	4.21760000	0.00000000
68	1328.50340000	16.32990000	0.00000000
69	1337.92600000	346.09590000	0.00000000
70	1342.93570000	3.88540000	0.00000000
71	1351.15430000	6.46060000	0.00000000
72	1354.08560000	0.70650000	0.00000000
73	1368.72150000	4.08390000	0.00000000
74	1371.90710000	8.81260000	0.00000000
75	1378.27010000	0.82550000	0.00000000
76	1397.30700000	0.92590000	0.00000000
77	1405.01980000	14.44070000	0.00000000
78	1407.56830000	9.50210000	0.00000000
79	1439.38500000	12.75870000	0.00000000
80	1493.40480000	4.56590000	0.00000000
81	1497.07960000	6.73810000	0.00000000
82	1502.09410000	7.54160000	0.00000000
83	1505.24740000	1.64410000	0.00000000
84	1513.99870000	4.72480000	0.00000000
85	1514.76600000	21.80510000	0.00000000
86	1515.67630000	15.26310000	0.00000000
87	1538.23600000	3.19130000	0.00000000
88	1541.22780000	7.11560000	0.00000000
89	1630.58000000	2.51180000	0.00000000
90	1647.52970000	3.24400000	0.00000000
91	1792.04380000	372.91100000	0.00000000
92	2256.50880000	361.54860000	0.00000000
93	3074.90600000	8.46930000	0.00000000
94	3076.50040000	4.27670000	0.00000000
95	3080.05380000	2.00540000	0.00000000
96	3084.68350000	0.89410000	0.00000000
97	3085.55900000	4.34050000	0.00000000
98	3088.62180000	8.32700000	0.00000000
99	3098.18600000	5.95480000	0.00000000
100	3120.81340000	0.81620000	0.00000000
101	3128.08790000	0.59150000	0.00000000
102	3129.87730000	3.07660000	0.00000000
103	3130.87460000	1.18780000	0.00000000
104	3142.36690000	3.61530000	0.00000000
105	3152.75900000	1.83840000	0.00000000
106	3155.56100000	2.35250000	0.00000000
107	3166.09980000	6.12820000	0.00000000
108	3170.17800000	5.97500000	0.00000000
109	3188.62290000	0.85290000	0.00000000
110	3198.11400000	3.90870000	0.00000000
111	3207.20840000	1.39610000	0.00000000

S38. CALCULATIONS ON TS 9 (ISOMER 1) ⟶ 9 (ISOMER 2)

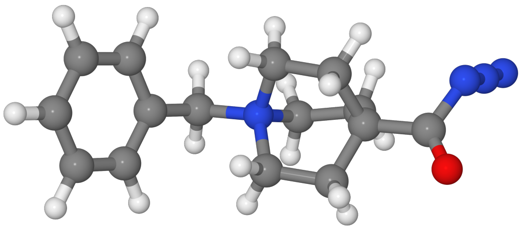

Route	:	# opt=(calcfc,ts,noeigentest) freq b3lyp/cc-pvtz empiricaldispersion=gd3bj pop=regular geom=connectivity int=ultrafine
SMILES	:	c1ccc(cc1)C[N]23CCC(CC2)(CC3)C(=O)N=[N+]=[N-]
Formula	:	$C_{15}H_{19}N_4O^+$
Charge	:	1
Multiplicity	:	1
Energy	:	-877.35669191 a.u.
Gibbs Energy	:	-877.07024800 a.u.

Cartesian Co-ordinates (XYZ format)

39

C	-0.16602300	-0.99347198	-0.57982600
C	-0.39266899	0.43138599	-0.04188200
C	1.34439301	-1.22171497	-0.74658501
H	-0.58192003	-1.72892296	0.10533100
H	-0.65244901	-1.13048506	-1.54392099
H	1.65123498	-2.19922209	-0.38511500
H	1.66308999	-1.12391102	-1.78125703
C	0.14206600	0.47404301	1.40452898
H	0.15275000	1.50398099	1.75720704
H	-0.50722700	-0.09239100	2.06768203
C	1.55691504	-0.12267400	1.43492103
H	2.23894405	0.47623199	2.02790308
H	1.56842995	-1.14107597	1.81667399
C	1.93023098	1.15533996	-0.62865400
H	2.37054110	1.89155602	0.03575700
H	2.50764799	1.14093804	-1.54839396
C	0.43770400	1.40581703	-0.88541299
H	0.18415800	2.43051291	-0.62971002
H	0.19955300	1.27627802	-1.93993402
N	2.12353396	-0.19004600	0.03638300
C	3.59950900	-0.60396999	0.05362200
H	3.86561990	-0.74456197	-0.99178803
H	3.61632800	-1.57322502	0.54701698
C	-1.86225700	0.84081101	-0.04785300
O	-2.24633694	1.89932203	-0.44006199
N	-2.75417590	-0.07574300	0.62410200
N	-3.16182995	-1.06693304	0.02245000
N	-3.59937191	-1.99387896	-0.44229800
C	4.52161312	0.36379799	0.72689497
C	5.13528776	1.38327503	-0.00229500
C	4.81185389	0.23167101	2.08589506
C	5.99650621	2.27399492	0.62223798
H	4.95689678	1.47251296	-1.06584704

C	5.67348289	1.12189698	2.71144605
H	4.38183689	-0.58275199	2.65459609
C	6.26058197	2.14830995	1.98126602
H	6.47089100	3.05642390	0.04747500
H	5.89647198	1.00676095	3.76244211
H	6.93616009	2.83837891	2.46624088

Frequencies

Mode	IR frequency	IR intensity	Raman intensity
1	-88.18340000	2.21990000	0.00000000
2	19.29690000	0.22980000	0.00000000
3	40.71740000	0.20850000	0.00000000
4	56.13560000	1.10700000	0.00000000
5	78.88020000	2.02050000	0.00000000
6	107.14010000	3.42780000	0.00000000
7	132.08580000	0.07670000	0.00000000
8	147.54130000	1.95440000	0.00000000
9	167.85030000	0.48170000	0.00000000
10	223.47660000	1.11340000	0.00000000
11	233.70580000	0.38440000	0.00000000
12	246.43230000	0.41200000	0.00000000
13	311.69450000	0.45470000	0.00000000
14	325.95710000	1.39710000	0.00000000
15	347.22140000	7.74090000	0.00000000
16	371.45660000	1.59740000	0.00000000
17	401.86680000	0.39240000	0.00000000
18	418.40760000	0.02190000	0.00000000
19	422.22500000	0.05720000	0.00000000
20	464.98090000	0.56720000	0.00000000
21	473.57660000	5.61140000	0.00000000
22	539.81370000	0.29050000	0.00000000
23	546.32450000	0.21570000	0.00000000
24	553.16510000	10.44390000	0.00000000
25	567.78080000	0.43680000	0.00000000
26	593.73670000	6.99520000	0.00000000
27	633.47080000	2.93780000	0.00000000
28	638.99250000	0.14310000	0.00000000
29	658.89610000	14.69650000	0.00000000
30	706.09870000	2.92660000	0.00000000
31	724.52490000	43.71440000	0.00000000
32	785.52460000	40.59410000	0.00000000
33	788.36920000	9.48070000	0.00000000
34	802.60000000	2.66450000	0.00000000
35	836.84920000	6.55800000	0.00000000
36	842.17220000	3.56180000	0.00000000
37	848.19680000	29.69390000	0.00000000
38	855.31000000	1.82700000	0.00000000
39	869.61540000	0.10820000	0.00000000
40	903.45250000	68.56850000	0.00000000
41	913.94580000	121.88620000	0.00000000
42	955.65870000	1.51370000	0.00000000
43	985.48310000	10.16130000	0.00000000
44	994.00610000	1.29760000	0.00000000
45	999.36170000	7.91110000	0.00000000
46	1006.63180000	0.04470000	0.00000000
47	1014.87190000	2.38440000	0.00000000
48	1018.87530000	5.65260000	0.00000000
49	1028.28570000	2.39880000	0.00000000
50	1031.15330000	31.24200000	0.00000000
51	1037.06910000	0.27890000	0.00000000
52	1052.76800000	3.23050000	0.00000000
53	1056.52410000	2.01970000	0.00000000
54	1063.34490000	11.32120000	0.00000000
55	1069.86930000	7.72510000	0.00000000
56	1122.25810000	5.34340000	0.00000000
57	1161.88510000	132.80440000	0.00000000
58	1190.62490000	7.68810000	0.00000000
59	1196.56830000	0.34010000	0.00000000
60	1204.25650000	10.92390000	0.00000000

61	1209.93260000	7.77460000	0.00000000
62	1214.68760000	0.44860000	0.00000000
63	1240.47090000	16.55120000	0.00000000
64	1257.75760000	3.58260000	0.00000000
65	1286.76120000	0.45390000	0.00000000
66	1297.25350000	8.07550000	0.00000000
67	1302.09590000	1.80600000	0.00000000
68	1330.13650000	4.12520000	0.00000000
69	1338.18810000	3.54310000	0.00000000
70	1348.98510000	1.25370000	0.00000000
71	1351.64040000	34.69080000	0.00000000
72	1358.93620000	279.99790000	0.00000000
73	1363.60410000	4.49220000	0.00000000
74	1372.83040000	5.29010000	0.00000000
75	1376.88070000	0.69980000	0.00000000
76	1396.13940000	0.13010000	0.00000000
77	1404.22440000	11.39340000	0.00000000
78	1406.01620000	9.16940000	0.00000000
79	1438.84890000	13.71900000	0.00000000
80	1493.03720000	4.06050000	0.00000000
81	1497.05850000	7.38680000	0.00000000
82	1504.23470000	6.07620000	0.00000000
83	1505.85180000	3.90250000	0.00000000
84	1514.28080000	12.77860000	0.00000000
85	1515.28420000	9.63120000	0.00000000
86	1517.21610000	20.64400000	0.00000000
87	1538.18930000	3.24480000	0.00000000
88	1541.92670000	4.00560000	0.00000000
89	1630.61640000	2.47910000	0.00000000
90	1647.57130000	3.20350000	0.00000000
91	1820.80020000	283.04220000	0.00000000
92	2280.50250000	563.89050000	0.00000000
93	3069.95270000	8.52850000	0.00000000
94	3072.39680000	3.83710000	0.00000000
95	3074.73720000	8.49570000	0.00000000
96	3075.51800000	1.83550000	0.00000000
97	3085.17090000	1.81120000	0.00000000
98	3087.83570000	6.76630000	0.00000000
99	3098.18080000	6.40850000	0.00000000
100	3110.49560000	2.12940000	0.00000000
101	3118.87420000	2.41410000	0.00000000
102	3126.56160000	1.02610000	0.00000000
103	3127.87330000	0.73460000	0.00000000
104	3139.98680000	3.54700000	0.00000000
105	3152.48550000	1.54880000	0.00000000
106	3155.25570000	2.57430000	0.00000000
107	3166.44500000	6.16110000	0.00000000
108	3169.81100000	6.23910000	0.00000000
109	3188.64280000	0.85980000	0.00000000
110	3198.15130000	3.97320000	0.00000000
111	3207.21450000	1.42920000	0.00000000

S39. CALCULATIONS ON 15s

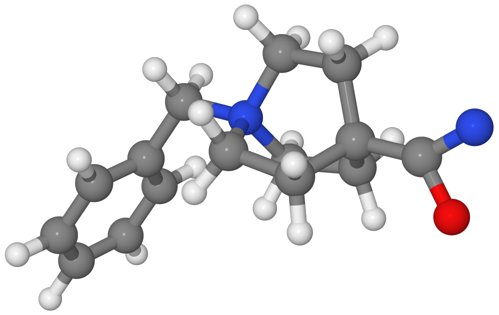

Route	:	# opt freq b3lyp/cc-pvtz empiricaldispersion=gd3bj pop=regular geom=connectivity int=ultrafine
SMILES	:	c1ccc(cc1)C[N]23CCC(CC2)(CC3)[C]4[N]O4
Formula	:	$C_{15}H_{19}N_2O^+$
Charge	:	1
Multiplicity	:	1
Energy	:	-767.75447410 a.u.
Gibbs Energy	:	-767.47756000 a.u.

Cartesian Co-ordinates (XYZ format)

37

C	-0.12168600	0.51689500	-1.23789895
C	0.30833700	-0.30837101	-0.01890200
C	-1.65549397	0.59990197	-1.25146699
H	0.31904101	1.51092100	-1.18807006
H	0.22793099	0.05313500	-2.15821600
H	-2.00941396	1.60637498	-1.45561004
H	-2.09862494	-0.07284700	-1.98144698
C	-0.02624700	0.48422199	1.25903106
H	0.13278601	-0.14233901	2.13403296
H	0.62931502	1.34707904	1.35551298
C	-1.48645902	0.95385098	1.17536604
H	-2.02313304	0.78619301	2.10235000
H	-1.56615901	2.00645208	0.91421998
C	-1.97414505	-1.28453600	0.29204801
H	-2.26338196	-1.51072598	1.31291497
H	-2.64754295	-1.81227398	-0.37678599
C	-0.50242299	-1.61870003	0.00561000
H	-0.11812400	-2.28557992	0.77397901
H	-0.40003899	-2.12985706	-0.95001799
N	-2.21944809	0.19424200	0.09406300
C	-3.71950507	0.51978600	0.10868600
H	-4.12255907	0.02931000	-0.77464199
H	-3.77533793	1.59603906	-0.03828900
C	1.75359499	-0.61997497	-0.05165900
O	2.50668192	-1.25487399	0.80326599
N	2.76791692	-0.46509999	-0.76911902
C	-4.44800901	0.10130400	1.34698200
C	-5.03927422	-1.16031897	1.42920005
C	-4.58144999	0.98523903	2.41922307
C	-5.72231388	-1.54316902	2.57460690
H	-4.98511791	-1.84082496	0.58950901

C	-5.26482201	0.60320801	3.56518006
H	-4.17065287	1.98439503	2.35180092
C	-5.82936287	-0.66415399	3.64626408
H	-6.18174314	-2.51981902	2.62604594
H	-5.36820889	1.29729104	4.38681984
H	-6.36688280	-0.96012002	4.53578901

Frequencies

Mode	IR frequency	IR intensity	Raman intensity
1	34.97630000	0.10890000	0.00000000
2	51.62670000	0.03950000	0.00000000
3	57.92370000	1.73900000	0.00000000
4	78.44850000	3.51300000	0.00000000
5	113.99930000	1.75270000	0.00000000
6	120.02910000	4.46630000	0.00000000
7	130.63570000	3.75300000	0.00000000
8	209.96150000	1.68820000	0.00000000
9	230.87480000	0.15750000	0.00000000
10	242.11000000	0.26980000	0.00000000
11	302.96720000	1.06780000	0.00000000
12	311.57480000	0.25330000	0.00000000
13	343.13520000	9.03870000	0.00000000
14	370.77550000	0.87720000	0.00000000
15	395.07350000	0.23600000	0.00000000
16	417.27010000	0.05890000	0.00000000
17	421.58380000	0.00800000	0.00000000
18	465.03380000	4.39300000	0.00000000
19	501.23910000	0.16580000	0.00000000
20	531.46170000	0.97350000	0.00000000
21	540.59670000	0.13740000	0.00000000
22	543.78300000	0.44250000	0.00000000
23	600.63190000	13.29670000	0.00000000
24	634.81040000	2.65360000	0.00000000
25	639.65950000	1.16250000	0.00000000
26	650.72180000	17.27200000	0.00000000
27	708.80690000	1.35770000	0.00000000
28	724.54310000	44.47180000	0.00000000
29	786.46180000	35.16680000	0.00000000
30	804.92850000	0.99100000	0.00000000
31	832.73770000	7.64380000	0.00000000
32	839.96870000	5.93220000	0.00000000
33	849.73050000	38.77200000	0.00000000
34	854.38370000	2.35360000	0.00000000
35	869.40640000	0.08450000	0.00000000
36	906.38880000	1.31820000	0.00000000
37	954.71130000	5.59080000	0.00000000
38	968.73690000	16.74980000	0.00000000
39	991.21090000	0.24150000	0.00000000
40	995.31360000	0.42680000	0.00000000
41	998.97310000	0.89310000	0.00000000
42	1007.03090000	0.02440000	0.00000000
43	1013.43410000	8.54450000	0.00000000
44	1017.32800000	2.85390000	0.00000000
45	1028.24420000	4.05060000	0.00000000
46	1037.76990000	0.00980000	0.00000000
47	1054.71440000	2.86510000	0.00000000
48	1056.27530000	0.88120000	0.00000000
49	1061.37520000	5.74110000	0.00000000
50	1068.29700000	22.45900000	0.00000000
51	1122.26510000	5.10710000	0.00000000
52	1135.95680000	9.87430000	0.00000000
53	1186.73580000	0.77460000	0.00000000
54	1192.60360000	7.88040000	0.00000000
55	1197.08480000	0.05130000	0.00000000
56	1205.50220000	0.05240000	0.00000000
57	1214.75140000	0.78310000	0.00000000
58	1240.53950000	19.34540000	0.00000000
59	1255.62000000	0.81980000	0.00000000
60	1275.53710000	16.28260000	0.00000000

61	1286.30510000	1.17650000	0.00000000
62	1301.65230000	6.60020000	0.00000000
63	1311.27730000	6.84250000	0.00000000
64	1333.02570000	7.24180000	0.00000000
65	1338.60810000	3.43070000	0.00000000
66	1349.29960000	1.79360000	0.00000000
67	1350.49020000	7.55200000	0.00000000
68	1364.29440000	4.83300000	0.00000000
69	1371.07010000	9.96600000	0.00000000
70	1377.30260000	0.56980000	0.00000000
71	1402.72310000	11.11220000	0.00000000
72	1405.76270000	7.44160000	0.00000000
73	1407.71530000	7.01890000	0.00000000
74	1439.48250000	9.50980000	0.00000000
75	1492.66570000	4.42490000	0.00000000
76	1497.11810000	7.66690000	0.00000000
77	1504.21060000	1.68670000	0.00000000
78	1505.40090000	4.55360000	0.00000000
79	1513.40850000	22.65100000	0.00000000
80	1515.00800000	7.70090000	0.00000000
81	1517.15740000	18.94300000	0.00000000
82	1538.09680000	3.11230000	0.00000000
83	1541.39750000	4.25400000	0.00000000
84	1630.47060000	2.52370000	0.00000000
85	1647.18800000	3.50640000	0.00000000
86	1822.63870000	70.63960000	0.00000000
87	3071.53540000	4.66360000	0.00000000
88	3073.08930000	5.36110000	0.00000000
89	3075.92140000	3.84120000	0.00000000
90	3077.04000000	2.84000000	0.00000000
91	3086.93420000	3.02640000	0.00000000
92	3089.39210000	4.55420000	0.00000000
93	3099.83890000	4.64400000	0.00000000
94	3110.65680000	1.44290000	0.00000000
95	3116.32310000	0.98250000	0.00000000
96	3117.90120000	2.16910000	0.00000000
97	3128.78150000	0.46700000	0.00000000
98	3140.91260000	2.81560000	0.00000000
99	3153.84190000	1.04870000	0.00000000
100	3156.53100000	2.13280000	0.00000000
101	3166.77300000	5.89280000	0.00000000
102	3168.97890000	6.32960000	0.00000000
103	3188.98280000	0.76060000	0.00000000
104	3198.50630000	3.59420000	0.00000000
105	3207.51820000	1.19050000	0.00000000

S40. CALCULATIONS ON 15s ⟶ 16s

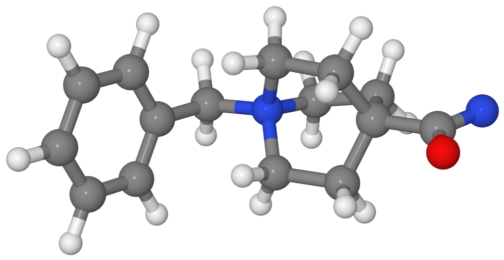

Route	:	# opt=(calcfc,ts) freq b3lyp/cc-pvtz empiricaldispersion=gd3bj pop=regular geom=connectivity int=ultrafine
SMILES	:	c1ccc(cc1)C[N]23CCC(CC2)(CC3)C(=O)[N]
Formula	:	$C_{15}H_{19}N_2O^+$
Charge	:	1
Multiplicity	:	1
Energy	:	-767.72979444 a.u.
Gibbs Energy	:	-767.45478700 a.u.

Cartesian Co-ordinates (XYZ format)

37

C	0.20725200	1.55977905	-0.08810300
C	0.90847600	0.19455400	-0.02141400
C	-1.29780102	1.30795896	-0.29305500
H	0.37784001	2.12098789	0.82846397
H	0.58255798	2.15962696	-0.91423100
H	-1.90733600	1.92620599	0.35979599
H	-1.60700095	1.48584294	-1.31991696
C	0.48187599	-0.51323998	1.27296901
H	0.86726201	-1.52935100	1.27503300
H	0.89490098	-0.00369700	2.14106894
C	-1.05452096	-0.49894300	1.35159504
H	-1.45487499	-1.46966302	1.62225294
H	-1.42488396	0.24027000	2.05787206
C	-1.01202297	-1.01193404	-1.04881203
H	-1.14743805	-2.03683996	-0.72008300
H	-1.57799304	-0.86766601	-1.96446800
C	0.47019500	-0.64265198	-1.23102105
H	1.07204902	-1.54535604	-1.29781103
H	0.61736202	-0.07331100	-2.14706707
N	-1.64304900	-0.13371700	0.00915100
C	-3.17094803	-0.27741200	0.01741000
H	-3.51248097	0.38687700	0.80807102
H	-3.49745893	0.12670900	-0.93827897
C	2.47388792	0.18406799	-0.03186800
O	3.15508509	-0.85716701	-0.03725100
N	2.93635607	1.36546600	-0.01871600
C	-3.67424607	-1.67168999	0.22242101
C	-3.91422391	-2.50626206	-0.87033099
C	-3.94996691	-2.13996100	1.50842702
C	-4.38882303	-3.79584289	-0.67892498
H	-3.74984598	-2.14361596	-1.87657797
C	-4.42499685	-3.42964506	1.70069396
H	-3.81537008	-1.48861694	2.36236191

C	-4.63763618	-4.26087904	0.60733002
H	-4.57688808	-4.43217182	-1.53172100
H	-4.64138079	-3.78027511	2.69957209
H	-5.01325989	-5.26318693	0.75586098

Frequencies

Mode	IR frequency	IR intensity	Raman intensity
1	-947.31140000	68.97170000	0.00000000
2	33.78050000	0.01540000	0.00000000
3	51.96230000	0.39710000	0.00000000
4	59.06170000	0.94850000	0.00000000
5	82.28840000	2.12550000	0.00000000
6	118.42290000	0.14520000	0.00000000
7	129.94750000	3.42050000	0.00000000
8	154.64530000	3.24360000	0.00000000
9	212.31580000	3.30420000	0.00000000
10	237.50890000	0.09550000	0.00000000
11	243.18210000	0.16220000	0.00000000
12	303.69010000	2.47170000	0.00000000
13	321.99100000	0.13710000	0.00000000
14	351.15170000	3.72710000	0.00000000
15	377.07470000	0.61230000	0.00000000
16	391.50530000	0.59120000	0.00000000
17	417.39590000	0.03850000	0.00000000
18	421.89970000	0.01260000	0.00000000
19	465.88760000	4.99460000	0.00000000
20	495.60190000	0.27420000	0.00000000
21	536.45490000	0.24380000	0.00000000
22	540.89410000	0.74350000	0.00000000
23	585.80990000	10.84830000	0.00000000
24	638.79110000	0.09340000	0.00000000
25	642.54890000	10.37820000	0.00000000
26	689.47920000	5.20090000	0.00000000
27	701.64860000	2.96950000	0.00000000
28	724.61040000	45.96550000	0.00000000
29	786.26120000	36.33160000	0.00000000
30	806.69070000	1.74530000	0.00000000
31	823.77160000	17.47790000	0.00000000
32	834.98560000	2.13920000	0.00000000
33	842.39730000	6.33940000	0.00000000
34	855.29100000	2.52350000	0.00000000
35	869.59900000	0.10680000	0.00000000
36	906.48520000	1.30940000	0.00000000
37	921.63220000	56.39770000	0.00000000
38	955.78280000	1.04720000	0.00000000
39	985.20360000	0.40550000	0.00000000
40	988.31900000	2.91260000	0.00000000
41	992.95380000	0.39620000	0.00000000
42	1005.75750000	6.59390000	0.00000000
43	1007.09180000	0.32940000	0.00000000
44	1016.19990000	1.03030000	0.00000000
45	1028.21210000	4.62180000	0.00000000
46	1037.65410000	0.02700000	0.00000000
47	1043.28700000	10.16890000	0.00000000
48	1049.17520000	3.23260000	0.00000000
49	1053.06760000	1.71760000	0.00000000
50	1056.48230000	1.12000000	0.00000000
51	1078.96840000	19.18090000	0.00000000
52	1122.36290000	5.07520000	0.00000000
53	1180.79490000	1.22040000	0.00000000
54	1187.83870000	3.36840000	0.00000000
55	1197.05190000	0.09430000	0.00000000
56	1205.08560000	0.31970000	0.00000000
57	1214.79830000	1.14890000	0.00000000
58	1240.44990000	18.23610000	0.00000000
59	1250.98800000	5.49560000	0.00000000
60	1269.18210000	5.87710000	0.00000000

61	1280.72970000	3.58990000	0.00000000
62	1299.63960000	7.58380000	0.00000000
63	1307.18960000	7.60010000	0.00000000
64	1329.28920000	1.14410000	0.00000000
65	1337.73250000	2.36520000	0.00000000
66	1345.13990000	7.43110000	0.00000000
67	1348.73660000	1.03250000	0.00000000
68	1362.88990000	4.41390000	0.00000000
69	1368.85150000	11.37210000	0.00000000
70	1376.09760000	1.54430000	0.00000000
71	1386.01180000	11.04120000	0.00000000
72	1399.90020000	14.31910000	0.00000000
73	1403.85620000	12.78930000	0.00000000
74	1437.61620000	9.82330000	0.00000000
75	1492.97760000	4.55230000	0.00000000
76	1497.01320000	7.60200000	0.00000000
77	1503.95090000	2.71700000	0.00000000
78	1505.18210000	5.23220000	0.00000000
79	1512.56370000	26.11330000	0.00000000
80	1513.71230000	11.94340000	0.00000000
81	1517.13170000	16.19340000	0.00000000
82	1526.46210000	44.51420000	0.00000000
83	1537.99720000	2.89720000	0.00000000
84	1540.85930000	1.09410000	0.00000000
85	1630.06150000	2.51880000	0.00000000
86	1646.94770000	5.12260000	0.00000000
87	3072.66950000	4.05790000	0.00000000
88	3073.08340000	3.81690000	0.00000000
89	3076.06090000	4.00130000	0.00000000
90	3080.29120000	0.74010000	0.00000000
91	3087.06380000	3.56410000	0.00000000
92	3089.51170000	5.44010000	0.00000000
93	3098.88040000	4.09920000	0.00000000
94	3111.75080000	1.32220000	0.00000000
95	3122.38520000	1.77090000	0.00000000
96	3125.28200000	2.48280000	0.00000000
97	3128.82050000	0.48230000	0.00000000
98	3141.39810000	2.62530000	0.00000000
99	3154.02860000	2.64810000	0.00000000
100	3157.60110000	1.11210000	0.00000000
101	3167.00230000	5.75350000	0.00000000
102	3169.19620000	6.26290000	0.00000000
103	3189.06580000	0.73080000	0.00000000
104	3198.56850000	3.58440000	0.00000000
105	3207.58430000	1.31810000	0.00000000

S41. CALCULATIONS ON TS 9 ⟶ 16s

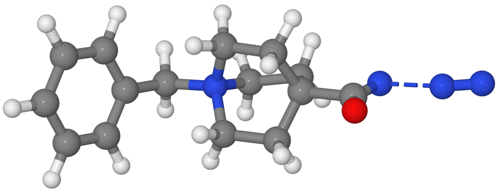

Route	:	# opt=(calcfc,ts,noeigentest) freq b3lyp/cc-pvtz empiricaldispersion=gd3bj pop=regular geom=connectivity int=ultrafine
SMILES	:	c1ccc(cc1)C[N]23CCC(CC2)(CC3)C(=O)[N].[N]=[N]
Formula	:	$C_{15}H_{19}N_4O^+$
Charge	:	1
Multiplicity	:	1
Energy	:	-877.32733833 a.u.
Gibbs Energy	:	-877.04701100 a.u.

Cartesian Co-ordinates (XYZ format)

39

C	-0.24903101	-1.41360903	0.09314900
C	0.25023100	0.03666700	0.03494700
C	-1.77904296	-1.39284396	0.27553999
H	0.01972300	-1.94229901	-0.81790203
H	0.20068100	-1.94970596	0.92333901
H	-2.27983809	-2.08826590	-0.39247099
H	-2.07413006	-1.62589395	1.29573596
C	-0.27299100	0.67760998	-1.25608695
H	-0.04314700	1.73977005	-1.24927402
H	0.21863700	0.24008900	-2.12264895
C	-1.78875101	0.43916699	-1.35302806
H	-2.32448912	1.34229100	-1.62333500
H	-2.04161906	-0.34194899	-2.06617689
C	-1.84469497	0.93108201	1.05313396
H	-2.12989998	1.92830706	0.73485899
H	-2.39117289	0.69249898	1.96094000
C	-0.32477799	0.78821898	1.24142504
H	0.12896100	1.77239001	1.31587994
H	-0.09973100	0.24351700	2.15688491
N	-2.33165503	-0.01737400	-0.01949800
C	-3.86131907	-0.09966800	-0.04291400
H	-4.13488293	-0.55466098	0.90652603
H	-4.09490299	-0.80097097	-0.84084702
C	1.84845901	0.38687599	0.04131100
O	2.21390104	1.53818500	0.03961300
N	2.28260303	-0.82328802	0.03157400
N	4.11858606	-0.62318897	0.00857200
N	5.16029501	-0.95118099	0.00072400
C	-4.56493521	1.20616806	-0.24490000
C	-4.93415213	1.98918200	0.84972298
C	-4.89737797	1.63694894	-1.53032899
C	-5.59327698	3.19546700	0.66125602
H	-4.72380686	1.64903903	1.85521305
C	-5.55716610	2.84313798	-1.71998203
H	-4.65967083	1.01896703	-2.38653207

C	-5.89903498	3.62676501	-0.62437397
H	-5.87918377	3.79168797	1.51588798
H	-5.81506491	3.16431093	-2.71894407
H	-6.41764021	4.56347418	-0.77081800

Frequencies

Mode	IR frequency	IR intensity	Raman intensity
1	-483.99030000	275.77000000	0.00000000
2	25.97090000	0.33030000	0.00000000
3	41.82230000	0.08840000	0.00000000
4	46.38110000	0.04730000	0.00000000
5	70.07370000	0.55690000	0.00000000
6	78.64230000	1.12390000	0.00000000
7	85.91620000	0.81790000	0.00000000
8	119.37020000	0.06140000	0.00000000
9	149.03950000	1.42420000	0.00000000
10	154.55140000	0.78680000	0.00000000
11	209.76280000	1.24560000	0.00000000
12	225.71110000	1.20630000	0.00000000
13	234.31020000	2.66850000	0.00000000
14	260.80390000	37.91840000	0.00000000
15	276.74940000	2.60160000	0.00000000
16	294.25210000	1.74590000	0.00000000
17	317.00670000	5.69200000	0.00000000
18	347.29270000	0.58990000	0.00000000
19	386.71500000	1.61650000	0.00000000
20	402.62810000	2.34520000	0.00000000
21	414.26820000	0.45890000	0.00000000
22	418.51920000	0.02020000	0.00000000
23	423.31590000	0.03760000	0.00000000
24	469.86990000	2.50220000	0.00000000
25	538.79970000	0.07120000	0.00000000
26	543.34830000	0.29070000	0.00000000
27	581.61110000	13.96800000	0.00000000
28	632.55100000	4.68020000	0.00000000
29	639.08370000	0.08330000	0.00000000
30	697.33400000	7.08190000	0.00000000
31	719.06540000	5.85690000	0.00000000
32	724.13330000	42.84420000	0.00000000
33	743.81520000	15.35570000	0.00000000
34	786.53960000	31.83050000	0.00000000
35	809.69830000	0.74440000	0.00000000
36	831.36570000	18.31160000	0.00000000
37	835.82480000	3.19170000	0.00000000
38	843.88080000	5.49420000	0.00000000
39	856.57110000	2.74720000	0.00000000
40	869.08820000	0.11680000	0.00000000
41	906.34330000	1.18410000	0.00000000
42	917.22740000	63.96240000	0.00000000
43	954.90380000	1.08010000	0.00000000
44	991.23290000	1.33220000	0.00000000
45	993.68040000	0.06300000	0.00000000
46	996.97040000	1.89480000	0.00000000
47	1005.99130000	0.00580000	0.00000000
48	1011.37980000	6.33100000	0.00000000
49	1018.38600000	1.64680000	0.00000000
50	1028.41260000	4.10070000	0.00000000
51	1036.27940000	0.11490000	0.00000000
52	1040.13630000	7.49810000	0.00000000
53	1052.71700000	1.90150000	0.00000000
54	1056.49280000	1.97210000	0.00000000
55	1057.14380000	1.92980000	0.00000000
56	1078.48930000	19.33120000	0.00000000
57	1122.30150000	5.15600000	0.00000000
58	1176.91950000	7.56590000	0.00000000
59	1188.69640000	0.66360000	0.00000000
60	1196.55990000	0.07370000	0.00000000

61	1206.16060000	0.17180000	0.00000000
62	1214.71080000	0.80710000	0.00000000
63	1227.35080000	11.75180000	0.00000000
64	1240.78970000	21.83910000	0.00000000
65	1256.41720000	0.07080000	0.00000000
66	1278.85960000	3.44480000	0.00000000
67	1298.51710000	5.11200000	0.00000000
68	1306.77740000	7.71090000	0.00000000
69	1330.68050000	2.35300000	0.00000000
70	1336.17420000	1.68720000	0.00000000
71	1345.82540000	7.34480000	0.00000000
72	1347.93580000	2.84480000	0.00000000
73	1363.30830000	6.43650000	0.00000000
74	1369.16320000	12.95230000	0.00000000
75	1376.94050000	0.45790000	0.00000000
76	1381.09210000	6.76150000	0.00000000
77	1402.09280000	12.74240000	0.00000000
78	1405.22110000	12.54780000	0.00000000
79	1436.99540000	9.93550000	0.00000000
80	1492.99450000	4.20880000	0.00000000
81	1497.03020000	7.62470000	0.00000000
82	1502.62330000	0.88880000	0.00000000
83	1506.57210000	2.57070000	0.00000000
84	1511.54740000	20.18610000	0.00000000
85	1514.88180000	12.86290000	0.00000000
86	1518.08600000	16.45570000	0.00000000
87	1538.25600000	3.20750000	0.00000000
88	1541.53030000	5.10340000	0.00000000
89	1630.67970000	2.42700000	0.00000000
90	1647.82890000	3.19030000	0.00000000
91	1860.17600000	286.75080000	0.00000000
92	2418.20280000	13.53210000	0.00000000
93	3072.01350000	5.73450000	0.00000000
94	3075.10210000	6.01480000	0.00000000
95	3078.65670000	1.08820000	0.00000000
96	3083.33030000	6.71940000	0.00000000
97	3086.95750000	1.51590000	0.00000000
98	3089.10420000	6.71710000	0.00000000
99	3097.50160000	7.26490000	0.00000000
100	3123.88940000	1.07920000	0.00000000
101	3123.98560000	0.61500000	0.00000000
102	3126.98180000	3.19230000	0.00000000
103	3129.42590000	0.75040000	0.00000000
104	3142.40840000	4.40900000	0.00000000
105	3152.96700000	1.98230000	0.00000000
106	3156.00540000	1.80300000	0.00000000
107	3167.17650000	6.09530000	0.00000000
108	3169.45590000	6.54800000	0.00000000
109	3188.34070000	0.93490000	0.00000000
110	3197.86320000	4.35970000	0.00000000
111	3207.01490000	1.68100000	0.00000000

S42. CALCULATIONS ON 16s

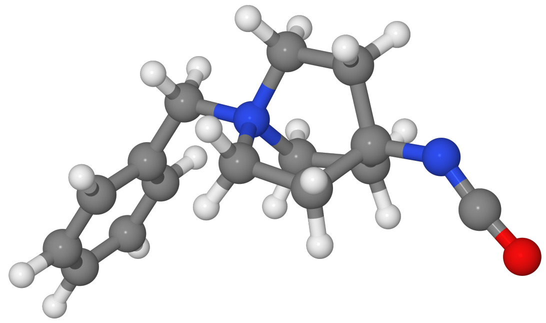

Route	:	# opt freq b3lyp/cc-pvtz empiricaldispersion=gd3bj pop=regular geom=connectivity int=ultrafine
SMILES	:	c1ccc(cc1)C[N]23CCC(CC2)(CC3)[N][C]=O
Formula	:	$C_{15}H_{19}N_2O^+$
Charge	:	1
Multiplicity	:	1
Energy	:	-767.88491606 a.u.
Gibbs Energy	:	-767.60663700 a.u.

Cartesian Co-ordinates (XYZ format)

37

C	0.05802100	0.44372201	-1.15333295
C	0.31375900	-0.34658900	0.13925400
C	-1.45486796	0.61982298	-1.35533905
H	0.55384600	1.41172302	-1.09283495
H	0.48440999	-0.09151800	-1.99852002
H	-1.72056103	1.64023602	-1.60759497
H	-1.84808803	-0.03971300	-2.12535405
C	-0.14541100	0.51741898	1.32470095
H	-0.09832800	-0.07012600	2.23959398
H	0.52585101	1.36534798	1.44538999
C	-1.57313299	1.02612102	1.06680202
H	-2.22134590	0.88263702	1.92641795
H	-1.59866095	2.07619190	0.79533702
C	-2.03664899	-1.21199501	0.17446201
H	-2.46907902	-1.39560902	1.15498698
H	-2.63949895	-1.73080206	-0.56562102
C	-0.55619699	-1.60983896	0.10465000
H	-0.30106401	-2.24871206	0.94655502
H	-0.34310600	-2.16932392	-0.80417502
N	-2.19576001	0.27056101	-0.08506000
C	-3.69139504	0.58221298	-0.21422499
H	-4.03130722	-0.01140300	-1.05987298
H	-4.14246082	0.18208000	0.69115603
N	1.68944800	-0.73758799	0.26394400
C	2.74036694	-0.14515799	0.32867900
O	3.80545211	0.31569400	0.40025800
C	-4.02648783	2.02950001	-0.39684600
C	-4.13917685	2.57164598	-1.67822301
C	-4.27240896	2.84469795	0.70900297
C	-4.45972919	3.91103506	-1.85013902

H	-3.99813199	1.94158697	-2.54692602
C	-4.59248590	4.18393278	0.53824103
H	-4.23355818	2.42975807	1.70768702
C	-4.67948914	4.71977997	-0.74151301
H	-4.55098104	4.31904316	-2.84652400
H	-4.78685522	4.80501890	1.40084505
H	-4.93519211	5.76126289	-0.87472302

Frequencies

Mode	IR frequency	IR intensity	Raman intensity
1	20.13430000	0.05180000	0.00000000
2	38.11810000	0.09290000	0.00000000
3	51.28390000	0.76220000	0.00000000
4	86.71660000	1.04150000	0.00000000
5	90.80270000	0.99680000	0.00000000
6	102.54170000	0.22220000	0.00000000
7	194.41160000	7.43320000	0.00000000
8	213.68960000	2.60050000	0.00000000
9	244.03680000	0.22180000	0.00000000
10	267.83460000	0.71570000	0.00000000
11	274.21510000	2.35050000	0.00000000
12	351.90410000	0.05230000	0.00000000
13	387.42490000	0.86820000	0.00000000
14	411.20470000	7.60790000	0.00000000
15	418.33290000	0.10610000	0.00000000
16	422.57340000	0.05260000	0.00000000
17	429.53260000	1.59660000	0.00000000
18	440.38070000	4.77080000	0.00000000
19	491.51110000	4.50420000	0.00000000
20	536.54480000	0.88700000	0.00000000
21	538.55460000	0.07950000	0.00000000
22	595.26480000	7.50380000	0.00000000
23	603.69120000	19.36210000	0.00000000
24	639.04940000	0.08200000	0.00000000
25	641.01510000	7.58000000	0.00000000
26	687.09720000	23.90640000	0.00000000
27	717.15730000	2.52360000	0.00000000
28	724.46210000	47.18870000	0.00000000
29	786.48900000	31.85280000	0.00000000
30	811.44160000	0.60560000	0.00000000
31	833.12320000	3.48390000	0.00000000
32	840.32280000	4.00430000	0.00000000
33	854.07790000	13.84280000	0.00000000
34	856.28550000	1.85470000	0.00000000
35	869.31520000	0.08240000	0.00000000
36	905.07510000	0.53310000	0.00000000
37	954.51420000	10.08260000	0.00000000
38	967.66120000	63.63090000	0.00000000
39	989.36970000	6.38850000	0.00000000
40	996.16430000	2.11380000	0.00000000
41	1002.77390000	3.48650000	0.00000000
42	1006.44190000	0.04410000	0.00000000
43	1017.10620000	14.31150000	0.00000000
44	1019.77090000	5.26160000	0.00000000
45	1028.31980000	3.78990000	0.00000000
46	1036.94390000	0.00710000	0.00000000
47	1054.47880000	0.95670000	0.00000000
48	1055.18800000	7.09460000	0.00000000
49	1057.56640000	1.69420000	0.00000000
50	1066.01490000	43.53200000	0.00000000
51	1102.54250000	4.98850000	0.00000000
52	1122.18190000	5.79810000	0.00000000
53	1192.18820000	3.38960000	0.00000000
54	1196.80390000	0.06650000	0.00000000
55	1200.47590000	0.45600000	0.00000000
56	1203.77300000	9.89480000	0.00000000
57	1214.77240000	0.74350000	0.00000000
58	1240.85730000	19.27040000	0.00000000
59	1257.87530000	0.12080000	0.00000000
60	1278.09470000	3.62730000	0.00000000

61	1296.19680000	5.72150000	0.00000000
62	1304.68470000	6.01050000	0.00000000
63	1329.50200000	2.93620000	0.00000000
64	1332.32260000	1.67220000	0.00000000
65	1344.74580000	6.93810000	0.00000000
66	1347.65710000	2.54410000	0.00000000
67	1363.43410000	8.05420000	0.00000000
68	1371.13350000	15.72440000	0.00000000
69	1377.76200000	0.10820000	0.00000000
70	1387.39920000	19.84220000	0.00000000
71	1404.77530000	8.82090000	0.00000000
72	1407.33420000	7.13840000	0.00000000
73	1439.47690000	10.81640000	0.00000000
74	1486.90790000	57.67380000	0.00000000
75	1493.19270000	4.80180000	0.00000000
76	1496.96010000	6.92940000	0.00000000
77	1503.04390000	3.96120000	0.00000000
78	1503.58100000	2.37120000	0.00000000
79	1513.05440000	22.45640000	0.00000000
80	1514.61460000	6.11710000	0.00000000
81	1517.44080000	6.70820000	0.00000000
82	1538.19170000	3.18090000	0.00000000
83	1542.65120000	14.32870000	0.00000000
84	1630.56220000	2.44490000	0.00000000
85	1647.60840000	3.30060000	0.00000000
86	2349.25610000	1316.29990000	0.00000000
87	3066.31750000	5.95250000	0.00000000
88	3070.62970000	8.41250000	0.00000000
89	3075.16240000	11.10710000	0.00000000
90	3077.18160000	1.87720000	0.00000000
91	3085.33470000	4.20860000	0.00000000
92	3088.48300000	4.63630000	0.00000000
93	3098.15530000	5.38130000	0.00000000
94	3110.99770000	2.62610000	0.00000000
95	3115.08370000	3.62610000	0.00000000
96	3117.67360000	2.05210000	0.00000000
97	3128.68150000	0.27270000	0.00000000
98	3139.28220000	4.46090000	0.00000000
99	3152.16710000	1.26510000	0.00000000
100	3155.14330000	3.39020000	0.00000000
101	3167.23320000	5.70150000	0.00000000
102	3169.07850000	6.19980000	0.00000000
103	3188.53370000	0.83760000	0.00000000
104	3198.01820000	4.07770000	0.00000000
105	3207.14920000	1.44200000	0.00000000

S43. CALCULATIONS ON 9 (ISOMER 1; TRIPLET)

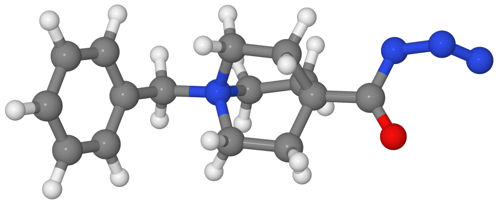

Route	:	# opt freq b3lyp/cc-pvtz empiricaldispersion=gd3bj pop=regular geom=connectivity int=ultrafine
SMILES	:	c1ccc(cc1)C[N]23CCC(CC2)(CC3)C(=O)N=[N+]=[N-]
Formula	:	$C_{15}H_{19}N_4O^{+,3}$
Charge	:	1
Multiplicity	:	3
Energy	:	-877.27904203 a.u.
Gibbs Energy	:	-876.99966000 a.u.

Cartesian Co-ordinates (XYZ format)

39

C	-1.77340198	1.38720500	-0.83695197
C	-1.80126202	0.21213000	0.16194500
C	-0.32108599	1.86072397	-0.99959099
H	-2.18981504	1.07294297	-1.79088700
H	-2.38227010	2.21200109	-0.47183800
H	-0.06150100	2.03189206	-2.04068208
H	-0.11938100	2.77185893	-0.44187200
C	-1.09306705	-0.98468697	-0.49837801
H	-0.95245701	-1.77841401	0.23400900
H	-1.69696605	-1.38960803	-1.30755997
C	0.26131901	-0.52130598	-1.05582702
H	1.06062996	-1.21217203	-0.81242502
H	0.23862700	-0.38340101	-2.13436389
C	0.47753099	0.74896300	1.03594899
H	1.06759298	-0.09921000	1.36723602
H	0.92335403	1.65145195	1.44375706
C	-1.00730205	0.61949599	1.40673995
H	-1.12693906	-0.12142600	2.19335198
H	-1.39378595	1.55734801	1.79995501
N	0.63630599	0.81823403	-0.46720099
C	2.06179905	1.21889997	-0.86422497
C	-3.23314810	-0.13473800	0.53544497
O	-3.69451809	-0.00359400	1.64337003
N	-3.99810505	-0.54345602	-0.58274698
N	-5.10140896	-1.33704698	-0.24011099
N	-6.12645912	-0.85592300	0.07265200
H	2.17966890	2.23797011	-0.50233603
H	2.05810308	1.23765004	-1.95176005
C	3.14221907	0.32502300	-0.34061101
C	3.59124708	-0.75728297	-1.09930098
C	3.74346304	0.58607697	0.89149302
C	4.59971714	-1.58215404	-0.62090802
H	3.16857195	-0.94489902	-2.07796001
C	4.75135517	-0.23823600	1.37085998

H	3.43880796	1.44688702	1.47212398
C	5.17538214	-1.32723296	0.61803001
H	4.94458389	-2.41319108	-1.21926105
H	5.21426678	-0.02395500	2.32343602
H	5.96487188	-1.96540105	0.98851198

Frequencies

Mode	IR frequency	IR intensity	Raman intensity
1	27.37740000	0.23750000	0.00000000
2	35.43780000	0.84550000	0.00000000
3	46.76640000	0.91610000	0.00000000
4	62.33840000	0.34000000	0.00000000
5	63.87910000	0.77930000	0.00000000
6	91.16390000	0.78920000	0.00000000
7	120.73270000	0.40340000	0.00000000
8	122.70060000	1.15300000	0.00000000
9	152.70610000	2.61870000	0.00000000
10	209.64050000	1.98260000	0.00000000
11	226.41490000	1.18250000	0.00000000
12	237.71390000	0.40990000	0.00000000
13	279.63130000	1.94450000	0.00000000
14	302.92190000	0.42170000	0.00000000
15	330.26660000	2.80630000	0.00000000
16	346.40650000	5.62570000	0.00000000
17	367.11870000	1.32100000	0.00000000
18	402.30010000	0.35070000	0.00000000
19	418.14810000	0.01260000	0.00000000
20	421.67010000	0.04300000	0.00000000
21	449.39160000	1.24350000	0.00000000
22	475.83960000	5.43180000	0.00000000
23	539.29700000	0.41090000	0.00000000
24	543.17080000	0.89950000	0.00000000
25	578.37830000	3.05710000	0.00000000
26	595.17860000	5.84710000	0.00000000
27	609.76950000	1.82580000	0.00000000
28	639.04800000	0.10270000	0.00000000
29	646.50880000	7.06470000	0.00000000
30	670.37640000	8.81930000	0.00000000
31	703.59420000	3.99950000	0.00000000
32	724.58760000	44.73240000	0.00000000
33	779.61180000	2.38620000	0.00000000
34	786.45080000	36.41420000	0.00000000
35	803.67280000	1.37980000	0.00000000
36	835.48090000	6.76470000	0.00000000
37	839.24460000	4.28190000	0.00000000
38	851.35740000	32.77340000	0.00000000
39	854.94680000	5.62210000	0.00000000
40	869.69090000	0.12920000	0.00000000
41	905.77860000	2.26130000	0.00000000
42	938.09380000	106.15710000	0.00000000
43	956.08030000	1.52300000	0.00000000
44	988.62110000	4.87740000	0.00000000
45	993.04320000	1.07720000	0.00000000
46	999.54180000	8.10070000	0.00000000
47	1006.62710000	0.05310000	0.00000000
48	1012.27830000	4.06490000	0.00000000
49	1018.18500000	3.44890000	0.00000000
50	1028.31130000	3.78410000	0.00000000
51	1037.01930000	0.03190000	0.00000000
52	1047.65850000	32.40000000	0.00000000
53	1052.76200000	8.13890000	0.00000000
54	1056.62100000	2.03760000	0.00000000
55	1063.54930000	6.85290000	0.00000000
56	1071.99180000	6.96370000	0.00000000
57	1122.26530000	5.18500000	0.00000000
58	1176.57650000	104.39780000	0.00000000
59	1189.46730000	4.14090000	0.00000000
60	1196.50750000	0.53240000	0.00000000

61	1205.60700000	0.76240000	0.00000000
62	1213.17880000	57.18840000	0.00000000
63	1214.79820000	3.13620000	0.00000000
64	1240.48940000	15.94470000	0.00000000
65	1258.39310000	5.37950000	0.00000000
66	1284.19470000	0.58370000	0.00000000
67	1299.90090000	7.23340000	0.00000000
68	1304.09300000	4.31200000	0.00000000
69	1329.96460000	5.20790000	0.00000000
70	1336.17220000	2.64170000	0.00000000
71	1347.46990000	0.80840000	0.00000000
72	1351.38960000	6.19030000	0.00000000
73	1363.10670000	6.57160000	0.00000000
74	1371.16770000	9.24660000	0.00000000
75	1377.13540000	0.44960000	0.00000000
76	1396.98710000	1.61970000	0.00000000
77	1404.60990000	11.36400000	0.00000000
78	1406.50860000	10.01490000	0.00000000
79	1438.60210000	10.52810000	0.00000000
80	1493.17590000	4.37720000	0.00000000
81	1497.00770000	6.75700000	0.00000000
82	1502.56290000	6.75050000	0.00000000
83	1504.40200000	1.20500000	0.00000000
84	1513.11650000	18.46440000	0.00000000
85	1514.30260000	7.14250000	0.00000000
86	1516.13360000	18.83410000	0.00000000
87	1538.24990000	3.21910000	0.00000000
88	1541.00880000	5.71830000	0.00000000
89	1630.58180000	2.46850000	0.00000000
90	1647.63600000	3.14020000	0.00000000
91	1683.49040000	84.07810000	0.00000000
92	1750.03240000	7.90870000	0.00000000
93	3066.07490000	5.77050000	0.00000000
94	3074.56820000	9.47750000	0.00000000
95	3076.69020000	0.49050000	0.00000000
96	3080.56960000	3.74200000	0.00000000
97	3085.41180000	3.28680000	0.00000000
98	3087.82230000	6.12380000	0.00000000
99	3098.41610000	6.33380000	0.00000000
100	3110.36810000	2.41940000	0.00000000
101	3115.89210000	1.13690000	0.00000000
102	3119.17000000	1.76120000	0.00000000
103	3128.27690000	0.44290000	0.00000000
104	3139.94400000	3.35390000	0.00000000
105	3152.22190000	1.24130000	0.00000000
106	3155.03340000	2.97570000	0.00000000
107	3166.46540000	6.09060000	0.00000000
108	3169.60300000	6.10820000	0.00000000
109	3188.50030000	0.84910000	0.00000000
110	3198.02670000	3.99130000	0.00000000
111	3207.09280000	1.47320000	0.00000000

S44. CALCULATIONS ON TS 9 (ISOMER 1; TRIPLET) $\longrightarrow$ 15t

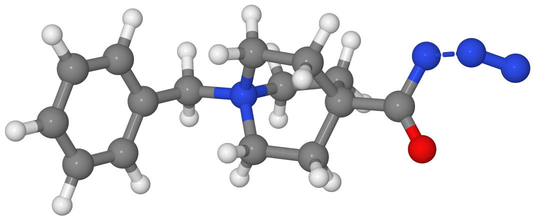

Route	:	# opt=(calcfc,ts,noeigentest) freq ub3lyp/cc-pvtz empiricaldispersion=gd3bj pop=regular geom=connectivity int=ultrafine
SMILES	:	c1ccc(cc1)C[N]23CCC(CC2)(CC3)C(=O)N=[N+]=[N-]
Formula	:	$C_{15}H_{19}N_4O^{+,3}$
Charge	:	1
Multiplicity	:	3
Energy	:	-877.27877544 a.u.
Gibbs Energy	:	-877.00064800 a.u.

Cartesian Co-ordinates (XYZ format)

39

C	-1.77184200	1.38366497	-0.87833899
C	-1.80918705	0.22826099	0.14285100
C	-0.31684500	1.84813201	-1.04324698
H	-2.18506789	1.05309999	-1.82826996
H	-2.37923694	2.21749210	-0.53188801
H	-0.05140000	1.99765396	-2.08620310
H	-0.11402200	2.76926804	-0.50263000
C	-1.10264802	-0.98408198	-0.49047300
H	-0.96897298	-1.76424205	0.25763100
H	-1.70352697	-1.40300405	-1.29503298
C	0.25639001	-0.53689098	-1.05000699
H	1.05181301	-1.22594297	-0.78931099
H	0.23955700	-0.41987199	-2.13111496
C	0.46706200	0.77326798	1.01779401
H	1.05202997	-0.07052200	1.36869800
H	0.91449100	1.68185794	1.41002297
C	-1.02012205	0.65716702	1.38357604
H	-1.14671004	-0.06740400	2.18424201
H	-1.40498102	1.60414195	1.75570905
N	0.63365799	0.81245399	-0.48565301
C	2.06264806	1.19985604	-0.88310897
C	-3.24524093	-0.10767900	0.51640600
O	-3.71435499	0.06480900	1.61737394
N	-4.01089621	-0.54183602	-0.58401299
N	-5.05988979	-1.50743794	-0.17573901
N	-6.10048008	-1.13642395	0.17031400
H	2.18194199	2.22603512	-0.54244500
H	2.06505895	1.19544899	-1.97081006
C	3.13724899	0.31407100	-0.33438000
C	3.58676505	-0.78597099	-1.06680202
C	3.73293304	0.60021400	0.89485401
C	4.59017515	-1.60323000	-0.56521302
H	3.16858912	-0.99361098	-2.04334497
C	4.73570299	-0.21645799	1.39741802

H	3.42812204	1.47450697	1.45490003
C	5.16023779	-1.32295895	0.67084002
H	4.93550110	-2.44822311	-1.14341295
H	5.19437313	0.01727600	2.34746790
H	5.94581985	-1.95520604	1.05934203

Frequencies

Mode	IR frequency	IR intensity	Raman intensity
1	-361.34820000	18.45320000	0.00000000
2	28.26920000	0.18640000	0.00000000
3	35.79630000	0.92640000	0.00000000
4	44.11070000	0.58850000	0.00000000
5	61.11650000	0.52810000	0.00000000
6	62.78320000	0.44810000	0.00000000
7	89.48750000	0.91890000	0.00000000
8	120.36540000	0.07720000	0.00000000
9	127.59250000	2.81990000	0.00000000
10	158.95300000	2.40250000	0.00000000
11	213.96750000	1.06730000	0.00000000
12	231.19310000	1.02360000	0.00000000
13	235.95750000	0.41430000	0.00000000
14	287.08030000	0.97390000	0.00000000
15	303.63360000	2.32730000	0.00000000
16	332.58080000	2.45670000	0.00000000
17	343.71810000	12.45510000	0.00000000
18	367.06680000	0.72210000	0.00000000
19	402.63110000	0.64310000	0.00000000
20	418.17730000	0.02260000	0.00000000
21	421.98800000	0.08710000	0.00000000
22	465.47290000	4.55630000	0.00000000
23	478.86860000	1.75060000	0.00000000
24	538.98800000	0.36500000	0.00000000
25	544.31240000	0.06270000	0.00000000
26	567.43390000	0.50260000	0.00000000
27	593.31050000	6.67640000	0.00000000
28	630.34600000	5.33800000	0.00000000
29	639.02700000	0.10100000	0.00000000
30	659.50850000	12.83390000	0.00000000
31	703.51220000	4.06210000	0.00000000
32	724.37490000	44.80300000	0.00000000
33	777.03180000	2.54780000	0.00000000
34	786.34750000	36.90800000	0.00000000
35	803.26700000	1.20890000	0.00000000
36	835.26820000	7.17650000	0.00000000
37	838.96070000	4.23590000	0.00000000
38	850.49920000	34.39500000	0.00000000
39	854.42980000	3.78830000	0.00000000
40	869.38000000	0.11240000	0.00000000
41	905.53010000	2.35780000	0.00000000
42	937.84110000	99.51800000	0.00000000
43	955.87940000	1.38280000	0.00000000
44	988.09520000	5.32940000	0.00000000
45	992.87940000	0.88130000	0.00000000
46	998.09160000	7.38010000	0.00000000
47	1006.45790000	0.05390000	0.00000000
48	1012.44680000	4.48290000	0.00000000
49	1018.07740000	2.99910000	0.00000000
50	1028.30060000	3.83650000	0.00000000
51	1036.94780000	0.01700000	0.00000000
52	1049.16710000	25.54570000	0.00000000
53	1053.04380000	12.72390000	0.00000000
54	1056.45870000	2.15670000	0.00000000
55	1063.03070000	9.10230000	0.00000000
56	1071.63100000	6.30720000	0.00000000
57	1122.17870000	5.22740000	0.00000000
58	1172.46600000	105.55270000	0.00000000
59	1189.37170000	3.33460000	0.00000000
60	1196.53200000	0.46010000	0.00000000

61	1205.61200000	0.88610000	0.00000000
62	1212.17580000	51.02150000	0.00000000
63	1214.64940000	0.22280000	0.00000000
64	1240.38130000	16.47680000	0.00000000
65	1258.28350000	5.26920000	0.00000000
66	1283.95740000	0.28840000	0.00000000
67	1299.98600000	7.66190000	0.00000000
68	1304.15160000	4.76420000	0.00000000
69	1329.71510000	5.13290000	0.00000000
70	1336.45410000	2.63810000	0.00000000
71	1347.39440000	0.87580000	0.00000000
72	1351.24080000	6.04990000	0.00000000
73	1363.16360000	6.60440000	0.00000000
74	1371.03610000	9.06550000	0.00000000
75	1377.19140000	0.44190000	0.00000000
76	1396.33860000	1.66680000	0.00000000
77	1404.60940000	11.43780000	0.00000000
78	1406.35850000	10.12690000	0.00000000
79	1438.50420000	10.61050000	0.00000000
80	1493.26800000	4.43300000	0.00000000
81	1496.91910000	6.70810000	0.00000000
82	1502.36700000	6.59760000	0.00000000
83	1504.05260000	0.99300000	0.00000000
84	1512.73180000	19.71750000	0.00000000
85	1513.89660000	6.19270000	0.00000000
86	1515.95150000	18.77310000	0.00000000
87	1538.17390000	3.12690000	0.00000000
88	1540.64680000	5.90370000	0.00000000
89	1630.54220000	2.47640000	0.00000000
90	1647.62300000	3.13500000	0.00000000
91	1670.83490000	74.33610000	0.00000000
92	1812.02590000	9.87860000	0.00000000
93	3065.23960000	5.63780000	0.00000000
94	3074.62070000	9.46900000	0.00000000
95	3076.67170000	0.56920000	0.00000000
96	3081.09660000	3.49060000	0.00000000
97	3085.39090000	3.07530000	0.00000000
98	3087.76830000	6.19180000	0.00000000
99	3098.29630000	6.38680000	0.00000000
100	3108.45100000	2.58370000	0.00000000
101	3115.39060000	1.18850000	0.00000000
102	3119.30360000	1.76510000	0.00000000
103	3128.26910000	0.44940000	0.00000000
104	3139.88370000	3.32850000	0.00000000
105	3152.14680000	1.21850000	0.00000000
106	3154.97860000	2.97980000	0.00000000
107	3166.43620000	6.10030000	0.00000000
108	3169.63060000	6.10740000	0.00000000
109	3188.53410000	0.84600000	0.00000000
110	3198.02820000	3.99550000	0.00000000
111	3207.12570000	1.47500000	0.00000000

S45. CALCULATIONS ON 15t

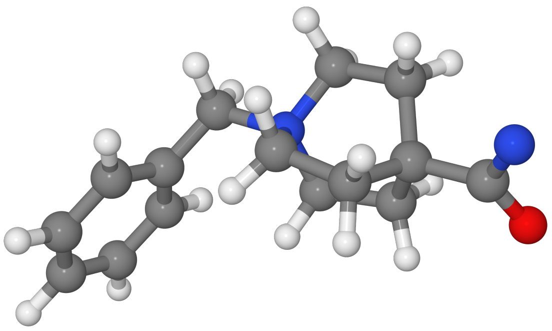

Route	:	# opt freq b3lyp/cc-pvtz empiricaldispersion=gd3bj pop=regular geom=connectivity int=ultrafine
SMILES	:	c1ccc(cc1)C[N]23CCC(CC2)(CC3)C(=O)[N]
Formula	:	$C_{15}H_{19}N_2O^{+,3}$
Charge	:	1
Multiplicity	:	3
Energy	:	-767.76239799 a.u.
Gibbs Energy	:	-767.48769400 a.u.

Cartesian Co-ordinates (XYZ format)

37

C	2.47695398	1.35089803	-0.20326801
C	2.40217495	-0.17340900	0.01529300
C	1.07309496	1.86842299	-0.55295902
H	2.85625696	1.83995199	0.69205898
H	3.16040397	1.58821297	-1.01634002
H	0.82194197	2.77285790	-0.00575900
H	0.96197098	2.06921196	-1.61558104
C	1.57868195	-0.42664599	1.29271698
H	1.37596500	-1.49190998	1.39238405
H	2.13220501	-0.11748300	2.17699599
C	0.26540500	0.36671600	1.21032202
H	-0.59181303	-0.23151200	1.49801695
H	0.28489801	1.26289499	1.82618904
C	0.19317500	-0.34642500	-1.13967204
H	-0.47451499	-1.12051201	-0.77606797
H	-0.15736800	-0.02855100	-2.11724401
C	1.66138804	-0.79730201	-1.17073095
H	1.71600699	-1.88188398	-1.12392402
H	2.13878989	-0.50018102	-2.10254407
N	0.02704300	0.83308297	-0.20617200
C	-1.35827804	1.47434497	-0.35297799
H	-1.37396705	1.88427997	-1.36046898
H	-1.36449099	2.29982400	0.35528901
C	3.79980993	-0.76497698	0.15182300
O	4.23672724	-1.64792204	-0.57971799
N	4.60417700	-0.27471000	1.14776599
C	-2.51342201	0.55124301	-0.12097100
C	-3.07384109	-0.16321100	-1.18081403
C	-3.07169294	0.42661899	1.15237904
C	-4.15064621	-1.01130903	-0.96555400

H	-2.68225098	-0.04404800	-2.18252110
C	-4.14909697	-0.42115399	1.36863005
H	-2.67910290	1.01029205	1.97504902
C	-4.68452787	-1.14630795	0.31086999
H	-4.58092690	-1.55557406	-1.79394102
H	-4.57799196	-0.50523800	2.35683703
H	-5.52726889	-1.80199003	0.47722000

Frequencies

Mode	IR frequency	IR intensity	Raman intensity
1	28.65070000	0.28510000	0.00000000
2	39.46000000	0.26740000	0.00000000
3	60.35330000	0.80370000	0.00000000
4	79.01580000	1.84030000	0.00000000
5	113.14600000	0.06010000	0.00000000
6	139.53830000	5.18820000	0.00000000
7	149.44310000	1.60020000	0.00000000
8	216.74870000	0.85920000	0.00000000
9	232.40620000	0.41480000	0.00000000
10	244.74780000	0.36160000	0.00000000
11	309.65460000	0.76410000	0.00000000
12	321.31650000	0.85340000	0.00000000
13	337.76890000	12.01080000	0.00000000
14	367.31000000	0.90430000	0.00000000
15	400.66820000	0.71110000	0.00000000
16	418.01110000	0.03970000	0.00000000
17	421.76310000	0.04140000	0.00000000
18	459.05140000	6.36550000	0.00000000
19	476.66270000	1.55160000	0.00000000
20	537.88460000	0.42910000	0.00000000
21	543.29930000	0.42860000	0.00000000
22	563.55180000	2.56610000	0.00000000
23	603.98820000	22.22290000	0.00000000
24	638.92550000	0.13720000	0.00000000
25	649.29410000	30.64040000	0.00000000
26	662.79270000	9.97540000	0.00000000
27	708.06880000	1.26350000	0.00000000
28	724.27960000	44.44120000	0.00000000
29	786.36270000	35.16390000	0.00000000
30	803.22700000	0.75100000	0.00000000
31	834.03530000	7.07650000	0.00000000
32	838.54440000	5.10060000	0.00000000
33	848.09090000	40.53270000	0.00000000
34	854.02690000	3.73560000	0.00000000
35	869.23570000	0.12950000	0.00000000
36	906.09470000	1.39760000	0.00000000
37	951.08350000	21.37840000	0.00000000
38	957.40640000	5.55930000	0.00000000
39	985.87740000	1.35630000	0.00000000
40	991.52490000	0.72030000	0.00000000
41	993.89630000	1.31430000	0.00000000
42	1006.61090000	0.05050000	0.00000000
43	1010.96820000	3.71570000	0.00000000
44	1017.57200000	1.62660000	0.00000000
45	1028.31040000	3.95030000	0.00000000
46	1037.17900000	0.01980000	0.00000000
47	1051.50590000	3.26520000	0.00000000
48	1056.58140000	2.11900000	0.00000000
49	1060.79980000	4.19330000	0.00000000
50	1066.17210000	17.60540000	0.00000000
51	1080.00920000	2.41260000	0.00000000
52	1122.29500000	5.17210000	0.00000000
53	1155.54950000	49.03710000	0.00000000
54	1188.12400000	1.35530000	0.00000000
55	1196.62680000	0.23820000	0.00000000
56	1205.52350000	0.02040000	0.00000000
57	1213.35490000	13.63100000	0.00000000
58	1214.93870000	0.12380000	0.00000000
59	1240.81330000	17.72360000	0.00000000
60	1259.86500000	2.18130000	0.00000000

61	1282.85030000	0.36240000	0.00000000
62	1300.19030000	5.35090000	0.00000000
63	1306.65400000	6.23210000	0.00000000
64	1330.15740000	4.82670000	0.00000000
65	1338.17100000	1.99010000	0.00000000
66	1347.74030000	1.07100000	0.00000000
67	1350.56650000	4.69170000	0.00000000
68	1363.50660000	5.91400000	0.00000000
69	1371.48180000	10.36490000	0.00000000
70	1377.31910000	0.53200000	0.00000000
71	1395.94270000	1.75460000	0.00000000
72	1404.85560000	10.93430000	0.00000000
73	1406.42020000	10.98250000	0.00000000
74	1438.84130000	10.32590000	0.00000000
75	1493.12510000	4.23810000	0.00000000
76	1496.95680000	6.37290000	0.00000000
77	1501.89880000	6.87220000	0.00000000
78	1503.59360000	1.52270000	0.00000000
79	1512.10250000	16.87020000	0.00000000
80	1513.82740000	9.77130000	0.00000000
81	1515.20190000	17.55750000	0.00000000
82	1538.17150000	3.17950000	0.00000000
83	1538.77980000	78.71960000	0.00000000
84	1541.09140000	27.19190000	0.00000000
85	1630.58940000	2.48130000	0.00000000
86	1647.44870000	3.28980000	0.00000000
87	3067.12750000	4.95890000	0.00000000
88	3069.42840000	5.73490000	0.00000000
89	3075.73240000	5.57680000	0.00000000
90	3077.91950000	3.27670000	0.00000000
91	3085.21730000	2.76200000	0.00000000
92	3087.67540000	5.59200000	0.00000000
93	3098.49340000	5.56170000	0.00000000
94	3108.11470000	1.86510000	0.00000000
95	3109.92820000	2.51190000	0.00000000
96	3119.94160000	1.48200000	0.00000000
97	3128.35290000	0.42740000	0.00000000
98	3139.06650000	3.13180000	0.00000000
99	3152.46400000	1.14640000	0.00000000
100	3155.16230000	2.73880000	0.00000000
101	3166.86150000	5.89160000	0.00000000
102	3169.41890000	6.13220000	0.00000000
103	3188.64570000	0.83000000	0.00000000
104	3198.19370000	3.91030000	0.00000000
105	3207.23360000	1.37410000	0.00000000

S46. CALCULATIONS ON TS 15t ⟶ 16t

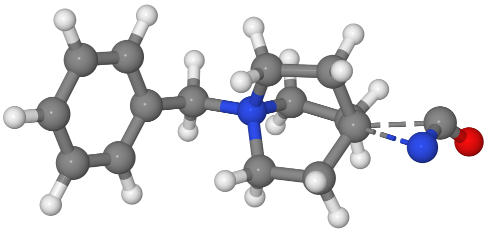

Route	:	# opt=(calcfc,ts,noeigentest) freq ub3lyp/cc-pvtz empiricaldispersion=gd3bj pop=regular geom=connectivity int=ultrafine
SMILES	:	c1ccc(cc1)C[N]23CC[C](CC2)CC3.[C](=O)[N]
Formula	:	$C_{15}H_{19}N_2O^{+,3}$
Charge	:	1
Multiplicity	:	3
Energy	:	-767.67775856 a.u.
Gibbs Energy	:	-767.40525100 a.u.

Cartesian Co-ordinates (XYZ format)

37

C	0.32119101	-0.85688102	-1.20785701
C	0.86451298	-0.13418800	0.01994700
C	-1.19051504	-0.56679100	-1.30872905
H	0.49913299	-1.92452800	-1.11927903
H	0.81867301	-0.51552403	-2.11191297
H	-1.42650497	0.17331900	-2.06967306
H	-1.76089001	-1.46677005	-1.50969899
C	0.33231401	-0.76708698	1.28581202
H	0.58631903	-0.15822200	2.15014601
H	0.75548100	-1.75692499	1.43864703
C	-1.20192897	-0.88556898	1.13089895
H	-1.52012205	-1.89404798	0.88968301
H	-1.72034204	-0.56804800	2.03062701
C	-1.08662403	1.37638497	0.16853200
H	-1.58275795	2.03201294	-0.54181200
H	-1.33893704	1.70201004	1.17448103
C	0.44306901	1.33442295	-0.05211500
H	0.71004498	1.75807500	-1.01720595
H	0.92588401	1.93427098	0.71206897
N	-1.68717206	-0.00085400	0.00058400
C	-3.21545696	0.12835599	0.01030700
H	-3.44840598	0.66239399	0.92901099
H	-3.44907188	0.77416998	-0.83316803
C	2.80443692	0.26233500	0.33322099
O	3.39899993	1.19671297	-0.13983400
N	2.66964102	-0.97888398	-0.12323600
C	-3.96043611	-1.16693294	-0.07350100
C	-4.33374786	-1.68661201	-1.31420302
C	-4.32742023	-1.84987795	1.08705294
C	-5.03170300	-2.88350892	-1.39452398
H	-4.09922600	-1.14443803	-2.22115302
C	-5.02499390	-3.04660296	1.00767100
H	-4.08642483	-1.43780398	2.05824494
C	-5.37127781	-3.56795597	-0.23362400

H	-5.32157516	-3.27382708	-2.35949302
H	-5.30970383	-3.56456804	1.91223395
H	-5.92016983	-4.49678516	-0.29514799

Frequencies

Mode	IR frequency	IR intensity	Raman intensity
1	-858.42910000	142.79440000	0.00000000
2	32.02360000	0.80500000	0.00000000
3	47.39590000	0.75580000	0.00000000
4	60.91190000	0.78100000	0.00000000
5	87.41120000	0.93780000	0.00000000
6	118.77120000	0.14120000	0.00000000
7	131.01270000	3.11240000	0.00000000
8	181.26100000	2.05880000	0.00000000
9	192.68450000	0.44110000	0.00000000
10	238.87070000	0.22880000	0.00000000
11	241.80800000	2.30600000	0.00000000
12	272.65460000	1.70320000	0.00000000
13	310.48180000	0.92690000	0.00000000
14	332.02320000	0.12990000	0.00000000
15	386.38990000	0.36600000	0.00000000
16	390.39710000	0.15270000	0.00000000
17	411.47890000	0.25500000	0.00000000
18	418.79810000	0.04620000	0.00000000
19	423.94360000	0.01710000	0.00000000
20	464.56280000	1.91480000	0.00000000
21	521.34350000	8.82130000	0.00000000
22	534.71340000	1.22410000	0.00000000
23	549.18930000	26.98960000	0.00000000
24	572.20550000	5.42460000	0.00000000
25	630.35870000	1.60600000	0.00000000
26	638.92590000	0.12200000	0.00000000
27	688.24590000	14.35500000	0.00000000
28	724.67630000	44.84300000	0.00000000
29	775.74770000	3.27400000	0.00000000
30	786.22090000	37.40650000	0.00000000
31	807.77300000	0.66960000	0.00000000
32	833.42350000	3.96020000	0.00000000
33	839.72720000	3.48160000	0.00000000
34	856.32700000	2.67930000	0.00000000
35	869.78520000	0.15840000	0.00000000
36	882.33250000	89.07990000	0.00000000
37	902.85670000	0.77230000	0.00000000
38	953.90020000	4.54800000	0.00000000
39	969.98450000	7.55480000	0.00000000
40	987.42090000	5.72670000	0.00000000
41	989.55240000	1.77620000	0.00000000
42	998.97900000	4.90640000	0.00000000
43	1006.95150000	0.03700000	0.00000000
44	1013.86690000	9.01260000	0.00000000
45	1016.54950000	2.25220000	0.00000000
46	1028.21220000	4.00370000	0.00000000
47	1033.50940000	0.88130000	0.00000000
48	1037.40800000	0.05090000	0.00000000
49	1056.36510000	1.64240000	0.00000000
50	1060.59450000	1.60080000	0.00000000
51	1069.94120000	20.64960000	0.00000000
52	1122.41820000	5.45020000	0.00000000
53	1159.14530000	23.10190000	0.00000000
54	1193.69690000	1.54620000	0.00000000
55	1197.05950000	0.02170000	0.00000000
56	1201.90120000	0.42410000	0.00000000
57	1204.18470000	1.18920000	0.00000000
58	1214.96100000	1.08950000	0.00000000
59	1240.48570000	19.81960000	0.00000000
60	1251.84700000	1.97660000	0.00000000

61	1269.27730000	6.73210000	0.00000000
62	1286.00020000	5.79600000	0.00000000
63	1298.57270000	10.74220000	0.00000000
64	1326.19340000	2.67440000	0.00000000
65	1328.25700000	8.41020000	0.00000000
66	1344.13940000	3.55400000	0.00000000
67	1347.29510000	3.50290000	0.00000000
68	1360.34600000	8.01110000	0.00000000
69	1361.17390000	9.04870000	0.00000000
70	1368.90970000	15.37070000	0.00000000
71	1376.81750000	0.52070000	0.00000000
72	1399.96380000	12.12060000	0.00000000
73	1402.23200000	11.55600000	0.00000000
74	1432.46110000	8.87650000	0.00000000
75	1491.76370000	3.27720000	0.00000000
76	1496.86770000	6.79730000	0.00000000
77	1500.35680000	1.11940000	0.00000000
78	1502.45790000	6.52900000	0.00000000
79	1508.94610000	21.46880000	0.00000000
80	1511.63590000	12.84780000	0.00000000
81	1514.99370000	18.84270000	0.00000000
82	1538.14920000	3.20610000	0.00000000
83	1539.37240000	3.19110000	0.00000000
84	1570.06940000	156.36730000	0.00000000
85	1630.43600000	2.49440000	0.00000000
86	1647.32420000	3.53930000	0.00000000
87	3075.11910000	6.51430000	0.00000000
88	3079.98090000	1.50780000	0.00000000
89	3084.65370000	3.35640000	0.00000000
90	3087.54870000	3.79510000	0.00000000
91	3090.13940000	1.14450000	0.00000000
92	3092.26620000	3.30370000	0.00000000
93	3101.07590000	5.62160000	0.00000000
94	3122.17220000	0.98120000	0.00000000
95	3127.29670000	0.41560000	0.00000000
96	3132.05190000	1.85480000	0.00000000
97	3132.83890000	0.88190000	0.00000000
98	3148.51860000	1.65000000	0.00000000
99	3156.02670000	1.74660000	0.00000000
100	3159.55680000	1.57280000	0.00000000
101	3167.27990000	5.87980000	0.00000000
102	3168.98630000	6.64520000	0.00000000
103	3188.78980000	0.82370000	0.00000000
104	3198.33920000	3.77950000	0.00000000
105	3207.37950000	1.35600000	0.00000000

S47. CALCULATIONS ON 16t

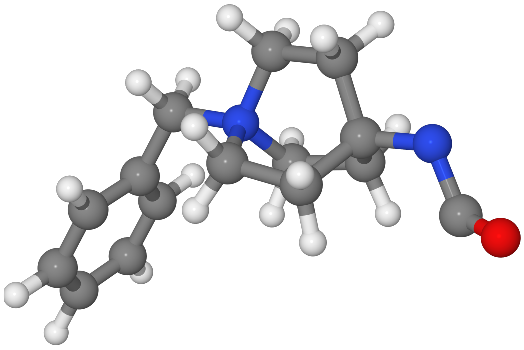

Route	:	# opt freq ub3lyp/cc-pvtz empiricaldispersion=gd3bj pop=regular geom=connectivity int=ultrafine
SMILES	:	c1ccc(cc1)C[N]23CCC(CC2)(CC3)[N][C]=O
Formula	:	$C_{15}H_{19}N_2O^{+,3}$
Charge	:	1
Multiplicity	:	3
Energy	:	-767.75729950 a.u.
Gibbs Energy	:	-767.48344300 a.u.

Cartesian Co-ordinates (XYZ format)

37

C	0.52520901	-0.76174402	0.95861697
C	0.78691000	0.44570100	0.03404800
C	-0.98980403	-0.97803199	1.07931006
H	1.01557600	-1.64394903	0.55132002
H	0.94887102	-0.57415402	1.94293797
H	-1.25808001	-2.02521110	0.99422002
H	-1.38926601	-0.59193403	2.01404810
C	0.36210400	0.03732600	-1.39839399
H	0.38598400	0.91395301	-2.04226494
H	1.06344604	-0.68975198	-1.79948604
C	-1.04787505	-0.57005101	-1.34188998
H	-1.68734205	-0.17844801	-2.12760806
H	-1.03653204	-1.65212595	-1.41990805
C	-1.57597005	1.24648798	0.22126600
H	-1.96931803	1.73753905	-0.66549301
H	-2.22039390	1.49184299	1.06070495
C	-0.10801100	1.60586202	0.48726100
H	0.16053300	2.50827909	-0.05654700
H	0.06024900	1.80317104	1.54436004
N	-1.71221995	-0.24134700	-0.02458300
C	-3.20402002	-0.59750199	-0.04477500
H	-3.57228994	-0.32353801	0.94133300
H	-3.64460611	0.07592200	-0.77672499
N	2.15794802	0.87350601	0.04842900
C	3.10438204	-0.10053000	-0.26300299
O	4.18054819	-0.14890900	0.23781499
C	-3.51149297	-2.02732205	-0.36213899
C	-3.71460795	-2.43282890	-1.68207896
C	-3.64008689	-2.96523499	0.66376603
C	-4.00767708	-3.75734091	-1.97348702

H	-3.66419792	-1.70976806	-2.48565412
C	-3.93363810	-4.29029083	0.37327799
H	-3.53369999	-2.65674591	1.69581997
C	-4.11032200	-4.68854094	-0.94639999
H	-4.16901112	-4.05990410	-2.99819493
H	-4.03762388	-5.00687981	1.17530799
H	-4.34487295	-5.71880722	-1.17296898

Frequencies

Mode	IR frequency	IR intensity	Raman intensity
1	35.34530000	0.08860000	0.00000000
2	52.90390000	1.04470000	0.00000000
3	57.04900000	1.23780000	0.00000000
4	84.41900000	1.17060000	0.00000000
5	89.74810000	0.86990000	0.00000000
6	124.60570000	0.12280000	0.00000000
7	142.31620000	2.65400000	0.00000000
8	199.88100000	3.22960000	0.00000000
9	214.85190000	1.84160000	0.00000000
10	244.09010000	0.10360000	0.00000000
11	259.44770000	0.48110000	0.00000000
12	298.92260000	0.86680000	0.00000000
13	337.62870000	0.57620000	0.00000000
14	381.80010000	1.56150000	0.00000000
15	393.83580000	0.03900000	0.00000000
16	410.51750000	2.14250000	0.00000000
17	418.31410000	0.05710000	0.00000000
18	422.72600000	0.02760000	0.00000000
19	465.66930000	4.47500000	0.00000000
20	491.94020000	0.81050000	0.00000000
21	535.02600000	0.11090000	0.00000000
22	537.14160000	0.10820000	0.00000000
23	581.63630000	11.21860000	0.00000000
24	631.46330000	3.61710000	0.00000000
25	638.93500000	0.10920000	0.00000000
26	691.74250000	8.87130000	0.00000000
27	724.96380000	43.68650000	0.00000000
28	748.01770000	7.83640000	0.00000000
29	786.44270000	34.98360000	0.00000000
30	802.72850000	1.83310000	0.00000000
31	831.85660000	6.09090000	0.00000000
32	837.43930000	4.02990000	0.00000000
33	846.84530000	40.87620000	0.00000000
34	854.27620000	2.15170000	0.00000000
35	869.91030000	0.08790000	0.00000000
36	893.55280000	18.24920000	0.00000000
37	903.50380000	1.65500000	0.00000000
38	955.41710000	2.53320000	0.00000000
39	965.82910000	2.97230000	0.00000000
40	983.59120000	6.75830000	0.00000000
41	993.13130000	5.11420000	0.00000000
42	1005.66210000	8.24610000	0.00000000
43	1007.10120000	0.60640000	0.00000000
44	1015.04460000	1.59480000	0.00000000
45	1021.66290000	12.72900000	0.00000000
46	1028.22900000	3.95190000	0.00000000
47	1037.46840000	0.00440000	0.00000000
48	1044.91910000	3.48040000	0.00000000
49	1055.26020000	2.72820000	0.00000000
50	1058.10150000	0.72570000	0.00000000
51	1065.31730000	27.93430000	0.00000000
52	1121.97340000	5.68260000	0.00000000
53	1149.13850000	5.07510000	0.00000000
54	1172.11810000	1.31100000	0.00000000
55	1196.81400000	0.11780000	0.00000000
56	1199.12500000	1.46020000	0.00000000
57	1206.01150000	8.14130000	0.00000000
58	1214.91910000	0.60000000	0.00000000
59	1240.70910000	18.81180000	0.00000000
60	1253.00930000	1.20850000	0.00000000

61	1278.77530000	2.85600000	0.00000000
62	1284.31130000	4.58900000	0.00000000
63	1291.31490000	13.31810000	0.00000000
64	1323.59920000	0.32800000	0.00000000
65	1330.33970000	2.55520000	0.00000000
66	1343.12240000	9.49360000	0.00000000
67	1347.19780000	1.02100000	0.00000000
68	1360.69910000	4.50150000	0.00000000
69	1364.44720000	7.65900000	0.00000000
70	1375.63590000	1.60260000	0.00000000
71	1381.70300000	9.22080000	0.00000000
72	1401.19950000	12.88940000	0.00000000
73	1405.54630000	9.86740000	0.00000000
74	1438.22650000	11.40750000	0.00000000
75	1492.90480000	5.02260000	0.00000000
76	1497.03890000	7.50780000	0.00000000
77	1502.71600000	1.29230000	0.00000000
78	1504.36420000	3.29940000	0.00000000
79	1511.94630000	21.18160000	0.00000000
80	1513.97170000	9.90750000	0.00000000
81	1515.43180000	14.85400000	0.00000000
82	1538.19910000	3.06460000	0.00000000
83	1540.51310000	5.74360000	0.00000000
84	1630.49840000	2.51610000	0.00000000
85	1647.34420000	3.19540000	0.00000000
86	1752.90570000	43.02940000	0.00000000
87	3071.59910000	2.54610000	0.00000000
88	3074.90790000	8.80250000	0.00000000
89	3076.29990000	2.84930000	0.00000000
90	3077.57270000	1.94060000	0.00000000
91	3085.77000000	4.61590000	0.00000000
92	3088.89740000	3.86520000	0.00000000
93	3098.31940000	4.75700000	0.00000000
94	3115.75610000	1.96130000	0.00000000
95	3118.12490000	1.45860000	0.00000000
96	3122.15190000	1.11160000	0.00000000
97	3128.70310000	0.32690000	0.00000000
98	3139.97120000	3.66200000	0.00000000
99	3152.93260000	1.87790000	0.00000000
100	3155.69050000	2.58860000	0.00000000
101	3166.67950000	5.92750000	0.00000000
102	3169.03200000	6.29880000	0.00000000
103	3188.73960000	0.79740000	0.00000000
104	3198.25570000	3.78440000	0.00000000
105	3207.32200000	1.32400000	0.00000000

S48. CALCULATIONS ON 10 (ISOMER 1)

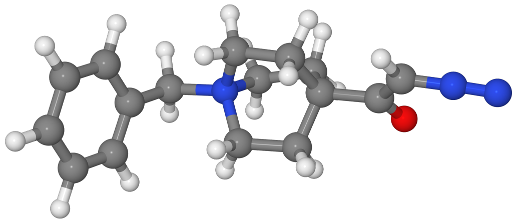

Route	:	# opt freq b3lyp/cc-pvtz empiricaldispersion=gd3bj pop=regular geom=connectivity int=ultrafine
SMILES	:	c1ccc(cc1)C[N]23CCC(CC2)(CC3)C(=O)[CH][N][N]
Formula	:	$C_{16}H_{20}N_3O^+$
Charge	:	1
Multiplicity	:	1
Energy	:	-861.33112346 a.u.
Gibbs Energy	:	-861.03439500 a.u.

Cartesian Co-ordinates (XYZ format)

40

C	-0.39415699	-1.21679997	-0.97023898
C	-0.92635101	-0.07520500	-0.09704800
C	1.13383305	-1.08663905	-1.09558201
H	-0.64768797	-2.18159008	-0.53248501
H	-0.83130199	-1.18088102	-1.96657395
H	1.63915002	-2.03621292	-0.95954198
H	1.43364298	-0.67008603	-2.05442190
C	-0.40339699	-0.29703900	1.33576596
H	-0.64420700	0.57521600	1.93874097
H	-0.89942002	-1.15245497	1.79127896
C	1.11103904	-0.54973000	1.30020106
H	1.64059806	0.02975000	2.05128908
H	1.36578500	-1.59563005	1.43705297
C	1.18218303	1.25459099	-0.35568699
H	1.44695699	1.86455798	0.50479501
H	1.75045300	1.60690796	-1.21229696
C	-0.32701001	1.24053597	-0.62055099
H	-0.80476803	2.07701802	-0.11852400
H	-0.52923101	1.33808804	-1.68673003
N	1.67073202	-0.14473499	-0.04376700
C	3.20111799	-0.13599201	-0.03608300
C	-2.45447612	0.07322500	-0.01870700
O	-2.91883707	1.06493199	0.51922399
C	-3.26216698	-0.99104899	-0.55318397
H	-2.93063498	-1.88849795	-1.04179001
N	-4.56989479	-0.85177702	-0.43291101
N	-5.66322708	-0.67337298	-0.30050400
H	3.47591710	0.65933198	0.65328300
H	3.48843598	0.16609500	-1.04074705
C	3.84553099	-1.43305898	0.34441200
C	4.18915510	-2.36457705	-0.63658398
C	4.15031815	-1.70918298	1.67792404
C	4.79663086	-3.56295109	-0.28823599
H	4.00239182	-2.14494705	-1.67986298

C	4.75706196	-2.90708303	2.02757001
H	3.93094206	-0.97942001	2.44616389
C	5.07448483	-3.83806705	1.04531205
H	5.06479883	-4.27383280	-1.05661201
H	4.99395895	-3.10818505	3.06251502
H	5.55322504	-4.76807308	1.31689799

Frequencies

Mode	IR frequency	IR intensity	Raman intensity
1	26.67110000	3.80750000	0.00000000
2	37.15410000	0.16580000	0.00000000
3	55.64370000	2.26200000	0.00000000
4	66.51560000	3.59470000	0.00000000
5	91.45810000	1.02510000	0.00000000
6	103.59800000	0.45690000	0.00000000
7	128.86850000	0.25350000	0.00000000
8	168.31860000	4.51360000	0.00000000
9	178.87700000	1.67730000	0.00000000
10	222.85510000	0.77400000	0.00000000
11	228.18070000	0.87180000	0.00000000
12	243.73750000	0.32880000	0.00000000
13	291.68510000	0.75410000	0.00000000
14	328.15310000	2.53970000	0.00000000
15	352.69680000	5.49800000	0.00000000
16	364.96970000	3.72790000	0.00000000
17	398.90430000	0.20980000	0.00000000
18	418.77950000	0.05910000	0.00000000
19	422.76030000	0.05560000	0.00000000
20	443.23070000	1.28810000	0.00000000
21	467.36040000	1.52300000	0.00000000
22	481.99130000	4.91430000	0.00000000
23	508.49170000	39.28870000	0.00000000
24	540.50890000	0.86660000	0.00000000
25	545.56200000	1.34690000	0.00000000
26	571.79550000	0.05490000	0.00000000
27	587.00640000	6.85730000	0.00000000
28	638.42910000	5.21100000	0.00000000
29	639.22440000	0.12590000	0.00000000
30	701.93560000	6.80000000	0.00000000
31	724.57500000	44.96380000	0.00000000
32	735.36180000	5.45360000	0.00000000
33	786.42590000	37.95340000	0.00000000
34	806.02510000	2.50640000	0.00000000
35	825.03260000	3.91960000	0.00000000
36	834.47160000	5.75760000	0.00000000
37	843.80970000	7.73360000	0.00000000
38	858.45920000	2.60570000	0.00000000
39	869.35600000	80.82320000	0.00000000
40	869.89130000	1.06310000	0.00000000
41	905.53700000	1.41100000	0.00000000
42	954.81330000	3.70320000	0.00000000
43	960.06250000	13.33300000	0.00000000
44	987.88960000	1.59830000	0.00000000
45	997.95500000	0.23940000	0.00000000
46	1001.27260000	1.26520000	0.00000000
47	1006.17560000	0.11250000	0.00000000
48	1008.38990000	12.69420000	0.00000000
49	1018.96930000	1.79130000	0.00000000
50	1028.37910000	3.91560000	0.00000000
51	1035.97040000	0.02660000	0.00000000
52	1051.95110000	2.56560000	0.00000000
53	1055.97550000	0.76010000	0.00000000
54	1058.38930000	3.45500000	0.00000000
55	1061.39920000	5.50470000	0.00000000
56	1072.59710000	14.54200000	0.00000000
57	1122.20720000	5.31720000	0.00000000
58	1151.69400000	5.13180000	0.00000000
59	1190.68270000	15.58310000	0.00000000
60	1195.67850000	73.26420000	0.00000000

61	1196.53570000	21.86320000	0.00000000
62	1204.37630000	2.01830000	0.00000000
63	1211.58740000	51.71630000	0.00000000
64	1214.78180000	1.93240000	0.00000000
65	1240.58430000	17.45470000	0.00000000
66	1255.63770000	1.00870000	0.00000000
67	1284.11570000	3.63500000	0.00000000
68	1291.30430000	3.45380000	0.00000000
69	1305.20720000	4.50130000	0.00000000
70	1326.50110000	0.83730000	0.00000000
71	1337.00280000	1.72520000	0.00000000
72	1345.22960000	13.48600000	0.00000000
73	1349.07430000	10.08660000	0.00000000
74	1364.97660000	12.19080000	0.00000000
75	1366.52550000	6.36360000	0.00000000
76	1378.03970000	0.70390000	0.00000000
77	1386.48110000	80.06890000	0.00000000
78	1403.13420000	15.90530000	0.00000000
79	1406.55240000	48.15320000	0.00000000
80	1413.18050000	314.94080000	0.00000000
81	1438.07970000	13.24330000	0.00000000
82	1493.37260000	3.85160000	0.00000000
83	1496.97730000	7.85980000	0.00000000
84	1505.24710000	4.26980000	0.00000000
85	1506.93800000	3.56230000	0.00000000
86	1514.62450000	13.82100000	0.00000000
87	1516.49750000	8.52050000	0.00000000
88	1517.97860000	16.02600000	0.00000000
89	1538.39790000	3.46850000	0.00000000
90	1542.19560000	5.47870000	0.00000000
91	1630.75640000	2.40440000	0.00000000
92	1648.15910000	2.67610000	0.00000000
93	1699.02830000	187.03790000	0.00000000
94	2266.29090000	505.77400000	0.00000000
95	3057.75630000	12.22920000	0.00000000
96	3064.48260000	9.01860000	0.00000000
97	3071.79470000	6.28830000	0.00000000
98	3075.00790000	4.75680000	0.00000000
99	3082.58390000	6.62420000	0.00000000
100	3086.12860000	4.01440000	0.00000000
101	3094.66520000	8.34050000	0.00000000
102	3102.21160000	6.71660000	0.00000000
103	3114.38090000	1.68720000	0.00000000
104	3122.40800000	2.57570000	0.00000000
105	3128.35130000	1.24110000	0.00000000
106	3138.10460000	4.34160000	0.00000000
107	3149.66950000	1.75830000	0.00000000
108	3153.11890000	3.41010000	0.00000000
109	3166.67000000	6.06810000	0.00000000
110	3170.00700000	5.87300000	0.00000000
111	3187.88730000	0.98030000	0.00000000
112	3197.34130000	4.66310000	0.00000000
113	3206.60070000	1.83110000	0.00000000
114	3262.74900000	5.40340000	0.00000000

S49. CALCULATIONS ON 10 (ISOMER 2)

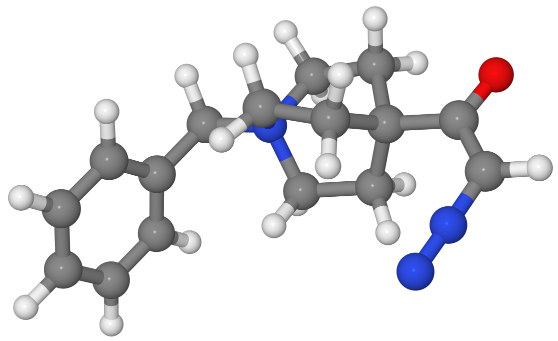

Route	:	# opt freq b3lyp/cc-pvtz empiricaldispersion=gd3bj pop=regular geom=connectivity int=ultrafine
SMILES	:	c1ccc(cc1)C[N]23CCC(CC2)(CC3)C(=O)[CH][N][N]
Formula	:	$C_{16}H_{20}N_3O^+$
Charge	:	1
Multiplicity	:	1
Energy	:	-861.32684776 a.u.
Gibbs Energy	:	-861.02897200 a.u.

Cartesian Co-ordinates (XYZ format)

40

C	1.31125200	0.17047501	-1.21129203
C	2.09357500	-0.40351799	-0.01324800
C	-0.08256500	-0.47708800	-1.26436806
H	1.20678699	1.24947703	-1.13785994
H	1.83920896	-0.03973700	-2.13939404
H	-0.86284202	0.25137001	-1.45437801
H	-0.14660300	-1.25893795	-2.01741099
C	1.40931404	0.06139900	1.28732002
H	1.81629705	-0.50208801	2.12551308
H	1.58693302	1.11182404	1.49792898
C	-0.10681500	-0.16130701	1.16960394
H	-0.52918398	-0.57661903	2.07990909
H	-0.64480197	0.75078201	0.93181098
C	0.51261300	-2.33253407	0.22057100
H	0.37436399	-2.67947412	1.24200499
H	0.15225600	-3.10550308	-0.45278299
C	1.96234000	-1.93329406	-0.07389800
H	2.63034391	-2.39507008	0.64697099
H	2.27249002	-2.28962398	-1.05364704
N	-0.40204000	-1.13755703	0.05522200
C	-1.85429502	-1.61929595	0.09846400
C	3.58838391	-0.03181400	-0.05985600
O	4.42751122	-0.90925598	-0.11148500
C	4.01591682	1.35568094	-0.05192600
H	5.06639290	1.59602404	-0.09486700
N	3.19647193	2.37011909	0.01230300
N	2.47968602	3.23571992	0.07041600
H	-1.93524301	-2.19129491	1.02023900
H	-1.95025694	-2.30461788	-0.74071002
C	-2.88677597	-0.53669101	0.03549400

C	-3.40719604	-0.12893300	-1.19381499
C	-3.37227988	0.04930200	1.20524204
C	-4.37081099	0.86814201	-1.25491297
H	-3.07586288	-0.60662198	-2.10681295
C	-4.33515215	1.04660296	1.14544702
H	-3.01304102	-0.28638899	2.16920209
C	-4.83013105	1.46181095	-0.08538300
H	-4.77170706	1.17129397	-2.21147704
H	-4.70849180	1.48949397	2.05766010
H	-5.58481598	2.23391891	-0.13156299

Frequencies

Mode	IR frequency	IR intensity	Raman intensity
1	30.06850000	0.78690000	0.00000000
2	50.50800000	0.35230000	0.00000000
3	62.82240000	1.15150000	0.00000000
4	83.47710000	0.53180000	0.00000000
5	99.50480000	0.15370000	0.00000000
6	122.27470000	3.99810000	0.00000000
7	133.14710000	0.51130000	0.00000000
8	152.22410000	0.60100000	0.00000000
9	164.13930000	7.77690000	0.00000000
10	224.81380000	0.41300000	0.00000000
11	237.52160000	0.79890000	0.00000000
12	242.50720000	0.25920000	0.00000000
13	316.51750000	0.43730000	0.00000000
14	322.13010000	0.24210000	0.00000000
15	357.71620000	9.81270000	0.00000000
16	379.02130000	0.21630000	0.00000000
17	392.72120000	1.00040000	0.00000000
18	419.00780000	0.04820000	0.00000000
19	425.63910000	0.00320000	0.00000000
20	438.53810000	5.09420000	0.00000000
21	473.53230000	5.40770000	0.00000000
22	531.24130000	1.65920000	0.00000000
23	542.84590000	0.22630000	0.00000000
24	555.68880000	7.52930000	0.00000000
25	559.71970000	29.16660000	0.00000000
26	565.99380000	0.02120000	0.00000000
27	595.71150000	6.84630000	0.00000000
28	639.14100000	0.05740000	0.00000000
29	645.74300000	11.95540000	0.00000000
30	659.07310000	26.90680000	0.00000000
31	707.92800000	1.10650000	0.00000000
32	724.53130000	44.13740000	0.00000000
33	747.34380000	17.85870000	0.00000000
34	786.56860000	34.21030000	0.00000000
35	795.97050000	0.47450000	0.00000000
36	832.89000000	5.01140000	0.00000000
37	838.64580000	8.21290000	0.00000000
38	849.59410000	33.00480000	0.00000000
39	853.13560000	2.13880000	0.00000000
40	869.39400000	0.04420000	0.00000000
41	906.51260000	1.71680000	0.00000000
42	947.28670000	22.94380000	0.00000000
43	955.87980000	0.39620000	0.00000000
44	991.46520000	0.38280000	0.00000000
45	996.84510000	0.78680000	0.00000000
46	1002.44450000	0.48810000	0.00000000
47	1006.11100000	0.06690000	0.00000000
48	1013.07830000	15.25340000	0.00000000
49	1019.22430000	3.34030000	0.00000000
50	1028.35050000	3.50350000	0.00000000
51	1036.24800000	0.01190000	0.00000000
52	1052.99940000	2.94590000	0.00000000
53	1056.14760000	1.90030000	0.00000000
54	1059.66650000	2.84610000	0.00000000
55	1067.06150000	8.90230000	0.00000000
56	1086.41340000	28.44190000	0.00000000
57	1122.06650000	5.05650000	0.00000000
58	1178.07510000	8.80920000	0.00000000
59	1186.08980000	1.05600000	0.00000000
60	1196.26050000	1.37060000	0.00000000

61	1198.19800000	19.79070000	0.00000000
62	1207.25460000	2.99320000	0.00000000
63	1214.68110000	0.60830000	0.00000000
64	1240.50370000	17.64760000	0.00000000
65	1253.85630000	23.71460000	0.00000000
66	1259.48950000	108.03970000	0.00000000
67	1285.56880000	3.08340000	0.00000000
68	1293.64390000	13.65030000	0.00000000
69	1304.38110000	2.91890000	0.00000000
70	1332.15380000	5.51440000	0.00000000
71	1335.79600000	1.54930000	0.00000000
72	1348.02990000	0.79100000	0.00000000
73	1355.76350000	9.07830000	0.00000000
74	1364.90800000	11.12670000	0.00000000
75	1369.67430000	19.74480000	0.00000000
76	1377.66080000	0.81840000	0.00000000
77	1382.36890000	101.16170000	0.00000000
78	1397.75560000	8.39110000	0.00000000
79	1403.77780000	11.59580000	0.00000000
80	1407.52160000	23.04190000	0.00000000
81	1438.46410000	9.84350000	0.00000000
82	1493.71490000	5.10100000	0.00000000
83	1496.88470000	7.12340000	0.00000000
84	1500.80040000	1.64100000	0.00000000
85	1505.84080000	2.84020000	0.00000000
86	1510.65360000	18.72770000	0.00000000
87	1514.89500000	17.34150000	0.00000000
88	1516.38770000	7.52050000	0.00000000
89	1538.31850000	3.14200000	0.00000000
90	1540.60550000	8.10620000	0.00000000
91	1630.72000000	2.43300000	0.00000000
92	1647.98640000	2.78760000	0.00000000
93	1716.29160000	349.17840000	0.00000000
94	2210.57640000	367.40780000	0.00000000
95	3073.55900000	8.45940000	0.00000000
96	3075.05080000	4.26760000	0.00000000
97	3078.58290000	3.58990000	0.00000000
98	3082.77540000	9.73950000	0.00000000
99	3086.03050000	1.58150000	0.00000000
100	3089.23210000	4.73890000	0.00000000
101	3096.89120000	6.09290000	0.00000000
102	3120.67980000	2.51350000	0.00000000
103	3123.92040000	1.32210000	0.00000000
104	3125.87690000	2.67540000	0.00000000
105	3129.30090000	1.47560000	0.00000000
106	3140.61540000	4.26930000	0.00000000
107	3151.11680000	1.59160000	0.00000000
108	3154.94970000	3.76500000	0.00000000
109	3166.92500000	5.69620000	0.00000000
110	3169.29570000	6.27780000	0.00000000
111	3188.07380000	0.93030000	0.00000000
112	3197.61010000	4.45960000	0.00000000
113	3206.79220000	1.61380000	0.00000000
114	3221.67180000	26.68600000	0.00000000

S50. CALCULATIONS ON TS 10 (ISOMER 1) ⟶ 10 (ISOMER 2)

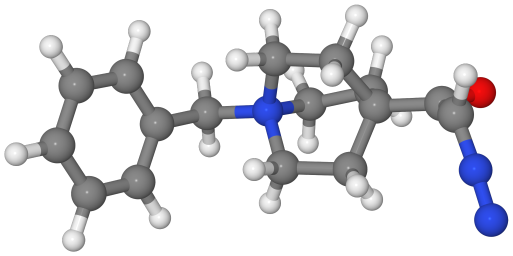

Route	:	# opt=(calcfc,ts,noeigentest) freq b3lyp/cc-pvtz empiricaldispersion=gd3bj pop=regular geom=connectivity int=ultrafine
SMILES	:	c1ccc(cc1)C[N]23CCC(CC2)(CC3)C(=O)[CH][N][N]
Formula	:	$C_{16}H_{20}N_3O^+$
Charge	:	1
Multiplicity	:	1
Energy	:	-861.30412260 a.u.
Gibbs Energy	:	-861.00806700 a.u.

Cartesian Co-ordinates (XYZ format)

40

C	0.18334299	0.23315100	-1.45294499
C	-0.29533699	0.49656799	-0.01312300
C	1.68980896	-0.06976800	-1.43378198
H	-0.37191400	-0.59880000	-1.88054204
H	0.00402700	1.10378003	-2.08184409
H	1.94334698	-0.92212802	-2.05428004
H	2.28592610	0.78162098	-1.75377405
C	-0.18992700	-0.83201200	0.75934601
H	-0.37264299	-0.65614700	1.81847894
H	-0.93799698	-1.53871799	0.40796301
C	1.20835400	-1.43404901	0.54702502
H	1.64995003	-1.77655900	1.47832501
H	1.20331705	-2.26419711	-0.15162100
C	2.03385997	0.86114401	0.81241298
H	2.21767807	0.55172902	1.83858204
H	2.84266090	1.51836598	0.50501102
C	0.65449798	1.50855505	0.63438302
H	0.26591700	1.82809103	1.59762394
H	0.72193903	2.40045691	0.01453800
N	2.14023805	-0.39404699	-0.02921300
C	3.59543800	-0.87155402	-0.00919600
H	4.16714811	-0.05406000	-0.44271001
H	3.85274792	-0.94994801	1.04484296
C	-1.73579299	1.00768006	-0.00663600
O	-2.01920891	2.05955410	0.50057501
C	-2.76594996	0.13776100	-0.69509101
H	-3.12208796	0.37568900	-1.68484998
N	-3.51997805	-0.62023699	0.03834000
N	-4.12885094	-1.30548096	0.69753301
C	3.85513401	-2.15728593	-0.73093802
C	3.77281404	-3.37719011	-0.05815600
C	4.22530508	-2.14833212	-2.07672811
C	4.02369404	-4.56723690	-0.72607702

H	3.53104997	-3.39944911	0.99637902
C	4.47701883	-3.33804107	-2.74576211
H	4.33974695	-1.20784795	-2.60011196
C	4.36927223	-4.54895592	-2.07241893
H	3.96303201	-5.50591183	-0.19424000
H	4.76966906	-3.31923103	-3.78577089
H	4.57118320	-5.47537279	-2.59088898

Frequencies

Mode	IR frequency	IR intensity	Raman intensity
1	-214.28990000	23.23150000	0.00000000
2	26.91540000	1.41340000	0.00000000
3	43.98890000	0.19320000	0.00000000
4	55.97630000	0.26900000	0.00000000
5	72.75280000	2.37920000	0.00000000
6	98.48400000	0.68430000	0.00000000
7	116.08120000	0.09540000	0.00000000
8	130.50900000	4.10470000	0.00000000
9	170.60160000	0.31040000	0.00000000
10	201.36990000	9.16560000	0.00000000
11	226.63530000	2.63220000	0.00000000
12	236.75580000	0.71010000	0.00000000
13	253.71250000	6.28950000	0.00000000
14	312.43140000	1.28850000	0.00000000
15	329.13190000	4.46290000	0.00000000
16	358.78080000	11.91340000	0.00000000
17	374.88420000	0.55030000	0.00000000
18	392.58650000	1.00870000	0.00000000
19	418.45100000	0.03650000	0.00000000
20	422.89230000	0.00450000	0.00000000
21	455.48920000	0.32920000	0.00000000
22	475.16340000	8.21590000	0.00000000
23	519.48360000	0.65950000	0.00000000
24	536.36890000	3.14600000	0.00000000
25	541.87360000	0.10270000	0.00000000
26	556.74660000	4.14660000	0.00000000
27	585.04540000	2.79240000	0.00000000
28	625.18000000	19.06970000	0.00000000
29	639.18220000	0.09940000	0.00000000
30	653.86820000	42.26290000	0.00000000
31	704.06770000	2.79990000	0.00000000
32	724.72080000	45.40150000	0.00000000
33	786.45360000	37.77050000	0.00000000
34	800.62360000	8.45950000	0.00000000
35	812.02380000	13.48220000	0.00000000
36	833.58940000	9.53620000	0.00000000
37	838.86460000	6.03410000	0.00000000
38	851.18860000	50.12890000	0.00000000
39	855.53990000	2.62440000	0.00000000
40	869.71960000	0.08270000	0.00000000
41	906.17020000	6.04860000	0.00000000
42	934.52760000	43.94450000	0.00000000
43	955.16110000	0.85570000	0.00000000
44	987.58610000	2.81980000	0.00000000
45	996.32280000	1.01660000	0.00000000
46	1002.65940000	10.53170000	0.00000000
47	1006.16520000	1.38320000	0.00000000
48	1007.92930000	7.87110000	0.00000000
49	1017.27850000	2.97150000	0.00000000
50	1028.33710000	3.91850000	0.00000000
51	1036.41090000	0.06680000	0.00000000
52	1046.92080000	23.30180000	0.00000000
53	1051.00350000	2.47200000	0.00000000
54	1056.40390000	1.34010000	0.00000000
55	1062.15070000	3.72090000	0.00000000
56	1069.63390000	14.66880000	0.00000000
57	1121.99650000	5.16400000	0.00000000
58	1165.67280000	99.08570000	0.00000000
59	1185.40120000	1.88440000	0.00000000
60	1195.74330000	28.08410000	0.00000000

61	1196.47140000	2.52380000	0.00000000
62	1204.89100000	1.45370000	0.00000000
63	1214.37370000	0.37280000	0.00000000
64	1217.64630000	48.66560000	0.00000000
65	1240.49740000	17.71890000	0.00000000
66	1256.02360000	0.49910000	0.00000000
67	1282.80600000	1.05340000	0.00000000
68	1294.30490000	7.49350000	0.00000000
69	1304.72690000	4.49430000	0.00000000
70	1326.12850000	1.44900000	0.00000000
71	1336.15130000	2.53820000	0.00000000
72	1346.92420000	11.63630000	0.00000000
73	1347.92880000	0.04210000	0.00000000
74	1363.17490000	16.29180000	0.00000000
75	1369.31510000	83.49660000	0.00000000
76	1369.89380000	13.67870000	0.00000000
77	1377.21690000	3.65720000	0.00000000
78	1392.39870000	2.23870000	0.00000000
79	1403.58410000	14.34840000	0.00000000
80	1406.96930000	10.46880000	0.00000000
81	1438.61900000	10.05460000	0.00000000
82	1493.27470000	4.81690000	0.00000000
83	1496.97620000	7.82970000	0.00000000
84	1501.52070000	1.15410000	0.00000000
85	1506.15250000	0.87550000	0.00000000
86	1511.02010000	19.12410000	0.00000000
87	1515.57380000	5.46340000	0.00000000
88	1516.34830000	18.18130000	0.00000000
89	1538.31070000	3.19700000	0.00000000
90	1540.90280000	6.11560000	0.00000000
91	1630.66070000	2.44240000	0.00000000
92	1647.93090000	2.87100000	0.00000000
93	1785.16480000	185.35700000	0.00000000
94	2204.12010000	477.01210000	0.00000000
95	3066.49320000	7.44610000	0.00000000
96	3067.72690000	5.58750000	0.00000000
97	3075.08190000	6.64290000	0.00000000
98	3079.62300000	2.74040000	0.00000000
99	3083.80310000	6.21570000	0.00000000
100	3087.54420000	4.52470000	0.00000000
101	3097.43180000	6.35040000	0.00000000
102	3109.80250000	3.82520000	0.00000000
103	3113.17200000	3.23400000	0.00000000
104	3119.83530000	1.19430000	0.00000000
105	3128.28280000	0.32860000	0.00000000
106	3138.49880000	4.59620000	0.00000000
107	3151.20930000	1.19260000	0.00000000
108	3153.84070000	3.30280000	0.00000000
109	3166.91710000	5.77310000	0.00000000
110	3169.18420000	6.20660000	0.00000000
111	3188.16300000	0.90610000	0.00000000
112	3197.66970000	4.25640000	0.00000000
113	3206.84810000	1.59210000	0.00000000
114	3212.55260000	17.21240000	0.00000000

S51. CALCULATIONS ON 17s

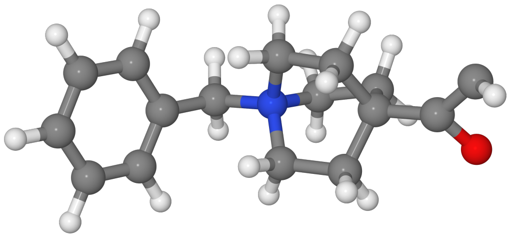

Route	:	# opt freq b3lyp/cc-pvtz empiricaldispersion=gd3bj pop=regular geom=connectivity int=ultrafine
SMILES	:	[CH]C(=O)C12CC[N](CC1)(CC2)Cc3ccccc3
Formula	:	$C_{16}H_{20}NO^{+}$
Charge	:	1
Multiplicity	:	1
Energy	:	-751.69195828 a.u.
Gibbs Energy	:	-751.40565800 a.u.

Cartesian Co-ordinates (XYZ format)

38

C	-0.46353501	-1.14886498	-0.85042202
C	-0.91455001	0.02417600	0.02392800
C	1.05934596	-1.07083404	-1.04053700
H	-0.73996902	-2.09200001	-0.38110000
H	-0.95435798	-1.11125600	-1.82158303
H	1.54066503	-2.03188610	-0.89931899
H	1.33479500	-0.68668598	-2.01991892
C	-0.35423300	-0.17717400	1.44273698
H	-0.54225200	0.71237999	2.03975010
H	-0.85476100	-1.00806403	1.93601000
C	1.15050101	-0.47415501	1.34490895
H	1.72843802	0.10649200	2.05811191
H	1.37903297	-1.52371800	1.49770701
C	1.21143103	1.28665304	-0.35999301
H	1.54276204	1.91217697	0.46531701
H	1.74327898	1.59291899	-1.25655901
C	-0.31064799	1.32302296	-0.54688102
H	-0.73044801	2.18373489	-0.03197900
H	-0.57056999	1.41318202	-1.60006905
N	1.66504300	-0.11979800	-0.03193800
C	3.19649696	-0.15997800	-0.08457700
C	-2.40011501	0.15593500	0.08024200
O	-3.03875089	1.00283206	0.76860398
C	-3.46074009	-0.28077000	-0.64008498
H	-4.21601677	-0.96976602	-0.28355199
H	3.52285099	0.64019102	0.57608199
H	3.45122504	0.11208100	-1.10642600
C	3.81304002	-1.46928000	0.29813400
C	4.08727312	-2.43099809	-0.67569500
C	4.16140509	-1.72822499	1.62454605
C	4.66896677	-3.64153910	-0.32563299
H	3.86706901	-2.22629905	-1.71548498
C	4.74255610	-2.93827391	1.97567403

H	3.99740696	-0.97604400	2.38502789
C	4.99036598	-3.89874291	1.00173497
H	4.88334703	-4.37623405	-1.08843994
H	5.01408005	-3.12629008	3.00453591
H	5.44916201	-4.83846378	1.27424395

Frequencies

Mode	IR frequency	IR intensity	Raman intensity
1	31.90940000	4.06710000	0.00000000
2	41.19660000	1.22000000	0.00000000
3	59.51290000	1.30100000	0.00000000
4	76.88310000	1.76310000	0.00000000
5	115.31260000	0.42010000	0.00000000
6	125.93320000	4.91220000	0.00000000
7	131.67450000	2.44930000	0.00000000
8	211.70410000	0.66960000	0.00000000
9	229.70550000	1.22860000	0.00000000
10	238.14150000	0.78380000	0.00000000
11	287.02280000	7.27470000	0.00000000
12	310.80680000	2.19920000	0.00000000
13	317.63770000	5.67830000	0.00000000
14	361.75720000	0.10550000	0.00000000
15	394.07480000	2.37000000	0.00000000
16	417.99980000	0.01750000	0.00000000
17	421.81360000	0.16600000	0.00000000
18	432.73630000	4.59850000	0.00000000
19	478.87030000	106.97490000	0.00000000
20	486.25930000	4.07810000	0.00000000
21	509.42700000	43.46660000	0.00000000
22	539.23690000	0.72230000	0.00000000
23	544.70680000	0.99890000	0.00000000
24	595.70500000	16.30410000	0.00000000
25	639.00950000	0.03960000	0.00000000
26	644.76000000	7.23880000	0.00000000
27	684.63060000	170.11350000	0.00000000
28	708.23560000	8.20690000	0.00000000
29	724.52040000	45.30810000	0.00000000
30	786.18110000	34.50610000	0.00000000
31	805.45030000	0.75000000	0.00000000
32	822.43180000	7.83780000	0.00000000
33	838.87060000	14.49780000	0.00000000
34	839.76450000	14.43500000	0.00000000
35	851.01480000	28.60970000	0.00000000
36	854.82470000	4.36280000	0.00000000
37	869.57180000	0.13610000	0.00000000
38	904.92290000	1.17220000	0.00000000
39	954.61330000	5.51170000	0.00000000
40	964.96920000	16.65120000	0.00000000
41	991.01160000	0.56860000	0.00000000
42	997.31370000	0.49370000	0.00000000
43	1002.87470000	1.44860000	0.00000000
44	1006.55080000	0.00920000	0.00000000
45	1012.26800000	6.32320000	0.00000000
46	1017.99320000	1.98710000	0.00000000
47	1028.29690000	3.99050000	0.00000000
48	1036.83480000	0.02510000	0.00000000
49	1052.73070000	2.76680000	0.00000000
50	1056.57260000	2.25830000	0.00000000
51	1061.33280000	1.96380000	0.00000000
52	1067.52140000	20.86970000	0.00000000
53	1122.14800000	5.54150000	0.00000000
54	1126.28030000	13.58470000	0.00000000
55	1191.26180000	1.43160000	0.00000000
56	1196.45200000	0.89330000	0.00000000
57	1197.71420000	2.41260000	0.00000000
58	1203.82810000	0.08100000	0.00000000
59	1214.76200000	0.94190000	0.00000000
60	1240.55460000	18.82950000	0.00000000

61	1256.08010000	0.48150000	0.00000000
62	1282.78420000	1.38630000	0.00000000
63	1294.70700000	4.57330000	0.00000000
64	1307.02540000	4.61610000	0.00000000
65	1326.59340000	4.40100000	0.00000000
66	1336.64720000	1.57370000	0.00000000
67	1345.87640000	4.66060000	0.00000000
68	1349.53220000	4.80040000	0.00000000
69	1364.00290000	12.57720000	0.00000000
70	1367.56520000	8.58840000	0.00000000
71	1377.38390000	0.26850000	0.00000000
72	1395.20910000	6.18160000	0.00000000
73	1402.66610000	9.19380000	0.00000000
74	1407.40270000	6.77310000	0.00000000
75	1431.07450000	54.00960000	0.00000000
76	1438.33880000	12.91110000	0.00000000
77	1493.05440000	4.15080000	0.00000000
78	1496.99450000	7.51340000	0.00000000
79	1504.34980000	3.60150000	0.00000000
80	1505.60940000	3.10300000	0.00000000
81	1513.40030000	19.37850000	0.00000000
82	1515.45960000	8.15780000	0.00000000
83	1516.96460000	16.76390000	0.00000000
84	1538.23950000	3.32390000	0.00000000
85	1541.20450000	6.62890000	0.00000000
86	1624.63200000	119.33700000	0.00000000
87	1630.62460000	2.48170000	0.00000000
88	1647.66440000	3.11290000	0.00000000
89	3063.08740000	6.04820000	0.00000000
90	3073.67720000	6.12450000	0.00000000
91	3075.02050000	5.18040000	0.00000000
92	3076.23390000	2.36920000	0.00000000
93	3084.65410000	4.91670000	0.00000000
94	3087.85530000	3.78880000	0.00000000
95	3097.58300000	6.10370000	0.00000000
96	3104.25830000	4.09050000	0.00000000
97	3114.35800000	1.56670000	0.00000000
98	3117.99080000	1.03320000	0.00000000
99	3128.55650000	0.47300000	0.00000000
100	3139.40790000	3.36550000	0.00000000
101	3152.30220000	0.97730000	0.00000000
102	3155.33710000	2.83390000	0.00000000
103	3166.87960000	5.82850000	0.00000000
104	3169.45080000	6.00780000	0.00000000
105	3188.39120000	0.86690000	0.00000000
106	3197.91230000	4.08430000	0.00000000
107	3203.05400000	19.16910000	0.00000000
108	3207.02170000	1.49400000	0.00000000

S52. CALCULATIONS ON TS 17s ⟶ 18s

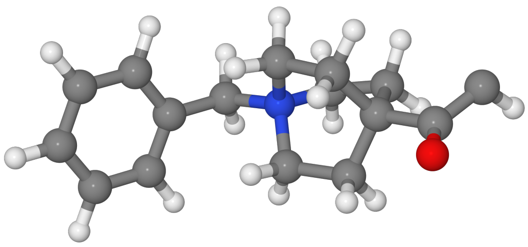

Route	:	# opt=(calcfc,ts) freq b3lyp/cc-pvtz empiricaldispersion=gd3bj pop=regular geom=connectivity int=ultrafine
SMILES	:	[CH]C(=O)C12CC[N](CC1)(CC2)Cc3ccccc3
Formula	:	$C_{16}H_{20}NO^{+}$
Charge	:	1
Multiplicity	:	1
Energy	:	-751.68247440 a.u.
Gibbs Energy	:	-751.39665400 a.u.

Cartesian Co-ordinates (XYZ format)

38

C	0.25499800	1.50215304	-0.24086200
C	0.93011802	0.13318101	-0.04825900
C	-1.25909495	1.27704597	-0.41291201
H	0.44464299	2.14770699	0.61516500
H	0.62901700	2.00946808	-1.12909901
H	-1.84539902	1.97155094	0.18214600
H	-1.57187295	1.36658299	-1.45022798
C	0.47606900	-0.43469200	1.30649698
H	0.83260000	-1.45756400	1.40004802
H	0.91159600	0.13936800	2.12166190
C	-1.05622804	-0.37162000	1.39401102
H	-1.48428202	-1.29972005	1.75616705
H	-1.40276301	0.44125000	2.02818799
C	-1.04301202	-1.10986304	-0.94739997
H	-1.20867896	-2.09416008	-0.52217001
H	-1.61260200	-1.03638804	-1.86934805
C	0.44630000	-0.80293798	-1.16334903
H	1.02445304	-1.72270095	-1.14149404
H	0.60407197	-0.33585501	-2.13482404
N	-1.64370799	-0.11785700	0.02512400
C	-3.17340207	-0.21321300	0.05174400
H	-3.49458194	0.10852700	-0.93654901
H	-3.49107504	0.53232998	0.77726799
C	2.49885392	0.10312500	-0.05625900
O	3.09295201	-0.96291202	-0.06359300
C	2.98603797	1.39660895	0.13747600
H	3.23946595	1.98882997	-0.74637198
C	-3.71941710	-1.56557405	0.38877600
C	-3.99236989	-2.49098706	-0.61954099
C	-4.00203705	-1.90202701	1.71369195
C	-4.50691414	-3.74088812	-0.30639201
H	-3.82104897	-2.23035693	-1.65573597
C	-4.51698923	-3.15187907	2.02796006

H	-3.83997297	-1.17859399	2.50226402
C	-4.76288891	-4.07485819	1.01848996
H	-4.71967220	-4.44823599	-1.09505403
H	-4.73771381	-3.40003395	3.05619502
H	-5.16907215	-5.04624891	1.26184499

Frequencies

Mode	IR frequency	IR intensity	Raman intensity
1	-373.45620000	0.51340000	0.00000000
2	33.82980000	0.61470000	0.00000000
3	56.95590000	2.80690000	0.00000000
4	59.53630000	0.69500000	0.00000000
5	83.02460000	1.76430000	0.00000000
6	117.11550000	0.04620000	0.00000000
7	136.90720000	5.34570000	0.00000000
8	158.33540000	1.59940000	0.00000000
9	216.54740000	7.00690000	0.00000000
10	238.03470000	0.39190000	0.00000000
11	245.48040000	0.17550000	0.00000000
12	315.63920000	6.01280000	0.00000000
13	321.76910000	0.86860000	0.00000000
14	361.60070000	1.08470000	0.00000000
15	376.51770000	1.06800000	0.00000000
16	395.78710000	0.68290000	0.00000000
17	417.55580000	0.03290000	0.00000000
18	421.86010000	0.04720000	0.00000000
19	469.49000000	4.87700000	0.00000000
20	522.94490000	0.89010000	0.00000000
21	540.19240000	0.65090000	0.00000000
22	543.93800000	0.18900000	0.00000000
23	587.89300000	40.63040000	0.00000000
24	589.26350000	74.09470000	0.00000000
25	639.03970000	0.12470000	0.00000000
26	644.41670000	11.23380000	0.00000000
27	694.77670000	48.89850000	0.00000000
28	707.44970000	11.94190000	0.00000000
29	724.60210000	46.06870000	0.00000000
30	786.08670000	35.47890000	0.00000000
31	807.27450000	1.08890000	0.00000000
32	831.73800000	40.89550000	0.00000000
33	835.20210000	13.98380000	0.00000000
34	840.70910000	19.78430000	0.00000000
35	852.97860000	57.90730000	0.00000000
36	855.08560000	7.45340000	0.00000000
37	869.68050000	0.09650000	0.00000000
38	907.86520000	4.44280000	0.00000000
39	938.55660000	22.66840000	0.00000000
40	955.42160000	1.68730000	0.00000000
41	980.29380000	0.25740000	0.00000000
42	990.10320000	0.14300000	0.00000000
43	998.47860000	0.37470000	0.00000000
44	1006.43940000	0.08930000	0.00000000
45	1008.24580000	5.09950000	0.00000000
46	1017.14190000	1.01980000	0.00000000
47	1028.40740000	4.16580000	0.00000000
48	1036.70780000	0.03620000	0.00000000
49	1048.75770000	7.78520000	0.00000000
50	1055.38090000	6.04490000	0.00000000
51	1056.33370000	2.01990000	0.00000000
52	1059.51230000	3.16430000	0.00000000
53	1089.02120000	29.20790000	0.00000000
54	1122.35260000	4.82660000	0.00000000
55	1127.90890000	5.02860000	0.00000000
56	1187.49630000	1.39220000	0.00000000
57	1196.69370000	0.16090000	0.00000000
58	1205.50100000	0.25600000	0.00000000
59	1209.74600000	7.91780000	0.00000000
60	1215.02450000	0.50620000	0.00000000

61	1240.68290000	18.55450000	0.00000000
62	1256.65750000	1.46060000	0.00000000
63	1281.42800000	2.76320000	0.00000000
64	1300.18790000	10.72140000	0.00000000
65	1304.20240000	3.98430000	0.00000000
66	1329.13350000	1.12650000	0.00000000
67	1335.94670000	2.78880000	0.00000000
68	1347.38050000	10.47750000	0.00000000
69	1347.99360000	0.60680000	0.00000000
70	1362.91970000	4.75870000	0.00000000
71	1370.50690000	10.95920000	0.00000000
72	1376.49430000	1.00750000	0.00000000
73	1386.20020000	2.22520000	0.00000000
74	1401.84070000	13.33750000	0.00000000
75	1404.97340000	11.79780000	0.00000000
76	1438.02350000	9.58510000	0.00000000
77	1493.26590000	3.59310000	0.00000000
78	1497.12830000	7.77960000	0.00000000
79	1504.47210000	3.83090000	0.00000000
80	1506.40540000	3.20760000	0.00000000
81	1513.91670000	18.94400000	0.00000000
82	1514.74600000	9.08910000	0.00000000
83	1517.53620000	17.84200000	0.00000000
84	1538.26640000	3.09070000	0.00000000
85	1541.03120000	3.91220000	0.00000000
86	1630.68290000	2.43600000	0.00000000
87	1647.62980000	3.34920000	0.00000000
88	1670.22120000	176.99790000	0.00000000
89	3059.16160000	6.43310000	0.00000000
90	3064.80030000	8.06280000	0.00000000
91	3075.06710000	5.12620000	0.00000000
92	3078.44670000	1.72970000	0.00000000
93	3084.60310000	3.14540000	0.00000000
94	3087.12700000	6.75780000	0.00000000
95	3093.69680000	2.58260000	0.00000000
96	3097.25430000	5.35480000	0.00000000
97	3098.93570000	3.82660000	0.00000000
98	3121.26980000	1.00170000	0.00000000
99	3123.37920000	3.43940000	0.00000000
100	3127.57620000	0.60260000	0.00000000
101	3138.73400000	3.32230000	0.00000000
102	3152.23480000	1.70040000	0.00000000
103	3155.25940000	1.64120000	0.00000000
104	3167.41160000	5.84000000	0.00000000
105	3169.36870000	6.52390000	0.00000000
106	3188.57070000	0.89050000	0.00000000
107	3198.04510000	4.15280000	0.00000000
108	3207.18420000	1.51180000	0.00000000

S53. CALCULATIONS ON TS 10 (ISOMER 1) ⟶ 18s

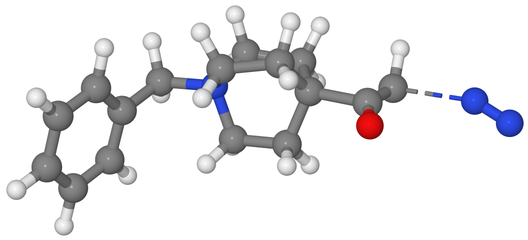

Route	:	# opt=(calcfc,ts,noeigentest) freq b3lyp/cc-pvtz empiricaldispersion=gd3bj pop=regular geom=connectivity int=ultrafine
SMILES	:	[CH]C(=O)C12CC[N](CC1)(CC2)Cc3ccccc3.[N]=[N]
Formula	:	$C_{16}H_{20}N_3O^+$
Charge	:	1
Multiplicity	:	1
Energy	:	-861.26529657 a.u.
Gibbs Energy	:	-860.97687400 a.u.

Cartesian Co-ordinates (XYZ format)

40

C	0.23316000	-0.53373998	1.25171494
C	-0.20009600	0.04011000	-0.10148700
C	1.75414503	-0.37917399	1.40913796
H	-0.27173200	-0.00771200	2.05902505
H	-0.05792800	-1.57942104	1.30676997
H	2.02238488	0.43235099	2.08180809
H	2.22302103	-1.29095995	1.76205802
C	0.39122000	1.45370102	-0.22800900
H	0.03069200	1.96296799	-1.11867297
H	0.11848100	2.05914497	0.63269901
C	1.92589200	1.33346498	-0.33528101
H	2.27924109	1.47965503	-1.35308504
H	2.43469310	2.04521203	0.30905300
C	1.88897002	-1.03720403	-0.95279598
H	2.49277711	-0.89807099	-1.84497201
H	2.09457493	-2.02414107	-0.55178303
C	0.39273000	-0.81614202	-1.22843397
H	0.24957100	-0.31911299	-2.18709207
H	-0.11472400	-1.77566099	-1.26923096
N	2.38306189	-0.04534500	0.07654900
C	3.91215396	-0.04572900	0.16865499
C	-1.84560204	-0.28784701	-0.17068499
O	-2.18299603	-1.44221795	-0.12770000
C	-2.23972201	0.99878800	-0.04257300
H	-2.23792291	1.69179499	-0.87354499
H	4.25446510	0.32757199	-0.79408401
H	4.15050077	0.69405401	0.92959797
N	-4.47066021	0.72780901	-0.12352700
N	-5.46208000	0.45815501	0.24297699
C	4.52865982	-1.37168300	0.48951301
C	4.89982605	-2.24683094	-0.53218901
C	4.77764082	-1.73142004	1.81496704
C	5.47826910	-3.47204709	-0.23331501

H	4.75437880	-1.96522605	-1.56683099
C	5.35667181	-2.95648789	2.11508298
H	4.53788710	-1.04442000	2.61619210
C	5.70068216	-3.83081388	1.09112501
H	5.76643181	-4.14023590	-1.03213000
H	5.55021191	-3.22260594	3.14431000
H	6.15643883	-4.78266621	1.32368195

Frequencies

Mode	IR frequency	IR intensity	Raman intensity
1	-322.33510000	92.60840000	0.00000000
2	28.69380000	0.50690000	0.00000000
3	32.50820000	0.24200000	0.00000000
4	39.46710000	0.00280000	0.00000000
5	59.86160000	0.61980000	0.00000000
6	69.78360000	1.04950000	0.00000000
7	84.78590000	2.19320000	0.00000000
8	95.21120000	0.67950000	0.00000000
9	120.44560000	0.08390000	0.00000000
10	145.72430000	1.85930000	0.00000000
11	157.18850000	9.54200000	0.00000000
12	201.21750000	6.35320000	0.00000000
13	213.22110000	3.70080000	0.00000000
14	231.38060000	9.79260000	0.00000000
15	246.89770000	0.16880000	0.00000000
16	260.25850000	0.24480000	0.00000000
17	317.51530000	1.31440000	0.00000000
18	335.53130000	0.27060000	0.00000000
19	356.55600000	7.68280000	0.00000000
20	388.46590000	0.78420000	0.00000000
21	402.92940000	0.02930000	0.00000000
22	418.45730000	0.03490000	0.00000000
23	422.92490000	0.03320000	0.00000000
24	468.74220000	1.65240000	0.00000000
25	537.82260000	4.44500000	0.00000000
26	543.27570000	0.82250000	0.00000000
27	561.96050000	76.59700000	0.00000000
28	577.14300000	16.12000000	0.00000000
29	614.21880000	6.63220000	0.00000000
30	639.07890000	0.08330000	0.00000000
31	652.73610000	30.66760000	0.00000000
32	700.76990000	1.02000000	0.00000000
33	724.65010000	46.62890000	0.00000000
34	755.86450000	53.59590000	0.00000000
35	786.30820000	34.75350000	0.00000000
36	808.65760000	1.59750000	0.00000000
37	819.15520000	15.57540000	0.00000000
38	835.05840000	4.34150000	0.00000000
39	844.39220000	7.60000000	0.00000000
40	855.82060000	3.14520000	0.00000000
41	869.61040000	0.14410000	0.00000000
42	902.09340000	54.12860000	0.00000000
43	906.58150000	1.55120000	0.00000000
44	952.82490000	13.65730000	0.00000000
45	956.13650000	4.92340000	0.00000000
46	987.68220000	1.38420000	0.00000000
47	991.95750000	0.53320000	0.00000000
48	996.78060000	1.70320000	0.00000000
49	1006.13720000	0.00950000	0.00000000
50	1011.09750000	2.18500000	0.00000000
51	1018.29110000	1.41800000	0.00000000
52	1028.46740000	3.99060000	0.00000000
53	1036.12400000	0.84340000	0.00000000
54	1038.01800000	9.34230000	0.00000000
55	1047.72640000	2.08670000	0.00000000
56	1056.52780000	1.47750000	0.00000000
57	1057.78740000	2.72530000	0.00000000
58	1076.42820000	23.26380000	0.00000000
59	1122.31580000	5.01610000	0.00000000
60	1137.52640000	7.98930000	0.00000000

61	1189.49030000	0.95770000	0.00000000
62	1196.56000000	0.11240000	0.00000000
63	1208.12710000	1.09330000	0.00000000
64	1209.74190000	5.83420000	0.00000000
65	1214.93010000	1.14830000	0.00000000
66	1240.59590000	19.30710000	0.00000000
67	1255.60720000	1.11940000	0.00000000
68	1280.51390000	2.95600000	0.00000000
69	1299.26880000	9.38800000	0.00000000
70	1309.29010000	7.53890000	0.00000000
71	1329.50760000	2.15530000	0.00000000
72	1334.40170000	2.47450000	0.00000000
73	1346.47580000	6.64270000	0.00000000
74	1347.62700000	4.50150000	0.00000000
75	1363.30080000	6.42490000	0.00000000
76	1369.20280000	13.14800000	0.00000000
77	1376.91370000	0.46660000	0.00000000
78	1378.72060000	3.97430000	0.00000000
79	1401.75780000	14.11590000	0.00000000
80	1405.38230000	12.23530000	0.00000000
81	1436.93590000	9.95030000	0.00000000
82	1493.10470000	3.68270000	0.00000000
83	1497.12510000	7.68530000	0.00000000
84	1502.89660000	1.93220000	0.00000000
85	1507.01910000	2.65650000	0.00000000
86	1511.67830000	17.62100000	0.00000000
87	1515.37390000	13.42290000	0.00000000
88	1517.38190000	16.19590000	0.00000000
89	1538.29360000	3.05210000	0.00000000
90	1540.93170000	6.07860000	0.00000000
91	1630.75700000	2.41630000	0.00000000
92	1647.90180000	3.11030000	0.00000000
93	1876.06710000	274.70530000	0.00000000
94	2447.98420000	5.02120000	0.00000000
95	3065.51190000	7.95080000	0.00000000
96	3073.64640000	9.58510000	0.00000000
97	3076.13500000	0.18530000	0.00000000
98	3080.05390000	0.74600000	0.00000000
99	3083.89020000	7.34670000	0.00000000
100	3086.68950000	7.12960000	0.00000000
101	3096.33230000	8.01560000	0.00000000
102	3114.97470000	2.28040000	0.00000000
103	3124.36700000	0.57660000	0.00000000
104	3125.79850000	4.41070000	0.00000000
105	3127.80340000	0.74970000	0.00000000
106	3138.82590000	4.96370000	0.00000000
107	3152.12830000	1.75140000	0.00000000
108	3155.29560000	1.70040000	0.00000000
109	3167.49430000	5.89150000	0.00000000
110	3169.55930000	6.66160000	0.00000000
111	3188.35360000	0.96610000	0.00000000
112	3197.86010000	4.43570000	0.00000000
113	3207.02480000	1.69590000	0.00000000
114	3210.96810000	4.80170000	0.00000000

S54. CALCULATIONS ON 18s

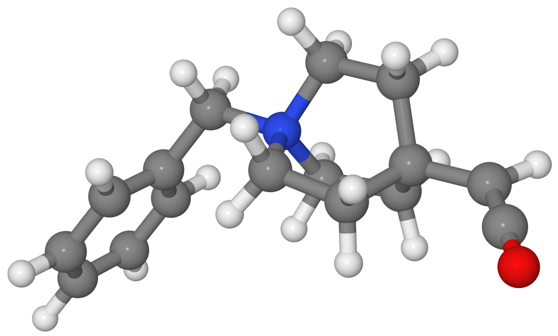

Route	:	# opt freq b3lyp/cc-pvtz empiricaldispersion=gd3bj pop=regular geom=connectivity int=ultrafine
SMILES	:	c1ccc(cc1)C[N]23CCC(CC2)(CC3)[CH][C][O]
Formula	:	$C_{16}H_{20}NO^{+}$
Charge	:	1
Multiplicity	:	1
Energy	:	-751.80670594 a.u.
Gibbs Energy	:	-751.51750700 a.u.

Cartesian Co-ordinates (XYZ format)

38

C	-1.01868403	-0.14111200	-1.48258603
C	-1.61254895	0.42105100	-0.17525899
C	0.51293403	-0.19276699	-1.38351595
H	-1.42860198	-1.13236499	-1.66779804
H	-1.30610502	0.48641399	-2.32372808
H	0.91837299	-1.14095604	-1.72627902
H	0.99321800	0.60548103	-1.94433403
C	-1.31767094	-0.59163803	0.94082701
H	-1.57952797	-0.16154300	1.90668797
H	-1.91982305	-1.48964000	0.81132698
C	0.16797300	-0.98169798	0.91451699
H	0.61540198	-0.96068001	1.90187597
H	0.32806200	-1.96787703	0.48474899
C	0.62348199	1.39279199	0.48385099
H	0.84132600	1.44820797	1.54534900
H	1.31417298	2.04927492	-0.03799500
C	-0.84524602	1.71456003	0.16552700
H	-1.30602396	2.19931006	1.02287197
H	-0.91167998	2.40859294	-0.67155898
N	0.95043302	-0.01861200	0.05412000
C	2.45409393	-0.29619199	0.13934100
C	-4.01647520	0.09321900	0.36712700
O	-4.82852411	-0.44762701	0.98928899
C	-3.08422494	0.69845599	-0.33104399
H	-3.42025805	1.43374896	-1.04960406
H	2.90508699	0.35799500	-0.60363501
H	2.57149410	-1.32545698	-0.19227099
C	3.06638503	-0.08910000	1.48977005
C	3.59455204	1.15266895	1.84553897
C	3.15544295	-1.14843202	2.39424610

C	4.17139387	1.34047997	3.09341502
H	3.57328200	1.97281504	1.13993597
C	3.73251796	-0.96190602	3.64265704
H	2.79240298	-2.12935209	2.11592007
C	4.23463488	0.28491700	3.99569702
H	4.58253098	2.30452394	3.35657692
H	3.80197906	-1.79050303	4.33274412
H	4.68964911	0.42946899	4.96518707

Frequencies

Mode	IR frequency	IR intensity	Raman intensity
1	29.97880000	0.98590000	0.00000000
2	40.89680000	0.25340000	0.00000000
3	62.30210000	0.41920000	0.00000000
4	79.79020000	0.72940000	0.00000000
5	100.27720000	0.00820000	0.00000000
6	108.47020000	1.16690000	0.00000000
7	187.89980000	1.72610000	0.00000000
8	208.69070000	0.56890000	0.00000000
9	241.86900000	0.75110000	0.00000000
10	259.91380000	1.44260000	0.00000000
11	268.10690000	0.98170000	0.00000000
12	342.11740000	1.16190000	0.00000000
13	362.63710000	3.52250000	0.00000000
14	400.06020000	0.16600000	0.00000000
15	402.30240000	1.07320000	0.00000000
16	418.33440000	0.07220000	0.00000000
17	422.82400000	0.00700000	0.00000000
18	434.74780000	0.59610000	0.00000000
19	489.56620000	3.75150000	0.00000000
20	532.97980000	1.20040000	0.00000000
21	538.76370000	0.67970000	0.00000000
22	543.51940000	0.55840000	0.00000000
23	590.96870000	12.90160000	0.00000000
24	599.46690000	42.23900000	0.00000000
25	639.06250000	0.48830000	0.00000000
26	639.91120000	10.46030000	0.00000000
27	699.66620000	3.15110000	0.00000000
28	724.36340000	45.02180000	0.00000000
29	756.10400000	1.87550000	0.00000000
30	786.47580000	32.55860000	0.00000000
31	807.15730000	0.82540000	0.00000000
32	832.00140000	3.66400000	0.00000000
33	837.99770000	4.20190000	0.00000000
34	854.30590000	1.68410000	0.00000000
35	859.44480000	27.89750000	0.00000000
36	869.37890000	0.06910000	0.00000000
37	905.49830000	1.32490000	0.00000000
38	954.85910000	2.87060000	0.00000000
39	968.57920000	27.28990000	0.00000000
40	986.47020000	1.04280000	0.00000000
41	992.52520000	3.77750000	0.00000000
42	1006.05950000	0.02430000	0.00000000
43	1010.17440000	6.11020000	0.00000000
44	1015.32080000	8.59610000	0.00000000
45	1019.14860000	2.93170000	0.00000000
46	1028.40030000	3.92920000	0.00000000
47	1036.18600000	0.01910000	0.00000000
48	1050.64540000	2.54870000	0.00000000
49	1054.74970000	5.28960000	0.00000000
50	1056.47420000	2.72050000	0.00000000
51	1069.92490000	29.70390000	0.00000000
52	1114.21390000	2.65020000	0.00000000
53	1121.90680000	5.33970000	0.00000000
54	1150.84260000	1.08040000	0.00000000
55	1190.74840000	4.70930000	0.00000000
56	1196.40340000	0.07660000	0.00000000
57	1204.39030000	0.24710000	0.00000000
58	1210.19330000	0.76390000	0.00000000
59	1214.63530000	1.05740000	0.00000000
60	1240.85400000	18.74480000	0.00000000

61	1256.67190000	0.27730000	0.00000000
62	1281.69630000	3.00040000	0.00000000
63	1301.23590000	7.46620000	0.00000000
64	1304.18040000	3.58340000	0.00000000
65	1326.96940000	5.95710000	0.00000000
66	1332.05340000	2.12420000	0.00000000
67	1344.71290000	4.64070000	0.00000000
68	1348.19820000	2.99910000	0.00000000
69	1363.55130000	7.38360000	0.00000000
70	1368.43020000	13.21160000	0.00000000
71	1376.96020000	0.34680000	0.00000000
72	1391.53360000	6.15930000	0.00000000
73	1403.27950000	13.72670000	0.00000000
74	1408.27330000	9.11220000	0.00000000
75	1429.08300000	8.34150000	0.00000000
76	1438.64420000	10.64560000	0.00000000
77	1492.53580000	4.42650000	0.00000000
78	1496.94280000	6.67800000	0.00000000
79	1501.14240000	2.90800000	0.00000000
80	1504.13220000	2.84170000	0.00000000
81	1511.08400000	19.37120000	0.00000000
82	1514.61400000	4.84320000	0.00000000
83	1515.77450000	12.45190000	0.00000000
84	1538.21660000	3.07480000	0.00000000
85	1540.63850000	10.92040000	0.00000000
86	1630.75790000	2.42780000	0.00000000
87	1647.95630000	2.94280000	0.00000000
88	2224.08450000	764.31120000	0.00000000
89	3060.69540000	7.89690000	0.00000000
90	3064.13660000	10.20150000	0.00000000
91	3069.07370000	13.97540000	0.00000000
92	3075.00150000	2.71970000	0.00000000
93	3082.69250000	6.08300000	0.00000000
94	3085.91490000	4.33380000	0.00000000
95	3094.63560000	7.32260000	0.00000000
96	3101.54720000	4.46850000	0.00000000
97	3107.24020000	3.50750000	0.00000000
98	3110.60110000	4.15860000	0.00000000
99	3127.05670000	0.16750000	0.00000000
100	3135.76320000	5.66980000	0.00000000
101	3149.69160000	2.01790000	0.00000000
102	3153.33990000	3.05260000	0.00000000
103	3167.19470000	5.79560000	0.00000000
104	3169.57820000	6.12200000	0.00000000
105	3180.33720000	9.44120000	0.00000000
106	3188.11880000	0.95460000	0.00000000
107	3197.63370000	4.52140000	0.00000000
108	3206.80920000	1.69600000	0.00000000

S55. CALCULATIONS ON 10 (ISOMER 1; TRIPLET)

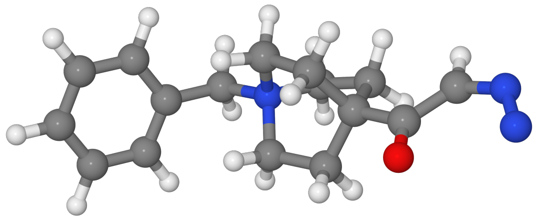

Route	:	# opt freq ub3lyp/cc-pvtz empiricaldispersion=gd3bj pop=regular geom=connectivity int=ultrafine
SMILES	:	c1ccc(cc1)C[N]23CCC(CC2)(CC3)C(=O)[CH]N=[N]
Formula	:	$C_{16}H_{20}N_3O^{+,3}$
Charge	:	1
Multiplicity	:	3
Energy	:	-861.25656424 a.u.
Gibbs Energy	:	-860.96588900 a.u.

Cartesian Co-ordinates (XYZ format)

```
40

C  -0.27803299  1.32434595 -0.48495501
C   0.28189299 -0.04979800 -0.09006600
C  -1.80544996  1.21622097 -0.62787300
H  -0.03267500  2.07140398  0.26839000
H   0.14208400  1.66219497 -1.43100703
H  -2.31985402  2.05719399 -0.17119400
H  -2.11730695  1.14895797 -1.66714203
C  -0.20311600 -0.35841000  1.34131205
H   0.04098000 -1.38999999  1.58652306
H   0.30606601  0.27515799  2.06619096
C  -1.71668601 -0.11365000  1.43255901
H  -2.22881198 -0.91250902  1.95725298
H  -1.95631599  0.82919401  1.91914499
C  -1.83310997 -1.23184299 -0.74716401
H  -2.07275796 -2.10987091 -0.15629201
H  -2.42195606 -1.26551104 -1.65918005
C  -0.33196801 -1.09936798 -1.03065300
H   0.16072400 -2.05532598 -0.88007498
H  -0.16207400 -0.80653501 -2.06634307
N  -2.31365705 -0.03758000  0.04754000
C  -3.84387994  0.02504400  0.10403600
H  -4.15744877  0.20432100 -0.92210799
H  -4.07022619  0.91074002  0.69356799
C   1.81501997 -0.18274100 -0.11294500
O   2.31239796 -1.29453504 -0.13889199
C   2.62734509  1.02327204 -0.02916900
H   2.18636394  2.00730991 -0.00817300
N   4.00887680  1.02331805  0.08367900
N   4.72127485  0.08383000 -0.12649900
C  -4.50816488 -1.18950701  0.67353702
C  -4.88816595 -2.24511099 -0.15640800
C  -4.79277182 -1.26250803  2.03814602
C  -5.51060677 -3.36658812  0.37272900
H  -4.71537304 -2.18597007 -1.22296405
```

C	-5.41589308	-2.38358498	2.56834602
H	-4.54709291	-0.43209401	2.68750191
C	-5.76857710	-3.43968797	1.73673606
H	-5.80536890	-4.17627478	-0.27927601
H	-5.63724804	-2.42720389	3.62507606
H	-6.25878811	-4.31061888	2.14779711

Frequencies

Mode	IR frequency	IR intensity	Raman intensity
1	12.99360000	1.82540000	0.00000000
2	37.28120000	0.16060000	0.00000000
3	37.96350000	2.87200000	0.00000000
4	53.91000000	1.73070000	0.00000000
5	70.48000000	6.98380000	0.00000000
6	87.79800000	3.13330000	0.00000000
7	112.90770000	1.12780000	0.00000000
8	136.28370000	0.26410000	0.00000000
9	167.97260000	1.37300000	0.00000000
10	200.72040000	0.51380000	0.00000000
11	208.95290000	0.63230000	0.00000000
12	236.00130000	1.33950000	0.00000000
13	254.38740000	1.19510000	0.00000000
14	292.38550000	1.54360000	0.00000000
15	325.28600000	0.26900000	0.00000000
16	342.63220000	8.12690000	0.00000000
17	378.94820000	0.90400000	0.00000000
18	399.29130000	1.09500000	0.00000000
19	418.35060000	0.02950000	0.00000000
20	422.61120000	0.04080000	0.00000000
21	452.19040000	1.32550000	0.00000000
22	474.35400000	6.90000000	0.00000000
23	501.72060000	8.14500000	0.00000000
24	540.45350000	0.48490000	0.00000000
25	547.51580000	0.59560000	0.00000000
26	586.83790000	10.99160000	0.00000000
27	594.94090000	2.53430000	0.00000000
28	638.84880000	9.13830000	0.00000000
29	639.03210000	0.15300000	0.00000000
30	701.97150000	2.66680000	0.00000000
31	724.26020000	44.74480000	0.00000000
32	770.23440000	3.79010000	0.00000000
33	786.27590000	37.00620000	0.00000000
34	803.27300000	2.19130000	0.00000000
35	820.79520000	1.99700000	0.00000000
36	835.40640000	5.98970000	0.00000000
37	843.97330000	5.80440000	0.00000000
38	854.93320000	2.41090000	0.00000000
39	862.33270000	70.22250000	0.00000000
40	869.06590000	0.11560000	0.00000000
41	907.68190000	1.60820000	0.00000000
42	934.85530000	29.68630000	0.00000000
43	955.43430000	0.41680000	0.00000000
44	976.05560000	3.51170000	0.00000000
45	989.32570000	1.63370000	0.00000000
46	994.30110000	0.12640000	0.00000000
47	1006.18860000	0.08400000	0.00000000
48	1007.76500000	1.63650000	0.00000000
49	1016.16110000	2.63670000	0.00000000
50	1019.59740000	9.66680000	0.00000000
51	1028.39390000	3.81680000	0.00000000
52	1036.78520000	0.01040000	0.00000000
53	1052.94470000	3.22290000	0.00000000
54	1056.60450000	1.59700000	0.00000000
55	1060.45520000	11.73320000	0.00000000
56	1063.89190000	9.22550000	0.00000000
57	1078.00880000	1.78210000	0.00000000
58	1122.23260000	5.14280000	0.00000000
59	1178.84630000	66.38760000	0.00000000
60	1193.58360000	24.99530000	0.00000000

61	1196.61530000	0.33890000	0.00000000
62	1205.24150000	6.82200000	0.00000000
63	1210.91670000	26.95800000	0.00000000
64	1214.75630000	0.10500000	0.00000000
65	1240.54500000	16.76740000	0.00000000
66	1257.08340000	2.20710000	0.00000000
67	1286.42430000	0.36170000	0.00000000
68	1295.10780000	4.74220000	0.00000000
69	1300.44200000	8.26140000	0.00000000
70	1327.63270000	8.76330000	0.00000000
71	1335.38720000	2.40560000	0.00000000
72	1347.43580000	6.61490000	0.00000000
73	1349.80270000	15.43760000	0.00000000
74	1357.11350000	51.33290000	0.00000000
75	1365.17910000	12.16080000	0.00000000
76	1372.19280000	5.71440000	0.00000000
77	1376.64150000	1.00500000	0.00000000
78	1394.47690000	1.76350000	0.00000000
79	1403.29100000	13.38660000	0.00000000
80	1405.23770000	12.09830000	0.00000000
81	1438.64590000	11.26150000	0.00000000
82	1493.11450000	4.14410000	0.00000000
83	1497.11760000	7.93290000	0.00000000
84	1504.31450000	2.29410000	0.00000000
85	1506.23120000	3.52040000	0.00000000
86	1513.51300000	19.74310000	0.00000000
87	1515.20050000	7.98100000	0.00000000
88	1517.46870000	16.97190000	0.00000000
89	1538.19860000	3.16610000	0.00000000
90	1541.34040000	5.51940000	0.00000000
91	1630.70210000	2.31560000	0.00000000
92	1632.13130000	42.42770000	0.00000000
93	1647.61250000	3.04830000	0.00000000
94	1651.60540000	11.89510000	0.00000000
95	3059.18050000	12.02390000	0.00000000
96	3065.05040000	9.46980000	0.00000000
97	3066.68730000	5.90450000	0.00000000
98	3074.69440000	5.12230000	0.00000000
99	3084.22420000	2.17380000	0.00000000
100	3086.87810000	7.51970000	0.00000000
101	3097.14150000	6.82330000	0.00000000
102	3100.49490000	4.72380000	0.00000000
103	3109.32410000	3.45430000	0.00000000
104	3126.86670000	0.61260000	0.00000000
105	3128.39690000	2.70960000	0.00000000
106	3138.13480000	4.03560000	0.00000000
107	3151.20870000	1.93430000	0.00000000
108	3153.89270000	1.96480000	0.00000000
109	3166.57410000	6.20330000	0.00000000
110	3169.85110000	6.32040000	0.00000000
111	3188.56300000	0.87980000	0.00000000
112	3198.08120000	4.10430000	0.00000000
113	3207.17730000	1.47730000	0.00000000
114	3222.10970000	4.72310000	0.00000000

S56. CALCULATIONS ON TS 10 (ISOMER 1; TRIPLET) ⟶ 17t

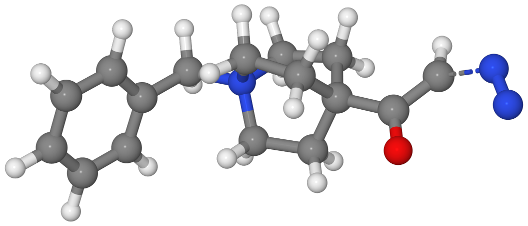

Route	:	# opt=(calcfc,ts,noeigentest) freq ub3lyp/cc-pvtz empiricaldispersion=gd3bj pop=regular geom=connectivity int=ultrafine
SMILES	:	c1ccc(cc1)C[N]23CCC(CC2)(CC3)C(=O)[CH]N=[N]
Formula	:	$C_{16}H_{20}N_3O^{+,3}$
Charge	:	1
Multiplicity	:	3
Energy	:	-861.24244530 a.u.
Gibbs Energy	:	-860.95461700 a.u.

Cartesian Co-ordinates (XYZ format)

40

C	-0.33989000	1.34490502	-0.50903898
C	0.24688400	-0.03326800	-0.18162601
C	-1.87449002	1.24229097	-0.52930897
H	-0.02394300	2.07996297	0.23004600
H	0.00165900	1.69194400	-1.48272097
H	-2.34853911	2.06181312	0.00376200
H	-2.27082300	1.21959603	-1.54143202
C	-0.13751601	-0.37745601	1.27085304
H	0.14050899	-1.40851998	1.47763002
H	0.41080499	0.25191501	1.97019303
C	-1.64493299	-0.16148899	1.46993506
H	-2.11225104	-0.98852003	1.99292898
H	-1.86537397	0.75728202	2.00887489
C	-1.90428698	-1.20271599	-0.73631197
H	-2.11302710	-2.10203099	-0.16618399
H	-2.54697108	-1.19903600	-1.61200202
C	-0.42069799	-1.06564403	-1.10383201
H	0.07890600	-2.02410603	-0.99624503
H	-0.31166399	-0.75447702	-2.14238405
N	-2.33207393	-0.03921800	0.13089199
C	-3.85476804	0.01431800	0.29054201
H	-4.23665905	0.22666000	-0.70569903
H	-4.04369402	0.87862599	0.92338502
C	1.77940094	-0.15391900	-0.30508199
O	2.28052092	-1.27525699	-0.24713901
C	2.58558989	1.01338196	-0.42154700
H	2.30151391	2.02831507	-0.64493698
N	4.23256207	0.97245598	0.22581901
N	4.85309315	0.03232100	0.20217501
C	-4.47846413	-1.22117198	0.86123002
C	-4.67243290	-1.34134603	2.23824191
C	-4.91203213	-2.24892402	0.02268600
C	-5.25923681	-2.48125100	2.76974106
H	-4.38394022	-0.53320497	2.89794993

C	-5.49832010	-3.38918090	0.55296701
H	-4.80923319	-2.15315795	-1.05023098
C	-5.66600800	-3.50922489	1.92766905
H	-5.41019392	-2.56143904	3.83668804
H	-5.83518314	-4.17691994	-0.10551900
H	-6.12803221	-4.39476395	2.34018207

Frequencies

Mode	IR frequency	IR intensity	Raman intensity
1	-502.63570000	71.99900000	0.00000000
2	18.19530000	0.34140000	0.00000000
3	29.98680000	1.90530000	0.00000000
4	39.99240000	1.19750000	0.00000000
5	51.60570000	0.83660000	0.00000000
6	77.26320000	1.18530000	0.00000000
7	88.58750000	0.84610000	0.00000000
8	114.76720000	4.00450000	0.00000000
9	123.94310000	1.16420000	0.00000000
10	162.57790000	2.10810000	0.00000000
11	172.24400000	1.99500000	0.00000000
12	211.63340000	1.76040000	0.00000000
13	238.42970000	0.58290000	0.00000000
14	246.64610000	1.32280000	0.00000000
15	301.64940000	1.27670000	0.00000000
16	322.81190000	0.32190000	0.00000000
17	335.44780000	8.43520000	0.00000000
18	378.56530000	0.79850000	0.00000000
19	398.03380000	1.27960000	0.00000000
20	418.02130000	0.03830000	0.00000000
21	422.23720000	0.03240000	0.00000000
22	437.49700000	1.44520000	0.00000000
23	468.96440000	4.96680000	0.00000000
24	475.24410000	3.12650000	0.00000000
25	531.98550000	3.10430000	0.00000000
26	542.42790000	0.33130000	0.00000000
27	550.76430000	1.19240000	0.00000000
28	588.40600000	9.43260000	0.00000000
29	638.73700000	7.52090000	0.00000000
30	639.05430000	0.12490000	0.00000000
31	699.56360000	5.13060000	0.00000000
32	708.95680000	5.29600000	0.00000000
33	724.27880000	44.80010000	0.00000000
34	763.81080000	16.46290000	0.00000000
35	786.54720000	34.43590000	0.00000000
36	807.85480000	0.80580000	0.00000000
37	833.61360000	9.52280000	0.00000000
38	842.83520000	6.42840000	0.00000000
39	851.04250000	46.78780000	0.00000000
40	855.22610000	2.47110000	0.00000000
41	869.12440000	0.09430000	0.00000000
42	907.49180000	1.04410000	0.00000000
43	949.49250000	21.33870000	0.00000000
44	958.45150000	4.96270000	0.00000000
45	983.08110000	2.07590000	0.00000000
46	988.83720000	1.67050000	0.00000000
47	999.86210000	1.32030000	0.00000000
48	1006.10160000	0.03870000	0.00000000
49	1012.65440000	3.01980000	0.00000000
50	1017.91540000	1.20460000	0.00000000
51	1028.43060000	4.10280000	0.00000000
52	1036.49110000	0.00390000	0.00000000
53	1039.48800000	10.62400000	0.00000000
54	1053.80250000	2.99270000	0.00000000
55	1056.54970000	1.40860000	0.00000000
56	1059.43250000	5.36890000	0.00000000
57	1067.71890000	15.59600000	0.00000000
58	1099.87030000	2.22500000	0.00000000
59	1122.13390000	5.18700000	0.00000000
60	1188.52960000	3.52300000	0.00000000

61	1196.50720000	0.15480000	0.00000000
62	1201.45780000	13.78050000	0.00000000
63	1205.20560000	0.69420000	0.00000000
64	1214.57020000	1.98510000	0.00000000
65	1239.39330000	90.80120000	0.00000000
66	1242.98480000	63.41970000	0.00000000
67	1258.79790000	23.35390000	0.00000000
68	1284.71190000	0.57020000	0.00000000
69	1297.95580000	11.96360000	0.00000000
70	1302.53750000	16.05130000	0.00000000
71	1329.22650000	1.01030000	0.00000000
72	1335.78070000	5.24940000	0.00000000
73	1347.70970000	8.81910000	0.00000000
74	1348.23560000	3.97410000	0.00000000
75	1362.68920000	4.64440000	0.00000000
76	1371.53560000	9.44840000	0.00000000
77	1376.56060000	0.82670000	0.00000000
78	1389.96780000	1.10800000	0.00000000
79	1403.34580000	11.14690000	0.00000000
80	1405.31910000	11.01040000	0.00000000
81	1438.82100000	10.80060000	0.00000000
82	1492.97580000	3.56180000	0.00000000
83	1497.12640000	7.80260000	0.00000000
84	1504.01620000	3.40620000	0.00000000
85	1506.42830000	3.14180000	0.00000000
86	1513.43800000	17.94380000	0.00000000
87	1515.02820000	8.18560000	0.00000000
88	1517.55180000	16.94430000	0.00000000
89	1538.26780000	3.17180000	0.00000000
90	1541.29370000	5.99930000	0.00000000
91	1548.50810000	33.13090000	0.00000000
92	1630.81610000	2.44120000	0.00000000
93	1647.82990000	2.98880000	0.00000000
94	1998.89050000	198.47070000	0.00000000
95	3058.87800000	11.58300000	0.00000000
96	3063.89610000	9.85890000	0.00000000
97	3068.50440000	5.48770000	0.00000000
98	3074.76110000	4.97550000	0.00000000
99	3083.54530000	2.82100000	0.00000000
100	3086.23150000	8.20090000	0.00000000
101	3096.32780000	7.59500000	0.00000000
102	3100.18830000	5.00510000	0.00000000
103	3112.15040000	2.65420000	0.00000000
104	3125.43020000	1.88800000	0.00000000
105	3127.11920000	0.76600000	0.00000000
106	3137.08990000	4.49020000	0.00000000
107	3150.57580000	1.82450000	0.00000000
108	3153.60740000	2.14920000	0.00000000
109	3166.88240000	6.12440000	0.00000000
110	3169.56470000	6.37360000	0.00000000
111	3188.37280000	0.91270000	0.00000000
112	3197.90530000	4.33150000	0.00000000
113	3207.05350000	1.61780000	0.00000000
114	3240.20090000	1.08820000	0.00000000

S57. CALCULATIONS ON 17t

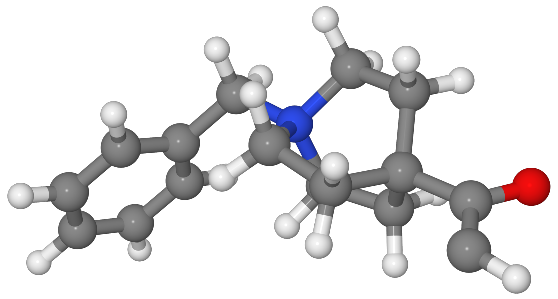

Route	:	# opt freq b3lyp/cc-pvtz empiricaldispersion=gd3bj pop=regular geom=connectivity int=ultrafine
SMILES	:	[CH]C(=O)C12CC[N](CC1)(CC2)Cc3ccccc3
Formula	:	$C_{16}H_{20}NO^{+,3}$
Charge	:	1
Multiplicity	:	3
Energy	:	-751.70031049 a.u.
Gibbs Energy	:	-751.41470500 a.u.

Cartesian Co-ordinates (XYZ format)

38

C	-1.54160798	-1.06105399	-0.91665798
C	-2.38218093	-0.18054600	0.01273200
C	-0.22177100	-0.33917800	-1.23565900
H	-1.34380996	-2.02347803	-0.44722801
H	-2.07429504	-1.26161802	-1.84447801
H	0.63647598	-0.99652201	-1.15368104
H	-0.22363500	0.10405400	-2.22883892
C	-1.65649199	-0.09326200	1.36985195
H	-2.15824294	0.63880301	1.99906397
H	-1.70233595	-1.05028999	1.88657403
C	-0.18614300	0.28764200	1.13951194
H	0.14250501	1.07577395	1.81113696
H	0.48691300	-0.55601698	1.25129402
C	-1.04418004	1.86590195	-0.52715498
H	-0.94662499	2.58710408	0.28079799
H	-0.77515602	2.35751009	-1.45803702
C	-2.44116211	1.23555601	-0.58282298
H	-3.14658499	1.84131598	-0.02005400
H	-2.79807806	1.18770802	-1.61094403
N	0.00129300	0.80169702	-0.26895699
C	1.38671994	1.42623103	-0.45860299
C	-3.79367399	-0.70379001	0.24506301
O	-4.62378216	-0.00965100	0.84207600
C	-4.18451977	-1.99139297	-0.18539099
H	-5.13336992	-2.49903893	-0.07457500
H	1.39217401	2.30340695	0.18470500
H	1.40801895	1.75910699	-1.49391305
C	2.54114199	0.52202398	-0.15632100
C	3.11721301	-0.24859600	-1.16750097
C	3.08343792	0.46998599	1.12848103
C	4.19433117	-1.07963598	-0.89264899

H	2.73815298	-0.18410601	-2.17925596
C	4.15999317	-0.36074001	1.40458000
H	2.67737103	1.09392905	1.91382396
C	4.71191978	-1.14155102	0.39558801
H	4.63684511	-1.66691101	-1.68443298
H	4.57598114	-0.38903400	2.40147710
H	5.55449486	-1.78366101	0.60913903

Frequencies

Mode	IR frequency	IR intensity	Raman intensity
1	21.97140000	3.78310000	0.00000000
2	37.25570000	0.03350000	0.00000000
3	60.57730000	0.62340000	0.00000000
4	79.35990000	1.06040000	0.00000000
5	115.57580000	0.19330000	0.00000000
6	142.75590000	3.51160000	0.00000000
7	149.02860000	0.74770000	0.00000000
8	219.59850000	0.68120000	0.00000000
9	235.07570000	0.81330000	0.00000000
10	245.57100000	0.67920000	0.00000000
11	311.25250000	2.62110000	0.00000000
12	321.17080000	1.71660000	0.00000000
13	349.74160000	11.55200000	0.00000000
14	364.59070000	0.65600000	0.00000000
15	397.64720000	0.88560000	0.00000000
16	418.47820000	0.07100000	0.00000000
17	422.37870000	0.06090000	0.00000000
18	455.23380000	18.72970000	0.00000000
19	485.92130000	4.14280000	0.00000000
20	529.51950000	5.69870000	0.00000000
21	542.67720000	0.29550000	0.00000000
22	559.58900000	8.19250000	0.00000000
23	562.75620000	10.70570000	0.00000000
24	600.37560000	21.01750000	0.00000000
25	639.14350000	0.08320000	0.00000000
26	646.47850000	24.77900000	0.00000000
27	707.16470000	0.37740000	0.00000000
28	724.61970000	44.92710000	0.00000000
29	747.11430000	8.47820000	0.00000000
30	786.39200000	34.03830000	0.00000000
31	805.77180000	0.50760000	0.00000000
32	833.21720000	7.20920000	0.00000000
33	841.01660000	12.93700000	0.00000000
34	850.48880000	29.73130000	0.00000000
35	854.42820000	2.08620000	0.00000000
36	869.69460000	0.09060000	0.00000000
37	905.20640000	0.90180000	0.00000000
38	922.04410000	17.14940000	0.00000000
39	954.89710000	6.30720000	0.00000000
40	963.44420000	18.94090000	0.00000000
41	988.81330000	0.79800000	0.00000000
42	996.49660000	0.13540000	0.00000000
43	1001.47660000	2.05840000	0.00000000
44	1006.27970000	0.00890000	0.00000000
45	1013.91830000	6.79710000	0.00000000
46	1018.35530000	1.85140000	0.00000000
47	1028.38670000	3.84600000	0.00000000
48	1036.24440000	0.01610000	0.00000000
49	1053.69540000	2.93840000	0.00000000
50	1056.53700000	1.65250000	0.00000000
51	1060.83420000	1.83080000	0.00000000
52	1067.29750000	20.33570000	0.00000000
53	1099.82330000	6.14190000	0.00000000
54	1122.36850000	5.15740000	0.00000000
55	1188.85830000	1.59050000	0.00000000
56	1196.39070000	0.05170000	0.00000000
57	1201.62020000	3.32220000	0.00000000
58	1204.20130000	0.34620000	0.00000000
59	1214.90370000	0.79320000	0.00000000
60	1240.94250000	18.44040000	0.00000000

61	1256.01060000	0.85380000	0.00000000
62	1276.20510000	34.62190000	0.00000000
63	1290.17980000	4.26030000	0.00000000
64	1300.88150000	46.06670000	0.00000000
65	1310.53160000	27.00020000	0.00000000
66	1328.39480000	24.94860000	0.00000000
67	1337.46570000	1.81770000	0.00000000
68	1345.93480000	3.09050000	0.00000000
69	1349.19900000	2.45840000	0.00000000
70	1365.43220000	8.32990000	0.00000000
71	1367.36350000	10.47260000	0.00000000
72	1378.06770000	1.12840000	0.00000000
73	1396.95020000	1.22300000	0.00000000
74	1403.26290000	12.84260000	0.00000000
75	1407.05120000	9.49600000	0.00000000
76	1438.17110000	9.07720000	0.00000000
77	1493.54250000	4.18520000	0.00000000
78	1496.97370000	7.36140000	0.00000000
79	1502.89080000	2.23210000	0.00000000
80	1504.74070000	3.17170000	0.00000000
81	1513.07290000	21.29310000	0.00000000
82	1514.73070000	6.64910000	0.00000000
83	1516.65520000	16.71960000	0.00000000
84	1528.43290000	63.10330000	0.00000000
85	1538.36450000	3.21410000	0.00000000
86	1541.00320000	3.42400000	0.00000000
87	1630.78320000	2.42120000	0.00000000
88	1647.99870000	2.85440000	0.00000000
89	3064.72560000	5.46360000	0.00000000
90	3066.26810000	9.04330000	0.00000000
91	3070.69530000	6.42580000	0.00000000
92	3075.27490000	4.66870000	0.00000000
93	3083.38930000	5.18790000	0.00000000
94	3086.38330000	4.84810000	0.00000000
95	3095.74110000	6.70660000	0.00000000
96	3104.83530000	5.01950000	0.00000000
97	3110.92730000	1.97270000	0.00000000
98	3118.41600000	1.48270000	0.00000000
99	3127.91140000	0.47220000	0.00000000
100	3137.93650000	4.16970000	0.00000000
101	3150.75010000	1.32220000	0.00000000
102	3154.08640000	2.92580000	0.00000000
103	3167.10560000	5.85810000	0.00000000
104	3169.57720000	5.98150000	0.00000000
105	3188.06610000	0.95360000	0.00000000
106	3197.62440000	4.46490000	0.00000000
107	3206.76330000	1.92530000	0.00000000
108	3206.95300000	10.06670000	0.00000000

S58. CALCULATIONS ON TS 17t ⟶ 18t

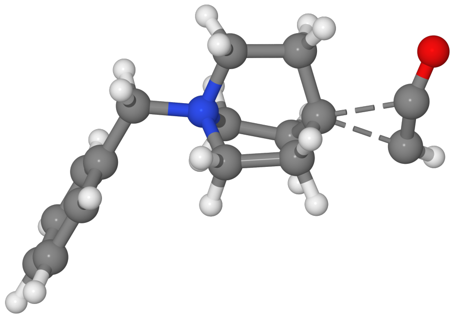

Route	:	# opt=(calcfc,ts,noeigentest) freq ub3lyp/cc-pvtz empiricaldispersion=gd3bj pop=regular geom=connectivity int=ultrafine
SMILES	:	[CH][C]=O.c1ccc(cc1)C[N]23CC[C](CC2)CC3
Formula	:	$C_{16}H_{20}NO^{+,3}$
Charge	:	1
Multiplicity	:	3
Energy	:	-751.61161138 a.u.
Gibbs Energy	:	-751.32904700 a.u.

Cartesian Co-ordinates (XYZ format)

38

C	-0.32467699	-0.57629502	-1.35734999
C	-0.85491598	-0.20011701	0.01226300
C	1.21477199	-0.68454802	-1.27017796
H	-0.73208898	-1.53006494	-1.68693995
H	-0.60956103	0.17453200	-2.09096098
H	1.56399596	-1.71073604	-1.22382605
H	1.70186996	-0.19435200	-2.10815191
C	-0.24654999	-1.12484598	1.06801295
H	-0.71033102	-0.97160602	2.03916502
H	-0.40109700	-2.16450405	0.78917998
C	1.26417100	-0.82281601	1.17988002
H	1.50697100	-0.23423600	2.06162810
H	1.86136699	-1.72793806	1.19103599
C	1.06586599	1.36155701	0.09090500
H	1.57641602	1.89553404	0.88799900
H	1.27423501	1.86460304	-0.85011399
C	-0.44969699	1.23117101	0.35224599
H	-0.67932701	1.45080197	1.39327097
H	-0.97692800	1.96202898	-0.25381801
N	1.70896494	-0.00679300	-0.00923500
C	3.22946405	0.17168701	-0.02828100
H	3.42254806	0.87324601	-0.83697498
H	3.46961308	0.65854001	0.91429800
C	-2.85933399	0.26530501	-0.37888801
O	-3.33953094	1.33264196	-0.15390199
C	-2.77499390	-1.01085603	0.11481800
H	-3.46686411	-1.57099605	0.76091200
C	4.01648378	-1.09015203	-0.20209000
C	4.44293690	-1.81462097	0.91215700
C	4.37037706	-1.53518403	-1.47651994

C	5.18114901	-2.97932196	0.75445598
H	4.21717501	-1.45727301	1.90854502
C	5.10805893	-2.69956899	-1.63545203
H	4.08622503	-0.96179497	-2.34910202
C	5.50803804	-3.42646408	-0.52009797
H	5.51187277	-3.52885509	1.62409401
H	5.38172007	-3.03212500	-2.62649798
H	6.08816481	-4.32981777	-0.64359099

Frequencies

Mode	IR frequency	IR intensity	Raman intensity
1	-754.68120000	169.03270000	0.00000000
2	31.87070000	2.39530000	0.00000000
3	47.32650000	1.26170000	0.00000000
4	60.81250000	0.72540000	0.00000000
5	84.98820000	0.12860000	0.00000000
6	124.64870000	0.80670000	0.00000000
7	134.41270000	1.84710000	0.00000000
8	160.62700000	8.23480000	0.00000000
9	167.89080000	1.36110000	0.00000000
10	225.68910000	3.91740000	0.00000000
11	237.65380000	1.10770000	0.00000000
12	267.08200000	0.94760000	0.00000000
13	307.65850000	1.15750000	0.00000000
14	323.42970000	3.50670000	0.00000000
15	371.25050000	3.04270000	0.00000000
16	379.35280000	3.94500000	0.00000000
17	405.00960000	2.94550000	0.00000000
18	418.63150000	0.09460000	0.00000000
19	423.51970000	0.03620000	0.00000000
20	440.02200000	41.11630000	0.00000000
21	466.37660000	5.85470000	0.00000000
22	527.19530000	1.20010000	0.00000000
23	539.24720000	0.55540000	0.00000000
24	568.76280000	16.48320000	0.00000000
25	627.31590000	3.19990000	0.00000000
26	639.14990000	0.11890000	0.00000000
27	685.28730000	12.37260000	0.00000000
28	724.38220000	46.34870000	0.00000000
29	739.17610000	50.31500000	0.00000000
30	785.95130000	29.53940000	0.00000000
31	796.71300000	29.48330000	0.00000000
32	808.95470000	7.55750000	0.00000000
33	832.78960000	4.34770000	0.00000000
34	837.33860000	2.79430000	0.00000000
35	855.56170000	1.97780000	0.00000000
36	869.43280000	0.09510000	0.00000000
37	884.22390000	79.08520000	0.00000000
38	901.66470000	2.54510000	0.00000000
39	919.39530000	42.06430000	0.00000000
40	952.96030000	2.66670000	0.00000000
41	969.50340000	3.93890000	0.00000000
42	983.79430000	3.92140000	0.00000000
43	988.10480000	2.46120000	0.00000000
44	1000.18280000	3.29250000	0.00000000
45	1006.12760000	0.01390000	0.00000000
46	1012.69310000	10.29010000	0.00000000
47	1017.12940000	2.11500000	0.00000000
48	1028.28810000	4.06600000	0.00000000
49	1032.81630000	0.85630000	0.00000000
50	1036.24360000	0.04510000	0.00000000
51	1054.25910000	0.83260000	0.00000000
52	1056.48680000	1.59180000	0.00000000
53	1070.85480000	17.97390000	0.00000000
54	1121.92410000	5.49420000	0.00000000
55	1174.31490000	8.88580000	0.00000000
56	1194.61930000	2.20780000	0.00000000
57	1196.95630000	0.40690000	0.00000000
58	1203.07480000	0.04450000	0.00000000
59	1214.51280000	1.52950000	0.00000000
60	1227.98200000	15.89040000	0.00000000

61	1240.61080000	16.03990000	0.00000000
62	1251.46030000	2.48780000	0.00000000
63	1271.35410000	6.35480000	0.00000000
64	1288.06220000	4.82950000	0.00000000
65	1295.95730000	13.39560000	0.00000000
66	1325.75480000	4.35180000	0.00000000
67	1327.99710000	2.12580000	0.00000000
68	1341.82960000	8.64170000	0.00000000
69	1345.99190000	1.27880000	0.00000000
70	1357.09020000	4.93820000	0.00000000
71	1360.52930000	5.51600000	0.00000000
72	1368.27860000	11.24450000	0.00000000
73	1375.47040000	0.99190000	0.00000000
74	1398.20510000	15.02730000	0.00000000
75	1401.29490000	12.34150000	0.00000000
76	1432.27140000	9.85600000	0.00000000
77	1492.28860000	3.08130000	0.00000000
78	1496.64620000	5.32910000	0.00000000
79	1500.62650000	6.90040000	0.00000000
80	1500.68930000	2.56860000	0.00000000
81	1508.82810000	19.65590000	0.00000000
82	1511.32680000	6.86160000	0.00000000
83	1513.01670000	18.93010000	0.00000000
84	1537.53730000	6.17260000	0.00000000
85	1538.29660000	4.28710000	0.00000000
86	1630.68740000	2.41590000	0.00000000
87	1647.93480000	2.86340000	0.00000000
88	1803.42060000	313.75710000	0.00000000
89	2944.93060000	34.22710000	0.00000000
90	3070.45310000	2.71730000	0.00000000
91	3074.00320000	6.79610000	0.00000000
92	3075.95060000	2.96190000	0.00000000
93	3076.70180000	3.91480000	0.00000000
94	3082.47130000	6.97480000	0.00000000
95	3086.13950000	6.13050000	0.00000000
96	3096.21380000	8.54670000	0.00000000
97	3111.50360000	2.06270000	0.00000000
98	3119.31810000	3.82500000	0.00000000
99	3121.70190000	1.93900000	0.00000000
100	3127.93070000	0.73970000	0.00000000
101	3139.90430000	4.01780000	0.00000000
102	3151.62650000	1.73940000	0.00000000
103	3154.70270000	3.14230000	0.00000000
104	3167.29170000	5.93430000	0.00000000
105	3169.52510000	6.48140000	0.00000000
106	3188.10990000	0.96890000	0.00000000
107	3197.62180000	4.49010000	0.00000000
108	3206.79970000	1.75510000	0.00000000

S59. CALCULATIONS ON 18t

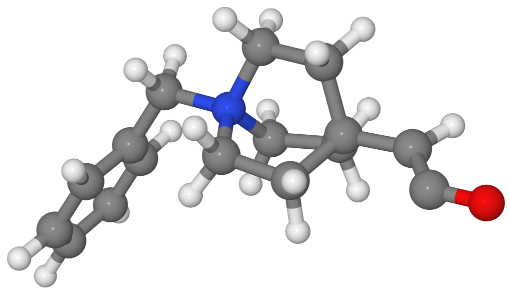

Route	:	# opt freq b3lyp/cc-pvtz empiricaldispersion=gd3bj pop=regular geom=connectivity int=ultrafine
SMILES	:	c1ccc(cc1)C[N]23CCC(CC2)(CC3)[CH][C]=O
Formula	:	$C_{16}H_{20}NO^{+,3}$
Charge	:	1
Multiplicity	:	3
Energy	:	-751.72669607 a.u.
Gibbs Energy	:	-751.43885100 a.u.

Cartesian Co-ordinates (XYZ format)

38

C	-2.34573698	-1.42486298	0.66807699
C	-2.36320901	-0.16792201	-0.23937000
C	-0.90146703	-1.92308199	0.82339400
H	-2.77523208	-1.17581701	1.63661802
H	-2.95665693	-2.21491694	0.23600300
H	-0.66115999	-2.16472912	1.85517299
H	-0.69812799	-2.79833293	0.21113800
C	-1.63859499	0.95982099	0.51028001
H	-1.48255002	1.80360603	-0.15998501
H	-2.25034499	1.32210004	1.33270800
C	-0.29733399	0.44364899	1.04942095
H	0.51380801	1.13872302	0.86439699
H	-0.33356199	0.23163700	2.11569405
C	-0.05991200	-0.68666798	-1.11739898
H	0.54642302	0.17264800	-1.38372898
H	0.37792999	-1.56684804	-1.57959104
C	-1.53703904	-0.50641102	-1.49983895
H	-1.62268698	0.29452899	-2.23106790
H	-1.92727995	-1.41138196	-1.96381295
N	0.07382400	-0.85835201	0.37716800
C	1.48864603	-1.30208194	0.76644701
C	-4.47563505	1.29022598	-0.06910400
O	-5.60129786	1.62011504	-0.28376001
C	-3.76333094	0.16947000	-0.60731000
H	-4.28400421	-0.48935801	-1.30093396
H	1.59995103	-2.29776692	0.34260601
H	1.46887195	-1.38917601	1.85049105
C	2.58764911	-0.39172599	0.31466001
C	3.20738196	-0.58785498	-0.92033702
C	3.03518009	0.63925898	1.14242303
C	4.23226023	0.25094500	-1.33410704

H	2.90362692	-1.41011596	-1.55490196
C	4.06046295	1.47870398	0.72964197
H	2.59726000	0.77494299	2.12284398
C	4.65478897	1.28943300	-0.51225603
H	4.70942116	0.08677200	-2.28961706
H	4.40347290	2.26978993	1.38087106
H	5.45728493	1.93882799	-0.83179301

Frequencies

Mode	IR frequency	IR intensity	Raman intensity
1	-3.40520000	3.98130000	0.00000000
2	38.21390000	0.16210000	0.00000000
3	63.47090000	0.56400000	0.00000000
4	79.13100000	1.31910000	0.00000000
5	115.29980000	0.04160000	0.00000000
6	125.39330000	2.23010000	0.00000000
7	166.32980000	0.11430000	0.00000000
8	201.14660000	2.01160000	0.00000000
9	224.50060000	1.08620000	0.00000000
10	242.99200000	0.12290000	0.00000000
11	267.83400000	0.72050000	0.00000000
12	291.16670000	1.48730000	0.00000000
13	348.67390000	1.66900000	0.00000000
14	365.05060000	2.24270000	0.00000000
15	380.32130000	1.23160000	0.00000000
16	410.68820000	0.18870000	0.00000000
17	418.09340000	0.04070000	0.00000000
18	422.26870000	0.02320000	0.00000000
19	435.38000000	1.42980000	0.00000000
20	471.37580000	3.92930000	0.00000000
21	537.56940000	0.23690000	0.00000000
22	541.75830000	0.40990000	0.00000000
23	576.96850000	7.18160000	0.00000000
24	628.51650000	1.45530000	0.00000000
25	639.00740000	0.12410000	0.00000000
26	690.10210000	13.16220000	0.00000000
27	724.50060000	42.67060000	0.00000000
28	745.19890000	13.03700000	0.00000000
29	762.99150000	13.80070000	0.00000000
30	786.37400000	33.87350000	0.00000000
31	802.15590000	0.99590000	0.00000000
32	832.33320000	3.44350000	0.00000000
33	836.95770000	7.93810000	0.00000000
34	851.91560000	4.10970000	0.00000000
35	855.50640000	49.63550000	0.00000000
36	869.41340000	0.11130000	0.00000000
37	904.73290000	1.42660000	0.00000000
38	955.14800000	2.37850000	0.00000000
39	957.42490000	6.31660000	0.00000000
40	978.40950000	1.55090000	0.00000000
41	988.33540000	0.49220000	0.00000000
42	1006.16170000	2.22830000	0.00000000
43	1006.82340000	2.60250000	0.00000000
44	1015.88700000	2.42540000	0.00000000
45	1018.84210000	2.91880000	0.00000000
46	1028.33660000	4.25160000	0.00000000
47	1036.76800000	0.04570000	0.00000000
48	1046.83090000	5.27810000	0.00000000
49	1056.40230000	1.19970000	0.00000000
50	1056.66020000	4.42010000	0.00000000
51	1065.58390000	1.94620000	0.00000000
52	1072.44640000	39.17970000	0.00000000
53	1122.16120000	5.79850000	0.00000000
54	1155.57620000	2.10090000	0.00000000
55	1179.48040000	3.36960000	0.00000000
56	1196.59680000	0.05870000	0.00000000
57	1204.01160000	1.36430000	0.00000000
58	1214.93910000	1.31000000	0.00000000
59	1218.03390000	2.31670000	0.00000000
60	1240.88410000	18.14260000	0.00000000

61	1254.88410000	1.61570000	0.00000000
62	1277.89510000	7.47360000	0.00000000
63	1289.46160000	2.74430000	0.00000000
64	1302.37760000	3.74360000	0.00000000
65	1325.45670000	1.70450000	0.00000000
66	1330.95320000	7.02190000	0.00000000
67	1344.81790000	11.39480000	0.00000000
68	1348.44180000	6.13420000	0.00000000
69	1358.31890000	7.07830000	0.00000000
70	1364.20810000	11.20680000	0.00000000
71	1368.51570000	4.46010000	0.00000000
72	1378.94550000	1.27220000	0.00000000
73	1395.17610000	0.39700000	0.00000000
74	1404.09130000	12.95970000	0.00000000
75	1408.21830000	10.73830000	0.00000000
76	1438.28140000	10.41180000	0.00000000
77	1492.84240000	4.24190000	0.00000000
78	1497.04110000	6.92100000	0.00000000
79	1501.55760000	1.00770000	0.00000000
80	1502.92020000	5.56320000	0.00000000
81	1511.20160000	21.29110000	0.00000000
82	1513.28150000	14.31900000	0.00000000
83	1515.43830000	7.84510000	0.00000000
84	1538.23860000	3.25700000	0.00000000
85	1539.87050000	6.54140000	0.00000000
86	1630.66610000	2.45420000	0.00000000
87	1647.65990000	3.11300000	0.00000000
88	1783.49780000	119.52250000	0.00000000
89	3063.76160000	7.89240000	0.00000000
90	3069.44380000	8.75680000	0.00000000
91	3071.73950000	4.47000000	0.00000000
92	3075.20010000	3.54420000	0.00000000
93	3083.40640000	3.91090000	0.00000000
94	3086.33760000	6.62450000	0.00000000
95	3090.48960000	8.45420000	0.00000000
96	3096.96430000	5.82170000	0.00000000
97	3106.79280000	2.40880000	0.00000000
98	3108.69840000	3.66600000	0.00000000
99	3116.04510000	2.23630000	0.00000000
100	3127.69010000	0.30930000	0.00000000
101	3137.37640000	4.38910000	0.00000000
102	3151.84260000	1.20220000	0.00000000
103	3154.97380000	2.86850000	0.00000000
104	3167.73220000	5.62280000	0.00000000
105	3168.94160000	6.51180000	0.00000000
106	3188.48350000	0.89170000	0.00000000
107	3197.98280000	4.14470000	0.00000000
108	3207.11800000	1.50240000	0.00000000
